# Genetic associations at 53 loci highlight cell types and biological pathways relevant for kidney function

**DOI:** 10.1038/ncomms10023

**Published:** 2016-01-21

**Authors:** Cristian Pattaro, Alexander Teumer, Mathias Gorski, Audrey Y. Chu, Man Li, Vladan Mijatovic, Maija Garnaas, Adrienne Tin, Rossella Sorice, Yong Li, Daniel Taliun, Matthias Olden, Meredith Foster, Qiong Yang, Ming-Huei Chen, Tune H. Pers, Andrew D. Johnson, Yi-An Ko, Christian Fuchsberger, Bamidele Tayo, Michael Nalls, Mary F. Feitosa, Aaron Isaacs, Abbas Dehghan, Pio d’Adamo, Adebowale Adeyemo, Aida Karina Dieffenbach, Alan B. Zonderman, Ilja M. Nolte, Peter J. van der Most, Alan F. Wright, Alan R. Shuldiner, Alanna C. Morrison, Albert Hofman, Albert V. Smith, Albert W. Dreisbach, Andre Franke, Andre G. Uitterlinden, Andres Metspalu, Anke Tonjes, Antonio Lupo, Antonietta Robino, Åsa Johansson, Ayse Demirkan, Barbara Kollerits, Barry I. Freedman, Belen Ponte, Ben A. Oostra, Bernhard Paulweber, Bernhard K. Krämer, Braxton D. Mitchell, Brendan M. Buckley, Carmen A. Peralta, Caroline Hayward, Catherine Helmer, Charles N. Rotimi, Christian M. Shaffer, Christian Müller, Cinzia Sala, Cornelia M. van Duijn, Aude Saint-Pierre, Daniel Ackermann, Daniel Shriner, Daniela Ruggiero, Daniela Toniolo, Yingchang Lu, Daniele Cusi, Darina Czamara, David Ellinghaus, David S. Siscovick, Douglas Ruderfer, Christian Gieger, Harald Grallert, Elena Rochtchina, Elizabeth J. Atkinson, Elizabeth G. Holliday, Eric Boerwinkle, Erika Salvi, Erwin P. Bottinger, Federico Murgia, Fernando Rivadeneira, Florian Ernst, Florian Kronenberg, Frank B. Hu, Gerjan J. Navis, Gary C. Curhan, George B. Ehret, Georg Homuth, Stefan Coassin, Gian-Andri Thun, Giorgio Pistis, Giovanni Gambaro, Giovanni Malerba, Grant W. Montgomery, Gudny Eiriksdottir, Gunnar Jacobs, Guo Li, H-Erich Wichmann, Harry Campbell, Helena Schmidt, Henri Wallaschofski, Henry Völzke, Hermann Brenner, Heyo K. Kroemer, Holly Kramer, Honghuang Lin, I. Mateo Leach, Ian Ford, Idris Guessous, Igor Rudan, Inga Prokopenko, Ingrid Borecki, Iris M. Heid, Ivana Kolcic, Ivana Persico, J. Wouter Jukema, James F. Wilson, Janine F. Felix, Jasmin Divers, Jean-Charles Lambert, Jeanette M. Stafford, Jean-Michel Gaspoz, Jennifer A. Smith, Jessica D. Faul, Jie Jin Wang, Jingzhong Ding, Joel N. Hirschhorn, John Attia, John B. Whitfield, John Chalmers, Jorma Viikari, Josef Coresh, Joshua C. Denny, Juha Karjalainen, Jyotika K. Fernandes, Karlhans Endlich, Katja Butterbach, Keith L. Keene, Kurt Lohman, Laura Portas, Lenore J. Launer, Leo-Pekka Lyytikäinen, Loic Yengo, Lude Franke, Luigi Ferrucci, Lynda M. Rose, Lyudmyla Kedenko, Madhumathi Rao, Maksim Struchalin, Marcus E. Kleber, Margherita Cavalieri, Margot Haun, Marilyn C. Cornelis, Marina Ciullo, Mario Pirastu, Mariza de Andrade, Mark A. McEvoy, Mark Woodward, Martin Adam, Massimiliano Cocca, Matthias Nauck, Medea Imboden, Melanie Waldenberger, Menno Pruijm, Marie Metzger, Michael Stumvoll, Michele K. Evans, Michele M. Sale, Mika Kähönen, Mladen Boban, Murielle Bochud, Myriam Rheinberger, Niek Verweij, Nabila Bouatia-Naji, Nicholas G. Martin, Nick Hastie, Nicole Probst-Hensch, Nicole Soranzo, Olivier Devuyst, Olli Raitakari, Omri Gottesman, Oscar H. Franco, Ozren Polasek, Paolo Gasparini, Patricia B. Munroe, Paul M. Ridker, Paul Mitchell, Paul Muntner, Christa Meisinger, Johannes H. Smit, ICBP Consortium, Goncalo R. Abecasis, Linda S. Adair, Myriam Alexander, David Altshuler, Najaf Amin, Dan E. Arking, Pankaj Arora, Yurii Aulchenko, Stephan J. L. Bakker, Stefania Bandinelli, Ines Barroso, Jacques S. Beckmann, John P. Beilby, Richard N. Bergman, Sven Bergmann, Joshua C. Bis, Michael Boehnke, Lori L. Bonnycastle, Stefan R. Bornstein, Michiel L. Bots, Jennifer L. Bragg-Gresham, Stefan-Martin Brand, Eva Brand, Peter S. Braund, Morris J. Brown, Paul R. Burton, Juan P. Casas, Mark J. Caulfield, Aravinda Chakravarti, John C. Chambers, Giriraj R. Chandak, Yen-Pei C. Chang, Fadi J. Charchar, Nish Chaturvedi, Yoon Shin Cho, Robert Clarke, Francis S. Collins, Rory Collins, John M. Connell, Jackie A. Cooper, Matthew N. Cooper, Richard S. Cooper, Anna Maria Corsi, Marcus Dörr, Santosh Dahgam, John Danesh, George Davey Smith, Ian N. M. Day, Panos Deloukas, Matthew Denniff, Anna F. Dominiczak, Yanbin Dong, Ayo Doumatey, Paul Elliott, Roberto Elosua, Jeanette Erdmann, Susana Eyheramendy, Martin Farrall, Cristiano Fava, Terrence Forrester, F. Gerald R. Fowkes, Ervin R. Fox, Timothy M. Frayling, Pilar Galan, Santhi K. Ganesh, Melissa Garcia, Tom R. Gaunt, Nicole L. Glazer, Min Jin Go, Anuj Goel, Jürgen Grässler, Diederick E. Grobbee, Leif Groop, Simonetta Guarrera, Xiuqing Guo, David Hadley, Anders Hamsten, Bok-Ghee Han, Rebecca Hardy, Anna-Liisa Hartikainen, Simon Heath, Susan R. Heckbert, Bo Hedblad, Serge Hercberg, Dena Hernandez, Andrew A. Hicks, Gina Hilton, Aroon D. Hingorani, Judith A Hoffman Bolton, Jemma C. Hopewell, Philip Howard, Steve E. Humphries, Steven C. Hunt, Kristian Hveem, M. Arfan Ikram, Muhammad Islam, Naoharu Iwai, Marjo-Riitta Jarvelin, Anne U. Jackson, Tazeen H. Jafar, Charles S. Janipalli, Toby Johnson, Sekar Kathiresan, Kay-Tee Khaw, Hyung-Lae Kim, Sanjay Kinra, Yoshikuni Kita, Mika Kivimaki, Jaspal S. Kooner, M. J. Kranthi Kumar, Diana Kuh, Smita R. Kulkarni, Meena Kumari, Johanna Kuusisto, Tatiana Kuznetsova, Markku Laakso, Maris Laan, Jaana Laitinen, Edward G. Lakatta, Carl D. Langefeld, Martin G. Larson, Mark Lathrop, Debbie A. Lawlor, Robert W. Lawrence, Jong-Young Lee, Nanette R. Lee, Daniel Levy, Yali Li, Will T. Longstreth, Jian’an Luan, Gavin Lucas, Barbara Ludwig, Massimo Mangino, K. Radha Mani, Michael G. Marmot, Francesco U. S. Mattace-Raso, Giuseppe Matullo, Wendy L. McArdle, Colin A. McKenzie, Thomas Meitinger, Olle Melander, Pierre Meneton, James F. Meschia, Tetsuro Miki, Yuri Milaneschi, Karen L. Mohlke, Vincent Mooser, Mario A. Morken, Richard W. Morris, Thomas H. Mosley, Samer Najjar, Narisu Narisu, Christopher Newton-Cheh, Khanh-Dung Hoang Nguyen, Peter Nilsson, Fredrik Nyberg, Christopher J. O’Donnell, Toshio Ogihara, Takayoshi Ohkubo, Tomonori Okamura, RickTwee-Hee Ong, Halit Ongen, N. Charlotte Onland-Moret, Paul F. O’Reilly, Elin Org, Marco Orru, Walter Palmas, Jutta Palmen, Lyle J. Palmer, Nicholette D. Palmer, Alex N. Parker, John F. Peden, Leena Peltonen, Markus Perola, Vasyl Pihur, Carl G. P. Platou, Andrew Plump, Dorairajan Prabhakaran, Bruce M. Psaty, Leslie J. Raffel, Dabeeru C. Rao, Asif Rasheed, Fulvio Ricceri, Kenneth M. Rice, Annika Rosengren, Jerome I. Rotter, Megan E. Rudock, Siim Sõber, Tunde Salako, Danish Saleheen, Veikko Salomaa, Nilesh J. Samani, Steven M. Schwartz, Peter E. H. Schwarz, Laura J. Scott, James Scott, Angelo Scuteri, Joban S. Sehmi, Mark Seielstad, Sudha Seshadri, Pankaj Sharma, Sue Shaw-Hawkins, Gang Shi, Nick R. G. Shrine, Eric J. G. Sijbrands, Xueling Sim, Andrew Singleton, Marketa Sjögren, Nicholas L. Smith, Maria Soler Artigas, Tim D. Spector, Jan A. Staessen, Alena Stancakova, Nanette I. Steinle, David P. Strachan, Heather M. Stringham, Yan V. Sun, Amy J. Swift, Yasuharu Tabara, E-Shyong Tai, Philippa J. Talmud, Andrew Taylor, Janos Terzic, Dag S. Thelle, Martin D. Tobin, Maciej Tomaszewski, Vikal Tripathy, Jaakko Tuomilehto, Ioanna Tzoulaki, Manuela Uda, Hirotsugu Ueshima, Cuno S. P. M. Uiterwaal, Satoshi Umemura, Pim van der Harst, Yvonne T. van der Schouw, Wiek H. van Gilst, Erkki Vartiainen, Ramachandran S. Vasan, Gudrun Veldre, Germaine C. Verwoert, Margus Viigimaa, D. G. Vinay, Paolo Vineis, Benjamin F. Voight, Peter Vollenweider, Lynne E. Wagenknecht, Louise V. Wain, Xiaoling Wang, Thomas J. Wang, Nicholas J. Wareham, Hugh Watkins, Alan B. Weder, Peter H. Whincup, Kerri L. Wiggins, Jacqueline C. M. Witteman, Andrew Wong, Ying Wu, Chittaranjan S. Yajnik, Jie Yao, J. H. Young, Diana Zelenika, Guangju Zhai, Weihua Zhang, Feng Zhang, Jing Hua Zhao, Haidong Zhu, Xiaofeng Zhu, Paavo Zitting, Ewa Zukowska-Szczechowska, AGEN Consortium, Yukinori Okada, Jer-Yuarn Wu, Dongfeng Gu, Fumihiko Takeuchi, Atsushi Takahashi, Shiro Maeda, Tatsuhiko Tsunoda, Peng Chen, Su-Chi Lim, Tien-Yin Wong, Jianjun Liu, Terri L. Young, Tin Aung, Yik-Ying Teo, Young Jin Kim, Daehee Kang, Chien-Hsiun Chen, Fuu-Jen Tsai, Li-Ching Chang, S. -J. Cathy Fann, Hao Mei, James E. Hixson, Shufeng Chen, Tomohiro Katsuya, Masato Isono, Eva Albrecht, Kazuhiko Yamamoto, Michiaki Kubo, Yusuke Nakamura, Naoyuki Kamatani, Norihiro Kato, Jiang He, Yuan-Tsong Chen, Toshihiro Tanaka, CARDIOGRAM, Muredach P Reilly, Heribert Schunkert, Themistocles L. Assimes, Alistair Hall, Christian Hengstenberg, Inke R. König, Reijo Laaksonen, Ruth McPherson, John R. Thompson, Unnur Thorsteinsdottir, Andreas Ziegler, Devin Absher, Li Chen, L. Adrienne Cupples13, Eran Halperin, Mingyao Li, Kiran Musunuru, Michael Preuss, Arne Schillert, Gudmar Thorleifsson, George A. Wells, Hilma Holm, Robert Roberts, Alexandre F. R. Stewart, Stephen Fortmann, Alan Go, Mark Hlatky, Carlos Iribarren, Joshua Knowles, Richard Myers, Thomas Quertermous, Steven Sidney, Neil Risch, Hua Tang, Stefan Blankenberg, Renate Schnabel, Christoph Sinning, Karl J. Lackner, Laurence Tiret, Viviane Nicaud, Francois Cambien, Christoph Bickel, Hans J. Rupprecht, Claire Perret, Carole Proust, Thomas F. Münzel, Maja Barbalic, Ida Yii-Der Chen, Serkalem Demissie-Banjaw, Aaron Folsom, Thomas Lumley, Kristin Marciante, Kent D. Taylor, Kelly Volcik, Solveig Gretarsdottir, Jeffrey R. Gulcher, Augustine Kong, Kari Stefansson, Gudmundur Thorgeirsson, Karl Andersen, Marcus Fischer, Anika Grosshennig, Patrick Linsel-Nitschke, Klaus Stark, Stefan Schreiber, Zouhair Aherrahrou, Petra Bruse, Angela Doering, Norman Klopp, Patrick Diemert, Christina Loley, Anja Medack, Janja Nahrstedt, Annette Peters, Arnika K. Wagner, Christina Willenborg, Bernhard O. Böhm, Harald Dobnig, Tanja B. Grammer, Michael M. Hoffmann, Andreas Meinitzer, Bernhard R. Winkelmann, Stefan Pilz, Wilfried Renner, Hubert Scharnagl, Tatjana Stojakovic, Andreas Tomaschitz, Karl Winkler, Candace Guiducci, Noel Burtt, Stacey B. Gabriel, Sonny Dandona, Olga Jarinova, Liming Qu, Robert Wilensky, William Matthai, Hakon H. Hakonarson, Joe Devaney, Mary Susan Burnett, Augusto D. Pichard, Kenneth M. Kent, Lowell Satler, Joseph M. Lindsay, Ron Waksman, Christopher W. Knouff, Dawn M. Waterworth, Max C. Walker, Stephen E. Epstein, Daniel J. Rader, Christopher P. Nelson, Benjamin J. Wright, Anthony J. Balmforth, Stephen G. Ball, CHARGe-Heart Failure Group, Laura R. Loehr, Wayne D. Rosamond, Emelia Benjamin, Talin Haritunians, David Couper, Joanne Murabito, Ying A. Wang, Bruno H. Stricker, Patricia P. Chang, James T. Willerson, ECHOGen Consortium, Stephan B. Felix, Norbert Watzinger, Jayashri Aragam, Robert Zweiker, Lars Lind, Richard J. Rodeheffer, Karin Halina Greiser, Jaap W. Deckers, Jan Stritzke, Erik Ingelsson, Iftikhar Kullo, Johannes Haerting, Thorsten Reffelmann, Margaret M. Redfield, Karl Werdan, Gary F. Mitchell, Donna K. Arnett, John S. Gottdiener, Maria Blettner, Nele Friedrich, Peter Kovacs, Philipp S. Wild, Philippe Froguel, Rainer Rettig, Reedik Mägi, Reiner Biffar, Reinhold Schmidt, Rita P. S. Middelberg, Robert J. Carroll, Brenda W. Penninx, Rodney J. Scott, Ronit Katz, Sanaz Sedaghat, Sarah H. Wild, Sharon L. R. Kardia, Sheila Ulivi, Shih-Jen Hwang, Stefan Enroth, Stefan Kloiber, Stella Trompet, Benedicte Stengel, Stephen J. Hancock, Stephen T. Turner, Sylvia E. Rosas, Sylvia Stracke, Tamara B. Harris, Tanja Zeller, Tatijana Zemunik, Terho Lehtimäki, Thomas Illig, Thor Aspelund, Tiit Nikopensius, Tonu Esko, Toshiko Tanaka, Ulf Gyllensten, Uwe Völker, Valur Emilsson, Veronique Vitart, Ville Aalto, Vilmundur Gudnason, Vincent Chouraki, Wei-Min Chen, Wilmar Igl, Winfried März, Wolfgang Koenig, Wolfgang Lieb, Ruth J. F. Loos, Yongmei Liu, Harold Snieder, Peter P. Pramstaller, Afshin Parsa, Jeffrey R. O’Connell, Katalin Susztak, Pavel Hamet, Johanne Tremblay, Ian H. de Boer, Carsten A. Böger, Wolfram Goessling, Daniel I. Chasman, Anna Köttgen, W. H. Linda Kao, Caroline S. Fox

**Affiliations:** 1 Center for Biomedicine, European Academy of Bozen/Bolzano (EURAC), affiliated to the University of Lübeck, Via Galvani 31, Bolzano 39100, Italy; 2 Interfaculty Institute for Genetics and Functional Genomics, Ernst-Moritz-Arndt-University Greifswald, Friedrich-Loeffler-Straße 15a, Greifswald 17487, Germany; 3 Institute for Community Medicine, University of Greifswald, Walther-Rathenau-Strasse 48, Greifswald 17487, Germany; 4 Department of Genetic Epidemiology, Institute of Epidemiology and Preventive Medicine, University of Regensburg, Franz-Josef-Strauß-Allee 11, Regensburg 93053, Germany; 5 Department of Nephrology, University Hospital Regensburg, Franz-Josef-Strauß-Allee 11, Regensburg 93053, Germany; 6 Preventive Medicine, Brigham and Women’s Hospital, 900 Commonwealth Avenue East, Boston, Massachusetts 02215, USA; 7 Department of Epidemiology, Johns Hopkins Bloomberg School of Public Health, 615 North Wolfe Street, Baltimore, Maryland 21205, USA; 8 Department of Life and Reproduction Sciences, University of Verona, Strada Le Grazie 8, Verona 37134, Italy; 9 Division of Genetics, Department of Medicine, Brigham and Women’s Hospital, Harvard Medical School, New Research Building77 Avenue Louis Pasteur, Room 458, Boston, Massachusetts 02115, USA; 10 Institute of Genetics and Biophysics "Adriano Buzzati-Traverso"—CNR, Via P. Castellino 111, Napoli 80131, Italy; 11 Department of Internal Medicine IV, University Hospital Freiburg, Berliner Allee 29, Freiburg 79110, Germany; 12 Division of Nephrology/Tufts Evidence Practice Center, Tufts University School of Medicine, Tufts Medical Center, Boston, Massachusetts 02111, USA; 13 Department of Biostatistics, Boston University School of Public Health, 715 Albany Street, Boston, Massachusetts 02118, USA; 14 Department of Neurology, Boston University School of Medicine, 72 East Concord ST B603, Boston, Massachusetts 02118, USA; 15 Division of Endocrinology and Center for Basic and Translational Obesity Research, Boston Children’s Hospital, 300 Longwood Avenue, Boston, Massachusetts 02115, USA; 16 Medical and Population Genetics Program, Broad Institute of MIT and Harvard, Cambridge, Massachusetts 2142, USA; 17 NHLBI's Framingham Heart Study and the Center for Population Studies, 73 Mt Wayte Avenue, Suite 2, Framingham, Massachusetts 01702, USA; 18 Renal Electrolyte and Hypertension Division, Perelman School of Medicine, University of Pennsylvania, 415 Curie Boulevard, 405B Clinical Research Building, Philadelphia, Pennsylvania 19104-4539, USA; 19 Department of Public Health Sciences, Loyola Medical Center, 2160 S First Avenue, Maywood, Illinois 60153, USA; 20 Laboratory of Neurogenetics, Building 35—Porter Building, 1A1015, National Institute on Aging/NIH, Bethesda, Maryland 20892, USA; 21 Division of Statistical Genomics, Washington University School of Medicine, 4444 Forest Park Boulevard, Box 8506, St Louis, Missouri 63108, USA; 22 Genetic Epidemiology Unit, Department of Epidemiology, Erasmus University Medical Center, Dr Molewaterplein 50, PO Box 2040, Rotterdam 3000 CA, The Netherlands; 23 Centre for Medical Systems Biology Leiden, Dr Molewaterplein 50, PO Box 2040, Rotterdam 3000 CA, The Netherlands; 24 Department of Epidemiology, Erasmus University Medical Center, PO Box 2040, Rotterdam 3000 CA, The Netherlands; 25 Institute for Maternal and Child Health—IRCCS "Burlo Garofolo" and University of Trieste, via dell'Istria 65/1, Trieste 34137, Italy; 26 Center for Research on Genomics and Global Health, National Human Genome Research Institute, Building 12A, Room 4047, 12 South Dr, MSC 5635, Bethesda, Maryland 20892-5635, USA; 27 Division of Clinical Epidemiology and Aging Research, German Cancer Research Center (DKFZ), Im Neuenheimer Feld 581, Heidelberg 69120, Germany; 28 German Cancer Consortium (DKTK), Im Neuenheimer Feld 581, Heidelberg 69120, Germany; 29 Laboratory of Personality and Cognition, National Institute on Aging, National Institutes of Health, NIH Biomedical Center, 251 Bayview Boulevard, Suite 100, Baltimore, Maryland 21224, USA; 30 Unit of Genetic Epidemiology and Bioinformatics, Department of Epidemiology, University Medical Center Groningen, PO Box 30001, Groningen 9700 RB, The Netherlands; 31 MRC Human Genetics Unit, Institute of Genetics and Molecular Medicine, University of Edinburgh, Crewe Road, Edinburgh EH4 2XU, UK; 32 Department of Medicine, University of Maryland School of Medicine, 685 West Baltimore Street, Baltimore, Maryland 21201, USA; 33 Geriatric Research and Education Clinical Center, Veterans Administration Medical Center, 10 North Greene Street, Baltimore, Maryland 21201, USA; 34 Human Genetics Center, University of Texas Health Science Center at Houston, 1200 Pressler St Suite 453E, Houston, Texas 77030, USA; 35 Icelandic Heart Association, Research Institute, Holtasmari 1, Kopavogur 201, Iceland; 36 University of Iceland, Sæmundargötu 2, Reykjavik 101, Iceland; 37 Division of Nephrology, University of Mississippi, 2500 North State Street, Jackson, Mississippi 39216, USA; 38 Institute of Clinical Molecular Biology, Christian-Albrechts University of Kiel, Schittenhelmstraße 12, Kiel 24105, Germany; 39 Department of Internal Medicine, Erasmus University Medical Center, PO Box 1738, Rotterdam 3000 DR, The Netherlands; 40 Estonian Genome Center of University of Tartu (EGCUT), Riia 23B, Tartu 51010, Estonia; 41 Institute of Molecular and Cell Biology, University of Tartu and Estonian Biocenter, Riia 23, Tartu 51010, Estonia; 42 Department of Medicine, University of Leipzig, Liebigstraße 18, Leipzig 04103, Germany; 43 Division of Nephrology, Department of Medicine, University of Verona, Piazzale Aristide Stefani 1, Verona 37126, Italy; 44 Uppsala University, Department of Immunology, Genetics and Pathology, Biomedical Center, SciLifeLab, Uppsala University, Uppsala SE- 75108, Sweden; 45 Innsbruck Medical University, Division of Genetic Epidemiology, Schoepfstraße 41, Innsbruck 6020, Austria; 46 Internal Medicine Department, Wake Forest School of Medicine, Medical Center Boulevard, Winston-Salem, North Carolina 27157-1053, USA; 47 Nephrology Division, Department of Specialties of Internal Medicine, Geneva University Hospital, 4 rue Gabrielle-Perret-Gentil, Geneve 1211, Switzerland; 48 Department of Clinical Genetics, Erasmus University Medical Center, Dr Molewaterplein 50, PO Box 2040, Rotterdam 3000 CA, The Netherlands; 49 First Department of Internal Medicine, Paracelsus Medical University/Salzburger Landeskliniken, Müllner Hauptstraße 48, Salzburg 5020, Austria; 50 University Medical Centre Mannheim, 5th Department of Medicine, University of Heidelberg, Theodor Kutzer Ufer 1-3, Mannheim 68167, Germany; 51 Department of Pharmacology and Therapeutics, University College Cork, Clinical Investigations Building, Western Rd, Cork, Ireland; 52 Division of Nephrology, University of California, San Francisco Medical School and San Francisco VA Medical Center, 4150 Clement Street, San Francisco, California 94121, USA; 53 INSERM, ISPED, Centre INSERM U897—Epidemiologie-Biostatistique, Bordeaux F-33000, France; 54 Université Bordeaux, ISPED, Centre INSERM U897-Epidemiologie-Biostatistique, Bordeaux F-33000, France; 55 Vanderbilt University School of Medicine, 2215-B Garland Avenue 1224—MRB4 (Light Hall)Nashville, Tennessee 37232, USA; 56 University Heart Center Hamburg, Clinic for general and interventional cardiology, Martinistraße 52, Hamburg 20246, Germany; 57 German Center for Cardiovascular Research (DZHK), Partner Site Hamburg/Lübeck/Kiel, Martinistraße 52, Hamburg 20246, Germany; 58 Division of Genetics and Cell Biology, San Raffaele Scientific Institute, Via Olgettina 58, Milano 20132, Italy; 59 INSERM U1078, Etablissement Français du Sang, 46 rue Félix Le Dantec, CS 51819, Brest Cedex 2 29218, France; 60 Institute of Molecular Genetics-CNR, Via Abbiategrasso 207, Pavia 27100, Italy; 61 The Charles Bronfman Institute for Personalized Medicine, Icahn School of Medicine at Mount Sinai, New York, New York 10029, USA; 62 Department of Health Sciences, University of Milano, Via Antonio di Rudinì 8, Milano 20142, Italy; 63 Max Planck Institute of Psychiatry, Kraepelinstraße 2–10, Munich 80804, Germany; 64 Cardiovascular Health Research Unit, Departments of Epidemiology and Medicine, University of Washington, 1730 Minor Ave, Suite 1360, Seattle, Washington 98101, USA; 65 Division of Psychiatric Genomics, Department of Psychiatry, Icahn School of Medicine at Mount Sinai, New York, New York 10029, USA; 66 Institute of Genetic Epidemiology, Helmholtz Zentrum München, German Research Center for Environmental Health, Ingolstaedter Landstraße 1, 85764 Neuherberg, Germany; 67 Research Unit of Molecular Epidemiology, Helmholtz Zentrum München, German Research Center for Environmental Health, Ingolstaedter Landstraße 1, 85764 Neuherberg, Germany; 68 Institute of Epidemiology II, Helmholtz Zentrum München, German Research Center for Environmental Health, 85764 Neuherberg, Germany; 69 German Center for Diabetes Research (DZD), Ingolstaedter Landstraße 1, Neuherberg 85764, Germany; 70 Westmead Millennium Institute, Centre for Vision Research, University of Sydney, C24 Westmead Hospital, New South Wales 2145, Australia; 71 Division of Biomedical Statistics and Informatics, Mayo Clinic, 200 First Street SW, Rochester, Minnesota 55905, USA; 72 Centre for Clinical Epidemiology and Biostatistics, School of Medicine and Public Health, University of Newcastle, HMRI Building1, Kookaburra Circuit, New Lambton New South Wales 2305, Australia; 73 Clinical Research Design, Information Technology and Statistical Support, Hunter Medical Research Institute, Newcastle, 1 Kookaburra Circuit, New Lambton Heights, New South Wales 2305, Australia; 74 Institute of Population Genetics—CNR, Traversa La Crucca 3, 07040 Reg. Baldinca, Li Punti, Sassari, Italy; 75 Department of Nutrition, Harvard School of Public Health, 665 Huntington Avenue, Building 2, Boston, Massachusetts 02115, USA; 76 Department of Internal Medicine, University Medical Center Groningen, University of Groningen, Hanzeplein 1, Groningen 9713 GZ, The Netherlands; 77 Brigham and Women’s Hospital and Channing Laboratory, Harvard Medical School, 181 Longwood Avenue, Boston, Massachusetts 02115, USA; 78 Cardiology, Department of Specialties of Internal Medicine, Geneva University Hospitals, Rue Gabrielle-Perret-Gentil 4, Geneva 1205, Switzerland; 79 Swiss Tropical and Public Health Institute, PO Box 4002, Basel, Switzerland; 80 University of Basel, Petersplatz 1, Basel 4003, Switzerland; 81 Division of Nephrology, Department of Internal Medicine and Medical Specialties, Columbus-Gemelli University Hospital, Catholic University, Via Moscati 31, Rome 00168, Italy; 82 Genetic Epidemiology, Queensland Institute of Medical Research, QIMR, PO Royal Brisbane Hospital, Queensland 4029, Australia; 83 Institute of Epidemiology and Biobank popgen, Christian-Albrechts University, Niemannsweg 11, Kiel 24105, Germany; 84 Institute of Epidemiology I, Helmholtz Zentrum München, German Research Center for Environmental Health, Ingolstädter Landstraße 1, Neuherberg 85764, Germany; 85 Institute of Medical Informatics, Biometry and Epidemiology, Ludwig-Maximilians-Universität, Ingolstädter Landstrasse 1, 85764 Neuherberg, Germany; 86 Klinikum Grosshadern, Ingolstädter Landstraße 1, Neuherberg 85764, Germany; 87 Centre for Population Health Sciences, University of Edinburgh, Teviot Place, Edinburgh, EH8 9AG Scotland, UK; 88 Austrian Stroke Prevention Study, Institute of Molecular Biology and Biochemistry, Department of Neurology, Medical University Graz, Harrachgasse 21, Graz 8010, Austria; 89 Institute of Clinical Chemistry and Laboratory Medicine, University Medicine Greifswald, Ferdinand-Sauerbruch-Straße, Greifswald 17475, Germany; 90 German Center for Cardiovascular Research (DZHK), Partner site Greifswald, Ferdinand-Sauerbruch-Straße, Greifswald 17475, Germany; 91 Institute of Pharmacology, University of Greifswald, Friedrich-Loeffler-Straße 23d, Greifswald 17487, Germany; 92 Boston University School of Medicine, 72 East Concord Street, B-616, Boston, Massachusetts 02118, USA; 93 Department of Cardiology, University Medical Center Groningen, University of Groningen, PO Box 30.001, Groningen 9700 RB, The Netherlands; 94 Robertson Centre for Biostatistics, University of Glasgow, R1122B Level 11, Robertson Centre, Boyd Orr Building, Glasgow G12 8QQ, UK; 95 Division of Primary Care Medicine, Department of Community Medicine, Primary Care and Emergency Medicine, Geneva University Hospitals, Faculty of Medicine, University of Geneva, Geneva 1211, Switzerland; 96 Community Prevention Unit, University Institute of Social and Preventive Medicine, Lausanne University Hospital, Route de la Corniche 10, Lausanne 1010, Switzerland; 97 Department of Epidemiology, Rollins School of Public Health, Emory University, 1518 Clifton Road, NE, Atlanta, Georgia 30322, USA; 98 Department of Genomics of Common Disease, School of Public Health, Imperial College London, London W12 0NN, UK; 99 Croatian Centre for Global Health, University of Split Medical School, Šoltanska 2, Split 21000, Croatia; 100 Department of Cardiology, Leiden University Medical Center, PO Box 9600, Leiden 2300 RC, The Netherlands; 101 Interuniversity Cardiology Institute of the Netherlands (ICIN), Moreelsepark 1, Utrecht 3511 EP, The Netherlands; 102 Einthoven Laboratory for Experimental Vascular Medicine, Albinusdreef 2, Leiden 2333 ZA, The Netherlands; 103 Durrer Center for Cardiogenetic Research, Meibergdreef 9, Amsterdam 1105 AZ, The Netherlands; 104 Division of Public Health Sciences, Department of Biostatistical Sciences, Wake Forest University Health Sciences, 2326 Medical Center Boulevard, Winston-Salem, North Carolina 27157-1063, USA; 105 INSERM U744, Institut Pasteur de Lille, 1 rue du Pr, Calmette, Lille Cédex 59019, France; 106 Department of Epidemiology, School of Public Health, University of Michigan, 1415 Washington Heights, Ann Arbor, Michigan 48109-2029, USA; 107 Survey Research Center, Institute for Social Research, University of Michigan, 426 Thompson Street, #3456, Ann Arbor, Michigan 48104, USA; 108 Centre for Vision Research, Westmead Millennium Institute, University of Sydney, C24 Westmead Hospital, New South Wales 2145, Australia; 109 Wake Forest School of Medicine, Department of Internal Medicine/Geriatrics, Medical center Boulevard, Winston-Salem, North Carolina 27157, USA; 110 Department of Genetics, Harvard Medical School, 77 Avenue Louis Pasteur, NRB 0330, Boston, Massachusetts 02115, USA; 111 University of Sydney, The George Institute for Global Health, Level 10, King George V Building, 83-117 Missenden Road, Camperdown, New South Wales 2050, Australia; 112 Department of Medicine, University of Turku, Turku University Hospital, PO Box 52, Turku 20521, Finland; 113 Welch Center for Prevention, Epidemiology and Clinical Research, 2024 East Monument St, Suite 2-600, Baltimore, Maryland 21287, USA; 114 Vanderbilt University School of Medicine, 448 Eskind Biomedical Library, 2209 Garland Avenue, Nashville, Tennessee 37212, USA; 115 Department of Genetics, University of Groningen, University Medical Centre Groningen, PO Box 72, Groningen 9700 AB, The Netherlands; 116 Division of Endocrinology, Medical University of South Carolina, 171 Ashley Avenue, Charleston, South Carolina 29425, USA; 117 Institute of Anatomy and Cell Biology, University of Greifswald, Friedrich-Loeffler-Straße 23c, Greifswald 17487, Germany; 118 Center for Health Disparities, Department of Biology, East Carolina University, 1001 East 10th Street, N209 Howell Science Complex Mailstop 551, Greenville, North Carolina 27858, USA; 119 Intramural Research Program, Laboratory of Epidemiology, Demography, and Biometry, National Institute on Aging, Gateway Building, 3C309, 7201 Winsconsin Avenue, Bethesda, Maryland 20892-9205, USA; 120 Department of Clinical Chemistry, Fimlab Laboratories, University of Tampere, School of Medicine, Tampere 33520, Finland; 121 CNRS UMR 8199, 1 Rue du Professeur Calmette, Lille 59000, France; 122 Lille Pasteur Institute, 1 Rue du Professeur Calmette, Lille 59000, France; 123 Lille II University, 42 Rue paul Duez, Lille 59000, France; 124 Clinical Research Branch, National Institute on Aging, 251 Bayview Blvd, Baltimore, Maryland 21250, USA; 125 Department of Epidemiology and Biostatistics, Erasmus University Medical Center, Dr Molewaterplein, Rotterdam 50-603015 GE, The Netherlands; 126 Department of Forensic Molecular Biology, Erasmus University Medical Center, Dr Molewaterplein, Rotterdam 50-603015 GE, The Netherlands; 127 Medical Clinic V, Medical Faculty Mannheim, Heidelberg University, Theodor-Kutzer-Ufer 1-3, Mannheim 68167, Germany; 128 Austrian Stroke Prevention Study, Department of Neurology, Division of Special Neurology, Medical University Graz, Auenbruggerplatz 22, Graz 8036, Austria; 129 Centre for Clinical Epidemiology and Biostatistics, University of Newcastle, Hunter Medical Research Institute, John Hunter Hospital, Locked Bag 1, HRMC, New South Wales 2310, Australia; 130 The George Institute for Global Health, Nuffield Department of Population Health, University of Oxford, Old Road Campus, Roosevelt Drive, Oxford OX3 7LF, UK; 131 Service of Nephrology, Lausanne University Hospital, Rue du Bugnon 17, Lausanne 1005, Switzerland; 132 Inserm UMRS 1018, CESP Team 10, Université Paris-Sud, 16 avenue Paul Vaillant Couturier, Villejuif 94807, France; 133 Health Disparities Research Section, Clinical Research Branch, National Institute on Aging, National Institutes of Health, NIH Biomedical Center, 251 Bayview Boulevard, Suite 100, Baltimore, Maryland 21224, USA; 134 Center for Public Health Genomics, Department of Medicine (Cardiovascular Medicine), University of Virginia, PO Box 800717, Charlottesville, Virginia 22908, USA; 135 Department of Clinical Physiology, Tampere University Hospital, University of Tampere, School of Medicine, Tampere 33521, Finland; 136 University Institute of Social and Preventive Medicine, Centre Hospitalier Universitaire Vaudois, University of Lausanne, Route de la Corniche 2, Epalinges CH-1066, Switzerland; 137 INSERM UMR970, Paris Cardiovascular Research Center (PARCC), 56 rue Leblanc, Paris F-75015, France; 138 Paris Descartes University, Faculty of medicine, Paris Cité Sorbonne, 12 Rue de l'école de Médecine, Paris F-75006, France; 139 Brigham and Women’s Hospital, Harvard Medical School, 77 Avenue Louis Pasteur, Boston, Massachusetts 02115, USA; 140 Wellcome Trust Sanger Institute, Hinxton CB10 1HH, UK; 141 University of Zurich, Institute of Physiology, Mechanisms of Inherited Kidney Disorders Group, Winterthurerstrasse 190, Zürich 8057, Switzerland; 142 Research Centre of Applied and Preventive Cardiovascular Medicine, University of Turku, Turku University Hospital, Department of Clinical Physiology, PO Box 52, Turku 20521, Finland; 143 Department Clinical Pharmacology, William Harvey Research Institute, Queen Mary University of London, London EC1M 6BQ, UK; 144 NIHR Barts Cardiovascular Biomedical Research Unit, Queen Mary University of London, London EC1M 6BQ, UK; 145 Harvard Medical School, 900 Commonwealth Avenue East, Boston, Massachusetts 02115, USA; 146 University of Alabama at Birmingham, Department of Medicine, 1530 3rd Avenue, South Birmingham, Alabama 35294-0022, USA; 147 University of Alabama at Birmingham, Department of Epidemiology, 1530 3rd Avenue, South Birmingham, Alabama 35294-0022, USA; 148 Department of Psychiatry and EMGO+ Institute, VU University Medical Center, A.J. Ernststraat 1187, Amsterdam 1081 HL, The Netherlands; 149 IFB AdiposityDiseases, University of Leipzig, Liebigstraße 21, Leipzig 04103, Germany; 150 Medical University Center Mainz, Langenbeckstraße 1, Mainz 55131, Germany; 151 Institute of Physiology, University of Greifswald, Greifswald 17487, Germany; 152 Clinic for Prosthodontic Dentistry, Gerostomatology and Material Science, University of Greifswald, Rotgerberstraße 8, Greifswald 17475, Germany; 153 School of Biomedical Sciences and Pharmacy, University of Newcastle, Hunter Medical Research Institute, John Hunter Hospital, Locked Bag 1, HRMC, New South Wales 2310, Australia; 154 Kidney Research Institute, University of Washington, Box 359606, 325 9th Avenue, Seattle, Washington 98104, USA; 155 Institute for Maternal and Child Health—IRCCS "Burlo Garofolo", Via dell'Istria 65, Trieste 34137, Italy; 156 Department of Internal Medicine, Division of Nephrology and Hypertension, Mayo Clinic, 200 First Street SW, Rochester, Minnesota 55905, USA; 157 Clinic for Internal Medicine A, University of Greifswald, Friedrich-Loeffler-Straße 23a, Greifswald 17475, Germany; 158 Faculty of Pharmaceutical Sciences, University of Iceland, Sæmundargata 2, Reykjavik 101, Iceland; 159 Department of Clinical Physiology, Turku University Hospital, Research Centre of Applied and Preventive Cardiovascular Medicine, University of Turku, PO Box 52, Turku 20521, Finland; 160 Synlab Academy, Synlab Services GmbH, Oberer Eselsberg 45, Ulm 89081, Germany; 161 Department of Internal Medicine II—Cardiology, University of Ulm Medical Centre, Albert-Einstein-Allee 23, Ulm 89081, Germany; 162 The Mindich Child Health and Development Institute, Ichan School of Medicine at Mount Sinai, New York, New York 10029, USA; 163 Department of Neurology, General Central Hospital, Via Lorenz Bohler 5, Bolzano 39100, Italy; 164 Department of Neurology, University of Lübeck, Ratzeburger Allee 160, Lübeck 23538, Germany; 165 University of Maryland Medical School, Division of Nephrology, 685 W. Baltimore Street, MSTF 314, Baltimore, Maryland 21201, USA; 166 CRCHUM, University of Montreal, CHUM Research Center, Technopôle Angus, 900 Saint-Denis, Montreal, Québec, Canada H2X 0A9; 167 Division of Endocrinology, Brigham and Women s Hospital, Harvard Medical School, 221 Longwood Avenue, Boston, Massachusetts 02115, USA; 168 Center for Statistical Genetics, Department of Biostatistics, University of Michigan, School of Public Health, Ann Arbor, Michigan 48103, USA; 169 Department of Nutrition, University of North Carolina, Chapel Hill, North Carolina 27599, USA; 170 Department of Public Health and Primary Care, University of Cambridge, Cambridge, CB1 8RN, UK; 171 Department of Medicine, Harvard Medical School, Boston, Massachusetts 02115, USA; 172 Department of Genetics, Harvard Medical School, Boston, Massachusetts 02115, USA; 173 Center for Complex Disease Genomics, McKusick-Nathans Institute of Genetic Medicine, Johns Hopkins University School of Medicine, Baltimore, Maryland 21205, USA; 174 Center for Human Genetic Research, Cardiovascular Research Center, Massachusetts General Hospital, Boston, Massachusetts 02114, USA; 175 Geriatric Rehabilitation Unit, Azienda Sanitaria Firenze (ASF), 50125 Florence, Italy; 176 Département de Génétique Médicale, Université de Lausanne, Lausanne 1015, Switzerland; 177 Pathology and Laboratory Medicine, University of Western Australia, 6009 Crawley, Western Australia, Australia; 178 Department of Physiology and Biophysics, Keck School of Medicine, University of Southern California, Los Angeles, California 90033, USA; 179 Cardiovascular Health Research Unit, Department of Medicine, University of Washington, Seattle 98195, Washington, USA; 180 National Human Genome Research Institute, National Institutes of Health, Bethesda, Maryland 20892, USA; 181 Department of Medicine III, Medical Faculty Carl Gustav Carus at the Technical University of Dresden, Dresden 01307, Germany; 182 Julius Center for Health Sciences and Primary Care, University Medical Center Utrecht, Heidelberglaan 100, Utrecht 3508 GA, The Netherlands; 183 Leibniz-Institute for Arteriosclerosis Research, Department of Molecular Genetics of Cardiovascular Disease, University of Münster, Münster, Germany; 184 University Hospital Münster, Internal Medicine D, Münster, Germany; 185 Department of Cardiovascular Sciences, University of Leicester, Glenfield Hospital, Leicester LE3 9QP, UK; 186 Clinical Pharmacology Unit, University of Cambridge, Addenbrookes Hospital, Hills Road, Cambridge CB2 2QQ, UK; 187 Department of Health Sciences, University of Leicester, University Rd, Leicester LE1 7RH, UK; 188 Faculty of Epidemiology and Population Health, London School of Hygiene and Tropical Medicine, London WC1E 7HT, UK; 189 Clinical Pharmacology and The Genome Centre, William Harvey Research Institute, Barts and The London School of Medicine and Dentistry, Queen Mary University of London, London EC1M 6BQ, UK; 190 Department of Epidemiology and Biostatistics, School of Public Health, Imperial College London, Norfolk Place, London W2 1PG, UK; 191 Centre for Cellular and Molecular Biology (CCMB), Council of Scientific and Industrial Research (CSIR), Uppal Road, Hyderabad 500 007, India; 192 University of Maryland, School of Medicine, Baltimore, Maryland 21201, USA; 193 School of Science and Engineering, University of Ballarat, Ballarat 3353, Australia; 194 International Centre for Circulatory Health, National Heart & Lung Institute, Imperial College, London, UK; 195 Center for Genome Science, National Institute of Health, Seoul, Korea; 196 Clinical Trial Service Unit and Epidemiological Studies Unit, University of Oxford, Oxford OX3 7LF, UK; 197 University of Dundee, Ninewells Hospital & Medical School, Dundee DD1 9SY, UK; 198 Centre for Cardiovascular Genetics, University College London, London WC1E 6JF, UK; 199 Centre for Genetic Epidemiology and Biostatistics, University of Western Australia, Crawley, Western Australia, Australia; 200 Department of Preventive Medicine and Epidemiology, Loyola University Medical School, Maywood, Illinois, USA; 201 Tuscany Regional Health Agency, Florence, Italy; 202 Department of Internal Medicine B, Ernst-Moritz-Arndt-University Greifswald, Greifswald 17487, Germany; 203 Occupational and Environmental Medicine, Department of Public Health and Community Medicine, Institute of Medicine, Sahlgrenska Academy, University of Gothenburg, Gothenburg 40530, Sweden; 204 MRC Centre for Causal Analyses in Translational Epidemiology, School of Social & Community Medicine, University of Bristol, Bristol BS8 2BN, UK; 205 BHF Glasgow Cardiovascular Research Centre, University of Glasgow, 126 University Place, Glasgow G12 8TA, UK; 206 Georgia Prevention Institute, Department of Pediatrics, Medical College of Georgia, 30912 Augusta, Georgia, USA; 207 Cardiovascular Epidemiology and Genetics, Institut Municipal d’Investigacio Medica, Barcelona Biomedical Research Park, 88 Doctor Aiguader, Barcelona 08003, Spain; 208 Medizinische Klinik II, Universität zu Lübeck, 23562 Lübeck, Germany; 209 Department of Statistics, Pontificia Universidad Catolica de Chile, Vicun˜a Mackena, Santiago 4860, Chile; 210 Department of Cardiovascular Medicine, The Wellcome Trust Centre for Human Genetics, University of Oxford, Oxford OX3 7BN, UK; 211 Department of Clinical Sciences, Lund University, SE-205 02 Malmö, Sweden; 212 Tropical Medicine Research Institute, University of the West Indies, Mona, Kingston, Jamaica; 213 Department of Medicine, University of Mississippi Medical Center, 2500 North State St, Jackson, Mississippi 39216, USA; 214 Genetics of Complex Traits, Peninsula Medical School, University of Exeter, EX1 2LU Exeter, UK; 215 U557 Institut National de la Santé et de la Recherche Médicale, U1125 Institut National de la Recherche Agronomique, Université Paris 13, F-93017 Bobigny, France; 216 Department of Internal Medicine, Division of Cardiovascular Medicine, University of Michigan Medical Center, Ann Arbor 48108, Michigan, USA; 217 Laboratory of Epidemiology, Demography, Biometry, National Institute on Aging, National Institutes of Health, Bethesda, Maryland 20892, USA; 218 Department of Clinical Sciences, Diabetes and Endocrinology Research Unit, University Hospital, SE-205 02 Malmö, Sweden; 219 Human Genetics Foundation (HUGEF), Via Lagrange 35, Torino 10123, Italy; 220 Medical Genetics Institute, Cedars-Sinai Medical Center, Los Angeles, 90048 California, USA; 221 Division of Community Health Sciences, St George’s University of London, London SW17 0RE, UK; 222 Atherosclerosis Research Unit, Department of Medicine, Karolinska Institute, SE-171 77 Stockholm, Sweden; 223 MRC Unit for Lifelong Health & Ageing, London WC1B 5JU, UK; 224 Institute of Clinical Medicine/Obstetrics and Gynecology, University of Oulu, 90014 Oulu, Finland; 225 Centre National de Génotypage, Commissariat de L’Energie Atomique, Institut de Génomique, 91000 Evry, France; 226 Department of Epidemiology, University of Washington, Seattle, Washington 98195, USA; 227 Epidemiology Public Health, UCL, London WC1E 6BT, UK; 228 William Harvey Research Institute, Barts and The London School of Medicine and Dentistry, Queen Mary University of London, London EC1M 6BQ, UK; 229 Cardiovascular Genetics, University of Utah, School of Medicine, Salt Lake City, 84132 Utah, USA; 230 HUNT Research Centre, Department of Public Health and General Practice, Norwegian University of Science and Technology, Levanger 7600, Norway; 231 Department of Community Health Sciences, Aga Khan University, 74800 Karachi, Pakistan; 232 Department of Medicine, Aga Khan University, 74800 Karachi, Pakistan; 233 Department of Genomic Medicine, National Cerebral and Cardiovascular Research Center, Suita 565-8565, Japan; 234 Department of Preventive Cardiology, National Cerebral and Cardiovascular Research Center, Suita 565-8565, Japan; 235 Medical Population Genetics, Broad Institute of Harvard and MIT, 5 Cambridge Center, Cambridge, Massachusetts 02142, USA; 236 Division of Non-communicable disease Epidemiology, The London School of Hygiene and Tropical Medicine London, Keppel Street, London WC1E 7HT, UK; 237 Department of Health Science, Shiga University of Medical Science, Otsu 520-2192, Japan; 238 National Heart and Lung Institute, Imperial College London, London W12 0HS, UK; 239 Diabetes Unit, KEM Hospital and Research Centre, Rasta Peth, Pune, 411011 Maharashtra, India; 240 Genetic Epidemiology Group, Epidemiology and Public Health, UCL, London WC1E 6BT, UK; 241 Department of Medicine, University of Kuopio, Kuopio University Hospital, Kuopio 70210, Finland; 242 Studies Coordinating Centre, Division of Hypertension and Cardiac Rehabilitation, Department of Cardiovascular Diseases, University of Leuven, Campus Sint Rafaël, Kapucijnenvoer 35, Block D, Box 7001, Leuven 3000, Belgium; 243 Institute of Molecular and Cell Biology, University of Tartu, Riia 23, Tartu 51010, Estonia; 244 Finnish Institute of Occupational Health, Finnish Institute of Occupational Health, Aapistie 1, Oulu 90220, Finland; 245 Gerontology Research Center, National Institute on Aging, Baltimore, Maryland 21224, USA; 246 Wake Forest University Health Sciences, Winston-Salem, North Carolina 27157, USA; 247 National Heart, Lung and Blood Institute Framingham Heart Study, Framingham, 01702-5827 Massachusetts, USA; 248 Office of Population Studies Foundation, University of San Carlos, Talamban, Cebu 6000, Philippines; 249 Department of Epidemiology and Biostatistics, Case Western Reserve University, 2103 Cornell Road, Cleveland, Ohio 44106, USA; 250 Department of Medicine and Neurology, University ofWashington, Seattle, Washington 98195, USA; 251 MRC Epidemiology Unit, Institute of Metabolic Science, Cambridge CB2 0QQ, UK; 252 Department of Twin Research and Genetic Epidemiology, King’s College London, SE1 7EH London, UK; 253 Department of Genetics, Biology and Biochemistry, University of Torino, Via Santena 19, Torino 10126, Italy; 254 ALSPAC Laboratory, University of Bristol, Bristol BS8 2BN, UK; 255 Institute of Human Genetics, Helmholtz Zentrum Munich, German Research Centre for Environmental Health, Neuherberg 85764, Germany; 256 U872 Institut National de la Santé et de la Recherche Médicale, Centre de Recherche des Cordeliers, 75006 Paris, France; 257 Mayo Clinic, Jacksonville, 32224 Florida, USA; 258 Department of Basic Medical Research and Education, Ehime University Graduate School of Medicine, Toon 791-0295, Japan; 259 Department of Geriatric Medicine, Ehime University Graduate School of Medicine, Toon 791-0295, Japan; 260 Department of Genetics, University of North Carolina, Chapel Hill, North Carolina 27599, USA; 261 Division of Genetics, GlaxoSmithKline, Philadelphia, Pennsylvania 19101, USA; 262 Department of Primary Care and Population Health, UCL, London NW3 2PF, UK; 263 Department of Medicine (Geriatrics), University of Mississippi Medical Center, Jackson, 39216 Mississippi, USA; 264 Laboratory of Cardiovascular Science, Intramural Research Program, National Institute on Aging, NIH, Baltimore, 21224-6825 Maryland, USA; 265 Department of Geriatric Medicine, Osaka University Graduate School of Medicine, Suita 565-0871, Japan; 266 Tohoku University, Graduate School of Pharmaceutical Sciences and Medicine, Sendai 980-8578, Japan; 267 Genome Institute of Singapore, Agency for Science, Technology and Research, Singapore 138672, Singapore; 268 Istituto di Neurogenetica e Neurofarmacologia, Consiglio Nazionale delle Ricerche, Cittadella Universitaria di Monserrato, 09042 Monserrato, Cagliari, Italy; 269 Columbia University, New York, 10027 New York, USA; 270 Amgen, 1 Kendall Square, Building 100, Cambridge, Massachusetts 02139, USA; 271 National Institute for Health and Welfare, Helsinki 00271, Finland; 272 Merck Research Laboratory, 126 East Lincoln Avenue, Rahway, New Jersey 07065, USA; 273 South Asia Network for Chronic Disease, Public Health Foundation of India, C-1/52, SDA, New Delhi 100016, India; 274 Division of Biostatistics, Washington University School of Medicine, Saint Louis, Missouri 63110, USA; 275 Center for Non-Communicable Diseases, 74800 Karachi, Pakistan; 276 Department of Biostatistics, University of Washington, Seattle, 98105 Washington, USA; 277 Department of Emergency and Cardiovascular Medicine, Institute of Medicine, Sahlgrenska Academy, University of Gothenburg, Gothenburg 41685, Sweden; 278 Epidemiology & Prevention, Division of Public Health Sciences, Wake Forest University, School of Medicine, Winston-Salem, North Carolina 27157, USA; 279 University of Ibadan, PMB 5017 Ibadan, Nigeria; 280 Prevention and Care of Diabetes, Department of Medicine III, Medical Faculty Carl Gustav Carus at the Technical University of Dresden, Dresden 01307, Germany; 281 Department of Laboratory Medicine, Institute of Human Genetics, University of California, San Francisco, 513 Parnassus Avenue, San Francisco, California 94143, USA; 282 Imperial College Cerebrovascular Unit (ICCRU), Imperial College, London W6 8RF, UK; 283 Centre for Molecular Epidemiology, Yong Loo Lin School of Medicine, National University of Singapore, Singapore 117597, Singapore; 284 Department of Medicine, Yong Loo Lin School of Medicine, National University of Singapore, Singapore 119074, Singapore; 285 Faculty of Medicine, University of Split, 21000 Split, Croatia; 286 Department of Biostatistics, Institute of Basic Medical Sciences, University of Oslo, Oslo 0317, Norway; 287 Diabetes Prevention Unit, National Institute for Health and Welfare, Helsinki 00271, Finland; 288 Lifestyle-related Disease Prevention Center, Shiga University of Medical Science, Otsu 520-2192, Japan; 289 Department of Medical Science and Cardiorenal Medicine, Yokohama City University School of Medicine, Yokohama 236-0004, Japan; 290 Tallinn University of Technology, Institute of Biomedical Engineering, Ehitajate tee 5, Tallinn 19086, Estonia; 291 Department of Epidemiology and Public Health, Imperial College, Norfolk Place, London W2 1PG, UK; 292 Department of Internal Medicine, Centre Hospitalier Universitaire Vaudois, Lausanne 1011, Switzerland; 293 Department of Medicine, Johns Hopkins University, Baltimore, 21205 Maryland, USA; 294 Lapland Central Hospital, Department of Physiatrics, Box 8041, Rovaniemi 96101, Finland; 295 Department of Internal Medicine, Diabetology, and Nephrology, Medical University of Silesia; 296 Laboratory for Statistical Analysis, RIKEN Center for Integrative Medical Sciences, Yokohama, Kanagawa 230-0045, Japan; 297 Department of Human Genetics and Disease Diversity, Graduate School of Medical and Dental Sciences, Tokyo Medical and Dental University, Tokyo 113-8510, Japan; 298 Institute of Biomedical Sciences, Academia Sinica, Taipei 115, Taiwan; 299 School of Chinese Medicine, China Medical University, Taichung 404, Taiwan; 300 Cardiovascular Institute and Fu Wai Hospital, Chinese Academy of Medical Sciences, Peking Union Medical College, Beijing 100037, China; 301 Department of Gene Diagnostics and Therapeutics, Research Institute, National Center for Global Health and Medicine, Tokyo 162-8655, Japan; 302 Laboratory for Endocrinology and Metabolism, RIKEN Center for Integrative Medical Sciences, Yokohama, Kanagawa 230-0045, Japan; 303 Laboratory for Medical Informatics, RIKEN Center for Integrative Medical Sciences, Yokohama, Kanagawa 230-0045, Japan; 304 Saw Swee Hock School of Public Health, National University of Singapore, Singapore 119077, Singapore; 305 Department of Medicine, Khoo Teck Puat Hospital, Singapore 768828, Singapore; 306 Department of Epidemiology and Public Health, Yong Loo Lin School of Medicine, National University of Singapore, Singapore 117597, Singapore; 307 Singapore Eye Research Institute, Singapore National Eye Centre, Singapore 168751, Singapore; 308 Department of Ophthalmology, Yong Loo Lin School of Medicine, National University of Singapore, Singapore 117597, Singapore; 309 Centre for Eye Research Australia, University of Melbourne, East Melbourne, 3002 Victoria, Australia; 310 Center for Human Genetics, Duke University Medical Center, Durham, 27710 North Carolina, USA; 311 Department of Statistics and Applied Probability, National University of Singapore, Singapore 117546, Singapore; 312 NUS Graduate School for Integrative Science and Engineering, National University of Singapore, Singapore 119077, Singapore; 313 Center for Genome Science, National Institute of Health, Osong Health Technology Administration Complex, 187 Chungcheongbuk-do, Korea; 314 Department of Preventive Medicine, Seoul National University College of Medicine, Seoul 08826, Korea; 315 Department of Epidemiology, Tulane University School of Public Health and Tropical Medicine, New Orleans, 70112 Louisiana, USA; 316 Department of Clinical Gene Therapy, Osaka University Graduate School of Medicine, Suita, Osaka 565-0871, Japan; 317 Department of Geriatric Medicine and Nephrology, Osaka University Graduate School of Medicine, Suita, Osaka 565-0871, Japan; 318 Department of Allergy and Rheumatology, Graduate School of Medicine, University of Tokyo, Tokyo 113-0033, Japan; 319 Laboratory for Genotyping Development, RIKEN Center for Integrative Medical Sciences, Yokohama, Kanagawa 230-0045, Japan; 320 Laboratory of Molecular Medicine, Human Genome Center, Institute of Medical Science, University of Tokyo, Tokyo 108-8639, Japan; 321 Laboratory for International Alliance, RIKEN Center for Integrative Medical Sciences, Yokohama, Kanagawa 230-0045, Japan; 322 Laboratory for Cardiovascular Diseases, RIKEN Center for Integrative Medical Sciences, Yokohama, Kanagawa 230-0045, Japan; 323 The Cardiovascular Institute, University of Pennsylvania, Philadelphia, Pennsylvania 19104, USA; 324 Institut für integrative und experimentelle Genomik, Universität zu Lübeck, Lübeck 23562, Germany; 325 Deutsches Herzzentrum München, Technische Universität München, München 80636, Germany; 326 Department of Medicine, Stanford University School of Medicine, Stanford, 94305-5101 California, USA; 327 Division of Cardiovascular and Neuronal Remodelling, Multidisciplinary Cardiovascular Research Centre, Leeds Institute of Genetics, Health and Therapeutics, University of Leeds, Leeds LS2 9JT, UK; 328 Klinik und Poliklinik für Innere Medizin II, Universität Regensburg, 93053 Regensburg, Germany; 329 Institut für Medizinische Biometrie und Statistik, Universität zu Lübeck, 23562 Lübeck, Germany; 330 Science Center, Tampere University Hospital, Tampere 33521, Finland; 331 The John & Jennifer Ruddy Canadian Cardiovascular Genetics Centre, University of Ottawa Heart Institute, Ottawa, Ontario K1Y 4W7, Canada; 332 deCODE Genetics, Reykjavik 101, Iceland; 333 Faculty of Medicine, University of Iceland, Reykjavik 101, Iceland; 334 Hudson Alpha Institute, Huntsville, 35806 Alabama, USA; 335 Cardiovascular Research Methods Centre, University of Ottawa Heart Institute, 40 Ruskin Street, Ottawa, Ontario K1Y 4W7, Canada; 336 The Blavatnik School of Computer Science, Department of Molecular Microbiology and Biotechnology, Tel-Aviv University, Tel-Aviv 6997801, Israel; 337 The International Computer Science Institute, Berkeley, 94704 California, USA; 338 Biostatistics and Epidemiology, University of Pennsylvania, Philadelphia, 19104 Pennsylvania, USA; 339 Cardiovascular Research Center and Cardiology Division, Massachusetts General Hospital, Boston, 02114 Massachusetts, USA; 340 Center for Human Genetic Research, Massachusetts General Hospital, Boston, 02114 Massachusetts, USA; 341 Division of Research, Kaiser Permanente, Oakland, 94611 California, USA; 342 Institute for Human Genetics, University of California, San Francisco, San Francisco, 94143 California, USA; 343 Department of Cardiovascular Medicine, Cleveland Clinic 7255 Old Oak Blvd, Cleveland, Ohio 44130, USA; 344 Medizinische Klinik und Poliklinik, Johannes-Gutenberg Universität Mainz, Universitätsmedizin, 55122 Mainz, Germany; 345 Institut für Klinische Chemie und Laboratoriumsmediizin, Johannes-Gutenberg Universität Mainz, Universitätsmedizin, 55122 Mainz, Germany; 346 INSERM UMRS 937, Pierre and Marie Curie University (UPMC, Paris 6) and Medical School, 75005 Paris, France; 347 Boston University, School of Public Health, Boston, 02118 Massachusetts, USA; 348 University of Minnesota School of Public Health, Division of Epidemiology and Community Health, School of Public Health (A.R.F.), Minneapolis, 55454 Minnesota, USA; 349 University of Washington, Department of Internal Medicine, Seattle, 98195-6420 Washington, USA; 350 University of Texas, School of Public Health, Houston, 77030 Texas, USA; 351 Department of Medicine, Landspitali University Hospital, Reykjavik 101, Iceland; 352 Institute of Epidemiology, Helmholtz Zentrum München, German Research Center for Environmental Health, 85764 Neuherberg, Germany; 353 Division of Endocrinology and Diabetes, Graduate School of Molecular Endocrinology and Diabetes, University of Ulm, 89069 Ulm, Germany; 354 Division of Endocrinology, Department of Medicine, Medical University of Graz, 8010 Graz, Austria; 355 Synlab Center of Laboratory Diagnostics Heidelberg, 69037 Heidelberg, Germany; 356 Division of Clinical Chemistry, Department of Medicine, Albert Ludwigs University, 79085 Freiburg, Germany; 357 Clinical Institute of Medical and Chemical Laboratory Diagnostics, Medical University Graz, 8010 Graz, Austria; 358 Cardiology Group Frankfurt-Sachsenhausen, 60594 Frankfurt, Germany; 359 The Center for Applied Genomics, Children’s Hospital of Philadelphia, 19104 Philadelphia, Pennsylvania, USA; 360 Cardiovascular Research Institute, Medstar Health Research Institute, Washington Hospital Center, Washington, DC 20010, USA; 361 Genetics Division and Drug Discovery, GlaxoSmithKline, King of Prussia, Pennsylvania 19406, USA; 362 The Institute for Translational Medicine and Therapeutics, School of Medicine, University of Pennsylvania, Philadelphia, 19104-5158 Pennsylvania, USA; 363 Department of Cardiovascular Surgery, University of Leicester, Leicester LE1 7RH, UK; 364 Division of Cardiovascular and Diabetes Research, Multidisciplinary Cardiovascular Research Centre, Leeds Institute of Genetics, Health and Therapeutics, University of Leeds, Leeds LS2 9JT, UK; 365 LIGHT Research Institute, Faculty of Medicine and Health, University of Leeds, Leeds LS2 9JT, UK; 366 Department of Medicine, University of North Carolina at Chapel Hill, North Carolina 27516, USA; 367 Department of Epidemiology, University of North Carolina at Chapel Hill, North Carolina 27599-7435, USA; 368 National Institute of Environmental Health Sciences, Research Triangle Park, North Carolina 27709, USA; 369 Department of Biostatistics, University of North Carolina at Chapel Hill, North Carolina 27514, USA; 370 University of Texas, Houston Health Science Center, Houston, Texas 77030, USA; 371 Texas Heart Institute, Houston, Texas 77225-0345, USA; 372 Department of Internal Medicine, Division of Cardiology, Medical University Graz, Graz 8036, Austria; 373 Department of Medical Sciences, Uppsala University, Uppsala 75185, Sweden; 374 Division of Cardiovascular Diseases, Mayo Clinic, Rochester, Minnesota 55905, USA; 375 Institute of Medical Epidemiology, Biostatistics and Informatics, Martin Luther University of Halle-Wittenberg, Halle-Wittenberg, Halle (Saale) 06097, Germany; 376 Department of Cardiology, Erasmus University Medical Center, Rotterdam 3000 CA, The Netherlands; 377 Medical Clinic 2, University of Lübeck, Lübeck 23538, Germany; 378 Department of Medical Epidemiology and Biostatistics, Karolinska Institute, Stockholm 17177, Sweden; 379 Martin Luther University, Halle-Wittenberg, Halle (Saale) 06097, Germany; 380 Department of Epidemiology, University of Alabama at Birmingham, Birmingham, Alabama 35294-0022, USA; 381 Division of Cardiology, University of Maryland Hospital, Baltimore, Maryland 21201, USA; 382 Institute of Medical Biometry, Epidemiology, and Informatics, Johannes Gutenberg University, Mainz 55101, Germany; 383 Institute for Community Medicine, Ernst-Moritz-Arndt-Universität, Greifswald 17475, Germany

## Abstract

Reduced glomerular filtration rate defines chronic kidney disease and is associated with cardiovascular and all-cause mortality. We conducted a meta-analysis of genome-wide association studies for estimated glomerular filtration rate (eGFR), combining data across 133,413 individuals with replication in up to 42,166 individuals. We identify 24 new and confirm 29 previously identified loci. Of these 53 loci, 19 associate with eGFR among individuals with diabetes. Using bioinformatics, we show that identified genes at eGFR loci are enriched for expression in kidney tissues and in pathways relevant for kidney development and transmembrane transporter activity, kidney structure, and regulation of glucose metabolism. Chromatin state mapping and DNase I hypersensitivity analyses across adult tissues demonstrate preferential mapping of associated variants to regulatory regions in kidney but not extra-renal tissues. These findings suggest that genetic determinants of eGFR are mediated largely through direct effects within the kidney and highlight important cell types and biological pathways.

## Abstract (web summary)

Reduced glomerular filtration rate (eGFR) is a hallmark of chronic kidney disease. Here, Pattaro *et al*. conduct a meta-analysis to discover several new loci associated with variation in eGFR and find that genes associated with eGFR loci often encode proteins potentially related to kidney development.

## Graphical abstract

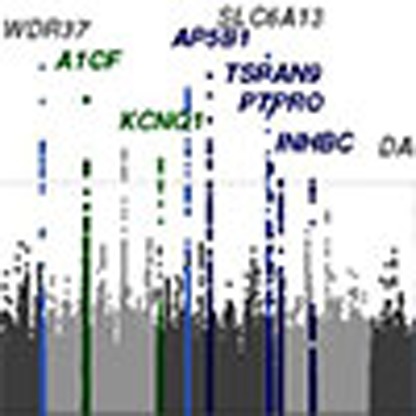

Chronic kidney disease (CKD) is a global public health problem^1^,^2^,^3^, and is associated with an increased risk for cardiovascular disease, all-cause mortality and end-stage renal disease^4^,^5^. Few new therapies have been developed to prevent or treat CKD over the past two decades^1^,^6^, underscoring the need to identify and understand the underlying mechanisms of CKD.

Prior genome-wide association studies (GWAS) have identified multiple genetic loci associated with CKD and estimated glomerular filtration rate (eGFR), a measure of the kidney’s filtration ability that is used to diagnose and stage CKD^7^,^8^,^9^,^10^,^11^,^12^,^13^,^14^,^15^. Subsequent functional investigations point towards clinically relevant novel mechanisms in CKD that were derived from initial GWAS findings^16^, providing proof of principle that locus discovery through large-scale GWAS efforts can translate to new insights into CKD pathogenesis.

To identify additional genetic variants associated with eGFR and guide future experimental studies of CKD-related mechanisms, we have now performed GWAS meta-analyses in up to 133,413 individuals, more than double the sample size of previous studies. Here we describe multiple novel genomic loci associated with kidney function traits and provide extensive locus characterization and bioinformatics analyses, further delineating the physiologic basis of kidney function.

## Results

### Stage 1 discovery analysis

We analysed associations of eGFR based on serum creatinine (eGFRcrea), cystatin C (eGFRcys, an additional, complementary biomarker of renal function) and CKD (defined as eGFRcrea <60 ml min^−1^ per 1.73 m^2^) with ∼2.5 million autosomal single-nucleotide polymorphisms (SNPs) in up to 133,413 individuals of European ancestry from 49 predominantly population-based studies (Supplementary Table 1). Results from discovery GWAS meta-analysis are publicly available at http://fox.nhlbi.nih.gov/CKDGen/. We performed analyses in each study sample in the overall population and stratified by diabetes status, since genetic susceptibility to CKD may differ in the presence of this strong clinical CKD risk factor. Population stratification did not impact our results as evidenced by low genomic inflation factors in our meta-analyses, which ranged from 1.00 to 1.04 across all our analyses (Supplementary Fig. 1).

**Figure 1:**
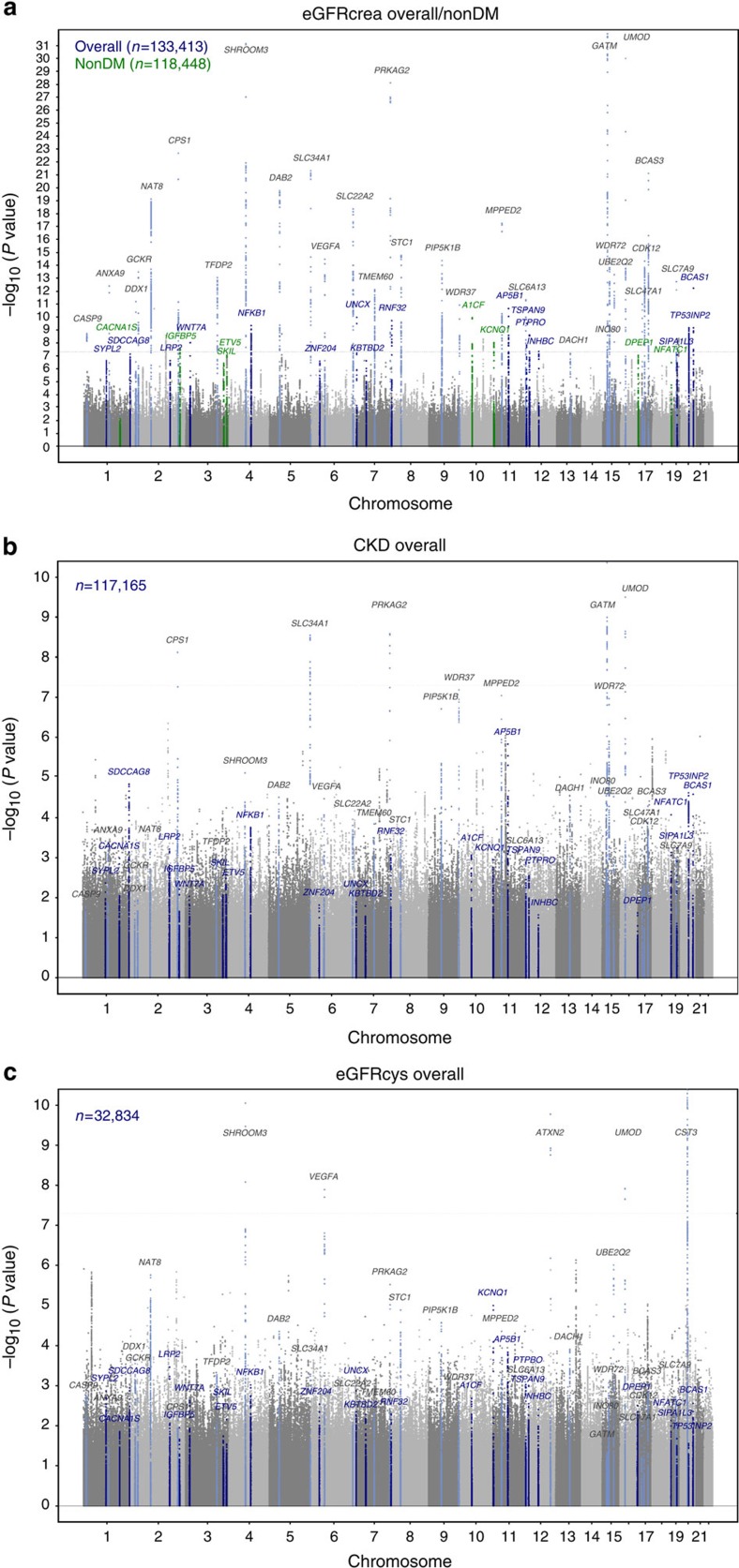
Discovery stage genome-wide association analysis. Manhattan plots for eGFRcrea, CKD and eGFRcys. Previously reported loci are highlighted in light blue (grey labels). (**a**) Novel loci uncovered for eGFRcrea in the overall and in the non-diabetes groups are highlighted in blue and green, respectively. (**b**) Results from CKD analysis with highlighted known and novel loci for eGFRcrea. (**c**) Results from eGFRcys with highlighted known and novel loci for eGFRcrea and known eGFRcys loci.

In addition to confirming 29 previously identified loci^7^,^8^,^9^ (Fig. 1a; Supplementary Table 2), we identified 48 independent novel loci (Supplementary Table 3) where the index SNP, defined as the variant with the lowest *P* value in the region, had an association *P* value <1.0 × 10^−6^. Of these 48 novel SNPs, 21 were genome-wide significant with *P* values <5.0 × 10^−8^. Overall, 43 SNPs were identified in association with eGFRcrea (nine in the non-diabetes sample), one with eGFRcys and four with CKD, as reported in Supplementary Table 3. Manhattan plots for CKD, eGFRcys and eGFRcrea in diabetes are shown in Fig. 1b,c and Supplementary Fig. 2, respectively.

### Stage-2 replication

Novel loci were tested for replication in up to 42,166 additional European ancestry individuals from 15 studies (Supplementary Table 1). Of the 48 novel candidate SNPs submitted to replication, 24 SNPs demonstrated a genome-wide significant combined stage 1 and 2 *P* value <5.0 × 10^−8^ (Table 1). Of these, 23 fulfilled additional replication criteria (*q*-value <0.05 in stage 2). Only rs6795744 at the *WNT7A* locus demonstrated suggestive replication (*P* value <5.0 × 10^−8^, *q*-value >0.05). Because serum creatinine is used to estimate eGFRcrea, associated genetic loci may be relevant to creatinine production or metabolism rather than kidney function *per se*. For this reason, we contrasted associations of eGFRcrea versus eGFRcys, the latter estimated from an alternative and creatinine-independent biomarker of GFR (Supplementary Fig. 3; Supplementary Table 4). The majority of loci (22/24) demonstrated consistent effect directions of their association with both eGFRcrea and eGFRcys.

Association plots of the 24 newly identified genomic regions that contain a replicated or suggestive index SNP appear in Supplementary Fig. 4. The odds ratio for CKD for each of the novel loci ranged from 0.93 to 1.06 (Supplementary Table 4). As evidenced by the relatively small effect sizes, the proportion of phenotypic variance of eGFRcrea explained by all new and known loci was 3.22%: 0.81% for the newly uncovered loci and 2.41% for the already known loci.

### Associations stratified by diabetes and hypertension status

**Figure 2:**
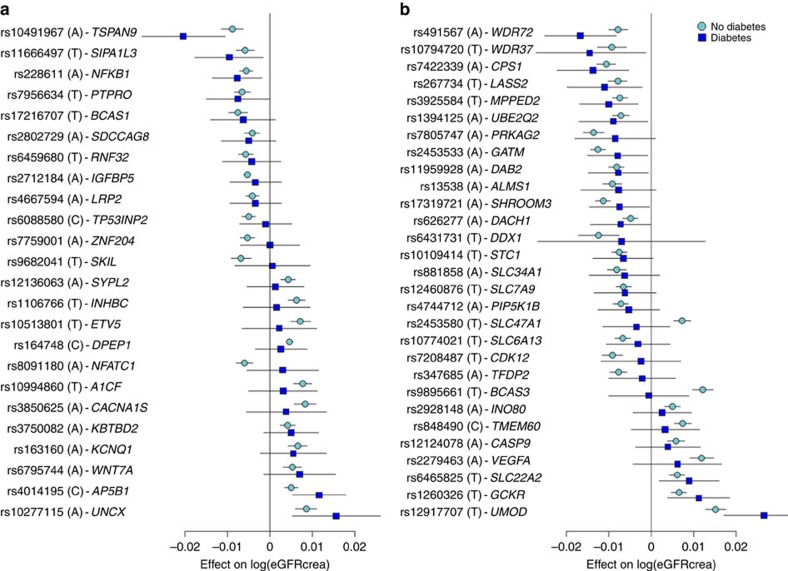
Association eGFRcrea loci in subjects with and without diabetes. Novel (**a**) and known (**b**) loci were considered. Displayed are effects and their 95% confidence intervals on ln(eGFRcrea). Results are sorted by increasing effects in the diabetes group. The majority of loci demonstrated similar effect sizes in the diabetes as compared with non-diabetes strata. SNP-specific information and detailed sample sizes are reported in Supplementary Table 5.

The effects of the 53 known and novel loci in individuals with (stage 1+stage 2 *n*=16,477) and without (stage 1+stage 2 *n*=154,881) diabetes were highly correlated (correlation coefficient: 0.80; 95% confidence interval: 0.67, 0.88; Supplementary Fig. 5) and of similar magnitude (Fig. 2; Supplementary Table 5), suggesting that identification of genetic loci in the overall population may also provide insights into loci with potential importance among individuals with diabetes. The previously identified *UMOD* locus showed genome-wide significant association with eGFRcrea among those with diabetes (Supplementary Fig. 2; rs12917707, *P* value=2.5 × 10^−8^), and six loci (*NFKB1*, *UNCX, TSPAN9, AP5B1, SIPA1L3* and *PTPRO*) had nominally significant associations with eGFRcrea among those with diabetes. Of the previously identified loci, 13 demonstrated nominal associations among those with diabetes, for a total of 19 loci associated with eGFRcrea in diabetes.

Exploratory comparison of the association effect sizes in subjects with and without hypertension based on our previous work^7^ showed that novel and known loci are also similarly associated with eGFRcrea among individuals with and without hypertension (Supplementary Fig. 6).

### Tests for SNP associations with related phenotypes

We tested for overlap with traits that are known to be associated with kidney function in the epidemiologic literature by investigating SNP associations with systolic and diastolic blood pressure^17^, myocardial infarction^18^, left ventricular mass^19^, heart failure^20^, fasting glucose^21^ and urinary albumin excretion (CKDGen Consortium, personal communication). We observed little association of the 24 novel SNPs with other kidney function-related traits, with only 2 out of 165 tests reaching the Bonferroni significance level of 0.0003 (see Methods and Supplementary Table 6).

To investigate whether additional traits are associated with the 24 new eGFR loci, we queried the NHGRI GWAS catalog (www.genome.gov). Overall, nine loci were previously identified in association with other traits at a *P* value of 5.0 × 10^−8^ or lower (Supplementary Table 7), including body mass index (*ETV5*) and serum urate (*INHBC*, *A1CF* and *AP5B1*).

### Trans-ethnic analyses

To assess the generalizability of our findings across ethnicities, we evaluated the association of the 24 newly identified loci with eGFRcrea in 16,840 participants of 12 African ancestry population studies (Supplementary Table 8) and in up to 42,296 Asians from the AGEN consortium^11^ (Supplementary Table 9). Seven SNPs achieved nominal direction-consistent significance (*P*<0.05) in AGEN, and one SNP was significant in the African ancestry meta-analysis (Supplementary Table 9). Random-effect meta-analysis showed that 12 loci (*SDCCAG8*, *LRP2*, *IGFBP5*, *SKIL*, *UNCX*, *KBTBD2*, *A1CF*, *KCNQ1*, *AP5B1*, *PTPRO*, *TP53INP2* and *BCAS1*) had fully consistent effect direction across the three ethnic groups (Supplementary Fig. 7), suggesting that our findings can likely be generalized beyond the European ancestry group.

To identify additional potentially associated variants and more formally evaluate trans-ethnic heterogeneity of the loci identified through meta-analysis in European ancestry populations, we performed a trans-ethnic meta-analysis^22^, combining the 12 African ancestry studies with the 48 European Ancestry studies used in the discovery analysis of eGFRcrea. Of the 24 new loci uncovered for eGFRcrea, 15 were also genome-wide significant in the trans-ethnic meta-analysis (defined as log_10_ Bayes Factor >6, Supplementary Table 10), indicating that for most of these loci, there is little to no allelic effect heterogeneity across the two ethnic groups. No additional loci were significantly associated with log_10_ Bayes Factor >6.

### Bioinformatic and functional characterization of new loci

We used several techniques to prioritize and characterize genes underlying the identified associations, uncover connections between associated regions, detect relevant tissues and assign functional annotations to associated variants. These included expression quantitative trait loci (eQTL) analyses, pathway analyses, DNAse I hypersensitivity site (DHS) mapping, chromatin mapping, manual curation of genes in each region and zebrafish knockdown.

### eQTL analysis

We performed eQTL analysis using publically available eQTL databases (see Methods). These analyses connected novel SNPs to transcript abundance of *SYPL2*, *SDCCAG8*, *MANBA*, *KBTBD2*, *PTPRO* and *SPATA33* (*C16orf55*), thereby supporting these as potential candidate genes in the respective associated regions (Supplementary Table 11).

### Pathway analyses

**Figure 3:**
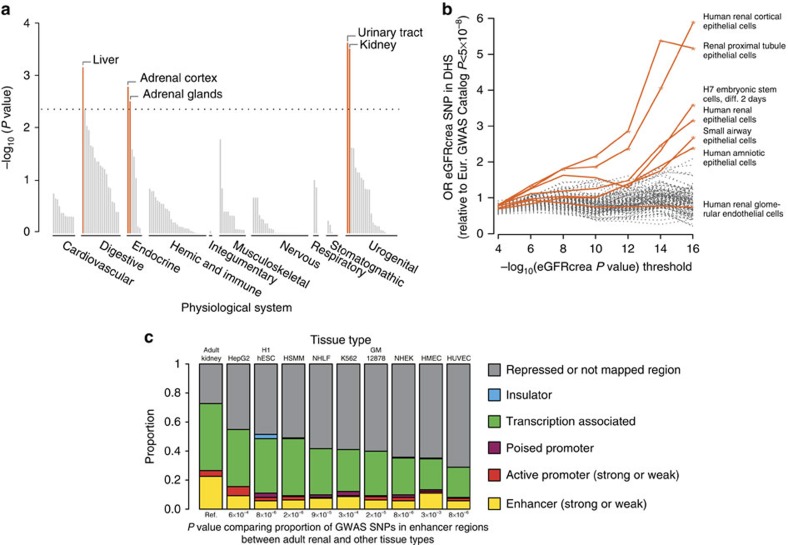
Bioinformatic analysis of eGFR-associated SNPs. Connection of eGFR-associated SNPs to gene expression and variant function across a variety of tissues, pathways and regulatory marks was considered. (**a**) The DEPICT method shows that implicated eGFR-associated genes are highly expressed in particular tissues, including kidney and urinary tract. Shown are permutation test *P* values (see Methods). (**b**) Enrichment of eGFRcrea-associated SNPs in DHS according to discovery *P* value threshold. SNPs from the eGFR discovery genome-wide scan meeting a series of *P* value thresholds in the range 10^−4^–10^−16^ preferentially map to DHSs, when compared with a set of control SNPs, in 6 of 123 cell types. Represented are main effects odds ratios from a logistic mixed effect model. Cell types indicated with coloured lines had nominally significant enrichment (* indicate *P* values <0.05) at the *P* value <10^−16^ threshold and/or were derived from renal tissues (H7esDiffa2d: H7 embryonic stem cells, differentiated 2 days with BMP4, activin A and bFGF; Hae, amniotic epithelial cells; Hrce, renal cortical epithelial cells; Hre, renal epithelial cells; Hrgec, renal glomerular endothelial cells; Rptec, renal proximal tubule epithelial cells; Saec, small airway epithelial cells). (**c**) ENCODE/Chromatin ChIP-seq mapping: known and replicated novel eGFRcrea-associated SNPs and their perfect proxies were annotated based on genomic location using chromatin annotation maps from different tissues including adult kidney epithelial cells. *P* values from Fishers’ exact tests for 2 × 2 tables are reported (significance level=5.6 × 10^−3^, see Methods). There is significant enrichment of variants mapping to enhancer regions specifically in kidney but not other non-renal tissues.

**Figure 4:**
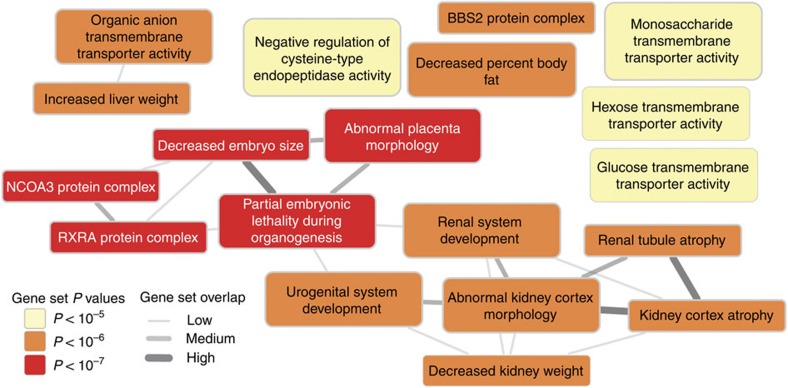
Gene set overlap analysis. The 19 reconstituted gene sets with *P* value<10^−5^ were considered. Their overlap was estimated by computing the pairwise Pearson correlation coefficient *ρ* between each pair of gene sets followed by discretization into one of three bins: 0.3≤*ρ*<0.5, low overlap; 0.5≤*ρ*<0.7, medium overlap; *ρ*⩾0.7, high overlap. Overlap is shown by edges between gene set nodes and edges representing overlap corresponding to *ρ*<0.3 are not shown. The network was drawn with Cytoscape^48^.

We used a novel method, Data-driven Expression Prioritized Integration for Complex Traits (DEPICT)^23^, to prioritize genes at associated loci, to test whether genes at associated loci are highly expressed in specific tissues or cell types and to test whether specific biological pathways and gene sets are enriched for genes in associated loci. On the basis of all SNPs with eGFRcrea association *P* values <10^−5^ in the discovery meta-analysis, representing 124 independent regions, we identified at least one significantly prioritized gene in 49 regions, including in 9 of the 24 novel genome-wide significant regions (Supplementary Table 12). Five tissue and cell type annotations were enriched for expression of genes from the associated regions, including the kidney and urinary tract, as well as hepatocytes and adrenal glands and cortex (Fig. 3a; Supplementary Table 13). Nineteen reconstituted gene sets showed enrichment of genes mapping into the associated regions at a permutation *P* value <10^−5^ (Supplementary Table 14; Fig. 4), highlighting processes related to renal development, kidney transmembrane transporter activity, kidney and urogenital system morphology, regulation of glucose metabolism, as well as specific protein complexes important in renal development.

### DNase I hypersensitivity and H3K4m3 chromatin mark analyses

To evaluate whether eGFRcrea-associated SNPs map into gene regulatory regions and to thereby gain insight into their potential function, we evaluated the overlap of independent eGFRcrea-associated SNPs with *P* values <10^−4^ (or their proxies) with DHSs using publicly available data from the Epigenomics Roadmap Project and ENCODE for 123 cell types (see Methods). DHSs mark accessible chromatin regions where transcription may occur. Compared with a set of control SNPs (see Methods), eGFRcrea-associated SNPs were significantly more likely to map to DHS in six specific tissues or cell types (Fig. 3b), including adult human renal cortical epithelial cells, adult renal proximal tubule epithelial cells, H7 embryonic stem cells (differentiated 2 days), adult human renal epithelial cells, adult small airway epithelial cells and amniotic epithelial cells. No significant enrichment was observed for adult renal glomerular endothelial cells, the only other kidney tissue evaluated.

Next, we analysed the overlap of the same set of SNPs with H3K4me3 chromatin marks, promoter-specific histone modifications associated with active transcription^24^, in order to gather more information about cell-type specific regulatory potential of eGFRcrea-associated SNPs. Comparing 33 available adult-derived cell types, we found that eGFRcrea-associated SNPs showed the most significant overlap with H3K4me3 peaks in adult kidney (*P* value=0.0029), followed by liver (*P* value=0.0117), and rectal mucosa (*P* value=0.0445). Taken together, these findings are suggestive of cell-type-specific regulatory roles for eGFR loci, with greatest specificity for the kidney.

### Chromatin annotation maps

In addition to assessing individual regulatory marks separately, we annotated the known and replicated novel SNPs, as well as their perfect proxies in a complementary approach. Chromatin annotation maps were generated integrating >10 epigenetic marks from cells derived from adult human kidney tissue and a variety of non-renal tissues from the ENCODE project (see Methods). The proportion of variants to which a function could be assigned was significantly higher when using chromatin annotation maps from renal tissue compared with using maps that investigated the same epigenetic marks in other non-renal tissues (Fig. 3c), again indicating that eGFRcrea associated SNPs are, or tag, kidney-specific regulatory variants. The difference between kidney and non-renal tissues was particularly evident for marks that define enhancers: the proportion of SNPs mapping to weak and strong enhancer regions in the kidney tissue was higher than in all non-kidney tissues (Fishers’ exact test *P* values from 3.1 × 10^−3^ to 7.9 × 10^−6^, multiple testing threshold *α*=5.6 × 10^−3^).

### Functional characterization of new loci

To prioritize genes for functional studies, we applied gene prioritization algorithms including GRAIL^25^, DEPICT and manual curation of selected genes in each region (Supplementary Table 12). For each region, gene selection criteria were as follows: (1) either GRAIL *P* value <0.05 or DEPICT false discovery rate (FDR) <0.05; (2) the effect of a given allele on eGFRcrea and on eGFRcys was direction-consistent and their ratio was between 0.2 and 5 (to ensure relative homogeneity of the beta coefficients); (3) nearest gene if the signal was located in a region containing a single gene. Using this approach, *NFKB1*, *DPEP1*, *TSPAN9*, *NFATC1*, *WNT7A*, *PTPRO*, *SYPL2*, *UNCX*, *KBTBD2*, *SKIL* and *A1CF* were prioritized as likely genes underlying effects at the new loci (Supplementary Table 12).

We investigated the role of these genes during vertebrate kidney development by examining the functional consequences of gene knockdown in zebrafish embryos utilizing antisense morpholino oligonucleotide (MO) technology. After knockdown, we assessed the expression of established renal markers *pax2a* (global kidney), *nephrin* (podocytes) and *slc20a1a* (proximal tubule) at 48 hours post fertilization by *in situ* hybridization^12^. In all cases, morphant embryos did not display significant gene expression defects compared with controls (Supplementary Table 15).

## Discussion

We identified 24 new loci in association with eGFR and confirmed 29 previously identified loci. A variety of complementary analytic, bioinformatic and functional approaches indicate enrichment of implicated gene products in kidney and urinary tract tissues. A greater proportion of the lead SNPs or their perfect proxies map into gene regulatory regions, specifically enhancers, in adult renal tissues compared with non-renal tissues. In addition to the importance in the adult kidney, our results indicate a role for kidney function variants during development.

We extend our previous findings, as well as those from other groups^7^,^8^,^9^,^10^,^11^,^12^,^13^ by identifying >50 genomic loci for kidney function, many of which were not previously known to be connected to kidney function and disease. Using a discovery data set that is nearly double in size to our prior effort^7^, we are now able to robustly link associated SNPs to kidney-specific gene regulatory function. Our work further exemplifies the continued value of increasing the sample size of GWAS meta-analyses to uncover additional loci and gain novel insights into the mechanisms underlying common phenotypes^26^.

There are several messages from our work. First, many of the genetic variants associated with eGFR appear to affect processes specifically within the kidney. The kidney is a highly vascular and metabolically active organ that receives 20% of all cardiac output, contains an extensive endothelium-lined capillary network, and is sensitive to ischaemic and toxic injury. As a result, hypertension, cardiovascular diseases and diabetes each affect renal hemodynamics and contribute to kidney injury. However, many of the eGFR-associated SNPs in our GWAS could be assigned gene regulatory function specifically in the kidney and its epithelial cells, but not in human glomerular endothelial cells or the general vasculature. In addition, variants associated with eGFR were not associated with vascular traits, such as blood pressure or myocardial infarction. Taken together, these findings suggest that genetic determinants of eGFR may be mediated largely through direct effects within the kidney.

Second, despite the specificity related to renal processes, we also identified several SNPs that are associated with eGFR in diabetes, and our pathway analyses uncovered gene sets associated with glucose transporter activity and abnormal glucose homeostasis. Uncovering *bona fide* genetic loci for diabetic CKD has been difficult. We have now identified a total of 19 SNPs that demonstrate at least nominal association with eGFR in diabetes. The diabetes population is at particularly high risk of CKD, and identifying kidney injury pathways may help develop new treatments for diabetic CKD.

Finally, even though CKD is primarily a disease of the elderly, our pathway enrichment analyses highlight developmental processes relevant to the kidney and the urogenital tract. Kidney disease has been long thought to have developmental origins, in part related to early programming (Barker hypothesis)^27^, low birth weight, nephron endowment and early growth and early-life nutrition^28^. Our pathway enrichment analyses suggest that developmental pathways such as placental morphology, kidney weight and embryo size, as well as protein complexes of importance in renal development may in part contribute to the developmental origins of CKD.

A limitation of our work is that causal variants and precise molecular mechanisms underlying the observed associations were not identified and will require additional experimental follow-up projects. Our attempt to gain insights into potentially causal genes through knockdown in zebrafish did not yield any clear CKD candidate gene, although the absence of a zebrafish phenotype upon gene knockdown does not mean that the gene cannot be the one underlying the observed association signal in humans. Finally, our conclusions that eGFRcrea-associated SNPs regulate the expression of nearby genes specifically in kidney epithelial cells are based on DHSs, H3K4me3 chromatin marks and chromatin annotation maps. Since these analyses rely mostly on variant positions, additional functional investigation such as luciferase assay that assess transcriptional activity more directly are likely to gain additional insights into the variants’ mechanism of action.

The kidney specificity for loci we identified may have important translational implications, particularly since our DHS and chromatin annotation analyses suggest that at least a set of gene regulatory mechanisms is important in the adult kidney. Kidney-specific pathways are important for the development of novel therapies to prevent and treat CKD and its progression with minimal risk of toxicity to other organs. Finally, the biologic insights provided by these new loci may help elucidate novel mechanisms and pathways implicated not only in CKD but also of kidney function in the physiological range.

In conclusion, we have confirmed 29 genomic loci and identified 24 new loci in association with kidney function that together highlight target organ-specific regulatory mechanisms related to kidney function.

## Methods

### Overview

This was a collaborative meta-analysis with a distributive data model. Briefly, an analysis plan was created and circulated to all participating studies. Studies then uploaded study-specific data centrally; files were cleaned, and a specific meta-analysis for each trait was performed. Details regarding each step are provided below. All participants in all discovery and replication studies provided informed consent. Each study had its research protocol approved by the local ethics committee.

### Phenotype definitions

Serum creatinine was measured in each discovery and replication study as described in Supplementary Tables 16 and 17, and statistically calibrated to the US nationally representative National Health and Nutrition Examination Study data in all studies to account for between-laboratory variation^9^,^29^,^30^. eGFRcrea was estimated using the four-variable Modification of Diet in Renal Disease Study Equation. Cystatin C, an alternative biomarker for kidney function, was measured in a sub-set of participating studies. eGFRcys was estimated as 76.7 × (serum cystatin C)^−1.19^ (ref. 31). eGFRcrea and eGFRcys values <15 ml min per 1.73 m^2^ were set to 15, and those >200 were set to 200 ml min^−1^ per 1.73 m^2^. CKD was defined as eGFRcrea <60 ml  min^−1^ per 1.73 m^2^.

Diabetes was defined as fasting glucose ⩾126 mg dl^−1^, pharmacologic treatment for diabetes or by self-report. In all studies, diabetes and kidney function were assessed at the same point in time.

### Genotypes

Genotyping was conducted in each study as specified in Supplementary Tables 18 and 19. After applying appropriate quality filters, 45 studies used markers of highest quality to impute ∼2.5 million SNPs, based on European-ancestry haplotype reference samples (HapMap II CEU). Four studies based their imputation on the 1000 Genomes Project data. Imputed genotypes were coded as the estimated number of copies of a specified allele (allelic dosage).

### Genome-wide association analysis

By following a centralized analysis plan, each study regressed sex- and age-adjusted residuals of the logarithm of eGFRcrea or eGFRcys on SNP dosage levels. Logistic regression of CKD status was performed on SNP dosage levels adjusting for sex and age. For all traits, adjustment for appropriate study-specific features, including study site and genetic principal components was included in the regression and family-based studies appropriately accounted for relatedness.

### Stage 1 discovery meta-analysis

GWAS of eGFRcrea were contributed by 48 studies (total sample size, *N*=133,413); 45 studies contributed GWAS data for the non-diabetes subgroup (*N*=118,448) and 39 for the diabetes group (*N*=11,522). GWAS of CKD were comprised by 43 studies, for a total sample size of 117,165, including 12,385 CKD cases. GWAS of eGFRcys were comprised by 16 studies for a total sample size of 32,834. All GWAS files underwent quality control using the GWAtoolbox package^32^ in R, before including them into the meta-analysis. Genome-wide meta-analysis was performed with the software METAL^33^, assuming fixed effects and using inverse-variance weighting. The genomic inflation factor *λ* was estimated for each study as the ratio between the median of all observed test statistics (*b*/s.e.)^2^ and the expected median of a *χ*^2^ with 1 degree of freedom, with *b* and s.e. representing the effect of each SNP on the phenotype and its standard error, respectively^34^. Genomic-control (GC) correction was applied to *P* values and s.e.’s in case of *λ*>1 (first GC correction). SNPs with an average minor allele frequency (MAF) of ⩾0.01 were used for the meta-analysis. To limit the possibility of false positives, after the meta-analysis, a second GC correction on the aggregated results was applied. Between-study heterogeneity was assessed through the *I*^2^ statistic.

After removing SNPs with MAF of <0.05 and which were available in <50% of the studies, SNPs with a *P* value of ≤10^−6^ were selected and clustered into independent loci through LD pruning based on an *r*^2^ of ≤0.2 within a window of ±1 MB to each side of the index SNP. After removing loci containing variants that have been previously replicated at a *P* value of 5.0 × 10^−8^ (refs 7, 8), the SNP with the lowest *P* value within each locus was selected for replication (‘index SNP’). If a SNP had an association *P* value of ≤10^−6^ with more than one trait, the trait where the SNP had the lowest *P* value was selected as discovery trait/stratum. Altogether, this resulted in 48 SNPs: 34 from eGFRcrea, 9 from eGFRcrea among those without diabetes, 4 from CKD and 1 from eGFRcys.

### Stage 2 replication analysis

*In silico* replication analysis for any of the studied traits was carried out using eight independent studies whose genotyping platforms are provided in Supplementary Table 19. *De novo* genotyping was performed in seven additional studies (*N*=22,850 individuals) of European ancestry (Supplementary Table 20), including the Bus Santé, ESTHER, KORA-F3 (subset of F3 without GWAS), KORA-F4 (subset of F4 without GWAS), Ogliastra Genetic Park (OGP, without Talana whose GWAS was included in the discovery analysis), SAPHIR and SKIPOGH studies (Supplementary Table 20). Summarizing all *in silico* and *de novo* replication studies (Supplementary Table 1), replication data for eGFRcrea were contributed by 14 studies (total sample size=42,166), which also contributed eGFRcrea results from non-diabetes (13 studies, *N*=36,433) and diabetes samples (13 studies, *N*=4,955). Thirteen studies contributed replication data on CKD (*N*=33,972; 4,245 CKD cases; studies with <50 CKD cases were excluded) and five on eGFRcys (*N*=14,930).

Association between eGFRcrea, CKD and eGFRcys and each of the 48 SNPs in the replication studies was assessed using the same analysis protocol detailed for the discovery studies above. Quality control of the replication files was performed with the same software as described above.

We performed a combined fixed-effect meta-analysis of the double-GC corrected results from the discovery meta-analysis and the replication studies, based on inverse-variance weighting. The total sample size in the combined analysis of eGFRcrea was 175,579 subjects (154,881 in the non-diabetes stratum and 16,477 in the diabetes stratum; the sum of these two sample sizes is smaller than the sample size of the overall analysis because some studies did not contribute both strata), 151,137 samples for CKD (16,630 CKD cases) and 47,764 for eGFRcys. Three criteria were used to ensure validity of novel loci declared as significant: (1) *P* value from the combined meta-analysis ≤5.0 × 10^−8^ in accordance with previously published guidelines^35^; (2) direction-consistent associations of the beta coefficients in stage 1 and stage 2 (one-sided *P* values were estimated to test for consistent effect direction with the discovery stage); (3) *q*-value <0.05 in the replication stage. Q-values were estimated using the package QVALUE^36^ in R. The tuning parameter lambda for the estimation of the overall proportion of true null hypotheses, π0, was estimated using the bootstrap method^37^. When the third criterion was not satisfied, the locus was declared ‘suggestive’.

### Power analysis

With the sample size achieved in the combined analysis of stage 1 and stage 2 data, the power to assess replication at the canonical genome-wide significance level of 5.0 × 10^−8^ was estimated with the software QUANTO^38^ version 1.2.4, assuming the same MAF and effect size observed in the discovery sample. Power to replicate associations ranged from 87 to 100% for eGFRcrea associated SNPs (median=98%), from 72 to 96% for the CKD-associated SNPs, and was equal to 59% for the eGFRcys-associated SNP (Supplementary Table 3).

### Associations stratified by diabetes and hypertension status

For all the 24 novel and 29 known SNPs, the difference between the SNP effect on eGFRcrea in the diabetes versus the non-diabetes groups was assessed by means of a two-sample *t*-test for correlated data at a significance level of 0.05. We used the following two-sample *t*-test for correlated data:




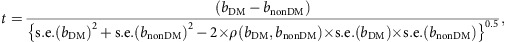




where *b*_DM_ and *b*_nonDM_ represent the SNP effects on log(eGFRcrea) in the two groups, s.e. is the standard error of the estimate and *ρ(.)* indicates the correlation between effects in the two groups, which was estimated as 0.044 by sampling 100,000 independent SNPs from our DM and nonDM GWAS, after removing known and novel loci associated with eGFRcrea. For a large sample size, as in our case, *t* follows a standard normal distribution.

A similar analysis was performed to compare results in subjects with and without hypertension, based on results from our previous work^7^. The correlation between the two strata was of 0.01.

### Proportion of phenotypic variance explained

The percent of phenotypic variance explained by novel and known loci was estimated as 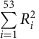, where 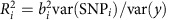 is the coefficient of determination for each of the 53 individual SNPs associated with eGFRcrea uncovered to date (24 novel and 29 known ones), *b*_*i*_ is the estimated effect of the i^th^ SNP on *y*, *y* corresponds to the sex- and age-adjusted residuals of the logarithm of eGFRcrea and var(SNP_i_)=2 × MAF_SNPi_ × (1−MAF_SNPi_)^39^. Var(*y*) was estimated in the ARIC study and all loci were assumed to have independent effects on the phenotype.

### Test for SNP associations with related traits

We performed evaluations of SNP association with results generated from consortia investigating other traits. Specifically, we evaluated systolic and diastolic blood pressure in ICBP^17^, myocardial infarction in CARDiOGRAM^18^, left ventricular mass^19^, heart failure^20^, the urinary albumin to creatinine ratio (CKDGen consortium, personal communication) and fasting plasma glucose in MAGIC^21^. In total, we performed 165 tests, corresponding to 7 traits tested for association against each of the 24 novel SNP, with the exception of myocardial infarction for which results from 3 SNPs were not available (Supplementary Table 6). Significance was evaluated at the Bonferroni corrected level of 0.05/165=0.0003.

### Lookup of replicated loci in the NHGRI GWAS catalog

All replicated SNPs, as well as SNPs in LD (*r*^2^>0.2) within ±1 MB distance were checked for their association with other traits according to the NHGRI GWAS catalog^40^ (accessed April 14, 2014).

### SNP assessments in other ethnic groups

We performed cross-ethnicity SNP evaluations in participants of African ancestry from a meta-analysis of African ancestry individuals and from participants of Asian descent from the AGEN consortium^11^.

### African ancestry meta-analysis

We performed fixed-effect meta-analysis of the genome-wide association data from 12 African ancestry studies (Supplementary Table 8) with imputation to HapMap reference panel, based on inverse-variance weighting using METAL. Only SNPs with MAF ⩾0.01 and imputation quality *r*^2^⩾0.3 were considered for the meta-analysis. After meta-analysis, we removed SNPs with MAF <0.05 and which were available in <50% of the studies. Statistical significance was assessed at the standard threshold of 5.0 × 10^−8^. Genomic control correction was applied at both the individual study level before meta-analysis and after the meta-analysis.

### Transethnic meta-analysis

We performed a trans-ethnic meta-analysis of GWAS data from cohorts of different ethnic backgrounds using MANTRA (Meta-Analysis of Trans-ethnic Association studies) software^22^. We combined the 48 European ancestry studies that contributed eGFRcrea, which were included in stage 1 discovery meta-analysis, and the 12 African ancestry studies mentioned above for a total sample size of 150,253 samples. We limited our analysis to biallelic SNPs with MAF ⩾0.01 and imputation quality *r*^2^⩾0.3. Relatedness between the 60 studies was estimated using default settings from up to 5.9 million SNPs. Only SNPs that were present in more than 25 European ancestry studies and 6 African ancestry studies (total sample size ⩾120,000) were considered after meta-analysis. Genome-wide significance was defined as a log_10_ Bayes’ Factor (log_10_BF) ⩾6 (ref. 41).

### Gene Relationships Across Implicated Loci (GRAIL)

To prioritize the gene(s) most likely to give rise to association signals in a given region, the software GRAIL was used^25^. The index SNP of all previously known kidney function associated regions, as well as the novel SNPs identified here was used as input, using the CEU HapMap (hg18 assembly) and the functional datasource text_2009_03, established before the publication of kidney function-related GWAS. Results from GRAIL were used to prioritize genes for follow-up functional work.

### Expression quantitative trait loci analysis

We identified alias rsIDs and proxies (*r*^2^>0.8) for our index SNPs using SNAP software across 4 HapMap builds. SNP rsIDs and aliases were searched for primary SNPs and LD proxies against a collected database of expression SNP (eSNP) results. The collected eSNP results met criteria for statistical thresholds for association with gene transcript levels in their respective original analyses (for references see Supplementary Table 11). Correlation of selected eSNPs to the best eSNPs per transcript per expression quantitative trait loci (eQTL) data set were assessed by pairwise LD. All results are reported in Supplementary Table 11.

### DEPICT analysis

In this work, we first used PLINK^42^ to identify independently associated SNPs using all SNPs with eGFRcrea association *P* values <10^−5^ (HapMap release 27 CEU data^43^; LD *r*^2^ threshold=0.01; physical kb threshold=1,000). We then used the DEPICT method^23^ to construct associated regions by mapping genes to independently associated SNPs if they overlapped or resided within LD (*r*^2^>0.5) distance of a given associated SNP. After merging overlapping regions and discarding regions that mapped within the major histocompatibility complex locus (chromosome 6, base pairs 20,000,000–40,000,000), 124 non-overlapping regions remained that covered a total of 320 genes. Finally, we ran the DEPICT software program on the 124 regions to prioritize genes that may represent promising candidates for experimental follow up studies, identify reconstituted gene sets that are enriched in genes from associated regions and therefore may provide insight into general kidney function biology, and identify tissue and cell-type annotations in which genes from associated regions are highly expressed. Specifically, for each tissue, the DEPICT method performs a *t*-test comparing the tissue-specific expression of eGFRcrea-associated genes and all other genes. Next, for each tissue, empirical enrichment *P* values are computed by repeatedly sampling random sets of loci (matched to the actual eGFRcrea loci by gene density) from the entire genome to estimate the empirical mean and s.d. of the enrichment statistic’s null distribution. To visualize the nineteen reconstituted gene sets with *P* value <1e−5 (Fig. 4), we estimated their overlap by computing the pairwise Pearson correlation coefficient *ρ* between each pair of gene sets followed by discretization into one of three bins; 0.3≤*ρ*<0.5, low overlap; 0.5≤*ρ*<0.7, medium overlap; *ρ*⩾0.7, high overlap.

### DNase I hypersensitivity analysis

The overlap of SNPs associated with eGFRcrea at *P*<10^−4^ with DHSs was examined using publically available data from the Epigenomics Roadmap Project and ENCODE. In all, DHS mappings were available for 123 mostly adult cells and tissues^44^ (downloaded from http://hgdownload.cse.ucsc.edu/goldenPath/hg19/encodeDCC/wgEncodeUwDnase/). The analysis here pertains to DHS’s defined as ‘broad’ peaks, which were available as experimental replicates (typically duplicates) for the majority of cells and tissues.

SNPs from our stage 1 eGFRcrea GWAS meta-analysis were first clumped in PLINK^42^ in windows of 100 kb and maximum *r*^2^ of 0.1 using LD relationships from the 1,000 Genomes EUR panel (phase I, v3, 3/14/2012 haplotypes) using a series of *P* value thresholds (10^−4^, 10^−6^, 10^−8^, ... and 10^−16^). LD proxies of the index SNPs from the clumping procedure were then identified by LD tagging in PLINK with *r*^2^=0.8 in windows of 100 kb, again using LD relationships in the 1000G EUR panel, restricted to SNPs with MAF >1% and also present in the HapMap2 CEU population. A reference set of control SNPs was constructed using the same clumping and tagging procedures applied to NHGRI GWAS catalog SNPs (available at http://www.genome.gov/gwastudies/, accessed 13 March 2013) with discovery *P* values <5.0 × 10^−8^ in European populations. In total, there were 1,204 such reference SNPs after LD pruning. A small number of reference SNPs or their proxies overlapping with the eGFRcrea SNPs or their proxies were excluded. For each cell-type and *P* value threshold, the enrichment of eGFR SNPs (or their LD proxies) mapping to DHSs relative to the GWAS catalog reference SNPs (or their LD proxies) was expressed as an odds ratio from logistic mixed effect models that treated the replicate peak determinations as random effects (lme4 package in R). Significance for enrichment odds ratio was derived from the significance of beta coefficients for the main effects in the mixed models.

### Interrogation of human kidney chromatin annotation maps

Different chromatin modification patterns can be used to generate tissue-specific chromatin-state annotation maps. These can serve as a valuable resource to discover regulatory regions and study their cell-type-specific distributions and activities, which may help with the interpretation especially of intergenic variants identified in association studies^45^. We therefore investigated the genomic mapping of the known and replicated novel index SNPs, as well as their perfect LD proxies (*n*=173, *r*^2^=1 for proxies) using a variety of resources, including chromatin maps generated from human kidney tissue cells (HKC-E cells). Chromatin immune-precipitation sequencing (ChIP-seq) data from human kidney samples were generated by NIH Roadmap Epigenomics Mapping Consortium^46^. Briefly, proximal tubule cells derived from an adult human kidney were collected and cross-linked with 1% formaldehyde. Subsequently, ChIP-seq was conducted using whole-cell extract from adult kidney tissue as the input (GSM621638) and assessing the following chromatin marks: H3K36me3 (GSM621634), H3K4me1 (GSM670025), H3K4me3 (GSM621648), H3K9ac (GSM772811) and H3K9me3 (GSM621651). The MACS version 1.4.1 (model-based analysis of ChIP-Seq) peak-finding algorithm was used to identify regions of ChIP-Seq enrichment^47^. A FDR threshold of enrichment of 0.01 was used for all data sets. The resulting genomic coordinates in bed format were further used in ChromHMM v1.06 for chromatin annotation^45^. For comparison, the same genomic coordinates were investigated in chromatin annotation maps of renal tissue, as well as across nine different cell lines from the ENCODE Project: umbilical vein endothelial cells (HUVEC), mammary epithelial cells (HMEC), normal epidermal keratinocytes (NHEK), B-lymphoblastoid cells (GM12878), erythrocytic leukemia cells (K562), normal lung fibroblasts (NHLF), skeletal muscle myoblasts (HSMM), embryonic stem cells (H1 ES) and hepatocellular carcinoma cells (HepG2). We tested whether the proportion of SNPs pointing to either strong or weak enhancers in the human kidney tissue cells was different from that of the other nine tissues by means of a Fishers’ exact test for 2 × 2 tables, contrasting each of the nine cell lines listed above against the reference kidney cell line, at a Bonferroni-corrected significance level of 0.05/9=5.6 × 10^−3^.

### Functional characterization of new loci

Replicated gene regions were prioritized for functional studies using the following criteria: (1) GRAIL identification of a gene in each region of *P* value<0.05 or DEPICT, FDR <0.05); (2) an eGFRcrea to eGFRcys ratio between 0.2 and 5 with direction consistency between the beta coefficients; (3) nearest gene if the signal was located in a gene-poor region. The list of genes selected for functional work can be found in Supplementary Table 12. This same prioritization scheme was also used to assign locus names. Morpholino knockdowns were performed in zebrafish.

Zebrafish (strain Tübingen, TU) were maintained according to established Harvard Medical School Institutional Animal Care and Use Committee protocols (protocol # 04626). Male and female fish were mated (age 6–12 months) for embryo production. Embryos were injected at the one-cell stage with MOs (GeneTools) designed to block either the ATG start site or an exon–intron splice site of the target gene (Supplementary Table 21). In cases where human loci are duplicated in zebrafish, both orthologues were knocked down simultaneously by combination MO injection. MOs were injected in escalating doses at concentrations up to 250 μM. Embryos were fixed in 4% paraformaldehyde at 48 h post fertilization for *in situ* hybridization using published methods (http://zfin.org/ZFIN/Methods/ThisseProtocol.html). Gene expression was visualized using established renal markers *pax2a* (global kidney), *nephrin* (podocytes) and *slc20a1a* (proximal tubule). The number of morphant embryos displaying abnormal gene expression was compared with control embryos by means of a Fisher’s exact test.

## Additional information

**How to cite this article:** Pattaro, C. *et al*. Genetic associations at 53 loci highlight cell types and biological pathways relevant for kidney function. *Nat. Commun.* 7:10023 doi: 10.1038/ncomms10023 (2016).

## Supplementary Material

Supplementary Information Supplementary Figures 1-7, Supplementary Tables 1-21, Supplementary Note 1 and Supplementary References

## Figures and Tables

**Table 1:** The 24 novel SNPs associated with eGFRcrea in European ancestry individuals.

**SNP ID** *	**Chr.**	**Position (bp)** †	**Locus name** ‡	**Effect/Non effect allele (EAF)**	**SNP function** §	**Stage 1 (discovery)** ||		**Stage 2 (replication)**		**Combined analysis** ¶		
						**Beta**	***P*** **value**	**Beta**	***q*** **-value**	**Beta**	***P*** **value**#	***I***^**2**^ %**
*The eight loci whose smallest P value was observed in the ‘no diabetes’ group*												
rs3850625	1	201,016,296	*CACNA1S*	A/G (0.12)	Exonic, nonsyn. SNV	0.0080	2.55E−09	0.0071	5.46E−03	0.0083	6.82E−11	0
rs2712184	2	217,682,779	*IGFBP5*	A/C (0.58)	Intergenic	−0.0049	1.65E−08	−0.0055	2.06E−03	−0.0053	1.33E−10	0
rs9682041	3	170,091,902	*SKIL*	T/C (0.87)	Intronic	−0.0067	1.36E−07	−0.0046	2.33E−02	−0.0068	2.58E−08	2
rs10513801	3	185,822,353	*ETV5*	T/G (0.87)	Intronic	0.0070	3.80E−09	0.0046	1.79E−02	0.0072	1.03E−09	0
rs10994860	10	52,645,424	*A1CF*	T/C (0.19)	UTR5	0.0075	1.00E−11	0.0061	5.46E−03	0.0077	1.07E−12	2
rs163160	11	2,789,955	*KCNQ1*	A/G (0.82)	Intronic	0.0067	9.02E−09	0.0050	9.89E−03	0.0065	2.26E−09	14
rs164748	16	89,708,292	*DPEP1*	C/G (0.53)	Intergenic	0.0047	9.92E−09	0.0019	4.19E−02	0.0046	1.95E−08	17
rs8091180	18	77,164,243	*NFATC1*	A/G (0.56)	Intronic	−0.0054	1.43E−08	−0.0052	5.46E−03	−0.0060	1.28E−09	0
												
*The 16 loci whose smallest P value was observed in the ‘overall’ group*												
rs12136063	1	110,014,170	*SYPL2*	A/G (0.70)	Intronic	0.0049	2.33E−07	0.0028	2.31E−02	0.0045	4.71E−08	0
rs2802729	1	243,501,763	*SDCCAG8*	A/C (0.43)	Intronic	−0.0050	7.37E−08	−0.0029	2.05E−02	−0.0046	2.20E−08	9
rs4667594	2	170,008,506	*LRP2*	A/T (0.53)	Intronic	−0.0045	2.37E−07	−0.0043	5.62E−03	−0.0044	3.52E−08	4
rs6795744‡‡	3	13,906,850	*WNT7A*	A/G (0.15)	Intronic	0.0071	9.60E−09	0.0019	5.15E−02	0.0060	3.33E−08	18
rs228611	4	103,561,709	*NFKB1*	A/G (0.47)	Intronic	−0.0055	4.66E−10	−0.0060	8.91E−04	−0.0056	3.58E−12	3
rs7759001	6	27,341,409	*ZNF204*	A/G (0.76)	ncRNA intronic	−0.0053	2.64E−07	−0.0045	9.10E−03	−0.0051	1.75E−08	0
rs10277115	7	1,285,195	*UNCX*	A/T (0.23)	Intergenic	0.0095	1.05E−10	0.0079	9.03E−04	0.0090	8.72E−14	0
rs3750082	7	32,919,927	*KBTBD2*	A/T (0.33)	Intronic	0.0049	2.52E−07	0.0031	1.96E−02	0.0045	3.22E−08	2
rs6459680	7	156,258,568	*RNF32*	T/G (0.74)	Intergenic	−0.0065	1.96E−10	−0.0019	4.62E−02	−0.0055	1.07E−09	0
rs4014195	11	65,506,822	*AP5B1*	C/G (0.64)	Intergenic	0.0061	2.19E−11	0.0034	1.42E−02	0.0055	1.10E−11	0
rs10491967	12	3,368,093	*TSPAN9*	A/G (0.10)	Intronic	−0.0092	3.03E−10	−0.0106	3.93E−04	−0.0095	5.18E−14	0
rs7956634	12	15,321,194	*PTPRO*	T/C (0.81)	Intronic	−0.0068	2.46E−09	−0.0069	1.51E−03	−0.0068	7.17E−12	0
rs1106766	12	57,809,456	*INHBC*	T/C (0.22)	Intergenic	0.0062	4.67E−08	0.0058	8.79E−03	0.0061	2.41E−09	9
rs11666497	19	38,464,262	*SIPA1L3*	T/C (0.18)	Intronic	−0.0064	8.58E−08	−0.0041	1.53E−02	−0.0058	4.25E−08	24
rs6088580	20	33,285,053	*TP53INP2*	C/G (0.47)	Intergenic	−0.0055	7.17E−10	−0.0027	2.31E−02	−0.0049	1.79E−09	0
rs17216707	20	52,732,362	*BCAS1*	T/C (0.79)	Intergenic	−0.0084	5.96E−13	−0.0051	6.69E−03	−0.0077	8.83E−15	1

bp, basepairs; Chr, chromosome; EAF, effect allele frequency; eGFRcrea, eGFR based on serum creatinine; GWAS, genome-wide association studies; SNP, single-nucleotide polymorphism; UTR, untranslated region.

^*^SNPs are grouped by the stratum where the smallest *P* value in the discovery and combined analysis was observed. In the ‘no diabetes’ group, sample size/number of studies were equal to 118,448/45, 36,433/13 and 154,881/58, in the discovery, replication and combined analyses, respectively. In the ‘overall’ group, the numbers for the three analyses were equal to 133,413/48, 42,116/14 and 175,579/62, respectively.

^†^On the basis of RefSeq genes (build 37).

^‡^Conventional locus name based on relevant genes in the region as identified by bioinformatic investigation (Supplementary Table 12) or closest gene. A complete overview of the genes in each locus is given in the regional association plots (Supplementary Fig. 4).

^§^SNP function is derived from NCBI RefSeq genes and may not correspond to the named gene.

^||^Twice genomic-control (GC) corrected *P* value from discovery GWAS meta-analysis: at the individual study level and after the meta-analysis.

^¶^For random-effect estimate, see Supplementary Table 4.

^#^*P* value of the meta-analysis of the twice GC-corrected discovery meta-analysis results and replication studies.

^**^Between-study heterogeneity, as assessed by the I^2^. Q statistic *P* value >0.05 for all SNPs, except rs11666497 (*SIPA1L3*, *P* value=0.04).

^‡‡^For this SNP, the conditions for replication were not all met (*q*-value >0.05 in the replication stage).
