# Supplementary material for: Genetic associations at 53 loci highlight cell types and biological pathways relevant for kidney function
Source: Nat Commun. 2016 Jan 21;7:10023. doi: 10.1038/ncomms10023 (PMC4735748; doi:10.1038/ncomms10023)
Supplement: Supplementary Information — Supplementary Figures 1-7, Supplementary Tables 1-21, Supplementary Note 1 and Supplementary References [file ncomms10023-s1.pdf]

Supplementary Figure 1. Q-Q plots of eGFRcrea (**A**), eGFRcys (**B**), CKD (**C**), and eGFRcrea in diabetes (**D**). λ_{1000} = 1.00 for all four analyses.[1, 2]

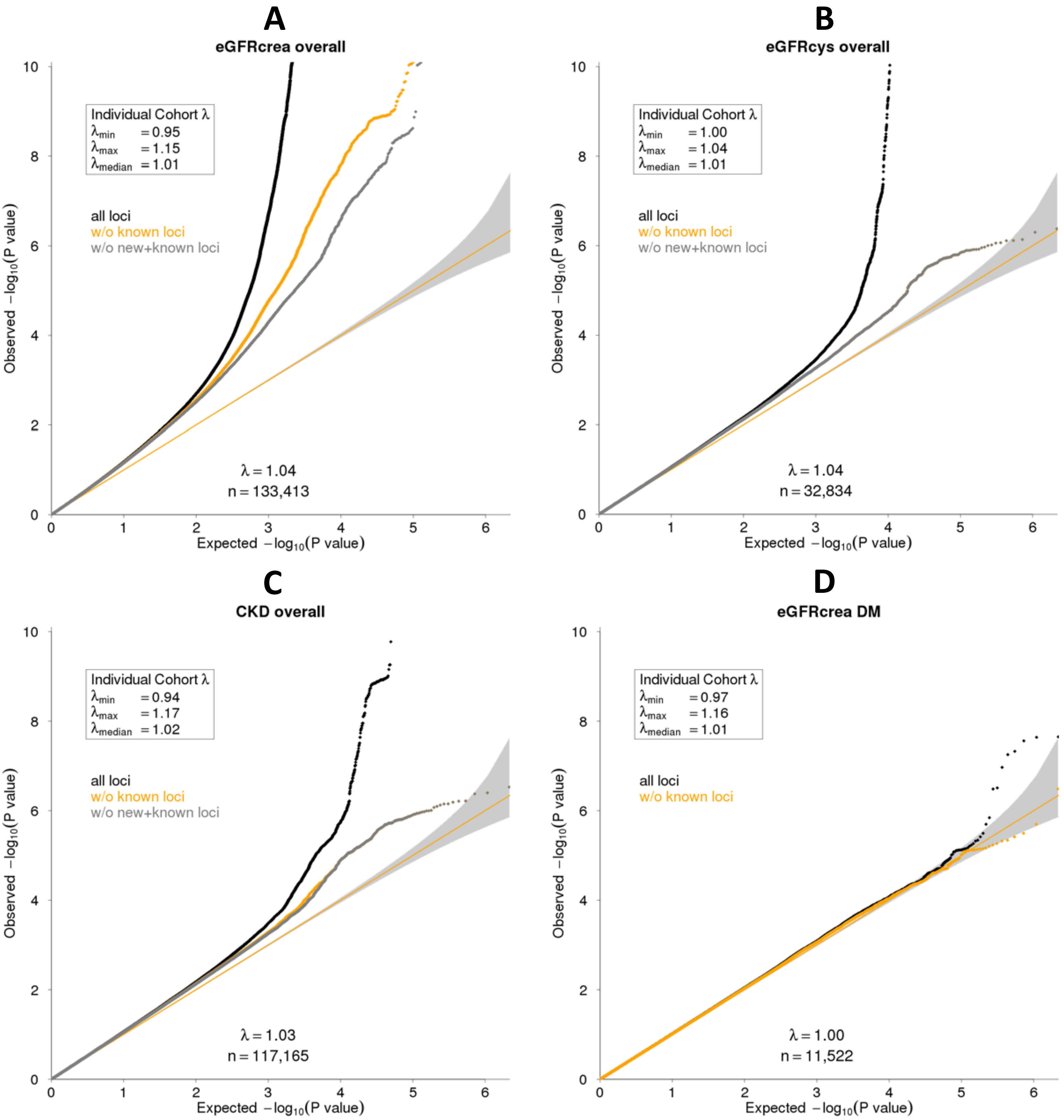

Supplementary Figure 2. Manhattan Plots of eGFRcrea in diabetes.

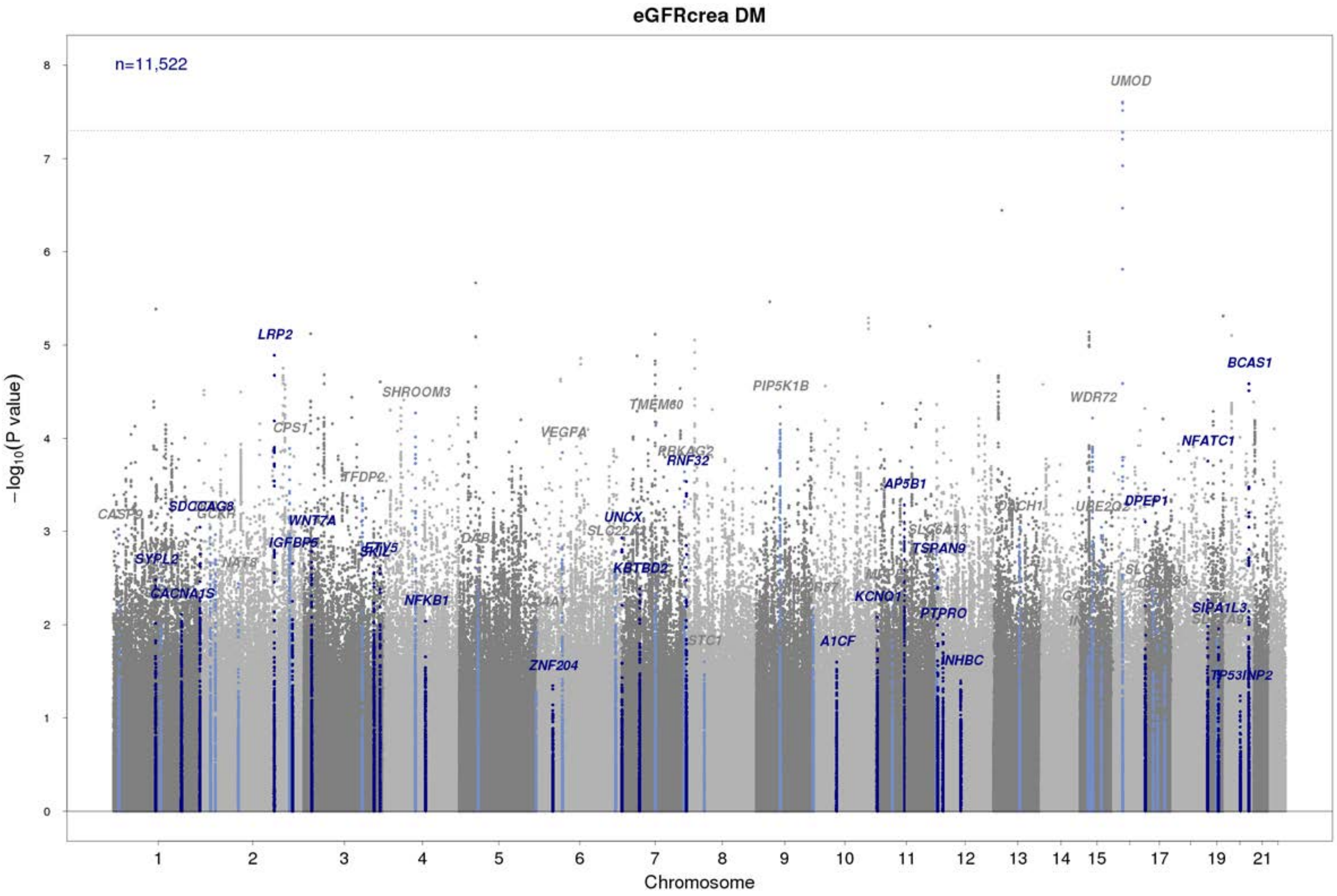

Supplementary Figure 3. All replicated loci comparing the effect sizes for eGFRcrea vs eGFRcys; known loci are in blue, new loci are in orange. 95% confidence intervals appear for the new loci only.

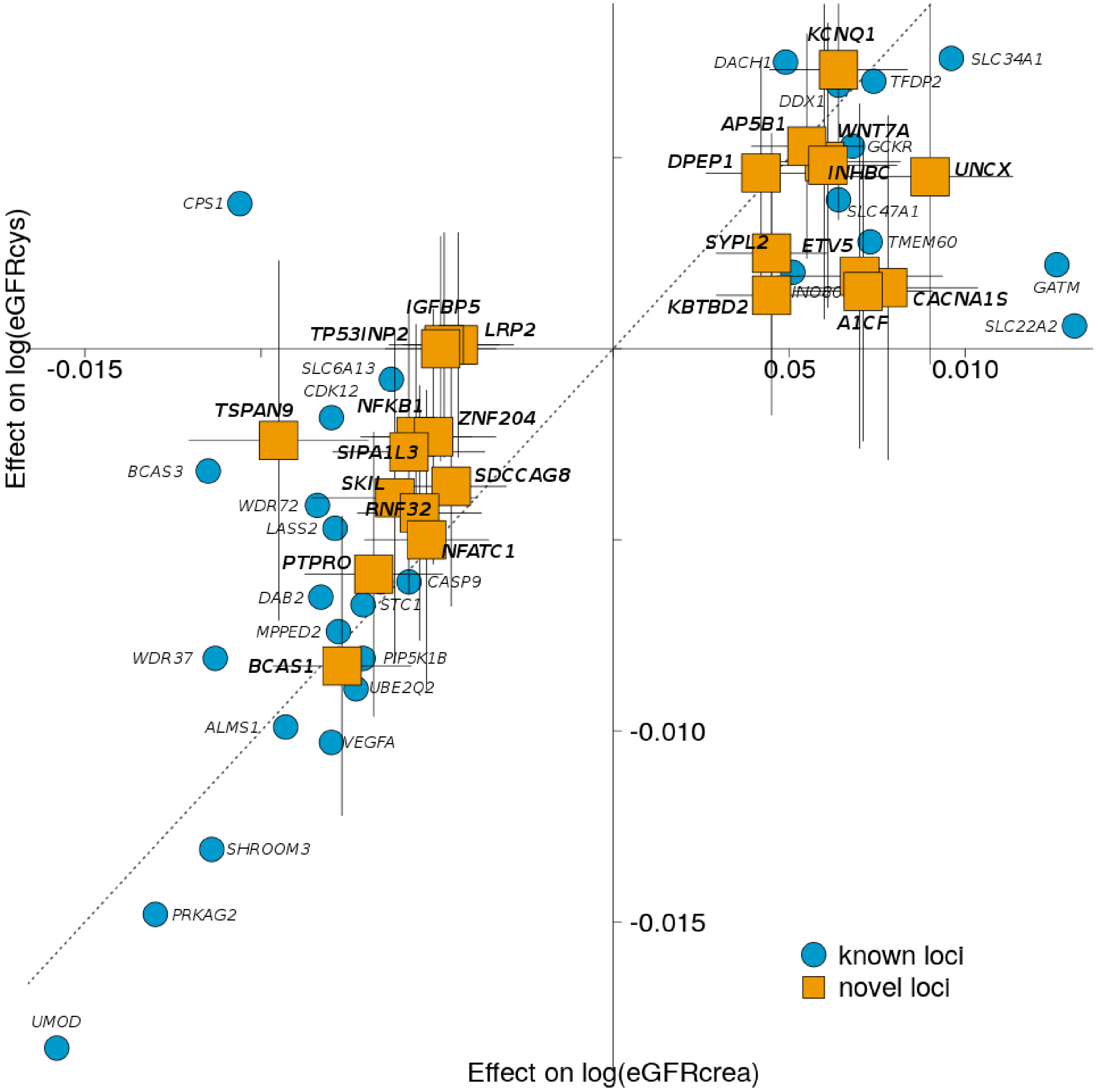

Supplementary Figure 4. Regional association plots for all 24 novel loci.

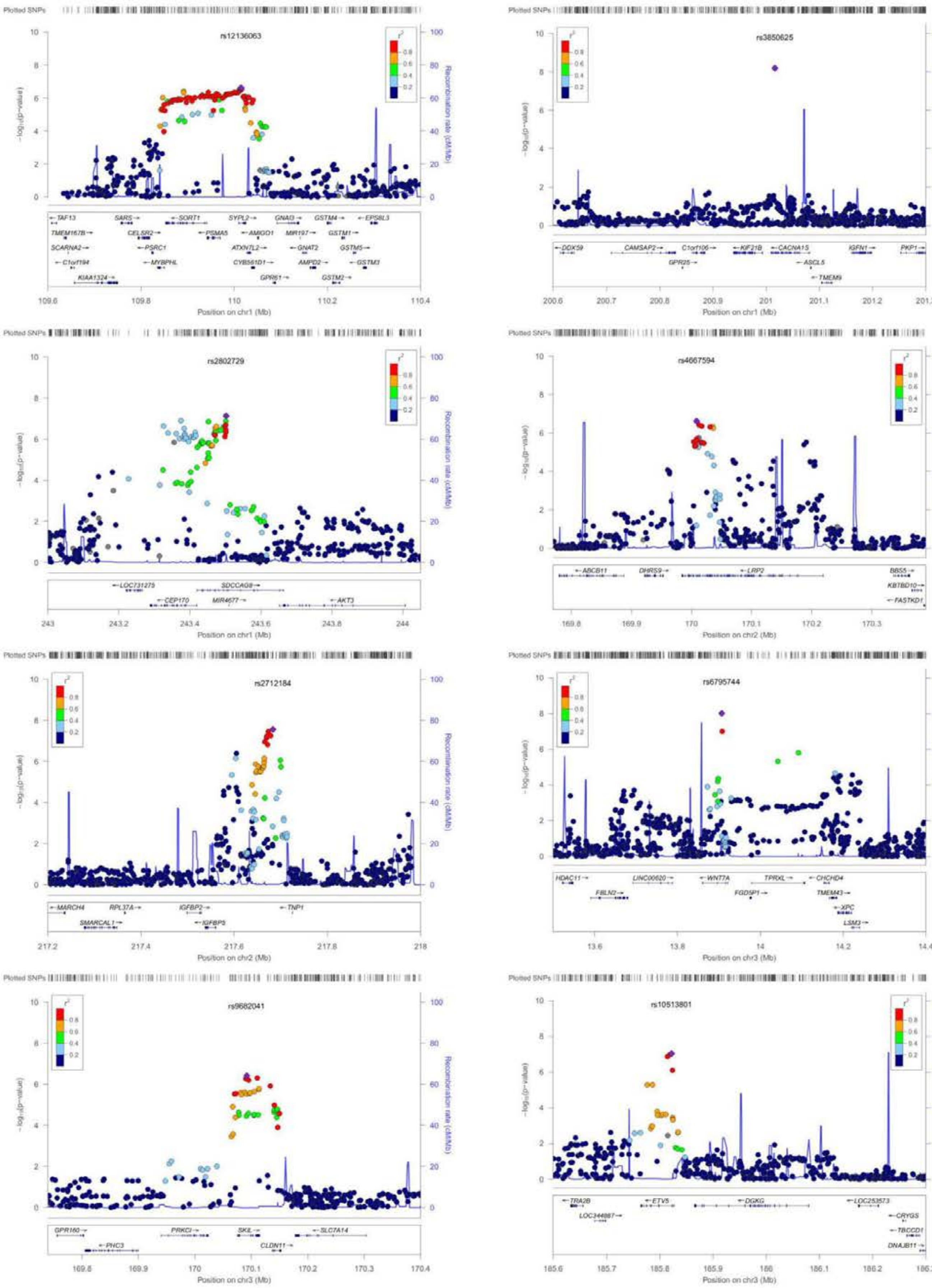

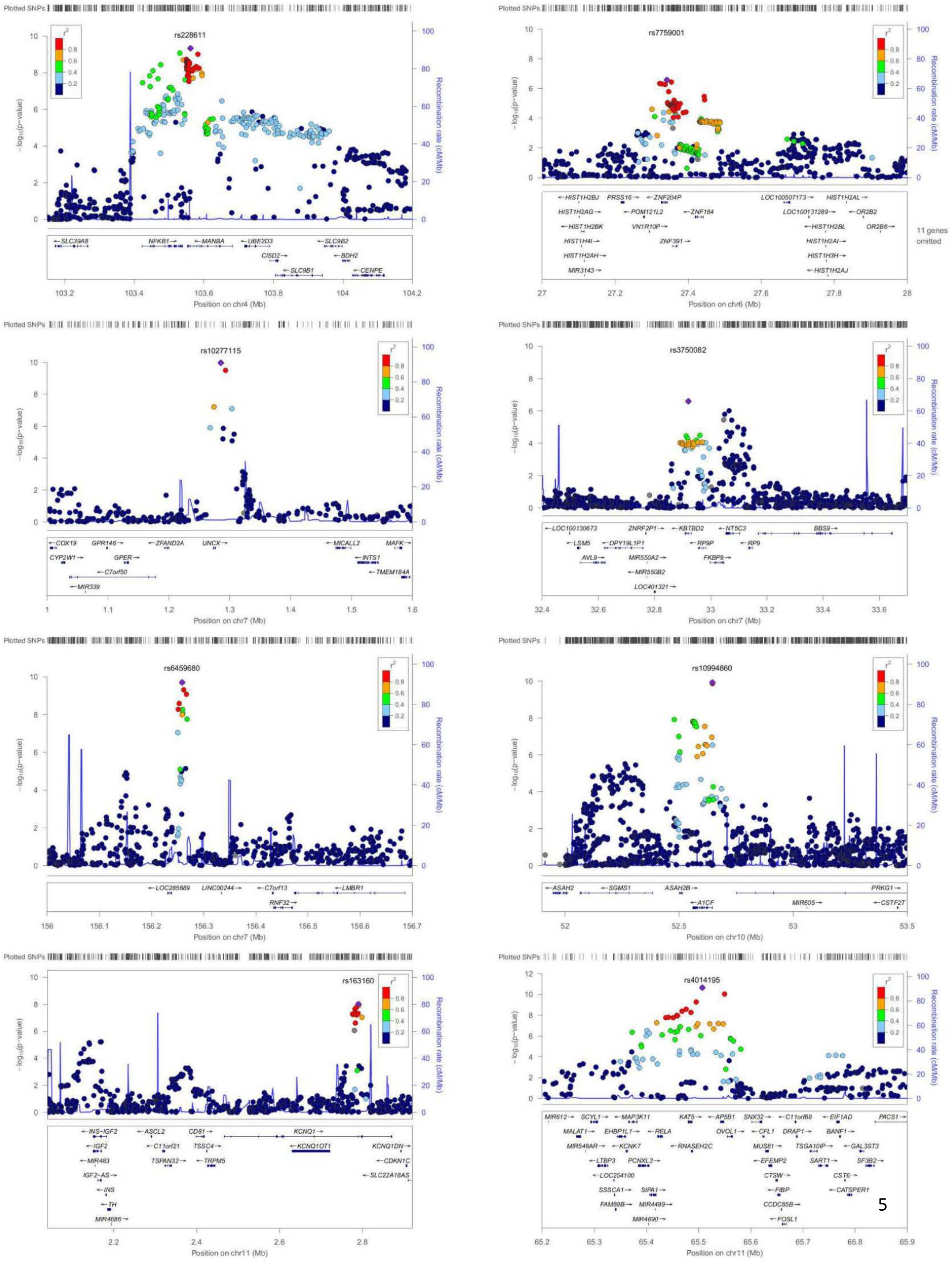
Plotted SNPs
rs228611
-log10(p-value)
Recombination rate (cM/Mb)
r2
0.8
0.6
0.4
0.2
SLC39A8
NFKB1
MANBA
UBE2D3
SLC9B2
CISD2
BDH2
SLC9B1
CENPE
Position on chr4 (Mb)
rs7759001
HIST1H2BJ
PRSS16
ZNF204P
LOC100507173
HIST1H2AL
HIST1H2AG
POM121L2
ZNF184
LOC100131289
OR2B2
HIST1H2BK
VN1R10P
HIST1H2BL
OR2B6
11 genes omitted
HIST1H4I
ZNF391
HIST1H2AI
HIST1H2AH
HIST1H3H
MIR3143
HIST1H2AJ
Position on chr6 (Mb)
rs10277115
COX19
GPR146
ZFAND2A
UNCX
MICALL2
MAFK
CYP2W1
GPER
INTS1
C7orf50
TMEM184A
MIR339
Position on chr7 (Mb)
rs3750082
LOC100130673
ZNRF2P1
KBTBD2
NT5C3
BBS9
LSM5
DPY19L1P1
RP9P
RP9
AVL9
MIR550A2
FKBP9
MIR550B2
LOC401321
Position on chr7 (Mb)
rs6459680
LOC285889
LINC00244
C7orf13
LMBR1
RNF32
Position on chr7 (Mb)
rs10994860
ASAH2
SGMS1
ASAH2B
PRKG1
A1CF
MIR605
CSTF2T
Position on chr10 (Mb)
rs163160
INS-IGF2
ASCL2
CD81
KCNQ1
IGF2
C11orf21
TSSC4
KCNQ1OT1
KCNQ1DN
MIR483
TSPAN32
TRPM5
CDKN1C
IGF2-AS
SLC22A18AS
INS
TH
MIR4686
Position on chr11 (Mb)
rs4014195
MIR612
SCYL1
MAP3K11
KAT5
AP5B1
SNX32
C11orf68
EIF1AD
PACS1
MALAT1
EHBP1L1
RELA
OVOL1
CFL1
DRAP1
BANF1
MIR548AR
KCNK7
RNASEH2C
MUS81
TSGA10IP
GAL3ST3
LTBP3
PCNXL3
EFEMP2
SART1
SF3B2
LOC254100
CTSW
CST6
SSSCA1
SIPA1
FIBP
CATSPER1
FAM89B
MIR4489
CCDC85B
MIR4690
FOSL1
Position on chr11 (Mb)

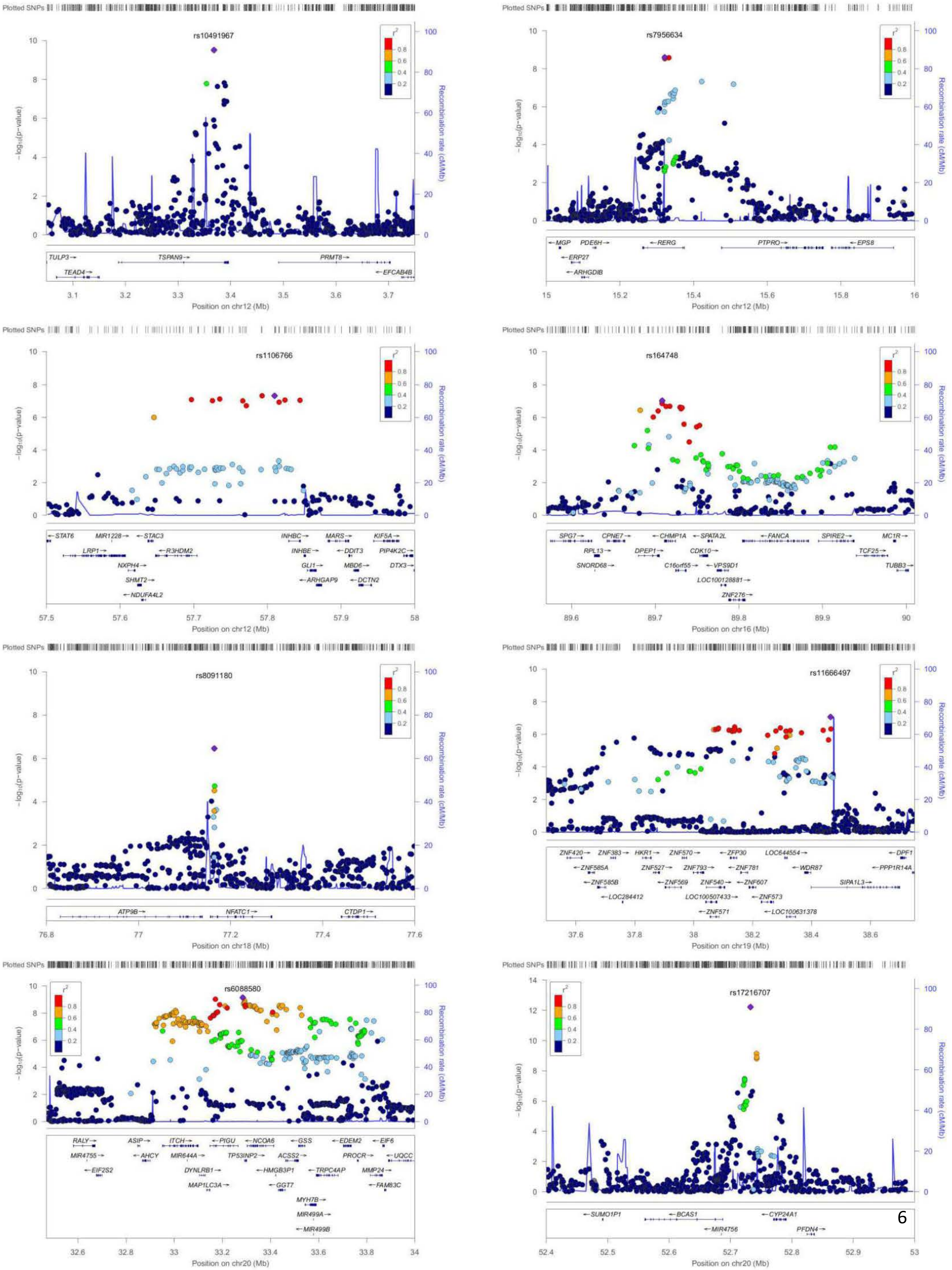
rs10491967
Position on chr12 (Mb)
rs7956634
Position on chr12 (Mb)
rs1106766
Position on chr12 (Mb)
rs164748
Position on chr16 (Mb)
rs8091180
Position on chr18 (Mb)
rs11666497
Position on chr19 (Mb)
rs6088580
Position on chr20 (Mb)
rs17216707
Position on chr20 (Mb)
Plotted SNPs
−log10(p−value)
Recombination rate (cM/Mb)
r2

Supplementary Figure 5. Effects of known and novel loci on eGFRcrea in individuals with (n=16,477) and without (n=154,881) diabetes. The Pearson's correlation coefficient between the effects in the two groups was 0.80 across all loci (95% confidence interval: 0.67, 0.88). The corresponding correlation coefficient when considering all ~2.5 million genetic variants was of 0.044 (see "Test for differential effects on eGFRcrea between diabetes and hypertension strata", in the Methods), highlighting that most of replicated loci are associated with eGFRcrea regardless of the presence or absence of diabetes.

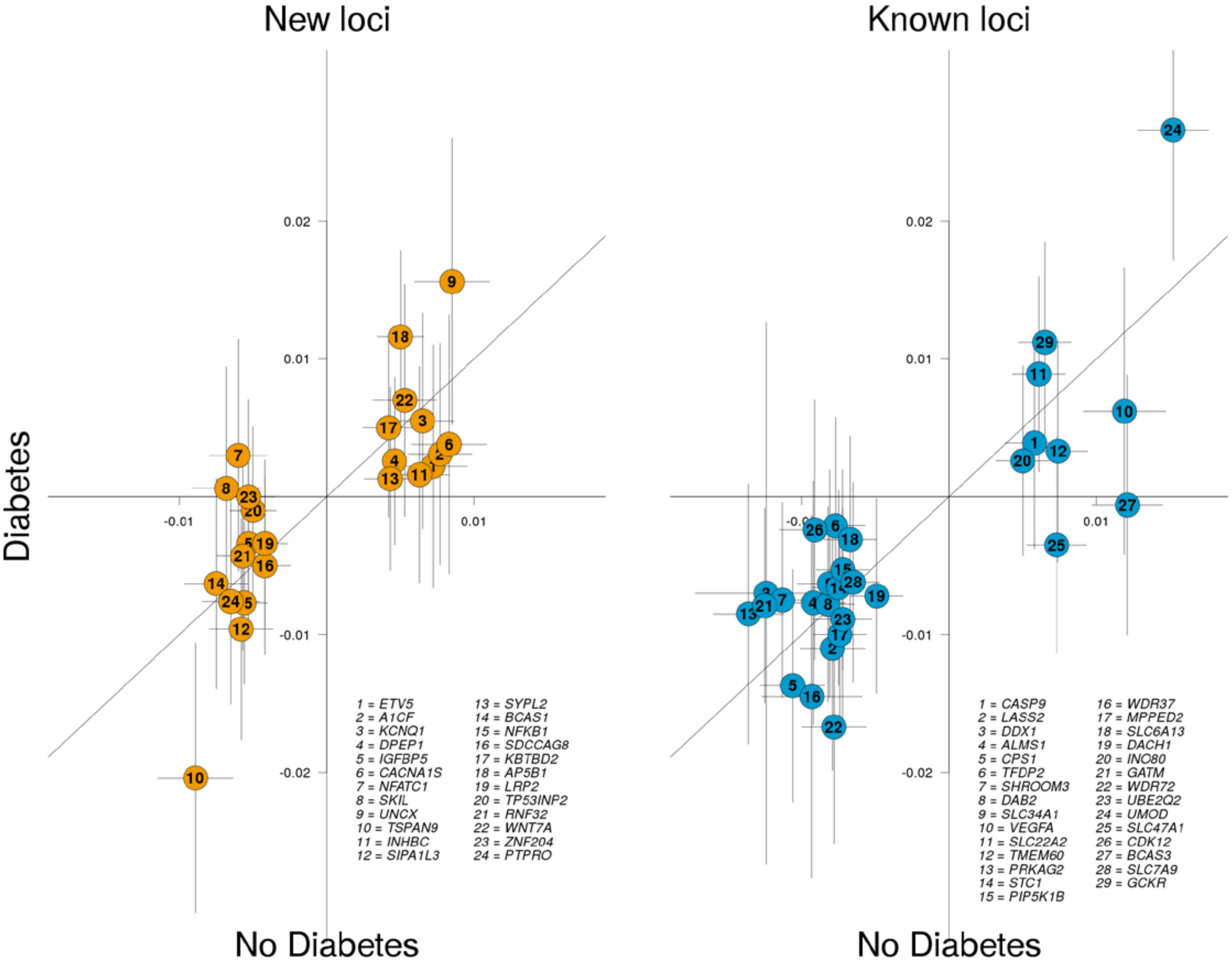

Supplementary Figure 6. Effect of the 53 known (light blue) and new (orange) loci on log(eGFRcrea) in the absence versus presence of hypertension. Data are derived from our previous analysis[3] and based on n = 29,592 and 44,319 hypertensive and non-hypertensive subjects, respectively.

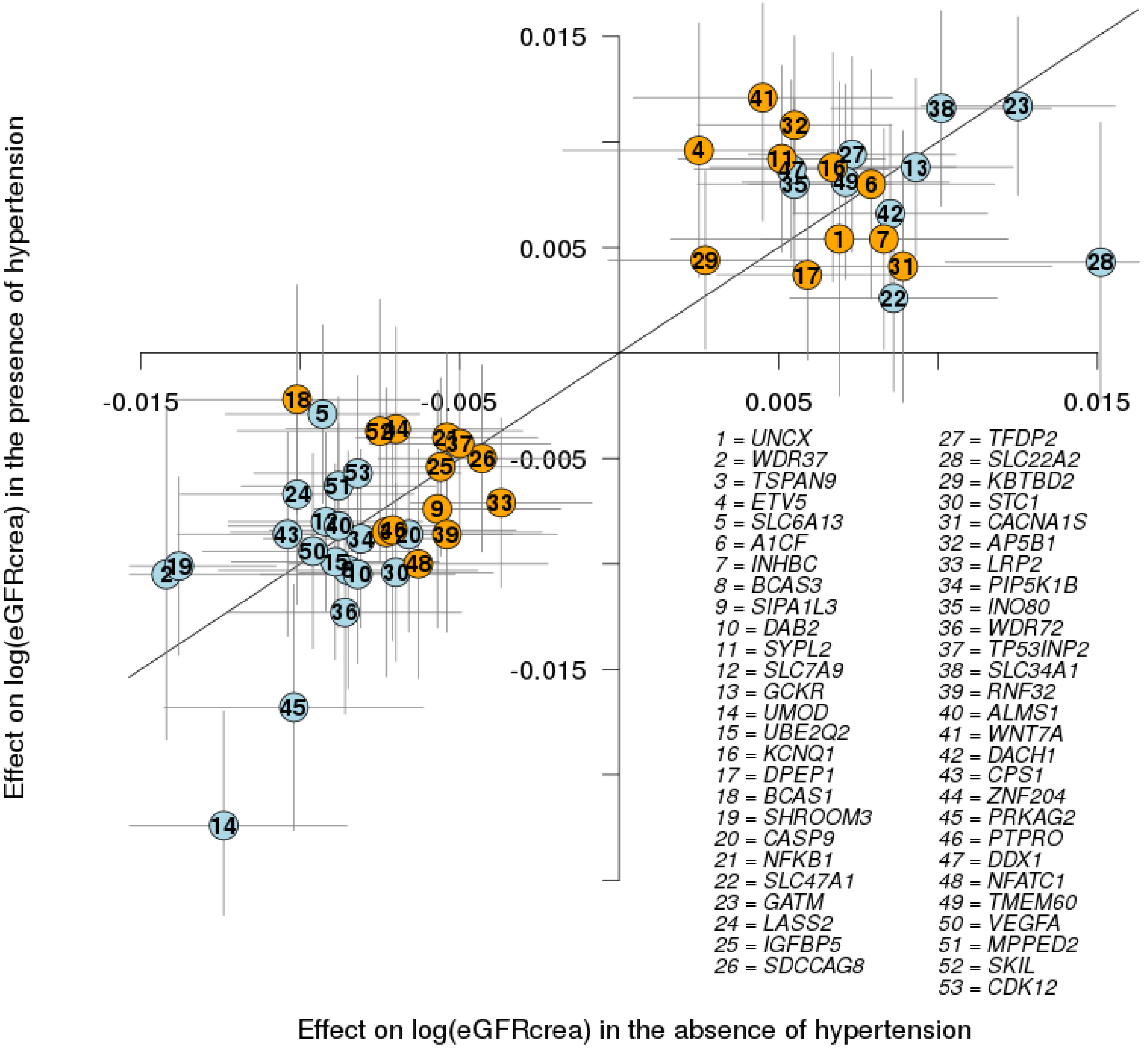

Supplementary Figure 7. Forest plots from trans-ethnic random-effect meta-analysis for all 24 novel loci. Indicated are: index SNP and effect allele; ancestry (Eu = European, As = Asian; Af = African) along with effect allele frequencies in parenthesis. Dot sizes are proportional to study sample sizes. Bars indicate 95% confidence intervals. The random-effect estimate is reported as a rhomb and as a number (b) followed by the p-value (p).

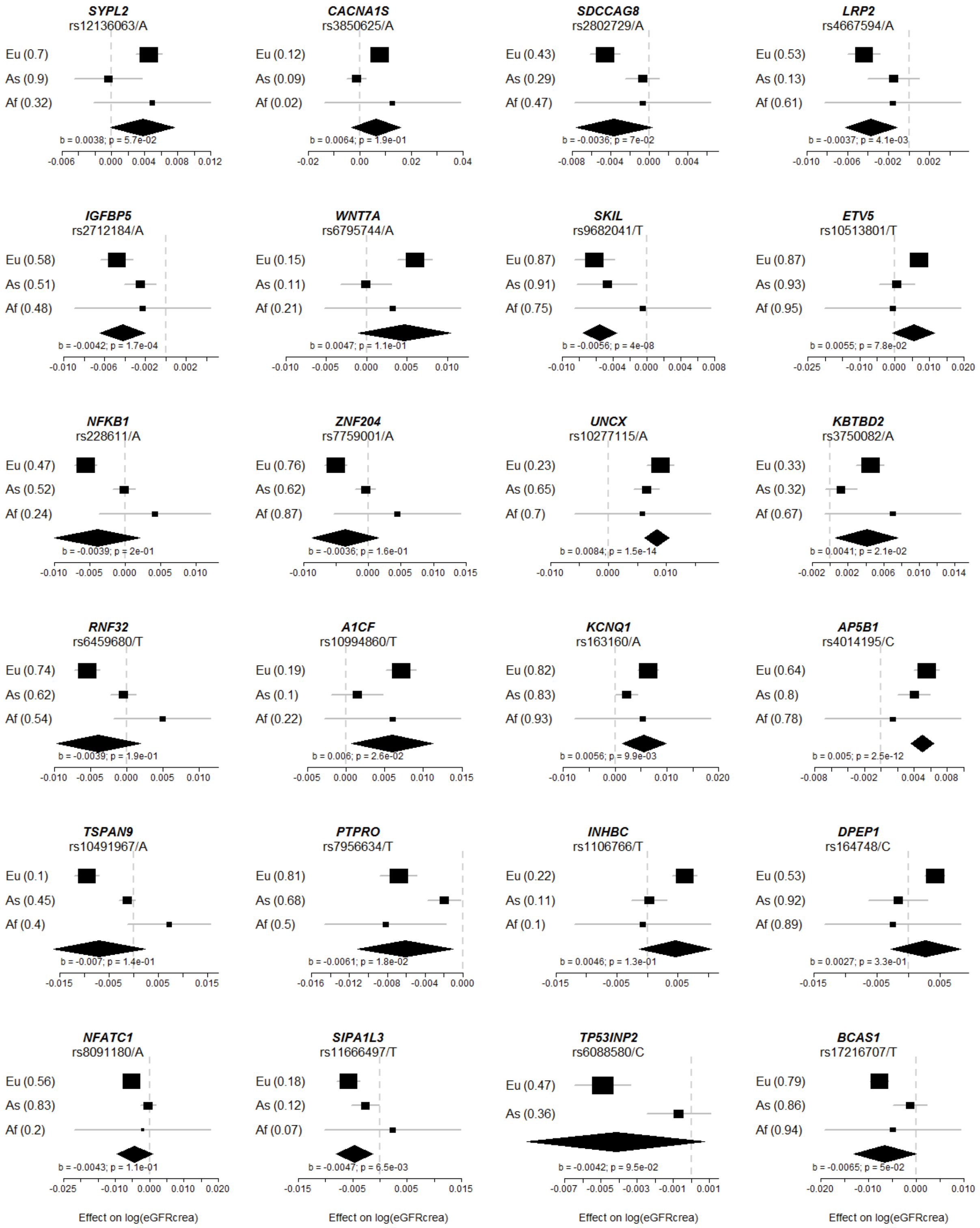

Supplementary Table 1. Sample characteristics of discovery and replication studies.

Study[1]	Sample size			Women % (N)	Age Mean (SD)	eGFRcrea Mean(SD) ml / min / $1.73m^2$	eGFRcys Mean(SD) ml / min / $1.73m^2$	CKD % (N)	HTN % (N)	DM % (N)
	eGFRcrea	CKD	eGFRcys							
**Discovery studies**										
**3C**	6431	6431	1243	60.8 (3911)	74(5)	73.1(16.9)	90.5(22.7)	20.4 (1311)	79.0 (6431)	9.7 (623)
**Advance**	2287	2287	NA	32.8 (755)	67(7)	85.1(29.2)	NA	14.7 (337)	47.6 (1096)	100.0 (2287)
**AGES**	3219	3219	NA	58.0 (1867)	76(5)	73.0(20.0)	NA	24.2 (781)	80.6 (2595)	11.5 (368)
**Amish**	1211	NA	783	48.9 (592)	49(17)	93.7(19.7)	114.9(18.0)	3.1 (37)	18.9 (229)	1.7 (20)
**ARIC**	8982	8982	7145	53.1 (4767)	62(6)	81.4(17.5)	84.2(19.7)	8.7 (782)	40.7 (3643)	14.2 (1276)
**ASPS**	848	848	NA	56.8 (482)	65(8)	96.5(39.9)	NA	8.1 (69)	72.5 (615)	9.2 (78)
**AUSTWIN**	9592	3320	NA	60.9 (5846)	46(13)	98.3(26.2)	NA	10.5 (350)	NA	NA
**BLSA**	723	723	NA	46.1 (333)	70(15)	80.3(23.1)	NA	17.4 (126)	21.9 (147)	7.7 (55)
**BMES**	2437	2437	NA	56.8 (1385)	69(9)	78.7(20.2)	NA	13.2 (322)	76.4 (1861)	10.9 (265)
**CHS**	2820	2353	2475	61.3 (1729)	72(5)	77.3(20.8)	81.0(17.9)	9.5 (224)	51.4 (1441)	11.0 (307)
**CROATIA-KORCULA**	888	888	NA	64.0 (568)	56(14)	87.3(20.6)	NA	7.5 (67)	54.2 (474)	13.1 (116)
**CROATIA-SPLIT**	478	478	NA	59.8 (286)	49(15)	104.8(23.8)	NA	5.0 (24)	39.4 (186)	5.0 (24)
**CROATIA-VIS**	768	768	NA	58.6 (450)	57(15)	88.2(22.1)	NA	6.9 (53)	52.2 (396)	12.0 (91)
**DESIR**	715	NA	NA	75.2 (550)	50(8)	92.0(16.9)	NA	0.7 (5)	NA	0.0 (0)
**EGCUT 370K**	863	NA	NA	51.2 (442)	37(16)	101.5(20.3)	NA	1.7 (15)	18.7 (161)	2.2 (19)
**EGCUT Omni**	261	261	NA	73.4 (193)	81(9)	71.2(24.1)	NA	34.2 (90)	82.5 (217)	21.8 (57)
**ERF**	2561	2561	NA	55.3 (1416)	49(14)	93.3(21.4)	NA	3.7 (96)	52.9 (1355)	6.1 (155)
**FamHS**	3838	3838	521	52.4 (2012)	52(14)	91.6(20.1)	86.3(33.5)	4.2 (161)	25.5 (979)	7.7 (291)
**FHS**	7782	4142	3002	54.3 (4229)	51(14)	92.1(21.7)	83.8(17.8)	10.7 (445)	57.5 (2390)	9.7 (405)
**GENOA**	1163	1163	NA	56.3 (655)	59(10)	87.7(24.0)	NA	10.7 (125)	73.3 (852)	15.3 (178)
**HABC**	1663	1663	1663	47.1 (784)	74(3)	71.2(14.8)	77.0(19.9)	25.0 (415)	63.7 (1060)	13.0 (216)
**HCS**	1235	1235	NA	49.6 (630)	66(7)	79.9(18.1)	NA	11.3 (144)	45 (544)	10.5 (127)
**HPFS**	818	818	NA	0.0 (0)	65(8)	85.2(22.7)	NA	9.5 (78)	59 (479)	100.0 (818)
**HYPERGENES HTN cases**	1591	1591	NA	33.0 (525)	48(10)	94.5(23.1)	NA	3.7 (59)	100.0 (1591)	0.0 (0)
**HYPERGENES HTN ctrls**	1662	1662	NA	39.6 (659)	60(10)	87.8(19.0)	NA	4.9 (81)	0.0 (0)	0.0 (0)
**INCIPE**	940	940	875	52.7 (495)	61(11)	82.6(18.5)	88.5(22.1)	8.6 (81)	69.6 (654)	10.6 (100)

Supplementary Table 1 (continued)**.**

Study[1]	Sample size eGFRcrea	Sample size CKD	Sample size eGFRcys	**Women** % (N)	**Age** Mean (SD)	**eGFRcrea** Mean(SD) ml / min / 1.73m^2	**eGFRcys** Mean(SD) ml / min / 1.73m^2	**CKD** % (N)	**HTN** % (N)	**DM** % (N)
**Discovery studies**										
**INGI-CARLANTINO**	447	NA	NA	60.8 (272)	50(16)	93.9(22.4)	NA	NA	34.9 (156)	9.4 (42)
**INGI-CILENTO**	821	821	NA	54.9 (451)	54(18)	88.7(21.8)	NA	8.0 (66)	38.4 (315)	10.5 (86)
**INGI-FVG**	874	874	NA	59.4 (519)	52(16)	90.6(21.8)	NA	6.0 (52)	48.8 (427)	6.7 (59)
**INGI-VAL BORBERA**	1636	1636	NA	55.8 (913)	55(18)	89.2(23.3)	NA	8.5 (139)	43.8 (717)	6.5 (107)
**JUPITER**	8780	8780	NA	32.2 (2827)	66(8)	80.1(18.1)	NA	11.5 (1008)	55.8 (4901)	0.4 (37)
**KORA-F3**	1641	1641	1642	50.5 (831)	62(10)	83.9(21.0)	111.8(26.3)	10.8 (177)	41.1 (674)	11.1 (179)
**KORA-F4**	1814	1814	1811	51.3 (930)	61(9)	85.1(20.2)	109.7(26.2)	7.0 (127)	20.9 (379)	9.2 (167)
**MESA**	2521	2521	2521	52.0 (1311)	63(10)	82.4(18.3)	90.0(21.7)	9.7 (245)	38.6 (974)	6.0 (151)
**MICROS**	1201	1201	1198	56.5 (678)	46(16)	94.6(20.9)	107.4(23.8)	3.8 (46)	37.7 (437)	4.3 (49)
**NESDA**	1856	NA	1856	67.5 (1253)	42(12)	98.4(20.7)	112.4(24.9)	1.1 (20)	30.3 (563)	5.1 (95)
**NHS**	786	786	NA	100.0 (786)	59(6)	86.2(22.1)	NA	10.7 (84)	70 (554)	100.0 (786)
**NSPHS**	565	565	NA	53.1 (300)	52(18)	91.0(22.1)	NA	5.7 (32)	43.4 (242)	7.8 (44)
**OGP-TALANA**	862	862	NA	57.3 (494)	51(19)	91.2(23.6)	NA	7.5 (65)	37.3 (322)	5.1 (44)
**ORCADES**	704	704	NA	53.6 (377)	54(15)	89.4(20.7)	NA	6.8 (48)	41.8 (287)	4.0 (28)
**POPGEN**	1163	1163	NA	44.4 (516)	55(14)	88.1(18.8)	NA	5.1 (59)	46.8 (541)	3.8 (44)
**PREVEND**	NA	NA	1885	48.9 (923)	50(12)	NA	105.4(25.3)	NA	33.5 (631)	3.7 (70)
**PROSPER-PHASE**	5236	5236	NA	51.7 (2718)	75(3)	72.0(21.4)	NA	29.6 (1549)	62.1 (3251)	10.4 (544)
**RS-I**	4390	4390	NA	61.4 (2696)	70(9)	77.1(17.2)	NA	13.7 (600)	34.1 (1497)	10.7 (470)
**RS-II**	1863	1863	NA	54.5 (1015)	65(8)	81.3(17.2)	NA	9.1 (169)	28.4 (530)	11.1 (207)
**SAPALDIA**	1444	1444	NA	51.0 (737)	52(11)	90.3(17.3)	NA	3.1 (44)	27.4 (389)	2.8 (40)
**SHIP**	3228	3228	3228	51.7 (1670)	54(15)	90.4(23.6)	97.1(25.3)	7.7 (248)	51.1 (1649)	11.2 (362)
**SHIP-TREND**	986	986	986	56.2 (554)	50(14)	92.4(22.1)	122.1(22.1)	4.3 (42)	39.6 (390)	1.8 (18)
**SORBS**	856	856	NA	58.5 (501)	49(16)	92.2(19.0)	NA	4.1 (35)	53.2 (455)	9.3 (80)
**WGHS**	21940	23186	NA	100.0 (21,940)	55(7)	90.0(22.5)	NA	6.1 (1329)	24.5 (5374)	2.5 (554)
**YFS**	2023	NA	NA	54.7 (1107)	38(5)	100.3(15.8)	NA	0.2 (5)	20.0 (404)	1.9 (39)

Supplementary Table 1 (continued).

Study[1]	Sample size			Women % (N)	Age Mean (SD)	eGFRcrea Mean(SD) ml / min / 1.73m^2	eGFRcys Mean(SD) ml / min / 1.73m^2	CKD % (N)	HTN % (N)	DM % (N)
	eGFRcrea	CKD	eGFRcys							
**Replication studies**										
**Bus Santé**	4408	4408	NA	49.4 (2178)	58(11)	85.6(15.6)	NA	4.3 (186)	28.3 (1249)	7.4 (327)
**EGCUT_replic**	1519	1519	1037	46.2 (703)	58(17)	97.4(29.3)	85.5(16.9)	8.8 (134)	59.5 (905)	6.3 (97)
**ESTHER**	3604	3604	NA	55.6 (2004)	62(7)	90.3(34.4)	NA	15.7 (565)	57.5 (2073)	15.9 (572)
**GENDIAN**	450	450	450	47.1 (212)	65(10)	69.1(20.2)	85.3(27.1)	32.3 (145)	53.0 (237)	100.0 (450)
**GHS 1**	2995	2995	NA	48.5 (1452)	56(11)	87.3(16.5)	NA	3.7 (112)	52.6 (1575)	7.2 (215)
**GHS 2**	1179	1179	NA	50.0 (590)	55(11)	86.7(16.2)	NA	4.5 (53)	48.4 (570)	7.6 (90)
**GSK**	1721	1721	NA	66.6 (1147)	51(13)	92.3(22.6)	NA	5.5 (95)	43.7 (752)	4.6 (80)
**HRS**	NA	NA	7700	58.9 (4537)	68(10)	NA	102.5(20.3)	2.9 (221)	66.1 (5087)	20.5 (1557)
**KORA-F3 non-GWAS**	1494	NA	1493	52.5 (787)	52(13)	92.6(21.3)	123.5(29.1)	2.6 (39)	29.4 (437)	5.1 (76)
**KORA-F4 non-GWAS**	1199	1200	1196	52.4 (629)	49(15)	92.6(22.4)	118.4(27.5)	5.8 (70)	13.3 (159)	4.0 (48)
**IPM_EA_Affy**	440	NA	NA	30.2 (133)	62(13)	94.8(36.8)	NA	19.1 (84)	60.5 (266)	26.0 (99)
**IPM_EA_Illu**	1307	1307	NA	48.6 (635)	68(9)	86.1(27.8)	NA	14.8 (194)	55.1 (720)	15.0 (185)
**LURIC**	3056	3056	3054	30.0 (917)	63(11)	86.0(21.7)	84.7(22.6)	10.0 (305)	72.7 (2223)	32.6 (996)
**OGP**	9554	5884	NA	56.9 (5440)	50(17)	98.6(35.0)	NA	8.1 (776)	36.0 (3443)	6.2 (596)
**SAPHIR**	1721	NA	NA	37.1 (639)	51(6)	91.7(16.1)	NA	6.9 (19)	55.7 (959)	3.3 (56)
**SKIPOGH**	870	NA	NA	52.3 (455)	47(18)	94.2(24.6)	NA	5.7 (50)	22.9 (198)	4.5 (39)
**Vanderbilt Omni1**	3221	3221	NA	47.3 (1525)	54(19)	80.0(36.9)	NA	27.7 (891)	70.5 (2271)	18.0 (581)
**Vanderbilt Omni5**	1129	1129	NA	46.9 (529)	50(21)	89.0(44.4)	NA	21.7 (245)	58.2 (657)	33.3 (376)
**Vanderbilt 660W**	2299	2299	NA	56.5 (1298)	56(17)	78.6(23.9)	NA	20.6 (474)	57.2 (1316)	17.9 (411)

[1] Extended study names are given in the Acknowledgements section.

Supplementary Table 2. Association of previously identified loci. For each locus, we report the best SNP from the current discovery GWAS (current best SNP). The previously reported best SNP is reported for comparison.

ID of current best SNP in the locus	Chr	Position (Build 37)	Locus name*	Ref. / Non-Ref. All. (Ref All Freq)	eGFRcrea		CKD		Previously reported SNP in the locus		
					beta(SE)†	P-value†	Odds Ratio (95%CI) †	P-value†	ID of previously reported SNP[REF]	LD# with current best SNP r^2/D'	P-value in current GWAS†
rs1800615	1	15,832,281	*CASP9*	T/C(0.30)	-0.0058(0.0009)	1.90E-09	1.04 (1.00,1.07)	3.40E-02	rs12124078[3]	1.00/1.00	3.09E-09
rs267734	1	150,951,477	*LASS2*	T/C(0.79)	-0.0079(0.0011)	4.01E-13	1.06 (1.02,1.10)	3.52E-03	same[4]		
rs807601	2	15,793,014	*DDX1*	T/G(0.34)	0.0064(0.0009)	6.60E-12	0.99 (0.96,1.02)	5.28E-01	rs6431731[3]	0.04/0.60	2.95E-07
rs1260326	2	27,730,940	*GCKR*	T/C(0.42)	0.0068(0.0009)	3.38E-14	0.98 (0.95,1.01)	1.36E-01	same[4]		
rs6546838	2	73,679,280	*ALMS1*	A/G(0.76)	-0.0093(0.0010)	7.72E-20	1.05 (1.02,1.09)	4.63E-03	rs13538[4]	0.95/1.00	3.15E-17
rs7422339	2	211,540,507	*CPS1*	A/C(0.32)	-0.0106(0.0010)	2.18E-23	1.11 (1.07,1.15)	7.54E-09	same[5]		
rs2861422	3	141,724,644	*TFDP2*	T/C(0.27)	0.0074(0.0010)	9.12E-14	0.96 (0.92,0.99)	7.59E-03	rs347685[4]	0.96/1.00	1.87E-13
rs17319721	4	77,368,847	*SHROOM3*	A/G(0.43)	-0.0114(0.0009)	1.32E-37	1.07 (1.04,1.10)	7.71E-06	same[5]		
rs11959928	5	39,397,132	*DAB2*	A/T(0.44)	-0.0083(0.0009)	1.66E-20	1.06 (1.02,1.09)	4.20E-04	same[4]		
rs6420094	5	176,817,636	*SLC34A1*	A/G(0.66)	0.0096(0.0010)	4.92E-22	0.91 (0.88,0.94)	3.68E-09	same[4]		
rs9472135	6	43,809,802	*VEGFA*	T/C(0.71)	-0.0080(0.0010)	3.34E-15	1.07 (1.04,1.11)	3.37E-05	rs881858[4]	0.88/0.96	7.51E-15
rs316009	6	160,675,764	*SLC22A2*	T/C(0.10)	0.0131(0.0014)	4.38E-19	0.96 (0.91,1.01)	9.71E-02	rs2279463[4]	0.01/1.00	2.94E-16
rs848490	7	77,555,005	*TMEM60*	C/G(0.73)	0.0073(0.0010)	7.80E-13	0.93 (0.90,0.97)	1.44E-04	rs6465825[4]	0.41/1.00	4.91E-12
rs7805747	7	151,407,801	*PRKAG2*	A/G(0.25)	-0.0130(0.0011)	7.96E-29	1.16 (1.11,1.20)	2.06E-14	same[4]		
rs3758086	8	23,714,992	*STC1*	A/G(0.42)	-0.0071(0.0009)	1.71E-15	1.06 (1.02,1.09)	4.13E-04	rs10109414[5]	1.00/1.00	2.31E-15
rs4744712	9	71,434,707	*PIP5K1B*	A/C(0.40)	-0.0071(0.0009)	4.29E-15	1.06 (1.03,1.09)	7.59E-05	same[4]		
rs1044261	10	1,065,710	*WDR37*	T/C(0.08)	-0.0113(0.0016)	1.21E-11	1.15 (1.09,1.22)	3.62E-07	rs10794720[4]	0.42/0.69	3.23E-09
rs963837	11	30,749,090	*MPPED2*	T/C(0.54)	-0.0078(0.0009)	5.69E-18	1.09 (1.05,1.12)	9.03E-08	rs3925584[3]	0.93/0.97	7.58E-18
rs10774021	12	349,298	*SLC6A13*	T/C(0.65)	-0.0063(0.0009)	4.77E-12	1.04 (1.01,1.07)	1.17E-02	same[4]		
rs716877	13	72,347,448	*DACH1*	C/G(0.40)	0.0049(0.0009)	6.22E-08	0.97 (0.94,1.00)	3.15E-02	rs626277[4]	1.00/1.00	7.98E-08
rs476633	15	41,392,134	*INO80*	C/G(0.57)	0.0051(0.0009)	8.90E-09	0.94 (0.91,0.97)	2.84E-05	rs2928148[3]	0.84/1.00	1.28E-07
rs2467853	15	45,698,793	*GATM*	T/G(0.62)	0.0126(0.0009)	1.05E-42	0.90 (0.87,0.93)	1.88E-11	rs2453533[5]	1.00/1.00	4.26E-42
rs491567	15	53,946,593	*WDR72*	A/C(0.78)	-0.0084(0.0010)	2.86E-15	1.08 (1.04,1.12)	7.48E-05	same[4]		
rs1394125	15	76,158,983	*UBE2Q2*	A/G(0.35)	-0.0073(0.0010)	5.47E-14	1.07 (1.04,1.11)	3.02E-05	same[4]		
rs13329952	16	20,366,507	*UMOD*	T/C(0.81)	-0.0158(0.0011)	9.47E-43	1.24 (1.19,1.29)	1.98E-25	rs12917707[5]	0.95/1.00	1.16E-41
rs2453580	17	19,438,321	*SLC47A1*	T/C(0.59)	0.0064(0.0009)	2.93E-11	0.94 (0.91,0.97)	2.60E-04	same[3]		
rs9916302	17	37,499,949	*CDK12 / FBXL20*	T/C(0.74)	-0.008(0.0010)	4.78E-15	1.03 (0.99,1.07)	9.43E-02	rs7208487[3]<br>rs11078903[6]	0.49/1.00<br>0.87/1.00	1.53E-12<br>5.09E-13
rs11657044	17	59,450,105	*BCAS3*	T/C(0.19)	-0.0115(0.0012)	7.89E-22	1.06 (1.02,1.10)	6.56E-03	rs9895661[4]	0.94/1.00	2.76E-21
rs12460876	19	33,356,891	*SLC7A9*	T/C(0.60)	-0.0066(0.0009)	1.86E-13	1.05 (1.02,1.08)	2.19E-03	same[4]		

*Based on previously reported index gene to facilitate comparison.

†Beta is the effect on log(eGFRcrea in ml/min/1.73 m^2); all reported standard errors, confidence intervals, and P-values, are based on the twice-GC corrected results from discovery GWAS meta-analysis.

#LD lookup is based on HapMap 22 CEU obtained using SNAP[7] version 2.2.

Supplementary Table 3. All SNPs tested for replication.

**SNPID** Locus name*	**Chr**	**Position (bp)** (Build 37)	**Eff./ Non Eff All (EAF)**[#]	**STAGE 1 (discovery)**[†]			**STAGE 2 (replication)**[‡]				**Combined analysis**					
				**N**	**Beta (SE)**	**P-value**	**N**	**Beta (SE)**	**1-sided P-value**	**q-value**	**N**	**Beta (SE)**	**P-value**	**I^2 (%)**	**Power**	**Median Rsq**
**eGFRcrea in the non-diabetes group**																
rs3850625 *CACNA1S*	1	201,016,296	A/G(0.12)	116,689	0.0086 (0.0014)	2.55E-09	36,418	0.0071 (0.0028)	5.10E-03	5.46E-03	153,107	0.0083 (0.0013)	6.82E-11	0	0.997	1.00
rs2712184 *IGFBP5*	2	217,682,779	A/C(0.58)	118,440	-0.0052 (0.0009)	1.65E-08	35,414	-0.0055 (0.0018)	1.20E-03	2.06E-03	153,854	-0.0053 (0.0008)	1.33E-10	0	0.983	1.00
rs9682041 *SKIL*	3	170,091,902	T/C(0.87)	118,454	-0.0072 (0.0013)	1.36E-07	32,457	-0.0046 (0.0031)	6.81E-02	2.33E-02	150,911	-0.0068 (0.0012)	2.58E-08	2	0.948	1.00
rs10513801 *ETV5*	3	185,822,353	T/G(0.87)	118,374	0.0079 (0.0013)	3.80E-09	36,400	0.0046 (0.0026)	3.96E-02	1.79E-02	154,774	0.0072 (0.0012)	1.03E-09	0	0.992	1.00
rs10994860 *A1CF*	10	52,645,424	T/C(0.18)	118,358	0.0082 (0.0012)	1.00E-11	36,286	0.0061 (0.0024)	5.10E-03	5.46E-03	154,644	0.0077 (0.0011)	1.07E-12	2	0.999	0.93
rs163160 *KCNQ1*	11	2,789,955	A/G(0.82)	118,373	0.0069 (0.0012)	9.02E-09	36,311	0.0050 (0.0023)	1.62E-02	9.89E-03	154,684	0.0065 (0.0011)	2.26E-09	14	0.992	0.98
rs164748 *DPEP1*	16	89,708,292	C/G(0.53)	118,373	0.0053 (0.0009)	9.92E-09	36,124	0.0019 (0.0018)	1.42E-01	4.19E-02	154,497	0.0046 (0.0008)	1.95E-08	17	0.991	0.99
rs8091180 *NFATC1*	18	77,164,243	A/G(0.56)	117,447	-0.0062 (0.0011)	1.43E-08	36,268	-0.0052 (0.0020)	5.10E-03	5.46E-03	153,715	-0.0060 (0.0010)	1.28E-09	0	0.999	0.81
rs437065 *ADAMTS5*	21	28,527,399	C/G(0.14)	118,358	0.0066 (0.0013)	8.64E-07	35,562	-0.0026 (0.0026)	1.63E-01	4.62E-02	153,920	0.0047 (0.0012)	6.78E-05	0	0.904	0.99
**eGFRcrea in the overall sample**																
rs12136063 *SYPL2*	1	110,014,170	A/G(0.70)	133,723	0.0049 (0.0009)	2.33E-07	41,703	0.0028 (0.0019)	6.48E-02	2.31E-02	175,426	0.0045 (0.0008)	4.71E-08	0	0.922	1.00
rs2802729 *SDCCAG8*	1	243,501,763	A/C(0.44)	133,608	-0.0050 (0.0009)	7.37E-08	41,200	-0.0029 (0.0018)	5.02E-02	2.05E-02	174,808	-0.0046 (0.0008)	2.20E-08	9	0.983	0.88
rs2888875 *THADA*	2	43,788,092	A/G(0.69)	129,114	0.0048 (0.0009)	7.75E-07	41,571	0.0012 (0.0018)	2.60E-01	5.72E-02	170,685	0.0041 (0.0008)	6.92E-07	0	0.894	0.99
rs17050272 *GLI2*	2	121,306,440	A/G(0.43)	132,764	-0.0048 (0.0009)	6.63E-07	41,681	-0.0024 (0.0018)	8.88E-02	2.93E-02	174,445	-0.0043 (0.0008)	1.36E-07	19	0.964	0.90
rs3820716 *ACVR2A*	2	148,680,260	A/G(0.53)	133,751	-0.0052 (0.0009)	2.73E-09	41,642	0.0006 (0.0017)	6.36E-01	1.22E-01	175,393	-0.0039 (0.0008)	1.36E-06	17	0.994	1.00
rs4667594 *LRP2*	2	170,008,506	A/T(0.53)	133,715	-0.0045 (0.0009)	2.37E-07	41,622	-0.0043 (0.0017)	5.90E-03	5.62E-03	175,337	-0.0044 (0.0008)	3.52E-08	4	0.922	1.00
rs6795744 *WNT7A*	3	13,906,850	A/G(0.15)	133,718	0.0071 (0.0012)	9.60E-09	41,772	0.0019 (0.0024)	2.10E-01	5.15E-02	175,490	0.0060 (0.0011)	3.33E-08	18	0.990	0.98

Supplementary Table 3 (continued).

SNPID<br>Locus name*	Chr	Position (bp)<br>(Build 37)	Eff./<br>Non Eff All<br>(EAF)[#]	STAGE 1 (discovery)[†]			STAGE 2 (replication)[‡]				Combined analysis					
				N	Beta (SE)	P-value	N	Beta (SE)	1-sided P-value	q-value	N	Beta (SE)	P-value	I^2 (%)	Power	Median Rsq
**eGFRcrea in the overall sample**																
rs6795744<br>*WNT7A*	3	13,906,850	A/G(0.15)	133,718	0.0071 (0.0012)	9.60E-09	41,772	0.0019 (0.0024)	2.10E-01	5.15E-02	175,490	0.0060 (0.0011)	3.33E-08	18	0.990	0.98
rs16852193<br>*EGFEM1P*	3	168,074,912	T/C(0.09)	133,691	0.0076 (0.0015)	8.72E-07	41,664	0.0005 (0.0030)	4.34E-01	8.86E-02	175,355	0.0061 (0.0014)	6.64E-06	0	0.885	0.95
rs228611<br>*NFKB1*	4	103,561,709	A/G(0.48)	133,788	-0.0055 (0.0009)	4.66E-10	41,657	-0.0060 (0.0017)	2.08E-04	8.91E-04	175,445	-0.0056 (0.0008)	3.58E-12	4	0.998	1.00
rs7735249<br>*ARL15*	5	53,310,139	C/G(0.92)	132,513	-0.0109 (0.0018)	2.09E-09	41,662	-0.0012 (0.0028)	3.28E-01	6.86E-02	174,175	-0.0079 (0.0015)	2.08E-07	8	1.000	0.98
rs11960179<br>*PIK3R1*	5	67,820,217	A/G(0.12)	133,714	-0.0072 (0.0014)	2.47E-07	23,693	-0.0051 (0.0033)	6.16E-02	2.31E-02	157,407	-0.0069 (0.0013)	1.57E-07	9	0.908	0.94
rs836788<br>*DHFR*	5	79,912,044	T/C(0.35)	133,810	-0.0045 (0.0009)	8.56E-07	41,731	-0.0038 (0.0018)	1.73E-02	9.89E-03	175,541	-0.0043 (0.0008)	9.89E-08	11	0.868	1.00
rs7759001<br>*ZNF204*	6	27,341,409	A/G(0.76)	133,723	-0.0053 (0.0010)	2.64E-07	41,760	-0.0045 (0.0020)	1.38E-02	9.10E-03	175,483	-0.0051 (0.0009)	1.75E-08	0	0.930	1.00
rs10277115<br>*UNCX*	7	1,285,195	A/T(0.24)	115,895	0.0095 (0.0014)	1.05E-10	40,626	0.0079 (0.0023)	3.16E-04	9.03E-04	156,521	0.0090 (0.0012)	8.72E-14	0	1.000	0.53
rs2290263<br>*MIR148A*	7	25,887,278	A/G(0.73)	132,930	0.0053 (0.0010)	2.35E-07	41,756	0.0016 (0.0019)	2.04E-01	5.15E-02	174,686	0.0045 (0.0009)	6.70E-07	5	0.959	1.00
rs3750082<br>*KBTBD2*	7	32,919,927	A/T(0.34)	127,284	0.0049 (0.0009)	2.52E-07	41,210	0.0031 (0.0018)	4.58E-02	1.96E-02	168,494	0.0045 (0.0008)	3.22E-08	2	0.934	1.00
rs1004402<br>*GRB10*	7	50,755,208	A/C(0.12)	127,292	0.0068 (0.0013)	4.79E-07	41,801	-0.0026 (0.0027)	8.31E-01	1.52E-01	169,093	0.0050 (0.0012)	2.93E-05	11	0.882	1.00
rs868055<br>*UBE2H*	7	129,435,191	T/C(0.10)	133,608	0.0082 (0.0016)	3.48E-07	41,843	-0.0008 (0.0029)	6.14E-01	1.22E-01	175,451	0.0060 (0.0014)	2.17E-05	22	0.981	0.83
rs6459680<br>*RNF32*	7	156,258,568	T/G(0.74)	133,692	-0.0065 (0.0010)	1.96E-10	41,655	-0.0019 (0.0019)	1.67E-01	4.62E-02	175,347	-0.0055 (0.0009)	1.07E-09	0	1.000	0.98
rs913423<br>*CYP26A1*	10	94,845,036	A/G(0.54)	132,861	-0.0052 (0.0009)	5.10E-09	41,638	-0.0014 (0.0017)	2.07E-01	5.15E-02	174,499	-0.0043 (0.0008)	7.65E-08	10	0.993	1.00
rs4014195<br>*AP5B1*	11	65,506,822	C/G(0.64)	133,723	0.0061 (0.0009)	2.19E-11	41,677	0.0034 (0.0018)	2.65E-02	1.42E-02	175,400	0.0055 (0.0008)	1.10E-11	0	1.000	1.00
rs10491967<br>*TSPAN9*	12	3,368,093	A/G(0.11)	133,800	-0.0092 (0.0014)	3.03E-10	41,870	-0.0106 (0.0027)	4.59E-05	3.93E-04	175,670	-0.0095 (0.0013)	5.18E-14	0	1.000	0.99
rs7956634<br>*PTPRO*	12	15,321,194	T/C(0.81)	133,722	-0.0068 (0.0011)	2.46E-09	41,726	-0.0069 (0.0022)	7.04E-04	1.51E-03	175,448	-0.0068 (0.0010)	7.17E-12	0	0.997	1.00

Supplementary Table 3 (continued).

**SNPID** Locus name*	**Chr**	**Position (bp)** (Build 37)	**Eff./ Non Eff All (EAF)**[#]	**STAGE 1 (discovery)**[†]			**STAGE 2 (replication)**[‡]				**Combined analysis**					
				**N**	**Beta (SE)**	**P-value**	**N**	**Beta (SE)**	**1-sided P-value**	**q-value**	**N**	**Beta (SE)**	**P-value**	**I^2 (%)**	**Power**	**Median Rsq**
**eGFRcrea in the overall sample**																
rs1106766 *INHBC*	12	57,809,456	T/C(0.22)	133,660	0.0062 (0.0011)	4.67E-08	21,005	0.0058 (0.0026)	1.23E-02	8.79E-03	154,665	0.0061 (0.0010)	2.41E-09	11	0.973	0.84
rs11180732 *PHLDA1*	12	76,283,354	T/G(0.31)	133,656	0.0049 (0.0009)	4.65E-07	41,586	0.0008 (0.0018)	3.26E-01	6.86E-02	175,242	0.0041 (0.0008)	6.23E-07	0	0.930	0.95
rs2071047 *BMP4*	14	54,418,411	A/G(0.41)	133,647	0.0044 (0.0009)	9.15E-07	41,772	0.0016 (0.0017)	1.85E-01	4.95E-02	175,419	0.0038 (0.0008)	3.20E-06	13	0.879	1.00
rs8056893 *SLC7A6*	16	68,304,392	A/C(0.72)	133,711	0.0051 (0.0010)	2.03E-07	41,695	0.0035 (0.0019)	3.21E-02	1.53E-02	175,406	0.0047 (0.0009)	1.28E-07	0	0.940	0.98
rs9807656 *SETBP1*	18	42,346,956	T/C(0.91)	133,688	-0.0081 (0.0016)	6.06E-07	40,684	0.0020 (0.0029)	7.61E-01	1.42E-01	174,372	-0.0056 (0.0014)	7.26E-05	22	0.947	0.99
rs9945268 *RNF152*	18	59,340,526	A/T(0.70)	127,290	-0.0048 (0.0010)	7.05E-07	41,612	-0.0020 (0.0018)	1.32E-01	4.05E-02	168,902	-0.0042 (0.0009)	3.18E-06	0	0.876	1.00
rs11666497 *SIPA1L3*	19	38,464,262	T/C(0.18)	127,271	-0.0064 (0.0012)	8.58E-08	41,640	-0.0041 (0.0022)	3.06E-02	1.53E-02	168,911	-0.0058 (0.0011)	4.25E-08	24	0.973	0.97
rs12975033 *IZUMO1*	19	49,249,443	A/T(0.55)	133,724	-0.0052 (0.0009)	4.75E-09	38,469	0.0006 (0.0017)	6.43E-01	1.22E-01	172,193	-0.0039 (0.0008)	1.31E-06	26	0.992	1.00
rs6088580 *TP53INP2*	20	33,285,053	C/G(0.47)	132,773	-0.0055 (0.0009)	7.17E-10	34,592	-0.0027 (0.0017)	6.28E-02	2.31E-02	167,365	-0.0049 (0.0008)	1.79E-09	0	0.997	0.99
rs2235808 *JPH2*	20	42,815,795	C/G(0.85)	129,605	0.0071 (0.0013)	1.50E-07	25,036	0.0020 (0.0025)	2.17E-01	5.17E-02	154,641	0.0060 (0.0012)	3.21E-07	0	0.967	0.87
rs17216707 *BCAS1*	20	52,732,362	T/C(0.79)	133,656	-0.0084 (0.0011)	5.96E-13	39,971	-0.0051 (0.0021)	7.80E-03	6.69E-03	173,627	-0.0077 (0.0010)	8.83E-15	1	1.000	0.85
**CKD in the overall sample**																
rs1032843 *FIGN*	2	164,184,329	T/C(0.07)	118,131	0.1508 (0.0299)	4.48E-07	32,338	0.0332 (0.0486)	2.47E-01	5.72E-02	150,469	0.1174 (0.0259)	5.70E-06	2	0.915	0.95
rs11039182 *MADD*	11	47,346,723	T/C(0.70)	118,130	-0.0818 (0.0167)	9.07E-07	35,062	-0.0325 (0.0271)	1.15E-01	3.66E-02	153,192	-0.0678 (0.0144)	2.64E-06	0	0.722	0.99
rs593790 *OR4C45*	11	48,391,116	A/T(0.82)	118,137	-0.1045 (0.0209)	5.96E-07	35,015	-0.0213 (0.0320)	2.53E-01	5.72E-02	153,152	-0.0788 (0.0178)	9.77E-06	0	0.956	0.99
rs2832559 *GRIK1*	21	31,417,144	T/C(0.91)	117,017	-0.1435 (0.0292)	9.56E-07	30,577	0.0668 (0.0540)	8.92E-01	1.61E-01	147,594	-0.0941 (0.0262)	3.31E-04	0	0.889	0.97

Supplementary Table 3 (continued).

**SNPID** Locus name*	**Chr**	**Position (bp)** (Build 37)	**Eff./ Non Eff All (EAF)**[#]	**STAGE 1 (discovery)**[†]			**STAGE 2 (replication)**[‡]				**Combined analysis**					
				**N**	**Beta (SE)**	**P-value**	**N**	**Beta (SE)**	**1-sided P-value**	**q-value**	**N**	**Beta (SE)**	**P-value**	**I^2 (%)**	**Power**	**Median Rsq**
**eGFRcys in the overall sample**																
rs12428035 *DZIP1*	13	96,300,872	T/C(0.12)	33,145	-0.0144 (0.0029)	7.28E-07	14,919	-0.0089 (0.0039)	1.13E-02	8.79E-03	48,064	-0.0125 (0.0023)	6.33E-08	0	0.588	1.00

*Based on Table 1 for replicated loci and on the closest gene name for the non-replicated loci, according to the UCSC Genome Browser on Human Feb. 2009 (GRCh37/hg19) Assembly.
#Effect Allele Frequency as estimated in the combined sample.
†All P-values and SEs reported from the STAGE 1 analysis were twice-GC corrected, to account for possible genomic inflation.
‡ In Stage 2 analysis, 1-sided P-values were computed to assess direction consistent effects with Stage 1; q-values were estimated based on the 1-sided P-values.

Supplementary Table 4. Association of replicated novel loci with eGFRcrea, CKD, and eGFRcys, in the overall combined sample.

					**eGFRcrea**			**CKD**		**eGFRcys**	
**SNP ID**	**Chr**	**Position (bp) (Build 37)**	**Locus name***	**Eff./ Non Eff. All. (EAF)**	**beta (SE)**	**P-value**	**beta (SE) from random-effect meta-analysis**	**OR (95%CI)**	**P-value**	**beta (SE)**	**P-value**
rs12136063	1	110,014,170	*SYPL2*	A/G(0.70)	0.0045(0.0008)	4.71E-08	0.0045(0.0008)	0.98(0.96,1.01)	2.37E-01	0.0025(0.0016)	1.25E-01
rs3850625	1	201,016,296	*CACNA1S*	A/G(0.12)	0.0078(0.0013)	5.53E-10	0.0079(0.0012)	0.95(0.91,0.99)	2.31E-02	0.0016(0.0023)	4.95E-01
rs2802729	1	243,501,763	*SDCCAG8*	A/C(0.43)	-0.0046(0.0008)	2.20E-08	-0.0047(0.0009)	1.05(1.02,1.08)	4.10E-04	-0.0036(0.0016)	2.49E-02
rs4667594	2	170,008,506	*LRP2*	A/T(0.53)	-0.0044(0.0008)	3.52E-08	-0.0045(0.0008)	1.05(1.02,1.07)	5.43E-04	0.0001(0.0015)	9.63E-01
rs2712184	2	217,682,779	*IGFBP5*	A/C(0.58)	-0.0048(0.0008)	3.02E-09	-0.0047(0.0009)	1.02(0.99,1.05)	1.54E-01	0.0001(0.0015)	9.39E-01
rs6795744	3	13,906,850	*WNT7A*	A/G(0.15)	0.0060(0.0011)	3.33E-08	0.0057(0.0013)	0.96(0.93,1.00)	3.98E-02	0.0049(0.0021)	1.84E-02
rs9682041	3	170,091,902	*SKIL*	T/C(0.87)	-0.0062(0.0012)	2.95E-07	-0.0062(0.0012)	1.02(0.98,1.06)	2.53E-01	-0.0039(0.0022)	7.99E-02
rs10513801	3	185,822,353	*ETV5*	T/G(0.87)	0.0070(0.0012)	2.47E-09	0.0069(0.0012)	0.93(0.89,0.96)	5.08E-05	0.0019(0.0023)	3.92E-01
rs228611	4	103,561,709	*NFKB1*	A/G(0.47)	-0.0056(0.0008)	3.58E-12	-0.0056(0.0008)	1.03(1.00,1.05)	3.36E-02	-0.0023(0.0015)	1.23E-01
rs7759001	6	27,341,409	*ZNF204*	A/G(0.76)	-0.0051(0.0009)	1.75E-08	-0.0052(0.0009)	1.03(1.00,1.06)	5.66E-02	-0.0023(0.0017)	1.71E-01
rs10277115	7	1,285,195	*UNCX*	A/T(0.23)	0.0090(0.0012)	8.72E-14	0.0090(0.0012)	0.96(0.92,1.00)	4.09E-02	0.0045(0.0025)	7.62E-02
rs3750082	7	32,919,927	*KBTBD2*	A/T(0.33)	0.0045(0.0008)	3.22E-08	0.0045(0.0009)	0.97(0.95,1.00)	5.76E-02	0.0014(0.0016)	3.95E-01
rs6459680	7	156,258,568	*RNF32*	T/G(0.74)	-0.0055(0.0009)	1.07E-09	-0.0055(0.0009)	1.04(1.01,1.07)	2.08E-02	-0.0043(0.0017)	1.06E-02
rs10994860	10	52,645,424	*A1CF*	T/C(0.19)	0.0071(0.0010)	1.66E-12	0.0069(0.0011)	0.99(0.95,1.02)	5.14E-01	0.0015(0.0020)	4.41E-01
rs163160	11	2,789,955	*KCNQ1*	A/G(0.82)	0.0064(0.0010)	1.72E-10	0.0063(0.0011)	0.95(0.92,0.99)	5.30E-03	0.0073(0.0020)	2.35E-04
rs4014195	11	65,506,822	*AP5B1*	C/G(0.64)	0.0055(0.0008)	1.10E-11	0.0056(0.0004)	0.93(0.91,0.96)	2.35E-07	0.0053(0.0015)	5.58E-04
rs10491967	12	3,368,093	*TSPAN9*	A/G(0.10)	-0.0095(0.0013)	5.18E-14	-0.0095(0.0013)	1.06(1.02,1.11)	6.54E-03	-0.0024(0.0024)	3.24E-01
rs7956634	12	15,321,194	*PTPRO*	T/C(0.81)	-0.0068(0.0010)	7.17E-12	-0.0066(0.0011)	1.03(1.00,1.06)	8.94E-02	-0.0059(0.0019)	2.17E-03
rs1106766	12	57,809,456	*INHBC*	T/C(0.22)	0.0061(0.0010)	2.41E-09	0.0060(0.0011)	0.98(0.95,1.02)	3.07E-01	0.0048(0.0019)	1.03E-02
rs164748	16	89,708,292	*DPEP1*	C/G(0.53)	0.0042(0.0008)	1.63E-07	0.0040(0.0010)	0.99(0.96,1.01)	3.27E-01	0.0046(0.0015)	1.80E-03
rs8091180	18	77,164,243	*NFATC1*	A/G(0.56)	-0.0053(0.0009)	5.57E-09	-0.0053(0.0009)	1.03(1.00,1.06)	3.57E-02	-0.0050(0.0020)	1.19E-02
rs11666497	19	38,464,262	*SIPA1L3*	T/C(0.18)	-0.0058(0.0011)	4.25E-08	-0.0061(0.0013)	1.00(0.97,1.04)	9.70E-01	-0.0027(0.0019)	1.70E-01
rs6088580	20	33,285,053	*TP53INP2*	C/G(0.47)	-0.0049(0.0008)	1.79E-09	-0.0049(0.0008)	1.04(1.01,1.07)	5.25E-03	0.0000(0.0015)	9.92E-01
rs17216707	20	52,732,362	*BCAS1*	T/C(0.79)	-0.0077(0.0010)	8.83E-15	-0.0076(0.0011)	1.05(1.01,1.09)	8.18E-03	-0.0083(0.0020)	3.25E-05

* Conventional locus name based on relevant genes in the region as identified by bioinformatic investigation (Suppl. Tab. 12) or closest gene. A complete overview of the genes in each locus is given in the regional association plots (Suppl. Fig. 4)

Supplementary Table 5. SNP associations for novel and known loci stratified by diabetes status**.**

SNP ID (effect allele)	Locus name	No Diabetes			Diabetes			P-value for difference*
		N	beta (SE)	P-value	N	beta (SE)	P-value	
**Novel loci**: sample size based on the combined (discovery and replication) sample								
rs3850625 (A)	*CACNA1S*	153,107	0.0083 (0.0013)	6.82E-11	16,275	0.0038 (0.0048)	4.34E-01	0.27
rs2712184 (A)	*IGFBP5*	153,854	-0.0053 (0.0004)	1.33E-10	16,463	-0.0034 (0.0031)	2.70E-01	0.22
rs9682041 (T)	*SKIL*	150,911	-0.0068 (0.0012)	2.58E-08	16,161	0.0006 (0.0045)	8.86E-01	0.20
rs10513801 (T)	*ETV5*	154,774	0.0072 (0.0012)	1.03E-09	16,470	0.0022 (0.0045)	6.19E-01	0.28
rs10994860 (T)	*A1CF*	154,644	0.0077 (0.0011)	1.07E-12	16,451	0.0031 (0.0041)	4.57E-01	0.27
rs163160 (A)	*KCNQ1*	154,684	0.0065 (0.0011)	2.26E-09	16,457	0.0055 (0.0040)	1.68E-01	0.81
rs164748 (C)	*DPEP1*	154,497	0.0046 (0.0004)	1.95E-08	16,416	0.0026 (0.0031)	4.07E-01	0.52
rs8091180 (A)	*NFATC1*	153,715	-0.0060 (0.0010)	1.28E-09	13,764	0.0030 (0.0043)	4.85E-01	0.04
rs12136063 (A)	*SYPL2*	152,998	0.0043 (0.0009)	2.25E-06	16,253	0.0013 (0.0034)	7.02E-01	0.38
rs2802729 (A)	*SDCCAG8*	152,501	-0.0042 (0.0009)	2.65E-06	16,156	-0.0050 (0.0033)	1.24E-01	0.81
rs4667594 (A)	*LRP2*	154,570	-0.0042 (0.0008)	2.09E-07	16,449	-0.0034 (0.0031)	2.67E-01	0.80
rs6795744 (A)	*WNT7A*	154,716	0.0053 (0.0011)	1.23E-06	16,462	0.0070 (0.0043)	1.02E-01	0.70
rs228611 (A)	*NFKB1*	154,683	-0.0056 (0.0008)	7.39E-12	16,454	-0.0077 (0.0030)	1.07E-02	0.49
rs7759001 (A)	*ZNF204*	154,713	-0.0053 (0.0009)	1.02E-08	16,458	-0.0000 (0.0036)	9.92E-01	0.15
rs10277115 (A)	*UNCX*	141,932	0.0085 (0.0013)	4.97E-11	13,565	0.0156 (0.0053)	3.30E-03	0.19
rs3750082 (A)	*KBTBD2*	148,429	0.0042 (0.0009)	3.82E-06	15,754	0.0050 (0.0033)	1.32E-01	0.81
rs6459680 (T)	*RNF32*	154,586	-0.0057 (0.0009)	4.64E-10	16,454	-0.0043 (0.0035)	2.25E-01	0.70
rs4014195 (C)	*AP5B1*	154,634	0.0050 (0.0008)	1.02E-09	16,452	0.0116 (0.0032)	2.52E-04	0.04
rs10491967 (A)	*TSPAN9*	154,881	-0.0089 (0.0013)	3.18E-11	16,477	-0.0204 (0.0050)	3.80E-05	0.02
rs7956634 (T)	*PTPRO*	154,677	-0.0065 (0.0010)	1.11E-10	16,460	-0.0076 (0.0038)	4.82E-02	0.78
rs1106766 (T)	*INHBC*	138,058	0.0063 (0.0010)	1.75E-09	14,990	0.0016 (0.0040)	6.89E-01	0.25
rs11666497 (T)	*SIPA1L3*	148,770	-0.0058 (0.0011)	6.04E-08	15,831	-0.0096 (0.0041)	2.01E-02	0.37
rs6088580 (C)	*TP53INP2*	150,326	-0.0050 (0.0008)	1.51E-09	15,387	-0.0010 (0.0031)	7.48E-01	0.21
rs17216707 (T)	*BCAS1*	153,362	-0.0075 (0.0011)	2.25E-12	15,977	-0.0063 (0.0039)	1.06E-01	0.76
**Known loci:** sample size based on the discovery sample								
rs12917707 (T)	*UMOD*	118,365	0.0152 (0.0012)	4.68E-36	11,522	0.0266 (0.0048)	2.48E-08	0.02
rs1260326 (T)	*GCKR*	118,415	0.0065 (0.0009)	1.86E-12	11,525	0.0112 (0.0037)	2.21E-03	0.21
rs6465825 (T)	*SLC22A2*	118,448	0.0061 (0.0009)	4.80E-11	11,529	0.0089 (0.0036)	1.44E-02	0.45
rs2279463 (A)	*VEGFA*	118,368	0.0119 (0.0014)	6.98E-18	11,521	0.0062 (0.0053)	2.43E-01	0.30
rs12124078 (A)	*CASP9*	116,718	0.0058 (0.0010)	4.54E-09	11,315	0.0039 (0.0039)	3.18E-01	0.63
rs848490 (C)	*TMEM60*	112,566	0.0074 (0.0010)	1.35E-12	10,897	0.0033 (0.0041)	4.30E-01	0.33
rs2928148 (A)	*INO80*	118,368	0.0050 (0.0009)	2.82E-08	11,521	0.0026 (0.0035)	4.73E-01	0.50
rs9895661 (T)	*BCAS3*	118,188	0.0121 (0.0012)	2.80E-22	11,520	-0.0006 (0.0048)	9.00E-01	0.01
rs347685 (A)	*TFDP2*	118,374	-0.0077 (0.0010)	1.70E-14	11,522	-0.0021 (0.0040)	5.93E-01	0.17
rs7208487 (T)	*CDK12*	118,453	-0.0091 (0.0012)	8.60E-14	11,526	-0.0024 (0.0048)	6.20E-01	0.17

Supplementary Table 5 (continued).

SNP ID (effect allele)	Locus name	No Diabetes			Diabetes			P-value for difference*
		N	beta (SE)	P-value	N	beta (SE)	P-value	
**Known loci:** sample size based on the discovery sample								
rs10774021 (T)	*SLC6A13*	118,448	-0.0067 (0.0009)	1.48E-12	11,526	-0.0031 (0.0038)	4.13E-01	0.35
rs2453580 (T)	*SLC47A1*	117,386	0.0073 (0.0010)	2.56E-13	11,474	-0.0035 (0.0040)	3.93E-01	0.01
rs4744712 (A)	*PIP5K1B*	118,374	-0.0072 (0.0009)	7.45E-15	11,518	-0.0053 (0.0037)	1.48E-01	0.62
rs12460876 (T)	*SLC7A9*	118,361	-0.0065 (0.0009)	2.86E-12	11,521	-0.0062 (0.0037)	9.16E-02	0.94
rs881858 (A)	*SLC34A1*	118,324	-0.0081 (0.0011)	4.03E-14	11,521	-0.0063 (0.0042)	1.35E-01	0.68
rs10109414 (T)	*STC1*	118,342	-0.0075 (0.0009)	3.53E-16	11,521	-0.0066 (0.0036)	6.75E-02	0.81
rs6431731 (T)	*DDX1*	109,308	-0.0124 (0.0025)	4.70E-07	10,419	-0.0070 (0.0100)	4.89E-01	0.60
rs626277 (A)	*DACH1*	118,450	-0.0049 (0.0009)	1.44E-07	11,528	-0.0072 (0.0036)	4.53E-02	0.53
rs17319721 (A)	*SHROOM3*	118,359	-0.0113 (0.0009)	5.08E-35	11,522	-0.0075 (0.0036)	3.64E-02	0.30
rs13538 (A)	*ALMS1*	115,154	-0.0092 (0.0011)	2.79E-16	11,135	-0.0077 (0.0045)	8.45E-02	0.74
rs11959928 (A)	*DAB2*	118,359	-0.0082 (0.0009)	7.14E-19	11,521	-0.0078 (0.0036)	3.33E-02	0.91
rs2453533 (A)	*GATM*	118,374	-0.0125 (0.0009)	4.83E-41	11,520	-0.0079 (0.0036)	3.16E-02	0.21
rs7805747 (A)	*PRKAG2*	116,602	-0.0136 (0.0012)	1.53E-29	11,378	-0.0085 (0.0048)	7.37E-02	0.30
rs1394125 (A)	*UBE2Q2*	118,404	-0.0072 (0.0010)	5.83E-13	11,525	-0.0089 (0.0041)	2.92E-02	0.69
rs3925584 (T)	*MPPED2*	118,345	-0.0074 (0.0009)	5.35E-16	11,521	-0.0100 (0.0035)	5.00E-03	0.47
rs267734 (T)	*LASS2*	116,718	-0.0079 (0.0011)	2.81E-12	11,315	-0.0110 (0.0045)	1.39E-02	0.50
rs7422339 (A)	*CPS1*	115,499	-0.0106 (0.0011)	3.25E-22	11,422	-0.0137 (0.0043)	1.61E-03	0.48
rs10794720 (T)	*WDR37*	118,433	-0.0093 (0.0017)	4.95E-08	11,528	-0.0145 (0.0067)	3.06E-02	0.45
rs491567 (A)	*WDR72*	118,374	-0.0078 (0.0011)	1.45E-12	11,521	-0.0167 (0.0043)	1.25E-04	0.04

* P-value of a two-sample *t* test for correlated data (see Methods: "Associations Stratified by Diabetes and Hypertension Status").

Supplementary Table 6. Association between replicated SNPs and additional kidney related phenotypes.*

			Myocardial Infarction		Left Ventricular Mass		Incident Heart Failure		DBP		SBP		UACR		Fasting glucose	
SNPID	Locus name	All. #	OR (95%CI)	P-value	beta (SE)	P-value	beta (SE)	P-value	beta (SE)	P-value	beta (SE)	P-value	beta(SE)	P-value	beta (SE)	P-value
rs3850625	*CACNA1S*	A/G	1.03 (0.99,1.08)	0.15	0.390 (0.680)	0.57	-0.034 (0.044)	0.44	0.003 (0.094)	0.97	-0.068 (0.147)	0.64	0.017 (0.011)	0.11	0.012 (0.005)	0.013
rs2712184	*IGFBP5*	A/C	1.00 (0.97,1.03)	0.81	-0.183 (0.449)	0.68	-0.051 (0.034)	0.14	-0.087 (0.062)	0.16	-0.016 (0.098)	0.87	-0.001 (0.006)	0.88	-0.001 (0.003)	0.77
rs9682041	*SKIL*	T/C	0.99 (0.95,1.04)	0.79	-0.036 (0.639)	0.96	-0.003 (0.043)	0.95	-0.026 (0.093)	0.78	-0.092 (0.147)	0.53	-0.015 (0.009)	0.11	-0.006 (0.005)	0.18
rs10513801	*ETV5*	T/G	0.97 (0.94,1.01)	0.18	0.255 (0.649)	0.69	0.019 (0.044)	0.67	-0.021 (0.091)	0.82	0.004 (0.145)	0.98	0.027 (0.011)	0.014	0.002 (0.005)	0.71
rs10994860	*A1CF*	T/C	0.99 (0.96,1.03)	0.56	0.294 (0.595)	0.62	-0.081 (0.041)	0.05	-0.064 (0.081)	0.43	-0.162 (0.127)	0.20	-0.006 (0.009)	0.55	0.004 (0.004)	0.28
rs163160	*KCNQ1*	A/G	0.98 (0.95,1.02)	0.39	0.360 (0.577)	0.53	-0.063 (0.038)	0.10	-0.040 (0.082)	0.62	0.032 (0.129)	0.80	0.011 (0.010)	0.26	0.001 (0.004)	0.86
rs164748	*DPEP1*	C/G	0.99 (0.97,1.02)	0.65	0.668 (0.439)	0.13	0.062 (0.029)	0.04	0.173 (0.063)	0.01	0.361 (0.099)	0.00026[§]	0.021 (0.006)	0.0004	-0.003 (0.003)	0.30
rs8091180	*NFATC1*	A/G	NA	NA	0.307 (0.816)	0.71	0.056 (0.044)	0.21	0.122 (0.103)	0.23	0.181 (0.162)	0.27	-0.018 (0.015)	0.21	0.006 (0.005)	0.19
rs12136063	*SYPL2*	A/G	1.03 (1.00,1.06)	0.08	-0.192 (0.478)	0.69	0.023 (0.032)	0.47	0.032 (0.066)	0.62	-0.033 (0.104)	0.75	0.010 (0.008)	0.19	-0.007 (0.003)	0.05
rs2802729	*SDCCAG8*	A/C	NA	NA	-0.127 (0.470)	0.79	-0.005 (0.032)	0.88	0.147 (0.067)	0.03	0.239 (0.105)	0.02	0.008 (0.007)	0.23	0.002 (0.003)	0.53
rs4667594	*LRP2*	A/T	0.99 (0.97,1.02)	0.59	0.940 (0.440)	0.03	0.019 (0.029)	0.51	0.098 (0.062)	0.11	0.152 (0.097)	0.12	-0.008 (0.006)	0.18	0.000 (0.003)	0.89
rs6795744	*WNT7A*	A/G	0.98 (0.94,1.02)	0.27	0.298 (0.643)	0.64	-0.071 (0.045)	0.12	-0.022 (0.088)	0.80	-0.110 (0.139)	0.43	-0.005 (0.009)	0.57	0.005 (0.004)	0.30
rs228611	*NFKB1*	A/G	0.98 (0.95,1.01)	0.18	0.165 (0.440)	0.71	0.008 (0.028)	0.78	0.090 (0.061)	0.14	0.003 (0.097)	0.98	0.008 (0.006)	0.20	0.011 (0.003)	0.00026[§]
rs7759001	*ZNF204*	A/G	0.99 (0.96,1.02)	0.51	0.172 (0.524)	0.74	-0.031 (0.034)	0.36	-0.022 (0.071)	0.75	-0.085 (0.112)	0.45	0.002 (0.007)	0.83	0.006 (0.004)	0.10
rs10277115	*UNCX*	A/T	NA	NA	-1.058 (0.777)	0.17	0.012 (0.052)	0.82	-0.099 (0.141)	0.48	-0.232 (0.219)	0.29	0.013 (0.012)	0.27	-0.009 (0.006)	0.15
rs3750082	*KBTBD2*	A/T	0.99 (0.96,1.02)	0.48	0.641 (0.470)	0.17	-0.005 (0.031)	0.86	-0.024 (0.066)	0.72	0.094 (0.104)	0.37	0.019 (0.006)	0.0036	-0.001 (0.003)	0.88
rs6459680	*RNF32*	T/G	0.98 (0.95,1.01)	0.14	-0.234 (0.514)	0.65	-0.010 (0.034)	0.76	-0.155 (0.071)	0.03	-0.184 (0.112)	0.10	-0.001 (0.008)	0.90	0.002 (0.004)	0.60
rs4014195	*AP5B1*	C/G	0.98 (0.95,1.01)	0.20	-0.825 (0.458)	0.07	-0.052 (0.035)	0.14	-0.079 (0.064)	0.22	-0.176 (0.101)	0.08	0.007 (0.006)	0.29	0.001 (0.003)	0.70

Supplementary Table 6 (continued).

			Myocardial Infarction		Left Ventricular Mass		Incident Heart Failure		DBP		SBP		UACR		Fasting glucose	
**SNPID**	**Locus name**	**All.** #	**OR (95%CI)**	**P-value**	**beta (SE)**	**P-value**	**beta (SE)**	**P-value**	**beta (SE)**	**P-value**	**beta (SE)**	**P-value**	**beta(SE)**	**P-value**	**beta (SE)**	**P-value**
rs10491967	*TSPAN9*	A/G	0.95 (0.91,1.00)	0.04	0.087 (0.696)	0.90	-0.033 (0.047)	0.48	0.003 (0.101)	0.98	-0.205 (0.160)	0.20	0.002 (0.011)	0.86	-0.003 (0.005)	0.60
rs7956634	*PTPRO*	T/C	1.01 (0.98,1.05)	0.49	0.528 (0.564)	0.35	0.010 (0.036)	0.79	0.047 (0.083)	0.57	0.113 (0.130)	0.39	-0.017 (0.008)	0.03	0.006 (0.004)	0.17
rs1106766	*INHBC*	T/C	0.99 (0.96,1.02)	0.56	-0.322 (0.584)	0.58	0.027 (0.037)	0.46	0.069 (0.079)	0.39	0.093 (0.125)	0.46	0.011 (0.009)	0.22	-0.001 (0.004)	0.82
rs11666497	*SIPA1L3*	T/C	1.02 (0.98,1.05)	0.42	0.624 (0.590)	0.29	0.058 (0.041)	0.15	-0.056 (0.081)	0.49	-0.034 (0.127)	0.79	0.000 (0.008)	1.00	0.002 (0.004)	0.72
rs6088580	*TP53INP2*	C/G	1.02 (0.99,1.05)	0.14	-0.864 (0.456)	0.06	0.014 (0.028)	0.63	-0.126 (0.061)	0.04	-0.066 (0.097)	0.49	0.010 (0.006)	0.10	0.010 (0.003)	0.0016
rs17216707	*BCAS1*	T/C	1.02 (0.98,1.05)	0.40	0.238 (0.605)	0.69	0.038 (0.041)	0.35	0.136 (0.084)	0.11	0.195 (0.132)	0.14	-0.000 (0.009)	0.98	0.004 (0.004)	0.36

* ***Myocardial infarction***: results from CARDIoGRAM meta-analysis[8] of up to 14 studies, for a total sample size comprised between 72,649 and 83,231, except for SNPs rs4014195 (N=61,259) and rs2712184 (N=61,275). SNPs not reported did not pass CARDIoGRAM internal quality control checks and were not assessed. ***Left Ventricular Mass*:** per-allele effect on left ventricular mass in grams, from a meta-analysis of five community-based studies within the EchoGen consortium, totaling 12,612 European ancestry individuals.[9] ***Incident heart failure***: CHARGE consortium meta-analysis on incident heart failure:[10] four studies on European ancestry individuals totaling a sample size of 20,926, except for SNPs rs2712184 and rs4014195 (N=13,282). ***DBP*** and ***SBP***: diastolic and systolic blood pressure results from the ICBP consortium:[11] meta-analysis of up to 43 studies, for a total sample size of 53,302 to 69,671 samples, except for SNPs rs10277115 (27 studies; N=17,644) and rs8091180 (21 studies; N=23,456). ***UACR***: urinary albumin-to-creatinine ratio, results from the CKDGen consortium (personal communication): meta-analysis of up to 29 studies, for a total sample size of 39,130 to 54,450. ***Fasting glucose***: per-allele effect on fasting glucose (mmol/L) not adjusted for BMI in 29 studies on up 58,074 non-diabetic participants of European ancestry.[12]

# All.: first is the effect and second is the non-effect allele, respectively.

§ P-values that are significant at a Bonferroni corrected level of 0.0003, corresponding to 0.05 over 165 tests.

Supplementary Table 7. NHGRI GWAS catalog query of novel loci associated with different traits at a genome-wide significant level.

Locus name	Index SNP	Published SNP	Chr	Position (Build 37)	Region	LD (r^2)*	Disease or Quantitative Trait[REF]	P-value
SDCCAG8	rs2802729	rs6703335	1	243,608,967	1q43	0.44	Schizophrenia[13]	5.00E-08
ETV5	rs10513801	rs1516725	3	185,824,004	3q27.2	1.00	Body mass index[14]	4.00E-08
		rs1516725	3	185,824,004	3q27.2	1.00	Obesity[14]	3.00E-09
		rs7647305	3	185,834,290	3q27.2	0.39	Body mass index[15]	7.00E-11
		rs7647305	3	185,834,290	3q27.2	0.39	Weight[15]	4.00E-09
		rs9816226	3	185,834,499	3q27.2	0.49	Obesity[14]	2.00E-13
		rs9816226	3	185,834,499	3q27.2	0.49	Obesity[14]	2.00E-14
		rs9816226	3	185,834,499	3q27.2	0.49	Body mass index[16]	2.00E-18
NFKB1	rs228611	rs3774959	4	103,511,114	4q24	0.29	Ulcerative colitis[17]	4.00E-12
		rs7665090	4	103,551,603	4q24	0.87	Primary biliary cirrhosis[18]	4.00E-12
UNCX	rs10277115	rs10275044	7	1,273,845	7p22.3	0.73	Blood Urea Nitrogen[19]	4.00E-09
		rs10277115	7	1,285,195	7p22.3	1.00	eGRFcrea[19]	1.00E-10
		rs10277115	7	1,285,195	7p22.3	1.00	Serum creatinine[19]	5.00E-11
A1CF	rs10994860	rs10821905	10	52,646,093	10q11.23	1.00	Urate levels[20]	7.00E-17
AP5B1	rs4014195	rs479844	11	65,551,957	11q13.1	0.37	Atopic dermatitis[21]	1.00E-13
		rs642803	11	65,560,620	11q13.1	0.40	Urate levels[20]	3.00E-13
INHBC	rs1106766	rs11613352	12	57,792,580	12q13.3	1.00	HDL cholesterol[22]	2.00E-08
		rs11613352	12	57,792,580	12q13.3	1.00	Triglycerides[22]	4.00E-10
		rs1106766	12	57,809,456	12q13.3	1.00	Urate levels[23]	2.00E-11
		rs3741414	12	57,844,049	12q13.3	0.97	Urate levels[20]	2.00E-25
DPEP1	rs164748	rs154657	16	89,708,096	16q24.3	1.00	Homocysteine levels[24]	2.00E-43
		rs258322	16	89,755,903	16q24.3	0.30	Melanoma[25]	3.00E-27
		rs258322	16	89,755,903	16q24.3	0.30	Melanoma[26]	3.00E-27
		rs258322	16	89,755,903	16q24.3	0.30	Black vs. red hair color[27]	2.00E-23
		rs12921383	16	89,859,753	16q24.3	0.25	Homocysteine levels[24]	8.00E-11
		rs1805007	16	89,986,117	16q24.3	0.25	Hair color[28]	3.00E-09
		rs1805007	16	89,986,117	16q24.3	0.25	Non-melanoma skin cancer[28]	3.00E-10
		rs1805007	16	89,986,117	16q24.3	0.25	Sunburns[28]	2.00E-19
		rs1805007	16	89,986,117	16q24.3	0.25	Tanning[28]	1.00E-65
		rs1805007	16	89,986,117	16q24.3	0.25	Basal cell carcinoma[29]	4.00E-17
		rs1805007	16	89,986,117	16q24.3	0.25	Blond vs. brown hair color[30]	2.00E-13
		rs1805007	16	89,986,117	16q24.3	0.25	Freckles[30]	1.00E-96
		rs1805007	16	89,986,117	16q24.3	0.25	Red vs non-red hair color[30]	2.00E-142
		rs1805007	16	89,986,117	16q24.3	0.25	Skin sensitivity to sun[30]	2.00E-55
TP53INP2	rs6088580	rs2284378	20	32,588,095	20q11.22	0.25	Breast cancer[31]	1.00E-08
		rs4911414	20	32,729,444	20q11.22	0.25	Tanning[28]	4.00E-09
		rs4911414	20	32,729,444	20q11.22	0.25	Burning and freckling[32]	6.00E-37
		rs4911414	20	32,729,444	20q11.22	0.25	Freckles[32]	8.00E-29
		rs4911414	20	32,729,444	20q11.22	0.25	Red vs. non-red hair color[32]	3.00E-09
		rs4911414	20	32,729,444	20q11.22	0.25	Skin sensitivity to sun[32]	2.00E-24
		rs910873	20	33,171,772	20q11.22	0.21	Melanoma[33]	1.00E-15
		rs8114671	20	33,789,142	20q11.22	0.47	Height[14]	1.00E-15
		rs6088765	20	33,799,280	20q11.22	0.34	Ulcerative colitis[17]	2.00E-08

* LD between published and index SNPs**.**

Supplementary Table 8. Study sample characteristics, African ancestry meta-analysis**.**

Study	Sample Size	European Ancestry % - change to median with 25/75th percentiles	Women %	Mean age (years)	Mean eGFRcrea (ml/min/1.73 m^2)	CKD %
ARIC	2786	15.3 (10.7, 22.1)	63.1	53.3	100	3.7
CARDIA	821	16.7 (12.2, 23.2)	61.1	39.4	111	0.9
CHS	728	20.6 (12.4, 32.7)	62.8	72.9	81	18.4
JHS	2135	15.7 (11.8, 21.1)	60.8	50	101	4.2
MESA	1640	18.8 (11.5, 29.7)	54.8	62.2	87	8.6
GENOA	1217	12.6 (7.2, 18.9)	71.7	63.2	88	13.0
HANDLS	989	16.1 (11.2, 22.0)	55.0	48.4	121	5.3
Health ABC	1139	22.4 (12.2, 32.6)	57.2	73.4	76	17.1
HUFS	1013	19.7 (14.3, 27.0)	58.8	48.3	104	4.9
IPM	712	12.5 (7.1, 19.5)	60.0	59.7	76	36.4
SIGNET-Sea Islands	1275	7.0 (4.3, 11.7)	77.1	53.7	106	9.6
SIGNET-REGARDS	2385	14.8 (9.0, 22.9)	63.7	63	100	10.0

Abbreviations: eGFRcrea: estimated glomerular filtration rate by serum creatinine, CKD: chronic kidney disease.

Supplementary Table 9. Evaluation of replicated SNPs among individuals of Asian (AGEN) and the African Ancestry Renal meta-analysis**.**

Locus name	SNPID	Eff. All.	Non Eff. All.	AGEN Consortium[19]				African Ancestry Renal Meta-Analysis			
				N	Eff. All. Freq.	Beta (SE)	P-value	N	Ref. all freq.	Beta (SE)	P-value
SYPL2	rs12136063	A	G	36,057	0.90	-0.0004 (0.0021)	0.85	16,349	0.32	0.0049 (0.0036)	0.16
CACNA1S	rs3850625	A	G	41,963	0.09	-0.0012 (0.0019)	0.53	11,194	0.02	0.0127 (0.0135)	0.35
SDCCAG8	rs2802729	A	C	42,296	0.29	-0.0007 (0.0009)	0.42	15,461	0.47	-0.0007 (0.0036)	0.85
LRP2	rs4667594	A	T	40,415	0.13	-0.0015 (0.0013)	0.24	15,417	0.61	-0.0016 (0.0034)	0.63
IGFBP5	rs2712184	A	C	40,415	0.51	-0.0025 (0.0008)	0.0024	16,210	0.48	-0.0023 (0.0034)	0.50
WNT7A	rs6795744	A	G	42,296	0.11	-0.0001 (0.0016)	0.94	16,420	0.21	0.0032 (0.0043)	0.46
SKIL	rs9682041	T	C	42,296	0.91	-0.0047 (0.0018)	0.0074	15,460	0.75	-0.0005 (0.0041)	0.91
ETV5	rs10513801	T	G	37,250	0.93	0.0006 (0.0026)	0.82	14,243	0.95	-0.0006 (0.0100)	0.95
NFKB1	rs228611	A	G	42,296	0.52	-0.0001 (0.0008)	0.88	16,450	0.24	0.0042 (0.0040)	0.29
ZNF204	rs7759001	A	G	40,415	0.62	-0.0005 (0.0008)	0.56	16,435	0.87	0.0044 (0.0050)	0.38
UNCX	rs10277115	A	T	40,415	0.65	0.0066 (0.0011)	7.30E-10	11,801	0.70	0.0059 (0.0060)	0.32
KBTBD2	rs3750082	A	T	39,440	0.32	0.0012 (0.0009)	0.19	15,461	0.67	0.0070 (0.0039)	0.07
RNF32	rs6459680	T	G	40,415	0.62	-0.0005 (0.0009)	0.56	16,435	0.54	0.0049 (0.0034)	0.15
A1CF	rs10994860	T	C	41,963	0.10	0.0014 (0.0017)	0.42	15,461	0.22	0.0060 (0.0045)	0.18
KCNQ1	rs163160	A	G	40,415	0.83	0.0022 (0.0011)	0.04	16,467	0.93	0.0053 (0.0067)	0.43
AP5B1	rs4014195	G	C	40,415	0.20	-0.0040 (0.0010)	0.00012	15,297	0.22	-0.0014 (0.0042)	0.74
TSPAN9	rs10491967	A	G	40,415	0.45	-0.0013 (0.0008)	0.13	15,460	0.40	0.0072 (0.0043)	0.09
PTPRO	rs7956634	T	C	40,415	0.68	-0.0020 (0.0009)	0.03	16,403	0.50	-0.0082 (0.0033)	0.01
INHBC	rs1106766	T	C	42,296	0.11	0.0003 (0.0015)	0.85	16,415	0.10	-0.0008 (0.0057)	0.89
DPEP1	rs164748	G	C	39,164	0.08	0.0016 (0.0024)	0.49	16,471	0.11	0.0025 (0.0055)	0.65
NFATC1	rs8091180	A	G	40,415	0.83	-0.0004 (0.0011)	0.69	3,838	0.20	-0.0021 (0.0101)	0.84
SIPA1L3	rs11666497	T	C	40,415	0.12	-0.0027 (0.0013)	0.03	16,422	0.07	0.0023 (0.0064)	0.72
TP53INP2	rs6088580	G	C	36,275	0.64	0.0007 (0.0009)	0.45	NA	NA	NA	NA
BCAS1	rs17216707	T	C	35,492	0.86	-0.0012 (0.0018)	0.52	15,399	0.94	-0.0049 (0.0073)	0.50

Supplementary Table 10. Transethnic meta-analysis of CKDGen and the African Ancestry Renal Meta-Analysis**.***

SNPID	Locus name	Chr.	Effect Allele	Non-Effect Allele	log10BF#	Posterior Probability
rs17216707	*BCAS1*	20	T	C	11.63	0.038
rs4014195	*AP5B1*	11	C	G	9.66	0.025
rs10994860	*A1CF*	10	T	C	9.52	0.025
rs7956634	*PTPRO*	12	T	C	9.07	0.015
rs10277115	*UNCX*	7	A	T	8.84	0.023
rs228611	*NFKB1*	4	A	G	7.65	0.07
rs6088580	*TP53INP2*	20	C	G	7.44	0.021
rs163160	*KCNQ1*	11	A	G	7.38	0.026
rs3850625	*CACNA1S*	1	A	G	7.34	0.046
rs6795744	*WNT7A*	3	A	G	7.04	0.046
rs10491967	*TSPAN9*	12	A	G	6.93	0.725
rs2712184	*IGFBP5*	2	A	C	6.72	0.021
rs3750082	*KBTBD2*	7	A	T	6.65	0.01
rs6459680	*RNF32*	7	T	G	6.42	0.701
rs12136063	*SYPL2*	1	A	G	6.18	0.012
rs2802729	*SDCCAG8*	1	A	C	6.00	0.021
rs10513801	*ETV5*	3	T	G	5.88	0.03
rs164748	*DPEP1*	16	C	G	5.83	0.045
rs4667594	*LRP2*	2	A	T	5.72	0.027
rs11666497	*SIPA1L3*	19	T	C	5.72	0.031
rs1106766	*INHBC*	12	T	C	5.56	0.053
rs7759001	*ZNF204*	6	A	G	5.23	0.051
rs9682041	*SKIL*	3	T	C	5.04	0.032
rs8091180	*NFATC1*	18	A	G	4.87	0.044

*The analysis was performed using the MANTRA (Meta-Analysis of Trans-ethnic Association studies) software.[34] The posterior probability is a measure of heterogeneity of allelic effects across the individual studies.

#Log_{10} Bayes Factor

Supplementary Table 11. SNP associations with transcript expression.*

Locus name; index SNP	*eSNP*									*Best eSNP*			
	eSNP rsID	*Dist. index SNP*	*r^2*	*eQTL tissue[REF]*	*P-value*	*Chr*	*position*	*Probe**	*Transcript***	*Best eSNP rsID*	*P-value*	*r^2 to eSNP*	*r^2 to index SNP*
SYPL2 rs12136063	rs12136063	0	1.00	Prefrontal cortex - all samples[35]	1.3E-05	1	109,815,693	10031920561	SYPL2	rs12136063	1.3E-05	Same	Same
	rs10494040	1,562	1.00	SubCutAdipose[36]	6.5E-50	1	109,817,255	10031920561	SYPL2	rs10494040	6.5E-50	Same	0.96
	rs10494040	1,562	1.00	Liver[36]	5.4E-47	1	109,817,255	10031920561	SYPL2	rs10494040	5.4E-47	Same	0.96
	rs10857787	3,881	1.00	Liver[37]	2.9E-35	1	109,811,812		SYPL2	rs10857787	2.9E-35	Same	0.96
	rs4970767	1,013	1.00	Liver (UChicago)[38]	8.9E-16	1	109,816,706	A_23_P317200	ATXN7L2	rs4970767	8.9E-16	Same	0.96
	rs10494040	1,562	1.00	OmentalAdipose[36]	9.4E-13	1	109,817,255	10031920561	SYPL2	rs10494040	9.4E-13	Same	0.96
	rs10494040	1,562	1.00	Liver[39]	1.1E-12	1	109,817,255		SYPL2	rs10494040	1.1E-12	Same	0.96
	rs4970767	1,013	1.00	Liver (UWash)[38]	3.3E-02	1	109,816,706	5360451	ATXN7L2	rs4970767	3.3E-02	Same	0.96
	rs4970729	34,968	0.92	Liver[36]	2.3E-24	1	109,780,725	10025910902	PSMA5	rs4970729	2.3E-24	Same	0.89
	rs12073497	39,720	0.92	Liver[36]	6.2E-09	1	109,775,973	10023805980	Contig42599 _RC	rs12073497	6.2E-09	Same	0.89
	rs12073497	39,720	0.92	Visual cortex - all samples[35]	3.1E-06	1	109,775,973	10031920561	SYPL2	rs12073497	3.1E-06	Same	0.89
	rs4970729	34,968	0.92	Cerebellum - all samples[35]	1.1E-05	1	109,780,725	10025932473	AMIGO1	rs4970729	1.1E-05	Same	0.89
	rs2781553	12,819	0.85	Liver[36]	4.8E-05	1	109,828,512	10025909878	PRPF38B	rs2781553	4.8E-05	Same	0.85
SDCCAG8 rs2802729	rs2802723	3,451	1.00	Periph artery plaque‡	4.1E-07	1	241,564,935	100142973_TGI _at	SDCCAG8	rs2802723	4.1E-07	Same	1.00
	rs2490395	42,841	0.83	Blood[40]	9.2E-06	1	241,525,545	460458	SDCCAG8	rs2490395	9.2E-06	Same	0.83
	rs2484639	39,396	0.81	Visual cortex - all samples[35]	3.2E-07	1	241,528,990	10025912019	SDCCAG8	rs2484639	3.2E-07	Same	0.80
NFKB1 rs228611	rs228611	0	1.00	LCL MuTHER [41]	1.6E-19	4	103,780,757	ILMN_1800733	MANBA	rs228611	1.6E-19	Same	Same
	rs228611	0	1.00	Prefrontal cortex (Huntington's)[35]	1.4E-08	4	103,780,757	10025907439	MANBA	rs228611	1.4E-08	Same	Same
	rs228611	0	1.00	Lymphocytes[42]	2.0E-08	4	103,780,757		MANBA	rs228611	2.0E-08	Same	Same
	rs228611	0	1.00	Visual cortex (Alzheimer's)[35]	5.6E-05	4	103,780,757	10025907439	MANBA	rs228611	5.6E-05	Same	Same
	rs228611	0	1.00	Cerebellum - all samples[35]	6.7E-02	4	103,780,757	10025907439	MANBA	rs228611	6.7E-02	Same	Same
	rs909349	5,393	0.97	Monocytes[43]	2.5E-121	4	103,775,364		MANBA	rs909349	2.5E-121	Same	0.97
	rs7665090	10,106	0.97	OmentalAdipose[36]	6.5E-42	4	103,770,651	10025907439	MANBA	rs7665090	6.5E-42	Same	0.97
	rs228611	0	1.00	Blood[40]	1.3E-30	4	103,780,757	4230168	MANBA	rs7665090	3.9E-37	0.97	0.97
	rs7665090	10,106	0.97	Prefrontal cortex - all samples[35]	2.4E-26	4	103,770,651	10025907439	MANBA	rs7665090	2.4E-26	Same	0.97

Supplementary Table 11 (continued).

Locus name; index SNP	*eSNP*									*Best eSNP*			
	eSNP rsID	*Dist. index SNP*	*r^2*	*eQTL tissue[REF]*	*P-value*	*Chr*	*position*	*Probe**	*Transcript***	*Best eSNP rsID*	*P-value*	*r^2 to eSNP*	*r^2 to index SNP*
NFKB1 rs228611	rs228614	16,928	0.91	Blood[40]	2.0E-22	4	103,797,685	4230168	MANBA	rs7665090	3.9E-37	0.87	0.97
	rs2866413	4,632	0.97	Prefrontal cortex - all samples[35]	5.8E-17	4	103,776,125	10025907439	MANBA	rs7665090	2.4E-26	1	0.97
	rs2866413	4,632	0.97	Visual cortex - all samples[35]	5.8E-08	4	103,776,125	10025907439	MANBA	rs2866413	5.8E-08	Same	0.97
	rs228614	16,928	0.91	Blood[44]	2.1E-05	4	103,797,685	HSG00228144	MANBA	rs228614	2.1E-05	Same	0.91
	rs404574	21,499	0.90	SubCutAdipose MuTHER[41]	1.7E-14	4	103,802,256	ILMN_1800733	MANBA	rs404574	1.7E-14	Same	0.84
	rs7674640	20,929	0.81	Lung[45]	<2E-16	4	103,759,828	100150393_TGI_at	CISD2	rs7674640	<2E-16	Same	0.81
ZNF204 rs7759001	rs7759001	0	1.00	Intestine normal ileum[46]	1.3E-10	6	27,341,409		ZNF391	rs7759001	1.3E-10	Same	Same
	rs7759001	0	1.00	Cerebellum - normal samples[35]	5.5E-09	6	27,449,388	10025913649	BC035154	rs7759001	5.5E-09	Same	Same
	rs2143062	12,443	1.00	Cerebellum (Alzheimer's)[35]	1.6E-11	6	27,461,831	10025913649	BC035154	rs2143062	1.6E-11	Same	1.00
	rs2143062	12,443	1.00	Prefrontal cortex (Huntington's)[35]	1.4E-05	6	27,461,831	10025913649	BC035154	rs2143062	1.4E-05	Same	1.00
	rs9368508	19,561	1.00	Cerebellum (Huntington's)[35]	3.4E-06	6	27,429,827	10025913649	BC035154	rs9368508	3.4E-06	Same	0.95
	rs10755644	4,696	1.00	SubCutAdipose[36]	7.0E-05	6	27,444,692	10025933640	ZNF391	rs10755644	7.0E-05	Same	0.90
	rs10807021	15,516	0.95	OmentalAdipose[36]	1.5E-08	6	27,464,904	10025913649	BC035154	rs10807021	1.5E-08	Same	0.86
	rs4713086	9,037	0.95	Periph artery plaque‡	1.9E-07	6	27,458,425	100139132_TGI_at	BC035154	rs4713086	1.9E-07	Same	0.86
	rs980963	19,528	0.86	Cerebellum - all samples[35]	2.3E-05	6	27,468,916	10025933640	ZNF391	rs980963	2.3E-05	Same	0.86
	rs10807020	15,493	0.95	Cerebellum - normal samples[35]	5.3E-05	6	27,464,881	10025907341	NM_178534	rs10807020	5.3E-05	Same	0.86
	rs10807021	15,516	0.95	Visual cortex - all samples[35]	1.3E-03	6	27,464,904	10025908866	ZNF184	rs10807021	1.3E-03	Same	0.86
KBTBD2 rs3750082	rs6462431	15,512	0.93	Blood[40]	2.3E-38	7	32,901,964	4920372	KBTBD2	rs6462431	2.3E-38	Same	0.89
	rs3750082	0	1.00	LCL asthmatics [47]	8.1E-17	7	32,886,452	212447_at	KBTBD2	rs7785065	8.1E-17	0.89	0.89
	rs3750082	0	1.00	LCL MuTHER[41]	1.1E-06	7	32,886,452	ILMN_1784540	KBTBD2	rs13230763	8.3E-08	0.80	0.89
	rs2392152	19,378	0.89	Liver[37]	2.0E-10	7	32,867,074		HSS00226368	rs2392152	2.0E-10	Same	0.85
	rs4723221	34,280	0.82	Lung[45]	6.4E-08	7	32,920,732	100132413_TGI_at	KBTBD2	rs4723221	6.4E-08	Same	0.82

Supplementary Table 11 (continued).

Locus name; index SNP	*eSNP*									*Best eSNP*			
	eSNP rsID	*Dist. index SNP*	r^2	*eQTL tissue[REF]*	*P-value*	*Chr*	*position*	*Probe**	*Transcript***	*Best eSNP rsID*	*P-value*	r^2 *to eSNP*	r^2 *to index SNP*
AP5B1 rs4014195	rs11227281	10,264	1.00	Intestine -normal ileum[46]	3.8E-05	11	65,496,558		EIF1AD	rs11227281	3.8E-05	Same	1.00
	rs11604451	44,888	1.00	LCL (Degner - DNAse QTLs)[48]	2.9E-04	11	65,308,286		DNAse QTL 65308500-65308600	rs11604451	2.9E-04	Same	1.00
PTPRO rs7956634	rs2193172	11,148	1.00	Liver (UChicago)[38]	6.6E-03	12	15,223,609	A_23_P204304	PTPRO	rs2193172	6.6E-03	Same	1.00
INHBC rs1106766	rs1106766	0	1.00	ER+ breast tumor cells[49]	7.7E-06	12	56,095,723		GLS2	rs11614506	3.4E-07	0.80	0.80
DPEP1 rs164748	rs164749	68	1.00	Liver[37]	2.5E-09	16	88,235,725		C16orf55 (SPATA33)	rs164749	2.5E-09	Same	1.00
	rs460879	4,597	1.00	Prefrontal cortex (Huntington's)[35]	3.0E-08	16	88,240,390	10025907286	C16orf55 (SPATA33)	rs460879	3.0E-08	Same	1.00
	rs460879	4,597	1.00	OmentalAdipose[36]	2.7E-06	16	88,240,390	10025902450	CHMP1A	rs460879	2.7E-06	Same	1.00
	rs154657	196	1.00	Periph artery plaque‡	1.7E-05	16	88,235,597	100143418_TGI_at	SPATA2L	rs154657	1.7E-05	Same	1.00
	rs460879	4,597	1.00	Cerebellum (Huntington's)[35]	3.1E-05	16	88,240,390	10025902450	CHMP1A	rs460879	3.1E-05	Same	1.00
	rs459920	22,535	0.97	OmentalAdipose[36]	3.7E-44	16	88,258,328	10025907286	C16orf55 (SPATA33)	rs459920	3.7E-44	Same	0.97
	rs459920	22,535	0.97	SubCutAdipose[36]	1.2E-30	16	88,258,328	10025907286	C16orf55 (SPATA33)	rs459920	1.2E-30	Same	0.97
	rs459920	22,535	0.97	Cerebellum -all samples[35]	1.8E-30	16	88,258,328	10025907286	C16orf55 (SPATA33)	rs459920	1.8E-30	Same	0.97
	rs459920	22,535	0.97	Prefrontal cortex - all samples[35]	4.8E-23	16	88,258,328	10025907286	C16orf55 (SPATA33)	rs459920	4.8E-23	Same	0.97
	rs459920	22,535	0.97	Liver[36]	2.1E-21	16	88,258,328	10025907286	C16orf55 (SPATA33)	rs459920	2.1E-21	Same	0.97
	rs459920	22,535	0.97	Visual cortex -all samples[35]	2.7E-17	16	88,258,328	10025907286	C16orf55 (SPATA33)	rs459920	2.7E-17	Same	0.97
	rs2115401	32,317	0.97	Peripheral artery plaque‡	3.6E-07	16	88,268,110	100138862_TGI_at	C16orf55 (SPATA33)	rs2115401	3.6E-07	Same	0.97
	rs467035	31,993	0.97	Peripheral artery plaque‡	6.0E-06	16	88,267,786	100131451_TGI_at	SNAI3	rs467035	6.0E-06	Same	0.97
	rs258319	23,732	0.97	Temporal cortex[50]	4.8E-05	16	88,259,525	ILMN_1759261	C16orf55 (SPATA33)	rs258319	4.8E-05	Same	0.97
	rs2115401	32,317	0.97	Liver (UWash)[38]	1.7E-04	16	88,268,110	1300411	C16orf55 (SPATA33)	rs2115401	1.7E-04	Same	0.97

Supplementary Table 11 (continued).

Locus name; index SNP	*eSNP*									*Best eSNP*			
	eSNP rsID	*Dist. index SNP*	r^2	*eQTL tissue*[REF]	*P-value*	*Chr*	*position*	*Probe**	*Transcript***	*Best eSNP rsID*	*P-value*	r^2 *to eSNP*	r^2 *to index SNP*
DPEP1 rs164748	rs2115401	32,317	0.97	Liver (UChicago)[38]	<1e-16	16	88,268,110	A_24_P159335	C16orf55 (SPATA33)	rs2115401	<1e-16	Same	0.97
	rs459920	22,535	0.97	Liver (UChicago)[38]	<1e-16	16	88,258,328	A_23_P106694	CHMP1A	rs459920	<1e-16	Same	0.97
TP53INP2 rs6088580	rs2273684	244,713	0.94	Blood[40]	7.6E-08	20	32,993,427	7150537	ACSS2	rs2273684	7.6E-08	Same	0.94
	rs2273684	244,713	0.94	Blood[40]	1.5E-06	20	32,993,427	2680161	GSS	rs2273684	1.5E-06	Same	0.94
	rs6059909	145,362	0.84	Lymphocytes[51]	2.2E-03	20	32,603,352	GI_31563517-A	MAP1LC3A	rs6059909	2.2E-03	Same	0.81

* Expression QTL results were identified for our index SNPs or their proxies within the following dataset sources: whole blood samples,[40, 44] Epstein-Barr transformed B-lymphoblastoid cell lines (LCL) from population samples,[41, 42, 47] DNase-I QTLs in LCLs,[48] fresh lymphocytes,[51] peripheral blood monocytes,[43] ER+ breast cancer tumor cells,[49] omental and/or subcutaneous adipose,[36, 41] peripheral artery plaque intestine,[46] lung,[45] brain,[35, 50] and liver.[36-39]

‡ Unpublished (Emilsson)

Supplementary Table 12. Background information on novel replicated loci**.**

SNPID (Locus name)	Evidence from functional databases		Gene prioritized for functional work	Description of Genes in each Region*
	Source	Gene		
rs228611 (*NFKB1*)	eSNP	*MANBA*	*NFKB1* closest gene	*MANBA* encodes beta-mannosidase, a lysosomal enzyme that catalyzes the final exoglycosidase step in the degradation pathway for N-linked oligosaccharide moieties of glycoproteins (RefSeq). Rare mutations have been identified as the cause for beta-mannosidosis (OMIM #248510). A mouse model has linked *MANBA* to lysosomal storage disease in multiple organs, including the kidney.[52] *NFKB1* encodes a pleiotropic transcription factor that is present in almost all cell types and is the endpoint of a series of signal transduction events that are initiated by a vast array of stimuli related to many biological processes such as inflammation, immunity, differentiation, cell growth, tumorigenesis and apoptosis (RefSeq).
	GRAIL	NO		
	DEPICT	NO		
rs7759001 (*ZNF204*)	eSNP	NA	NO	ZNF204 is known as a transcribed pseudogene (RefSeq). ZNF391 and ZNF184 are two neighboring zinc finger protein-coding genes (RefSeq). Little is known about other nearby genes in the region.
	GRAIL	NO		
	DEPICT	NO		
rs1106766 (*INHBC*)	eSNP	NO	NO	*R3HDM2* encodes a protein with R3H domain. *INHBC* encodes the beta C chain of inhibin, which forms heterodimers with beta A and beta B subunits. Inhibins are involved in hormonal secretion and growth and differentiation of various cell types (RefSeq). *GLI1* encodes a transcription factor that is activated by the sonic hedgehog signal transduction cascade that regulates stem cell proliferation (RefSeq). The hedgehog-GlI pathway has been implicated in kidney fibrosis in mouse studies.[53] *ARHGAP9* encodes a member of the Rho-GAP family of GTPase activating proteins, converting them to an inactive GDP-bound state (RefSeq).
	GRAIL	NO		
	DEPICT	NO		
rs10513801 (*ETV5*)	eSNP	NA	NO	The *ETV5* gene is a ubiquitously expressed transcription factor. A role in renal development has been shown in a mouse model[54]. In humans, it has not yet been connected to kidney disease. *DGKG* encodes diacylglycerol Kinase, Gamma, a glycerol kinase that metabolizes 1,2,diacylglycerol to produce the second messenger phosphatidic acid. The transcript is expressed in kidney.
	GRAIL	NO		
	DEPICT	NO		
rs3850625 (*CACNA1S*)	eSNP	NA	NO	*CACNA1S* encodes one of five subunits of the slowly inactivating L-type voltage-dependent calcium channel, which plays a role in skeletal muscle contraction. Known mutations in *CACNA1S* have been associated with susceptibility to malignant hyperthermia, hypokalemic periodic paralysis, and thyrotoxic periodic paralysis. The identified variant is a non-synonymous coding SNP at a highly conserved position (R1539C on protein level) that is predicted as damaging or pathogenic by 3 out of 4 prediction software.
	GRAIL	NO		
	DEPICT	NO		
rs10491967 (*TSPAN9*)	eSNP	NA	*TSPAN9*	*TSPAN9* encodes for a member of the tetraspanin family, which assemble in complexes with additional proteins such as integrins to transduct signals. Its association to renal function is unclear; mutations in some integrins cause monogenic kidney disease, as do mutations in the gene paralog CD151. Other genes in the region have not specifically been connected to kidney disease.
	GRAIL	NO		
	DEPICT	*TSPAN9* FDR<0.05		

Supplementary Table 12 (continued).

SNPID (Locus name)	Evidence from functional databases		Gene prioritized for functional work	Description of Genes in each Region*
	**Source**	**Gene**		
rs164748 (*DPEP1*)	**eSNP**	*C16orf55* (also known as *SPATA33*)	*DPEP1*	*DPEP1* encodes dipeptidase 1, an enzyme responsible for hydrolysis of glutathione and certain types of antibiotics in the kidney membrane[55,56] and is highly expressed in the kidney and pancreas (GeneCards), but there are no studies linking this gene to kidney function. *RPL13* encodes ribosomal protein L13, a protein within the large 60S ribosomal subunit complex[57]. There are no publications linking RPL13 to the kidney or kidney function. *SNORD68* encodes small nucleolar RNA C/D box 68 a class molecules that modify other RNAs. This particular class of snoRNA is responsible for RNA methylation. There are no known connections to kidney function or disease. *SPG7* encodes paraplegin, an enzyme component of m-AAA protease (a mitochondrial enzyme responsible for degradation of malformed or misfolded proteins).[58] Mutations in the *SPG7* gene lead to monogenic forms of spastic paraplegia.[59, 60, 60, 61, 61] There are no reported studies linking *SPG7* and kidney function. *ANKRD11* encodes ankyrin repeat domain protein 11, a protein inhibiting ligand-dependent activation of transcription. Variation in *ANKRD11* can lead to KBG syndrome (a disorder with abnormal skeletal development and delay in neurological development.[62, 63, 63] No associations of *ANKRD*11 and kidney function have been published. *C16orf55* (also known as *SPATA33*) encodes spermatogenesis-associated protein 33 and is thought to be involved in spermatogenesis. No associations with kidney disease have been identified.
	**GRAIL**	NO		
	**DEPICT**	DPEP1 FDR<0.05		
rs8091180 (*NFATC1*)	**eSNP**	NA	*NFATC1*	*NFATC1* encodes for nuclear factor of activated T-cells (cytoplasmic, calcineurin dependent 1) and is a part of a complex involved in the activation of immune response, specifically activation of the T-cell antigen receptor.[64] In rodents, *NFAT1C* is potentially involved in proximal tubules after injury[65] and activation of this nuclear factor may lead to glomeruloscerosis by mutant forms of *TRPC6* - a podocyte protein involved in maintaining the filtration barrier.[66] *NFAT*1C has not been linked with kidney function in humans. *ATP9B* encodes an ATPase, class II, type 9b - a transmembrane transporter. No association with kidney disease has been previously reported. *CTDP1* encodes the c-terminal domain of RNA polymerase II subunit A phosphatase. Variations in *CTDP1* have been associated with congenital cataracts, facial dimorphism and neuropathy in Bulgarian gypsy populations.[67] There have been no previous reports of *CTDP1* and kidney disease.
	**GRAIL**	*NFATC1* p=0.03		
	**DEPICT**	NO		
rs11666497 (*SIPA1L3*)	**eSNP**	NA	NO	*SIPA1L3* encodes the signal-induced proliferation-associated 1 like 3 protein. There are no published reports in the literature describing this gene or its protein product. *WDR87* encodes the WD repeat-containing protein 87, a protein 2.8K amino acids in length. There are no published reports of this gene or gene product in the literature.
	**GRAIL**	NO		
	**DEPICT**	NO		

Supplementary Table 12 (continued).

SNPID (Locus name)	Evidence from functional databases		Gene prioritized for functional work	Description of Genes in each Region*
	**Source**	**Gene**		
rs4667594 (*LRP2*)	**eSNP**	NA	NO	*LRP2* encodes the megalin receptor, which plays an important role in the reabsorption of albumin and other low-molecular-weight proteins[68] along with cubilin (*CUBN*) and amnionless (*AMN*). *LRP2* mutations are associated with Donnai-Barrow and facio-oculo-acoustico-renal syndromes.[69] *DHRS9* is a protein-coding gene that encodes dehydrogenase/reductase member 9 (GeneCards). It is a 3-alpha-hydroxysteroid dehydrogenase that is responsible for the synthesis of dihydroxyprotesterone; low levels of activity with retinoids have also been identified (UCSC). There are no published studies linking this gene to kidney function. *ABCB11* belongs to the ATP-binding cassette superfamily. The protein is involved in bile salt export (www.genecards.org), and mutations are involved in familial intrahepatic cholestasis (OMIM). In addition, this gene may be involved with bile acid transport in the kidney.[70]
	**GRAIL**	*LRP2* p=0.0004		
	**DEPICT**	*LRP2* FDR<0.05		
rs6795744 (*WNT7A*)	**eSNP**	NA	*WNT7A* closest gene	*WNT7A* is a member of set of genes that are signaling proteins, specifically those that are involved in embryogenesis (www.genecards.org). Mutations in this gene have been associated with Fuhrmann syndrome (OMIM #228930) and the Al-Awadi/Raas-Rothschild/Schinzel phocomelia syndrome (AARRS; OMIM #276820), disorders characterized by limb malformation. Wnt-7a signalling may allow for the development of sexual dimorphism via development of the mullerian ducts.[71] *TPRXL* is a homeobox gene thought to be involved in embryonic development (GeneCards). There are no published papers linking this gene to kidney function. Of the remaining genes in the region, only *XPC* has been previously linked to kidney function in the published literature. *XPC* is a DNA repair gene. Mutations result in Xeroderma pigmentosum, a disease characterized by sunlight sensitivity and early carcinomas. Mutations in *XPC* may be associated with renal cell carcinoma.[72]
	**GRAIL**	NO		
	**DEPICT**	NO		
rs7956634 (*PTPRO*)	**eSNP**	*PTPRO*	*PTPRO* closest gene	*RERG* belongs to the RAS superfamily and is involved with cell proliferation and tumor pathogenesis (GeneCards). *RERG* expression may be lost in kidney cancer.[73] *PTPRO* is expressed in the podocyte foot processes of the kidney; mutations in *PTPRO* are associated with autosomal-recessive nephrotic syndrome.[74] Knock-out mice display reduced eGFR but no proteinuria[75]. *EPS8 is* an epidermal growth factor receptor pathway substrate gene (GeneCards). Eps8 proteins are involved in the organization of actin filaments.[76]
	**GRAIL**	NO		
	**DEPICT**	NO		
rs10277115 (*UNCX*)	**eSNP**	NA	*UNCX* (no other genes in the LD block)	*UNCX* is a transcription factor that is involved in neurogenesis and somitogenesis (GeneCards). In addition, it may be involved in differentiation of the axial skeleton (GeneCards). There is no published literature linking it to kidney function. *ZFAND2A* is a protein coding gene involved in zinc ion binding (GeneCards). It has been shown to be part of a network of genes expressed in human renal epithelial cells in response to cadium exposure, a known nephrotoxin.[77] *GPER* is a member of the G-protein coupled receptor family with a primary role of binding estrogen (GeneCards). *GPER* may mediate the effects of estrogen (but not aldosterone)[78] on the vasculature[79] including the rat kidney.[80] *GPER* binds estrogen in addition to other substances including endocrine disruptors.[81]
	**GRAIL**	NO		
	**DEPICT**	NO		

Supplementary Table 12 (continued).

SNPID (Locus name)	Evidence from functional databases		Gene prioritized for functional work	Description of Genes in each Region*
	**Source**	**Gene**		
rs12136063 (*SYPL2*)	**eSNP**	*SYPL2*	*SYPL2*	*SYPL2* encodes synaptophysin-like 2 protein (also known as mitsugumin 29), a membrane protein involved in the communication between the transverse tubular and junctional sarcoplasmatic reticulum membranes (Entrez Gene). It is expressed in the kidney (www.proteinatlas.org). There are no publications linking this gene to kidney function. *PSMA5* encodes proteasome (prosome, macropain) subunit alpha type 5, which is a proteosome involved in the processing of MHC class I peptides (Entrez Gene) and is expressed in many tissues including the kidney (ProteinAtlas). There are no publications linking this gene to kidney function. There is no disease linked to this gene in OMIM. *ATXN7L2* encodes for ataxin 7-like 2 protein (GeneCards), which is expressed in several tissues including the kidney (ProteinAtlas). There is no publication linking this gene to kidney function. *AMIGO1* encodes adhesion molecule with Ig-like domain 1, which is part of a family of transmembrane proteins involved in axon tract development.[82] It is expressed in the kidney. *CELSR2* encodes cadherin EGF LAG seven-pass G-type receptor 2, which is a member of the flamingo subfamily cadherins which does not interact with catenins (Entrez Gene). It is a plasma membrane protein postulated to be involved in contact-mediated communication and expressed in many tissues including the kidney.
	**GRAIL**	NO		
	**DEPICT**	*SYPL2* FDR<0.05		
rs2802729 (*SDCCAG8*)	**eSNP**	*SDCCAG8*	NO	*SDCCAG8* encodes serologically defined colon cances antigen 8, a centrosome associated protein involved in interphase and mitosis (Entrez Gene). Truncating mutations cause Senior-Loken syndrome 7 (OMIM #613615), an autosomal recessive ciliopathy with nephronophthisis and Leber congenital amaurosis. *CEP170* encodes centrosomal protein 170kDa, which is the major microtubule-organizing center in animals (Entrez Gene). It is expressed in several tissues including the kidney, but has not been linked to kidney function in any publication. *AKT3* encodes v-akt murine thymoma viral oncogene homolog 3, which is a kinase regulating cell signaling in response to insulin and rowth factors; it is stimulated by PDGF, insulin and IGF-1 (Entrez Gene). It shows low-level expression in the kidney (ProteinAtlas). Rare mutations cause megalencephaly-polymicrogyria-polydactyly-hydrocephalus syndrome (MPPH, OMIM #603387), which does not show organ involvement beyond central nervous defects. There are no publications linking this gene to kidney function.
	**GRAIL**	NO		
	**DEPICT**	NO		
rs17216707 (*BCAS1*)	**eSNP**	NA	*BCAS1* but no fish orthologs	*BCAS1* encodes breat carcinoma amplified sequence 1, which has been implicated as a breast cancer oncogene (Entrez Gene). There is no expression in the kidney (ProteinAtlas). *CYP24A1* encodes cytochrome P450, family 24, subfamily A, polypeptide 1, which initiates the degradation of 1,25 hydrox-Vitamin D3, thus regulating calcium homeostasis (Entrez Gene). Rare mutations in this gene cause infantile hypercalcemia (OMIM #143880). *CYP24A1* is expressed in the kidney. *PFDN4* encodes prefoldin subunit 4, which is part of a molecular chaperone complex needed for correct folding of newly synthesised polypeptides (Entrez Gene). There are no publications linking *PFDN4* to kidney function. *SUMO1P1* encodes *SUMO1* (small ubiquitin-like modifier 1) pseudogene 1 (Entrez Gene); *SUMO1* is part of a post-translational modification system regulating NFKB under high glucose conditions in kidney mesangial cells;[83] sumoylation protects from oxidative stress.[84] There is no publication linking *SUMO1P1* to kidney function.
	**GRAIL**	NO		
	**DEPICT**	NO		

Supplementary Table 12 (continued).

<table>
<tr><th rowspan="2">SNPID (Locus name)</th><th colspan="2">Evidence from functional databases</th><th rowspan="2">Gene prioritized for functional work</th><th rowspan="2">Description of Genes in each Region*</th></tr>
<tr><th>Source</th><th>Gene</th></tr>
<tr><td rowspan="3">rs3750082 (KBTBD2)</td><td>eSNP</td><td>KBTBD2</td><td rowspan="3">KBTBD2</td><td rowspan="3">AVL9 plays a role in late exocytic transport,[85] at the level of the Golgi.[86] This gene has not formally been associated with kidney function in the published literature. LSM5 is a Sm-like protein that plays a role in pre-mRNA splicing (GeneCards). It has not previously been linked to kidney function in the published literature. There is little known about KBTBD2, and no published literature linking it to kidney function. There are several other genes in the region.</td></tr>
<tr><td>GRAIL</td><td>NO</td></tr>
<tr><td>DEPICT</td><td>KBTBD2 FDR<0.05</td></tr>
<tr><td rowspan="3">rs6088580 (TP53INP2)</td><td>eSNP</td><td>NO</td><td rowspan="3">NO</td><td rowspan="3">TP53INP2 regulates both transcription and autophagy (UCSC). It is a scaffold protein that interacts with VMP1.[87] It has not been formally linked to kidney function in the literature. There are dozens of additional genes in the region.</td></tr>
<tr><td>GRAIL</td><td>NO</td></tr>
<tr><td>DEPICT</td><td>ACSS2 and NCOA6 FDR<0.05</td></tr>
<tr><td rowspan="3">rs6459680 (RNF32)</td><td>eSNP</td><td>NA</td><td rowspan="3">NO</td><td rowspan="3">RNF32 plays a role in spermatogenesis. The gene has not previously been identified in associaiton with kidney function. LMBR1 plays a role in limb malformation, which may occur via altered Sonic hedgehog signaling. The gene has not been connected to kidney function or disease.</td></tr>
<tr><td>GRAIL</td><td>NO</td></tr>
<tr><td>DEPICT</td><td>NO</td></tr>
<tr><td rowspan="3">rs4014195 (AP5B1)</td><td>eSNP</td><td>NA</td><td rowspan="3">AP5B1 but no existing Morpholino</td><td rowspan="3">KAT5 encodes for K(Lysine) acetyltransferase 5, which is important in transcriptional regulation. It has not been previously identified in association with kidney function. OVOL1 encodes for a transcription factor. OVOL1 deficient mice of C57BL/6 background show increased perinatal lethality and other abnomalities, including cystic kidneys.[88]</td></tr>
<tr><td>GRAIL</td><td>NO</td></tr>
<tr><td>DEPICT</td><td>AP5B1 FDR<0.05</td></tr>
<tr><td rowspan="3">rs2712184 (IGFBP5)</td><td>eSNP</td><td>NA</td><td rowspan="3">NO</td><td rowspan="3">IGFBP5 (insulin-like growth factor binding protein 5) is a protein-coding gene that either induces or suppresses cell proliferation. IGFBP5 participates in cellular pathways of adaptation to hypertonicity in renal medulla under TonEBP control.[89] IGFBP2 (insulin-like growth factor binding protein 2, 36kDA) regulates cell growth by enhancing or suppressing IGF bioavailability. RNA and protein are ubiquitously expressed. IGFBP2 is the most expressed among IGF binding proteins, particularly in glomerular mesangial cells where it is controlled by angiotensin and glucose concentrations.[90] TNP1 (transition protein 1) is a protein-coding gene. In the course of spermiogenesis, TNP1 is a spermatid-specific product that replaces histone and is itself replaced in the mature sperm by protamines. It is expressed in testis. There is no published literature relating this gene or its protein product to kidney function.</td></tr>
<tr><td>GRAIL</td><td>NO</td></tr>
<tr><td>DEPICT</td><td>NO</td></tr>
<tr><td rowspan="3">rs10994860 (A1CF)</td><td>eSNP</td><td>NA</td><td rowspan="3">A1CF</td><td rowspan="3">A1CF is a protein coding gene that mediates the deamination of apolipoprotein B mRNA through a multi-componenent enzyme complex including APOBEC-1 and a complementation factor coded by A1CF gene. It is expressed in the gastrointestinal tract, liver, pancreas, brain and kidneys. One publication[20] links this gene to kidney reabsorption of urate (elevated serum urate concentrations). ASAH2B (N-acylsphingosine amidohydrolase (non-lysosomal ceramidase) 2B) is a protein-coding gene. Ceramidases are a group of enzymes which catalyze the hydrolysis of ceramides to produce sphingosine but ASAH2B lacks the active site and therefore could be catalytically inactive. No publications link this gene to kidney function. PRKG1 encodes the soluble alpha and I beta1 isoforms of the cGMP dependent protein kinase, involved in the regulation of cardiovascular and neuronal functions, smooth muscle relaxation, platelet aggregation, and in cell growth by modulating cellular calcium.</td></tr>
<tr><td>GRAIL</td><td>NO</td></tr>
<tr><td>DEPICT</td><td>A1CF FDR<0.05</td></tr>
</table>

Supplementary Table 12 (continued).

SNPID (Locus name)	Evidence from functional databases		Gene prioritized for functional work	Description of Genes in each Region*
	**Source**	**Gene**		
rs9682041 (*SKIL*)	**eSNP**	NA	*SKIL*	The protein encoded by SKI-like oncogene is a component of the SMAD pathway, that has a regulatory role on the cell division and differentiation through TGFß. RNA and protein are expressed in most human tissues except pancreas. *SKIL* is implicated in ubiquitin dependent tubulointerstitial fibrosis along with TGFbeta1.[91] Claudin 11 is a member of the claudin family of tight junction associated proteins. The protein encoded by this gene is a major component of the central nervous system (CNS) myelin and plays an important role in regulating proliferation and migration of oligodendrocytes. RNA and protein are expressed in Sertoli cells in testis and oligodendrocytes in the CNS. CLDN11 Claudin family is differentially expressed in tight junctions of human cortical nephron and influences pathologies involving abnormalities of absorption.[92] Its expression is significantly unregulated in genetic model of polycystic kidney in early stages.[93] RNA is expressed in cerebral cortex, while the protein is strongly expressed in hyppocampus and prostate. There are no publications linking this gene to kidney function.
	**GRAIL**	*SKIL* p=0.02		
	**DEPICT**	*SKIL* FDR<0.05		
rs163160 (*KCNQ1*)	**eSNP**	NA	*KCNQ1* work already completed	*KCNQ1* encodes a voltage-gated potassium channel required for cardiac repolarization. RNA is ubiquitously expressed but the protein product is mostly expressed in glandular cells. In addition to be recognized as a gene conferring risk of T2D in Caucasians and more recently in African Americans,[94] it is also reported as a gene conferring susceptibility to diabetic nephropathy in Asians[95] and CKD in African Americans.[96] *TRPM5* encodes the transient receptor potential cation channel, subfamily M, member 5 gene, a member of the transient receptor potential (TRP) protein family with structural features typical of ion channels (non-selctive cations except Ca2+) and mediates a transient membrane depolarization in the presence of low concentrations of intracellular calcium. mRNA is found mainly in the prostate, testis, ovary, colon and leukocytes but the protein is expressed in the majority of human tissues. There are no publications linking this gene or its protein to kidney function. The tumor suppressing subtransferable candidate 4 (*TSSC4*) gene is located in the p15.5 region of the chromosome 11, a region involved in Wilm's tumor and a known important tumor-supressor gene region. TSSC4 RNA is widely expressed in human tissues while protein is strongly expressed in renal tubules and moderately in digestive system as well as male and female reproductive systems.
	**GRAIL**	NO		
	**DEPICT**	*IGF2* (FDR<0.05) but very far away from lead SNP		

*Cited web resources: Entrez Gene: http://www.ncbi.nlm.nih.gov/gene; GenCards: www.genecards.org; OMIM: http://www.ncbi.nlm.nih.gov/omim; ProteinAtlas: www.proteinatlas.org; RefSeq: http://www.ncbi.nlm.nih.gov/refseq/; UCSC: www.genome.ucsc.edu.

Supplementary Table 13. DEPICT tissue and cell-type enrichment.*

MeSH ID	Name	MeSH# first level term	MeSH second level term	P-value	FDR	Genes at associated loci§
A05.810	Urinary Tract	Urogenital System	Urinary Tract	0.000226	<0.025	*NAT8, SLC17A3, SLC22A2, SLC17A1, LRP2, UMOD, SLC7A9, SLC6A13, SLC34A1, ENSG00000204872, PCK1, SLC47A1, WDR72*
A05.810.453	Kidney	Urogenital System	Urinary Tract	0.000292	<0.025	*NAT8, SLC17A3, SLC22A2, SLC17A1, LRP2, UMOD, SLC7A9, SLC6A13, SLC34A1, ENSG00000204872, PCK1, SLC47A1, WDR72*
A03.620	Liver	Digestive System	Liver	0.000663	<0.025	*ITIH1, LPA, SLC17A2, SLC22A1, ITIH3, KNG1, LEAP2, GCKR, SLC22A7, CPS1, A1CF*
A11.436.348	Hepatocytes	Cells	Epithelial Cells	0.001338	<0.025	*ITIH3, A1CF, LEAP2, SLC17A2, GCKR, AGMAT, ITIH1, SCGN, LCAT, SLC22A3, SLC7A9, SLC22A1*
A06.407.071.140	Adrenal Cortex	Endocrine System	Endocrine Glands	0.001574	<0.025	*ENSG00000256731, SLC47A1, FNDC4, KCNQ1, RERG, CASP9*
A06.407.071	Adrenal Glands	Endocrine System	Endocrine Glands	0.002988	0.025	*SLC47A1, FNDC4, ENSG00000256731, KCNQ1, RERG, MAMSTR, CASP9*

*DEPICT was used to assess whether genes at associated loci were highly expressed in any of 209 tissue and cell type annotations. In total we found 6 significantly enriched tissues (FDR < 0.05). Only significant tissues are shown in the table.

# MeSH: Medical Subject Headings.

§ Lists of genes that are within an associated region and highly expressed in the given tissue or cell type.

Supplementary Table 14. DEPICT pathway analysis**.** Gene sets with P-value < 1e–05 are shown.

Reconstituted gene set ID	Reconstituted gene set name	Part of meta gene set	P-value	FDR	Reconstituted gene set genes at associated loci
ENSG00000186350	RXRA protein complex	NCOA1 protein complex	5.94E-08	<0.002	*NCOA6, SKIL, TRIB1, MED1, CDK12, EDC4, RELA, ARNT, ERBB2, PGAP3, NFATC1, PTPN12, NFKB1, A1CF, NRBP1, TPRKB, AFF4, NRIP1, NFATC3, INO80*
MP:0011423	Kidney Cortex Atrophy	Dilated Renal Tubules	3.40E-07	<0.002	*SLC7A9, SLC22A2, SLC34A1, VEGFA, NAT8, UMOD, DPEP1, AGMAT, PCK1, LRP2, PTPRO, IGF2, ENSG00000204872, ADAMTS5, DAB2, BMP4, SLC12A4, CA12, SLC6A13, TRIB1*
ENSG00000125124	BBS2 protein complex	BBS4 protein complex	4.76E-07	<0.002	*KNG1, UMOD, CPS1, ITIH3, ESRP2, RASIP1, PCK1, SLC7A9, AGMAT, SLC22A1, NAT8, LRP2, NUTF2, DACH1, ITIH4, EDC4, SLC22A2, ENSG00000204872, PSMD12, SGMS1*
ENSG00000124151	NCOA3 protein complex	NCOA1 protein complex	7.21E-07	<0.002	*TRIB1, MED1, NCOA6, FBXL20, ETV5, CDK12, VEGFA, GCKR, GLI2, RAI1, PSKH1, GTF3C2, MOV10, PIK3R1, PHLDA1, PGAP3, ITIH3, NFATC1, SETBP1, RMND5A*
MP:0003918	Decreased Kidney Weight	Dilated Renal Tubules	8.75E-07	0.002	*RAPSN, IGF2-AS, ACVR2A, NFATC3, PCK1, NAT8, SLC7A9, SLC6A13, IGF2, SLC34A1, PTPRO, ADAMTS5, PAPPA, IGF2R, UMOD, GSS, LCAT, TCEA3, SVEP1, AGMAT*
GO:0015145	Monosaccharide Transmembrane Transporter Activity	Monosaccharide Trans-membrane Transporter Activity	1.21E-06	0.002	*SLC7A9, ENSG00000204872, SLC34A1, AGMAT, LEAP2, NAT8, SLC22A2, TSPAN9, TRIM58, SLC2A9, DPEP1, C12orf68, ENSG00000230288, VEGFA, SLC28A2, DAB2, UMOD, SLC6A13, XYLB, OR2W3*
GO:0008514	Organic Anion Transmembrane Transporter Activity	Organic Cationanionzwitterion Transport	2.08E-06	0.006	*SLC34A1, SLC22A2, NAT8, SLC6A13, KNG1, SLC22A7, UMOD, SLC22A1, ITIH1, SLC7A9, AGMAT, WDR72, ENSG00000204872, INHBC, SLC47A1, A1CF, LRP2, XYLB, PCK1, DPEP1*
MP:0000521	Abnormal Kidney Cortex Morphology	Dilated Renal Tubules	2.60E-06	0.005	*UMOD, SLC7A9, SLC22A2, SLC34A1, AGMAT, VEGFA, NAT8, CA12, IGF2-AS, STC1, KNG1, GLI2, RASIP1, LRP2, DPEP1, EYA4, DACH1, SETBP1, PCK1, TSPAN9*
GO:2000117	Negative Regulation Of Cysteine-Type Endopeptidase Activity	Negative Regulation Of Cysteine-Type Endopeptidase Activity	3.26E-06	0.004	*OR2W3, UMOD, SLC34A1, NRIP1, RMND5A, PSMA5, PRELID1, PTPN12, ENSG00000187446, SNX17, SLC7A6, ENSG00000232656, PSKH1, IZUMO1, SLC22A2, TRIB1, IDI1, NFE2L2, DCDC5, LRP2*

Supplementary Table 14 (continued).

Reconstituted gene set ID	Reconstituted gene set name	Part of meta gene set	P-value	FDR	Reconstituted gene set genes at associated loci
MP:0001698	Decreased Embryo Size	Complete Embryonic Lethality During Organogenesis	4.30E-06	0.006	*PTPN12, HNRNPR, NCOA6, HSPA4, IGF2, AFF4, IGF2R, ACVR2B, NSD1, VEGFA, MED1, CELF1, NRF1, BMP4, SETDB1, LAMA5, JARID2, RIF1, RMND5A, INO80*
MP:0011108	Partial Embryonic Lethality During Organogenesis	Complete Embryonic Lethality During Organogenesis	4.61E-06	0.005	*IGF2, LAMA5, VEGFA, BMP4, TRIB1, RARB, PHLDA1, R3HDM2, RHOC, TSPAN9, JARID2, ACVR2B, AFF4, NSD1, PTPN12, SIPA1L3, RASIP1, A1CF, GLI2, GRB10*
MP:0001711	Abnormal Placenta Morphology	Abnormal Placenta Labyrinth Morphology	4.80E-06	0.005	*VEGFA, ITIH1, GRHL2, TRIB1, KNG1, IGF2, SPATA5L1, NSD1, ITIH3, ESRP2, INO80, RHOC, LEAP2, LAMA5, CELF1, ARNT, ITCH, ERBB2, NCOA6, PAPPA*
GO:0005355	Glucose Transmembrane Transporter Activity	Monosaccharide Transmembrane Transporter Activity	5.07E-06	0.005	*SLC7A9, ENSG00000204872, SLC34A1, AGMAT, SLC2A9, DPEP1, NAT8, SLC22A2, C12orf68, LEAP2, TRIM58, ENSG00000230288, VEGFA, SLC28A2, TSPAN9, GGT7, UMOD, SLC22A7, TFDP2, SLC6A13*
GO:0001655	Urogenital System Development	Kidney Development	5.43E-06	0.006	*UMOD, EYA4, DACH1, SLC34A1, SLC22A2, RARB, DPEP1, IGF2-AS, ADAMTS5, IGF2, SETBP1, GRHL2, ERBB2, GLI2, LRP2, SHH, PAPPA, ACVR2B, BMP4, STC1*
GO:0015149	Hexose Transmembrane Transporter Activity	Monosaccharide Transmembrane Transporter Activity	6.45E-06	0.006	*SLC7A9, ENSG00000204872, SLC34A1, AGMAT, SLC2A9, NAT8, SLC22A2, DPEP1, TSPAN9, LEAP2, C12orf68, TRIM58, ENSG00000230288, VEGFA, SLC28A2, GGT7, UMOD, PRELID1, SLC22A7, OR2W3*
MP:0002981	Increased Liver Weight	Decreased Circulating Cholesterol Level	7.33E-06	0.006	*ITIH1, PCK1, KNG1, CPS1, LEAP2, SLC22A1, SLC22A7, ACSS2, SLC7A9, ITIH3, GCKR, IDI1, LPA, INHBC, FASN, A1CF, DPEP1, ITIH4, GSS, IGF2R*
GO:0072001	Renal System Development	Kidney Development	7.35E-06	0.005	*UMOD, DPEP1, DACH1, SLC22A2, RARB, SLC34A1, ADAMTS5, EYA4, IGF2, IGF2-AS, SETBP1, BMP4, LRP2, NAT8, GLI2, SHH, CLDN11, ERBB2, LAMA5, CA12*
MP:0011346	Renal Tubule Atrophy	Dilated Renal Tubules	8.41E-06	0.005	*UMOD, SLC34A1, SLC7A9, DPEP1, VEGFA, PTPRO, SLC47A1, AGMAT, SLC22A2, SLC22A7, KNG1, PCK1, MANBA, CPS1, ENSG00000204872, NAT8, WDR72, SLC2A9, LEAP2, SLC22A3*
MP:0005459	Decreased Percent Body Fat	Abnormal Glucose Homeostasis	8.78E-06	0.005	*CTRL, CELA2B, TRIB1, PCK1, RAPSN, SORT1, ACSS2, SLC34A1, SLC7A9, ENSG00000187446, RSBN1L, FASN, STC1, THADA, LPA, PIK3R1, LMAN2, CASP9, IGF2R, PIGU*

Supplementary Table 15. Zebrafish knock-down results.*

	MO	Dose	*Pax2a*	*Nephrin*	*Slc20a1a*
**Clutch 1**	**Control**	N/A	0/33 (0%)	0/36 (0%)	0/51 (0%)
	Wnt7aa	250 uM	3/38 (8%)<br>p= 0.24	0/29 (0%)	0/29 (0%)
	Nfkb1	250 uM	0/33 (0%)	0/32 (0%)	0/30 (0%)
	Ptpro	250 uM	0/22 (0%)	0/27 (0%)	0/14 (0%)
	A1cf	250 uM	0/31 (0%)	0/36 (0%)	0/23 (0%)
	Tspan9a+b	125 uM each	1/25 (4%)<br>p=0.43	0/22 (0%)	1/19 (5%)<br>p=0.27
**Clutch 2**	**Control**	N/A	3/18 (17%)	0/21 (0%)	0/20 (0%)
	Kbtbd2	250 uM	0/20 (0%)<br>p=0.10	0/22 (0%)	0/17 (0%)
	Uncx	250 uM	3/30 (10%)<br>p=0.66	1/26 (4%)<br>p=1.00	0/19 (0%)
	Dpep	250 uM	1/26 (4%)<br>p=0.29	1/23 (4%)<br>p=1.00	0/24 (0%)
	Sypl2a+b	250 uM each	7/35 (20%)<br>p=1.00	1/25 (4%)<br>p=1.00	0/29 (0%)
**Clutch 3**	**Control**	N/A	0/37 (0%)	0/55 (0%)	0/41 (0%)
	Nfatc1	250 uM	2/26 (8%)<br>p=0.17	0/29 (0%)	0/31 (0%)
	Skila+b	62.5 uM each	0/41 (0%)	0/46 (0%)	0/32 (0%)

Zebrafish embryos were injected with morpholinos (MOs) targeting orthologous loci, and renal gene expression was assessed at 48 hours post-fertilization (hpf) by whole mount *in situ* hybridization. Renal markers examined include *pax2a* (global kidney), *nephrin* (podocyte), and *slc20a1a* (proximal tubule). Data are presented as the number of observed abnormalities per total number of embryos scored.

*P-values provided for non-0 results only.

Supplementary Table 16. Study information: discovery. Extensive study names are reported in the Acknowledgements section.

Study[REF]	Study Design	N§	Study characteristics	Creatinine Measurement	Cystatin measurement
3C[97, 98]	Prospective population-based	6440	**Study exclusions or disease enrichment**. None.<br>**Exclusions**. Of the 6440, those with genotypes and creatinine were 6431 and those with genotypes and cystatin C were 1243.	Modified kinetic Jaffe reaction.	Particle-enhanced immuno-nephelometric method (BNII, Dade-Behring/ Siemens)
Advance[99]	Randomised controlled trial	11,140	**Study exclusions or disease enrichment**. Multicenter trial done by 215 collaborating centres in 20 countries. All patients had T2D and were of Caucasian origin. Those with genotypes and creatinine were 2301.<br>**Exclusions**. NA	Serum creatinine was measured in local laboratories at individual study sites.	NA
AGES[100]	Population based	3664	**Study exclusions or disease enrichment**. None.<br>**Exclusions**. Sample exclusion criteria included sample failure, genotype mismatch with reference panel, and sex mismatch, resulting in clean genotype data on 3219 individuals.	Jaffé reaction.	NA
Amish[101, 102]	Population based "founder" cohort	1264	**Study exclusions or disease enrichment**. None.<br>**Exclusions**. Age <20, severe chronic disease, call rate <95%, pHWE<10E-6.	Modified kinetic Jaffe reaction.	Particle-enhanced immunonephelometric method (BNII, Dade-Behring).
ARIC[103]	Prospective, population-based	9713	**Study exclusions or disease enrichment**. None.<br>**Exclusions**. Of the 9713 genotyped individuals of European ancestry, we excluded 658 individuals based on discrepancies with previous genotypes, disagreement between reported and genotypic sex, one randomly selected member of a pair of first-degree relatives, or outlier based on measures of average DST or >8 SD away on any of the first 10 principal components.	Modified kinetic Jaffé reaction.	Particle enhanced immunonephelometric assay (N Latex Cystatin C, Dade Behring).
ASPS[104, 105]	Prospective study	922	**Study exclusions or disease enrichment**. Excluded were subjects with histrory of neuropsychiatric disease, previous stroke and/or TIA, and dementia.<br>**Exclusions**. Of the 922 participants who underwent genotyping, we made the following exclusions: sample call rate <98% (74). This resulted in 848 genotyped individuals.	Modified kinetic Jaffé reaction.	NA
AUSTWIN[106]	Families, population-based	9592	**Study exclusions or disease enrichment**. NA.<br>**Exclusions**. NA.	Jaffé method, Hitachi 917 analyser.	NA
BLSA[107]	Population based study	1200	**Study exclusions or disease enrichment**. None.<br>**Exclusions**. Those of non-European descent or with missing phenotype information.	Modified kinetic Jaffé reaction.	NA
BMES[108-110]	Prospective cohort study	2761	**Study exclusions or disease enrichment**. None.<br>**Exclusions**. Sample call rate <95% (n=9), outlying autosomal heterozygosity (n=28), sex discrepancies or ambiguous sample identification (n=69), cryptic relatedness (average IBD sharing proportion > 0.1875: n=121), non-European ancestry (n=13). This resulted in 2534 genotyped individuals.	Measured within 4 hours of collection using a Hitachi 747 Biochemistry analyser (Roche reagents, modified kinetic Jaffé).	NA

Abbreviations: T2D = type 2 diabetes. §Total genotyped sample size.

Supplementary Table 16 (continued).

Study[REF]	Study Design	N[§]	Study characteristics	Creatinine Measurement	Cystatin measurement
CHS[111, 112]	Prospective population-based	3397	**Study exclusions or disease enrichment**. A total of 1908 persons excluded from the GWAS study sample due to the presence at study baseline of coronary heart disease, congestive heart failure, peripheral vascular disease, valvular heart disease, stroke or transient ischemic attack or lack of available DNA.<br>**Exclusions**. The present report is based upon genotyping results from 3329 Caucasian participants, who were free of clinical cardiovascular disease at baseline, consented to genetic testing, and had DNA available for genotyping. Genotyping was successful in 3291 persons.	Colorimetric method (Ektachem 700, Eastman Kodak).	Particle-enhanced immunonephelometric assay [N Latex Cystatin C, Dade Behring (now Siemens), Deerfield, Ill, USA] with a nephelometer [BNII, Dade Behring (now Siemens)].
CROATIA-KORCULA[113]	Cross-sectional, family-based	888	**Study exclusions or disease enrichment**. None.<br>**Exclusions**. Subjects were excluded if they fulfilled any of the following criteria: genotypic call rate <97%; reported vs. genotypic sex mismatch; unexpectedly low genomic sharing with 1st degree relatives; excess of autosomal heterozygosity; outliers identified by IBS clustering analysis; pregnant women.	Jaffé rate method in plasma.	NA
CROATIA-SPLIT[114]	Population-based	499	**Study exclusions or disease enrichment**. Fasting urine and blood samples were collected from 1012 healthy volunteers aged 18+ from Split on the Dalmatian coast in Croatia in 2008/2011.<br>**Exclusions**. Missing creatinine levels	Jaffé protein compensated method in the serum.	NA
CROATIA-VIS[115, 116]	Cross-sectional, family-based	768	**Study exclusions or disease enrichment**. None.<br>**Exclusions**. Subjects were excluded if they fulfilled any of the following criteria: genotypic call rate <97%, mismatch between reported and genotypic sex, unexpectedly low genomic sharing with first degree relatives, excess autosomal heterozygosity, or outliers identified by IBS clustering analysis. Pregnant women were excluded from the study.	Enzymatic photometric assay using an ADVIA1650 clinical chemistry analyzer (Siemens Healthcare Diagnostics GmbH, Eschborn, Germany)[117] at the Institute for Clinical Chemistry and Laboratory Medicine, Regensburg University Medical Center, Germany.	NA
DESIR[118]	Population-based	731	**Study exclusions or disease enrichment**. Excluded 15 ethnicity outliers.<br>**Exclusions**. Individuals with genotype call rate <0.90, outlying heterozygosity, gender discrepancies, missing clinical data, cryptic relationships, non-European.	Kinetic colorimetry using Jaffé's Method. The assay utilized a Technicon DAX24 automated analyser from Bayer Diagnostics, Puteaux, France or a Specific or a Delta from Konelab, Evry, France.	NA
EGCUT 370K[119, 120]<br><br>EGCUT Omni[119, 120]	Population-based	2700<br><br>9500	**Study exclusions or disease enrichment**. None.<br>**Exclusions**. Missing creatinine levels; genetic outliers; cryptic relatedness (one random member up to 2nd cousins was only included)	Modified Jaffé protein compensated method in the serum.	NA

Abbreviations: T2D = type 2 diabetes. §Total genotyped sample size.

Supplementary Table 16 (continued).

Study[REF]	Study Design	N[§]	Study characteristics	Creatinine Measurement	Cystatin measurement
ERF[121]	Population based family study	2834	**Study exclusions or disease enrichment**. None. **Exclusions**. NA.	Jaffé rate method using a Synchron LX20.	NA
FamHS[122]	Family based	3838	**Study exclusions or disease enrichment**. None. **Exclusions**. Age <18, call rate <98%, pHWE <10E-6, sex mismatch, non-European ancestry.	Thin film adaptation of the amidohydrolase enzymatic method using the Vitros analyzer (Johnson & Johnson Clinical Diagnostics, Inc. Rochester NY 14650).	Immune particle-enhanced turbidimetric (PET) kit (DAKO A/S, Produktionsvej 42, DK-2600 Glostrup, Denmark. Code no. K0071)
FHS[123-125]	Prospective family-based	9300	**Study exclusions or disease enrichment**. None. **Exclusions**. Of the 9,274 participants who underwent genotyping, we made the following exclusions: sample call rate <97% (n=666), genotype heterozygosity > 5 SDs, and ambiguous family data (n=127). This resulted in 8481 genotyped individuals.	Modified kinetic Jaffé reaction.	Particle-enhanced immunonephelometric method, Cystatin C was measured (BNII, Dade-Behring).
GENOA[126-128]	Family-based	1553	**Study exclusions or disease enrichment**. 5 Non-white, 1 Missing Exam. **Exclusions**. For the Affymetrix 6.0 data, we excluded 25 subjects who failed pre-processing, 123 with Contrast QC<0.4, 2 for inconsistent relatedness, 11 identical twins. We re-ran the samples that failed pre-processing or the Contrast QC filter on the Affymetrix 6.0 data along with 50 that had passed. Of these samples, 19 failed genotyping completely, 9 had call rate <0.95, 2 had inconsistent relatedness, and 2 were identical twins. Of the 1509 remaining samples, 346 had no serum creatinine information. Our final data had 1163 samples with genotype and phenotype data.	Compensated rate Jaffé reaction.	NA
HABC	prospective cohort study	1663	**Study exclusions or disease enrichment**. None. **Exclusions**. Sample failure, genotypic sex mismatch, and first-degree relative of an included individual based on genotype data.	Colorimetric technique on a Johnson & Johnson VITROS 950 Chemistry Analyzer (Johnson & Johnson, New Brunswick, N.J., USA) using the enzymatic method.	BNII nephelometer (Dade Behring Inc., Deerfield, Ill., USA) that utilized a particleenhanced immunonephelometric assay (N Latex Cystatin C).
HCS[129]	Population-based	2204	**Study exclusions or disease enrichment**. None. **Exclusions**. Individuals with genotype call rate <0.95, outlying heterozygosity, gender discrepancies, missing clinical data, cryptic relationships, non-European ancestry or missing creatinine measurement.	Siemens Dimension Vista 1500 Intelligent Lab System using a modified Jaffé assay in a NATA accredited lab.	NA
HPFS[130-134]	Nested case-control study of T2D	2487	**Study exclusions or disease enrichment**. All subjects had T2D. **Exclusions**. Of the 2487 subjects with genome-wide scans, 818 (all T2D cases) had creatinine measured.	Modified kinetic Jaffé reaction in plasma.	NA

Abbreviations: T2D = type 2 diabetes. §Total genotyped sample size.

Supplementary Table 16 (continued).

Study[REF]	Study Design#	N*	Study characteristics	Creatinine Measurement	Cystatin measurement
HYPERGENES HTN cases[135]	Cases from a nested case-control study of Hypertension	2125	**Study exclusions or disease enrichment**. We excluded 81 samples with call rate <0.95, 33 with genotypic sex mis-match, 39 duplicated, 94 related subjects, and 13 outliers.<br>**Exclusions**. Of the 1865 subjects with GWA data after QC, 1591 had creatine measured	Kinetic Jaffé reaction.	NA
HYPERGENES HTN controls[135]	Controls from a nested case-control study of Hypertension	1934	**Study exclusions or disease enrichment**. We excluded 62 samples with call rate <0.95, 23 with genotypic sex mismatch, 25 duplicated, 62 related subjects, and 12 outliers.<br>**Exclusions**. Of the 1750 subjects with GWA data after QC, 1662 had creatine measured	Kinetic Jaffé reaction.	NA
INCIPE[136]	Cross-sectional, population based	940	**Study exclusions or disease enrichment**. Excluded were individuals <40 year old.<br>**Exclusions**. Pregnant women	Kinetic rates using the Jaffé method, recalibrated to standardized creatinine determinations obtained at the Cleveland Clinic Research Laboratory.	Immunonephelometric method that used monospecific antisera on an Immage 800 (Beckman Coulter).
INGI-CARLANTINO[137-139]	Isolated population	679	**Study exclusions or disease enrichment**. None.<br>**Exclusions**. We obtained the levels of creatinine of 447 participants; excluded were those with call rate <97%.	Jaffè reaction.	NA
INGI-CILENTO[140-147]	Cross-sectional population-based study of isolated populations with pedigree information	859	**Study exclusions or disease enrichment**. None.<br>**Exclusions**. 38 individuals aged <20 years	Modified kinetic Jaffé reaction.	NA
INGI-FVG[137-139]	Isolated population	1376	**Study exclusions or disease enrichment**. None.<br>**Exclusions**. We obtained the levels of creatinine of 874 participants; excluded were those with call rate <97%.	Jaffé reaction.	NA
INGI-VAL BORBERA[148]	Family Population-based	1665	**Study exclusions or disease enrichment**. None.<br>**Exclusions**. Of the 1665 participants who underwent genotyping, one was excluded because of a sample call rate <95%.	Jaffé reaction.	NA

Abbreviations: T2D = type 2 diabetes. §Total genotyped sample size.

Supplementary Table 16 (continued).

Study[REF]	Study Design	N[§]	Study characteristics	Creatinine Measurement	Cystatin measurement
JUPITER[149, 150]	Randomized, placebo-controlled trial	8782	**Study exclusions or disease enrichment**. Excluded were subjects with LDL-C ≥ 130 mg/dl or CRP < 2 mg/l.<br>**Exclusions**. None	Performed in JUPITER core central laboratories using the Roche Modular Analytics Chemistry System with Roche creatinine reagents (modified Jaffe reaction with rate blanking) (Roche Diagnostics, Township of Branchburg, New Jersey).	NA
KORA-F3[151, 152]<br><br>KORA-F4[151, 152]	Prospective population based	1644<br><br>1814	**Study exclusions or disease enrichment**. None.<br>**Exclusions**. None.	Modified kinetic Jaffe reaction.	Particle-enhanced immunonephelometric method (BNII, Dade-Behring).
MESA[153]	Community-based cohort study	2520	**Study exclusions or disease enrichment**. None.<br>**Exclusions**. NA.	Serum creatinine was measured by rate reflectance spectrophotometry using thin film adaptation of the creatine amidinohydrolase method on the Vitros analyzer (Johnson & Johnson Clinical Diagnostics, Inc., Rochester, NY 14650). The laboratory analytical CV is 2.2%. All creatinine measurements for the MDRD Study were performed at Cleveland Clinic Labs using a CX3 assay. The Vitros analyzer used here was previously calibrated to a CX3 machine with the Cleveland Clinic lab and found the results were nearly identical.	BNII nephelometer (Dade Behring Inc., Deerfield, IL) that utilizes a particle enhanced immunonepholometric assay (N Latex Cystatin-C) 7 on fasting plasma specimens stored at -70°C. The assay is stable over 5 cycles of freeze / thaw. Among 61 healthy individuals with 3 cystatin-C measurements over a 6-month period, the intra-individual coefficient of variation was 7.7%.
MICROS[154-156]	Cross-sectional, population-based study on extended pedigrees	1391	**Study exclusions or disease enrichment**. None.<br>**Exclusions**. Excluded were samples with overall SNP call rate <95%, showing excess of heterozygosity, or being classified as outliers by IBS clustering analysis.	Enzymatic photometric assay using an ADVIA1650 clinical chemistry analyzer (Siemens Healthcare Diagnostics GmbH, Eschborn, Germany).	BN-ProSpec analyzer (Dade Behring, Marburg, Germany) at the Institute for Clinical Chemistry and Laboratory Medicine, Regensburg University Medical Center, Germany.

Abbreviations: T2D = type 2 diabetes. §Total genotyped sample size.

Supplementary Table 16 (continued).

Study[REF]	Study Design	N§	Study characteristics	Creatinine Measurement	Cystatin measurement
NESDA[157, 158]	Longitudinal cohort study	1862	**Study exclusions or disease enrichment**. Individuals were almost all cases with major depression or anxiety disorder (N=1705) and of Western-European ancestry.<br>**Exclusions**. Excluded were ethnic outliers, XO and XXY samples, and samples with a call rate <95%, high genome-wide homo- or heterozygosity, or excess of IBS.	Enzymatic assay from Roche Modular P unit (CREAplus; Roche Diagnostics, Ltd., Lewes, UK). Outliers (>4SD from mean) were excluded.	BNII nephelometer on plasma specimens (N Latex Cystatin C; Dade Behring Inc., Deerfield, IL). Excluded were values >4SD from the mean.
NHS[130, 132, 133, 159, 160]	Nested case-control study of T2D	3286	**Study exclusions or disease enrichment**. All individuals had T2D.<br>**Exclusions**. Of the 3286 subjects with GWA data, 784 had creatinine measured.	Modified kinetic Jaffé reaction in plasma. Creatinine values were not normalized to the Cleveland Clinic standard.	NA
NSPHS[161, 162]	cross-sectional, family-based	565	**Study exclusions or disease enrichment**. None.<br>**Exclusions**. Subjects were excluded if they fulfilled any of the following criteria: genotypic call rate <97%, mismatch between reported and genotypic sex, unexpectedly low genomic sharing with first degree relatives, excess of autosomal heterozygosity, or outliers identified by IBS clustering analysis.	Enzymatic photometric assay in plasma using an ADVIA1650 clinical chemistry analyzer (Siemens Healthcare Diagnostics GmbH, Eschborn, Germany).	NA
OGP-TALANA[163-165]	Isolated population	1376	**Study exclusions or disease enrichment**. None.<br>**Exclusions**. We obtained the levels of creatinine of 874 participants; exclusions were samples with call rate<97%.	Jaffé reaction.	NA
ORCADES[166]	cross-sectional, family-based	704	**Study exclusions or disease enrichment**. None.<br>**Exclusions**. Subjects were excluded if they fulfilled any of the following criteria: genotypic call rate <97%, mismatch between reported and genotypic sex, unexpectedly low genomic sharing with first degree relatives, excess autosomal heterozygosity, or outliers identified by IBS clustering analysis.	Enzymatic photometric assay in plasma using an ADVIA1650 clinical chemistry analyzer (Siemens Healthcare Diagnostics GmbH, Eschborn, Germany)[117] at the Institute for Clinical Chemistry and Laboratory Medicine, Regensburg University Medical Center, Germany.	NA
POPGEN[167]	Prospective population-based	1163	**Study exclusions or disease enrichment**. None.<br>**Exclusions**. Samples with > 5% missing genotypes, showing excess genetic dissimilarity to the remaining subjects, or with evidence for a cryptic relatedness to other study participants were removed. These quality control measures left 1241 control samples for inclusion in the study. Of them, 1163 had serum creatinine available. All sex assignments could be verified by reference to the proportion of heterozygous SNPs on the X chromosome.	Enzymatic in vitro assay (CREAplus, Cobas®, Roche Diagnostics, Indianapolis, IN)	NA
PREVEND[168]	population based	3634	**Study exclusions or disease enrichment**. Subjects were aged 28 to 75 years and were enriched for microalbuminuria.<br>**Exclusions**. NA	NA	BNII nephelometer (Dade Behring Inc.).

Abbreviations: T2D = type 2 diabetes. §Total genotyped sample size.

Supplementary Table 16 (continued).

Study[REF]	Study Design	N§	Study characteristics	Creatinine Measurement	Cystatin measurement
PROSPER-PHASE[169-171]	randomized placebo controlled trial	5763	**Study exclusions or disease enrichment**. Subjects were included when they had had a vascular event or were at increased risk of vascular disease; age: 70-82.<br>**Exclusions**. After QC (sample rate <97.5%, genotype heterozygosity, ambigous family data), 5244 samples were available for analysis.	Measured at central laboratories, one in each of the three participating countries.	NA
RS-I[172-174]	Prospective population based study	5974	**Study exclusions or disease enrichment**. NA.<br>**Exclusions**. Excluded were samples with call rate < 97.5%, excess autosomal heterozygosity >0.336 (~FDR <0.1%), mismatch between called and phenotypic gender, or outliers identified by the IBS clustering analysis with >3 SDs from population mean or IBS probabilities >97%.	Modified kinetic Jaffé reaction.	NA
RS-II[172-174]	Prospective population based study	1895	**Study exclusions or disease enrichment**. NA.<br>**Exclusions**. Excluded were samples with call-rate <97.5%, excess of autosomal heterozygosity >0.336 (~FDR<0.1%), mismatch between called and phenotypic gender, and outliers identified by the IBS clustering analysis with >3 SDs from population mean or IBS probabilities >97%.	Modified kinetic Jaffé reaction.	NA
SAPALDIA	population based	1444	**Study exclusions or disease enrichment**. NA.<br>**Exclusions**. Excluded were subjects with cryptic relatedness and call rate <95%.	Jaffé reaction (Roche) and calibrated to the Roche enzymatic gold standard reference yielding slightly lower serum creatinine measurements than the Cleveland Clinic Jaffé reaction.	NA
SHIP[175, 176]	Prospective population-based	4105	**Study exclusions or disease enrichment**. None.<br>**Exclusions**. Excluded were samples with call rate <92%, duplicate samples (by IBS estimation), individuals with reported/genotyped gender mismatch.	Jaffè method. (A blood sample was drawn from the cubital vein in the supine position - the participants were non-fasting due to the duration of the cumulative examinations, 4-6 hours in total).	Siemens N Latex Cystatin C assay, a particle-enhanced nephelometric immunoassay, on the BN ProSpec® System.
SHIP-TREND	Prospective population-based	988	**Study exclusions or disease enrichment**. Excluded were individuals with no genotype and those with known T2D.<br>**Exclusions**. Excluded were samples with call rate <94%, duplicate samples (by IBS estimation), individuals with reported/genotyped gender mismatch.	Jaffé method.	Dimension Vista® System, CYSC Flex® reagent cartridge, SIEMENS, Eschborn, Germany
SORBS[177-179]	Population-based	1097	**Study exclusions or disease enrichment**. None.<br>**Exclusions**. Sample call rate<0.94; Ethnic outliers; duplicates; gender mismatch; IBS>0.2	Kinetic enzymatic method.	NA

Abbreviations: T2D = type 2 diabetes. §Total genotyped sample size.

Supplementary Table 16 (continued).

Study[REF]	Study Design	N[§]	Study characteristics	Creatinine Measurement	Cystatin measurement
WGHS[180]	Prospective population based	21,940	**Study exclusions or disease enrichment**. Excluded were individuals of non-European ancestry.<br>**Exclusions**. Samples with <98% successful SNP calls were excluded. Data had been collected on 21940 individuals had successful genotype information and verified European ancestry at the time of the analysis.	Rate-blanked method based on the Jaffé reaction using Roche Diagnostics reagents with reproducibility of 3.67% and 1.60% at concentrations of 1.17 and 6.40 mg/dL, respectively.	NA
YFS	Population based	2023	**Study exclusions or disease enrichment**. NA.<br>**Exclusions**. None.	Jaffé method (picric acid; Olympus Diagnostica GmbH) from frozen plasma samples in 2007.	NA

Abbreviations: T2D = type 2 diabetes. §Total genotyped sample size.

Supplementary Table 17. Study information, replication. Extensive study names are reported in the Acknowledgements section.

Study Name[REF]	Study Design	N§	Study characteristics	Creatinine Measurement	Cystatin measurement
Bus Santé[181, 182]	Cross-sectional population-based	5589	**Study exclusions or disease enrichment**. None.<br>**Exclusions**. Unsuccessful genotyping (N=106). Analyses were restricted to Caucasians, defined as self-reported citizenship corresponding to South / North America, Europe, and Australia regions (N=4671). Creatinine was available in 4408 participants.	Modified kinetic Jaffé reaction (Abbott Architect).	NA
EGCUT replic[120]	Population-based	8576	**Study exclusions or disease enrichment**. None.<br>**Exclusions**. Sample call rate <95% (N=279), genotype heterozygosity >5 SDs and ambiguous family data (N=264), gender mismatch (N=241).	Modified kinetic Jaffé reaction.	Immune turbidimetric method.
ESTHER[183-185]	Prospective cohort study	3490	**Study exclusions or disease enrichment**. Inclusion criteria: age ≥50 and good knowledge of German language.<br>**Exclusions**. Samples with insufficient amount of DNA were not genotyped.	Modified kinetic Jaffé reaction.	NA
GENDIAN[186, 187]	Cohort study of T2D complications	1026	**Study exclusions or disease enrichment**. After exclusion of N=53 subjects due to subject QC, excluded were patients with ESRD (N=438) or advanced, histologically proven diabetic nephropathy (N=84) or missing phenotype (N=1).<br>**Exclusions**: call-rate<95% (N=22); relatedness and duplicates (N=11); gender mismatch (N=16); ethnicity check (N=4).	Enzymatic assay.	Dade Behring assay (BNII)
GHS-I[188, 189]<br><br>GHS-II[188, 189]	Population-based	3422<br><br>1438	**Study exclusions or disease enrichment**. Excluded if age <35 and >74.<br>**Exclusions**: N=426 based on a call-rate <97%, a rate of heterorzygosity of 3 SDs away from the mean, disagreement between reported and genotypic sex, estimated IBD >0.25, IBS based principal components. This resulted in 2996 genotyped individuals.	Modified kinetic Jaffé reaction (Abbott).	NA
GSK[190-192]	Case-control study	1776	**Study exclusions or disease enrichment**. Cases were patients with unipolar recurrent depression; exclusion criteria: presence of manic or hypomanic episodes, mood incongruent psychotic symptoms, lifetime diagnosis of drug abuse and depressive symptoms secondary to alcohol or substance abuse or dependence or to a medical illness or medication. Controls: exclusion criteria: anxiety and affective disorders.<br>**Exclusions**: call-rate<98% (N=2).	Standard clinical chemistry assays from Roche Hitachi.	NA
HRS	Population-based Longitudinal	12,507	**Study exclusions or disease enrichment**. Non-European participants were excluded.<br>**Exclusions**. Sample call-rate<98%, gender discrepancy, unexpected duplicates and/or relatives with KC>1/32, missing cystatin C data.	NA	See note (1)
KORAF3 non-GWAS[151, 152]<br><br>KORAF4 non-GWAS[151, 152]	Prospective Population based	1498<br><br>1202	**Study exclusions or disease enrichment**. None.<br>**Exclusions**. None.	Modified kinetic Jaffé reaction.	Particle-enhanced immunonephelometric method (BNII, Dade-Behring).

§ Total genotyped sample size.

(1) Measured from dried blood spots (DBS) obtained during a face-to-face interview with the respondent at the same time as saliva collection. Blood was taken by pricking the participant's finger with a sterile lancet after cleansing the finger with an alcohol swab. Droplets of blood were extracted from the finger and directly placed on specially treated filter paper. Cystatin C for was assayed at the University of Vermont in 2006 and 2008. Serum equivalent values were calculated using a formula derived from a validation study done in conjunction with the USC/UCLA Center on Biodemography and Population Health.

Supplementary Table 17 (continued).

Study Name[REF]	Study Design	N§	Study characteristics	Creatinine Measurement	Cystatin measurement
IPM[193]	Hospital-based	1747	**Study exclusions or disease enrichment**. None.<br>**Exclusions**. None.	Colorimetric method in (CPT82565) performed by the New York State / CLIA Clinical Chemistry Laboratory at Mount Sinai Medical Center.	NA
LURIC[194]	Case control	3061	**Study exclusions or disease enrichment**. Inclusion criteria: Caucasian, availability of coronary angiogram, stable condition except acute coronary syndrome. Exclusion criteria: any chronic disease other than cardiovascular, any acute illnesss other than ACS, any malignancy in the previous five years.<br>**Exclusions**. Sample call-rate<95%, ambiguous sex, duplicates, relatedness. This resulted in 3061 genotyped individuals.	Jaffé method (CREA/Hitachi 717).	N LATEX Cystatin C/ Behring nephelometer II.
OGP[163-165]	Population-based study with pedigree information	9554	**Study exclusions or disease enrichment**. None.<br>**Exclusions**. NA.	Modified kinetic Jaffé reaction or enzymatic reaction.	NA
SAPHIR[195, 196]	Healthy working population	1726	**Study exclusions or disease enrichment**. None.<br>**Exclusions**. None.	Modified kinetic Jaffé reaction (CREA®, Roche Diagnostics GmbH, Mannheim, Germany).	NA
SKIPOGH[197]	cross-sectional family-based population-based	941	**Study exclusions or disease enrichment**. None.<br>**Exclusions**. Excluded were 71 participants with call-rate <90%, resulting in 870 genotyped individuals.	IDMS-traceable Jaffé kinetic compensated method.	NA
Vanderbilt Omni1<br><br>Vanderbilt Omni5	Practice-based cohort	5184<br><br>2005	**Study exclusions or disease enrichment**. Samples chosen based on being a case or control for one of 31 pharmacogenetic analyses. **Exclusions**. Excluded individuals of non-white ancestry in the electronic medical record. Also excluded any lab measurements of individuals after initiation of dialysis or a kidney transplant. The median outpatient creatinine value was chosen.	Jaffé reaction.	NA
Vanderbilt 660W	Practice-based cohort	3021	**Study exclusions or disease enrichment**. Samples chosen for normal cardiac conduction, meaning that at some point in time they had a normal electrocardiogram without the presence of heart disease, arrhythmias, or electro-cardiographically-active medications.<br>**Exclusions**. Children (age<18) and individuals of non-white ancestry in the electronic medical record. Also excluded any lab measurements from individuals after initiation of dialysis or a kidney transplant. The median outpatient creatinine value was chosen. At some point in their electronic medical record, the patients were absent of heart disease, but could later develop it.	Jaffé reaction.	NA

§ Total genotyped sample size.

Supplementary Table 18. Genotyping information, discovery studies.

Study name	Array type	Genotype calling	QC filters genotyped SNPs used for imputation‡	No. of SNPs used for imputation	Imputation software	Imputation Backbone (NCBI build)	Filtering of imputed genotypes†	Data management and statistical analysis
3C	Illumina Human610-Quad	Illumina BeadStudio	call rate < 98%, pHWE<10e-6, MAF<1%	492,897	MACH	1000 Genomes - CEU - Dec 2010 (build 37)	none	R, ProbABEL
Advance	Affymetrix 5.0 Affymetrix 6.0	Affymetrix	Affymetrix 5.0: call rate < 96% (<99% if MAF < 5%); Affymetrix 6.0: call rate < 97% (<99% if MAF<5%)	876,688	IMPUTE 2 v2.1.2	1000 Genomes - CEU Pilot - Jun 2010 plus HapMap3 rel. 2 (all available haplotypes) – Feb 2009 (build 36)	imputation info<0.5	SNPTEST
AGES	Illumina Hu370CNV	Illumina	pHWE<1e-6, call rate<97%, mishap p<1e-9, MAF<1%, SNPs not in Hapmap or with strand issues when merging with Hapmap	329,804	MACH v1.0.16	HapMap rel. 22 (build 36)	none	R, ProbABEL, Linear and Logistic Regression
Amish	Affymetrix 500K	BRLMM	MAF<1%, non-HapMap, call rate < 95%, pHWE<10e-6	338,598	MACH v1.0.15	HapMap rel. 22 - phased CEU haplotypes (build 36)	none	Measured genotype accounting for polygenic component
ARIC	Affy 6.0	Birdseed	call rate <95%, MAF<1%, pHWE <10e-5	669,450	MACH v1.0.16	HapMap rel. 22 (build 36)	none	R, ProbABEL, PLINK
ASPS	Illumina Human610-Quad BeadChip	Illumina	pHWE<1e-6, call rate < 98%, mishap p<1e-9, MAF<1%, Mendelian errors>100, SNPs not in Hapmap or with strand issues when merging with Hapmap	550,635	MACH v1.0.15	HapMap rel. 22 (build 36)	none	R, linear and logistic fixed effects model
AUSTWIN	Illumina370, iLLumina610	BeadStudio-gencall v3.0	call rate < 95%, pHWE<1e-6	271,069	MACH v1.0.15	HapMap rel. 22 - phased CEU haplotypes (build 36)	Rsq<0.3	Stata, SPSS
BLSA	Illumina Infinium HumanHap 550K	BeadStudio	call rate <99%, MAF <1%, pHWE<10e-4	501,764	MACH v1.0.15	HapMap rel. 21 - phased CEU haplotypes (build 35)	MAF<0.01, r2hat < 0.3	SAS, MERLIN, R

Supplementary Table 18 (continued).

Study name	Array type	Genotype calling	QC filters genotyped SNPs used for imputation‡	No. of SNPs used for imputation	Imputation software	Imputation Backbone (NCBI build)	Filtering of imputed genotypes†	Data management and statistical analysis
BMES	Illumina 670K-Quad	CHIAMO	call rate <95%, pHWE<10e-6, MAF<1%, genotype discrepancies in > 2.5% of 1356 samples independently genotyped for the Illumina 610K array	501,910	MACH v1.0.16	HapMap rel. 22 - phased CEU haplotypes (build 36.1)	none	Plink, mach2dat, SAS
CHS	Illumina 370CNV	Illumina BeadStudio	call rate<97%, heterozygotes=0, pHWE<10e-5, SNP not in HapMap	306,655	BimBam v0.99	HapMap CEU rel. 22 (build 36)	dosage variance < 0.01	R, linear and logistic regression, robust SE estimates
CROATIA-KORCULA	Illumina Infinium HumanCNV370 v1 SNP bead microarrays	BeadStudio	MAF<1%, pHWE<1e-6, call rate < 98%	317,896	MACH v1.0.16	HapMap rel. 22 (build 36)	none	R, GenABEL, ProbABEL;
CROATIA-SPLIT	Illumina HAP370CNV	Illumina	call rate <98%, pHWE<10e-10	330,997	MACH v1.0.15	HapMap rel. 22 - phased CEU haplotypes (build 36)	none	R (GenABEL, ProABEL)
CROATIA-VIS	Illumina HumanHap300 beadchip	BeadStudio	MAF≤1% , pHWE ≤1e-6 , call rate ≤ 98%	305,068	MACH v1.0.15	HapMap rel. 22 (build 36)	none	R, GenABEL, ProbABEL;
DESIR	Illumina Human CNV370-Duo Array and Illumina HAP300 array	Illumina Beadstation Genotyping Solution	call rate < 95%<br>p HWE < 1e-4	291,609	IMPUTE v0.3.2 (genotyped SNPs used where available)	HapMap rel. 22 - phased CEU haplotypes (build 36)	none	SNPTEST v2.2.0
EGCUT 370K	Illumina 370CNV; Illumina OmniExpress	GenCall GenomeStudio	call rate <95%, pHWE<10e-6, MAF<1%	320784; 622800	IMPUTE v1.0	HapMap 2 CEU rel. 22	none	PLINK
EGCUT Omni	Illumina 370CNV; Illumina OmniExpress	GenCall GenomeStudio	call rate <95%, pHWE<10e-6, MAF<1%	320784; 622800	IMPUTE v1.0	HapMap 2 CEU rel. 22	none	PLINK
ERF	Illumina 6K/318K/380K, Affy 250K	BeadStudio (Affymetrix)	pHWE<1e-6, call rate<98%, MAF<1%, gender mismatch, excess heterozygosity	487,573	MACH v1.0.15	HapMap rel. 22 (build 36)	none	R, GenABEL, ProbABEL (linear mixed effect models)
FamHS	Illumina 550K, Illumina 610K, and Illumina 1M	BeadStudio-GenCall v3.0	call rate <98%, pHWE<10e-6, MAF<1%	499,979	MACH v1.0.16	HapMap rel. 22 - phased CEU haplotypes (build 36)	none	R (quantitative traits), SAS (qualitative trait)

Supplementary Table 18 (continued).

Study name	Array type	Genotype calling	QC filters genotyped SNPs used for imputation‡	No. of SNPs used for imputation	Imputation software	Imputation Backbone (NCBI build)	Filtering of imputed genotypes†	Data management and statistical analysis
FHS	Affymetrix 500K Affymetrix 50K Supplementary	Affymetrix	call rate <95%, pHWE<10e-6	503,526	MACH v1.0.15	HapMap rel. 22 - phased CEU haplotypes (build 36)	none	R
GENOA	Affymetrix 6.0 (primary), Illumina 610-Quad, Illumina 660-Quad, Illumina 1M-Duo	Birdseed (Affymetrix data), Genome Studio (Illumina data)	call rate < 95%, pHWE < 0.001, MAF<1%	1,233,495 (because of the different platforms, some SNPs may have had many missings)	MACH v1.0.16	HapMap CEU rel. 22 (build 36)	none	R, multic and GEE
HABC	Illumina 1M	BeadStudio v3.3.7	MAF<1%, call rate < 97%, pHWE<1e-6	914,263	MACH v1.0.16	HapMap CEU rel. 22 (build 36)	None	R, linear regression and logistic regression
HCS	Illumina 610K-Quad	Illumina	call rate <95%, pHWE<10e-6, MAF<1%	513,977	MACH v1.0.16	HapMap rel. 24 - phased CEU haplotypes (build 36.1)	MAF<0.01, Rsq<0.3	plink, SAS
HPFS	Affymetrix Genome-Wide Human 6.0 array	Birdseed	call rate <97%, pHWE<10e-4, MAF <0.02, more than 1 discordance over 29 replicates, significant plate associations	607,569 (autosomal)	MACH v1.0.15	HapMap rel. 22 - phased CEU haplotypes (build 36)	none	ProbABEL (R), SAS 9.0, PLINK
HYPERGENES HTN cases	Illumina 1M Duo	Illumina	call rate <99%, pHWE<10e-8	882,935	MACH v1.0	HapMap rel. 22 - phased CEU haplotypes (build 36)	Rsq<0.3	PLINK, R
HYPERGENES HTN ctrls	Illumina 1M Duo	Illumina	call rate <99%, pHWE<10e-8	882,935	MACH v1.0	HapMap rel. 22 - phased CEU haplotypes (build 36)	Rsq<0.3	PLINK, R
INCIPE	Illumina	Illumina	call rate <95%, pHWE<10e-6	635,646	IMPUTE 2	HapMap rel. 22 - phased CEU haplotypes (build 36)	none	R
INGI-CARLANTINO	Illumina 370K	BeadStudio	MAF<5%, call rate < 90%, pHWE < 0.05	374,498	MACH	HapMap rel. 22 (build 36)	Rsq<0.3 and <5 copies of the rare allele.	R, GenABEL, mmscore
INGI-CILENTO	370 K Illumina	Illumina	call rate < 95%, SNPs not in Hapmap	285,674	MACH v1.0.15	HapMap rel. 22 (build 36)	none	R, linear model, GenABEL, ProbABEL (mmscore)
INGI-FVG	Illumina 370K	BeadStudio	MAF<5%, call rate < 90%, pHWE < 0.05	374,498	MACH	HapMap rel. 22 (build 36)	Rsq<0.3 and <5 copies of the rare allele.	R, GenABEL, mmscore

Supplementary Table 18 (continued).

Study name	Array type	Genotype calling	QC filters genotyped SNPs used for imputation‡	No. of SNPs used for imputation	Imputation software	Imputation Backbone (NCBI build)	Filtering of imputed genotypes†	Data management and statistical analysis
INGI-VAL BORBERA	Illumina SNP array 370K - HumanCNV370 -Quadv3	BeadStudio	pHWE<1e-4, call rate < 90%, MAF<1%	324,319	MACH	HapMap rel. 22 (build 36)	none	R
JUPITER	Omni 1M Quad	Illumina	sample call rate <98%, call rate < 90%, pHWE<1e-6	740,416	MACH v1.0	1000 Genomes - CEU - Pilot - Jun 2010	none	R, probABEL
KORA-F3	Affymetrix 500K	BRLMM	per-chip call rate <93%, MAF <5%,discrepancy for one of the 50 SNPs common on both chips, gender mismatch	380,407	MACH	HapMap rel. 22 (build 35)	none	MACH2QTL, PROBABEL, R, VISUAL BASIC
KORA-F4	Affymetrix 6.0	BRLMM	per-chip call rate <93%, per SNP call rate <93%, MAF<1%, gender mismatch	629,893	MACH	HapMap rel. 22 (build 36)	none	MACH2QTL, PROBABEL, R, VISUAL BASIC
MESA	Affymetrix Genome-Wide Human SNP Array 6.0	Birdseed v2	call rate < 95%, MAF≤1%	897,979	IMPUTE v2.1.0	HapMap rel. 22 - phased CEU haplotypes (build 36)	none	PLINK
MICROS	Illumina Infinium HumanHap300 v2 SNP bead microarrays	BeadStudio	call rate <98%, MAF<1%, pHWE<10e-6	292,917	MACH v1.0.16	HapMap rel. 22 (build 36)	none	R, GenABEL, ProbABEL;
NESDA	Perlegen 600K	Perlegen	call rate <95%, MAF<1%, >5% genotype mismatches, >5% Mendelian errors, unknown SNP location	435,291	IMPUTE v0.4.2	HapMap rel. 24 - phased CEU haplotypes (build 36)	none	SNPTEST v2.2.0, R
NHS	Affymetrix Genome-Wide Human 6.0 array	Birdseed	call rate <97%, pHWE<10e-4, MAF <2%, >1 discordance/12 replicates, significant plate associations	606,626 (autosomal)	MACH v1.0.15	HapMap rel. 22 - phased CEU haplotypes (build 36)	none	R, ProbABEL, SAS 9.0, PLINK
NSPHS	Illumina 300K	BeadStudio	MAF≤1%, pHWE≤1e-5 , call rate ≤ 97%	318,049	MACH v1.0.15	HapMap rel. 22 (build 36)	none	R, GenABEL, ProbABEL
OGP-TALANA	Affymetrix500k	Affymetrix	call rate <95%, pHWE<10e-6	329,122	MACH v1.0.16	HapMap rel. 22 - phased CEU haplotypes (build 36)	Rsq<0.3	R, GenABEL, ProbABEL (mmscore)
ORCADES	Illumina 300K	BeadStudio	MAF≤1%, pHWE ≤ 1e-6, call-rate ≤ 98%	306,207	MACH v1.0.15	HapMap rel. 22 (build 36)	none	R, GenABEL, ProbABEL

Supplementary Table 18 (continued).

Study name	Array type	Genotype calling	QC filters genotyped SNPs used for imputation‡	No. of SNPs used for imputation	Imputation software	Imputation Backbone (NCBI build)	Filtering of imputed genotypes†	Data management and statistical analysis
POPGEN	Affymetrix 6.0	Birdseed v2	sample call rate < 0.90, call rate < 0.95, pHWE<1e-4, MAF<1%	709,003	MACH v1.0.16	HapMap rel. 22 - phased CEU haplotypes (build 36)	none	PLINK, R
PREVEND	Illumina CytoSNP12 v2	GenomeStudio	call rate < 95%, pHWE<1e-5	232,571		HapMap rel. 22 - phased CEU haplotypes (build 36)	none	STATA
PROSPER-PHASE	Illumina 660K	Illumina	call rate < 97,5%	557	MACH v1.0.15	HapMap rel. 22 (build 36)	none	R
RS-I	Version 3 Illumina Infinium II HumanHap550	BeadStudio	pHWE<1e-5, call rate<90%, MAF<1%, Mendelian errors > 100, SNPs not in Hapmap or strandedness issues merging with Hapmap	491,875	MACH	HapMap rel. 22 (build 36)	none	ProbABEL
RS-II	Version 3 Illumina Infinium II HumanHap550	BeadStudio	pHWE<1e-5, call rate<90%, MAF<1%, Mendelian errors>100, SNPs not in Hapmap or strandedness issues merging with Hapmap	495,478	MACH	HapMap rel. 22 (build 36)	none	ProbABEL
SAPALDIA	Illumina Human610-Quad BeadChip	Gencall	none	567,589	MACH v1.0.16	HapMap rel. 22 - phased CEU haplotypes (build 36)	none	ProbABEL, STATA v11
SHIP	Affymetrix 6.0	Affymetrix Birdseed2	none	869,224	IMPUTE v0.5.0	HapMap rel. 22 (build 36)	none	SNPTEST v1.1.5, QUICKTEST v0.94, R, InforSense, InterSystems Caché,
SHIP-TREND	Illumina Human Omni 2.5	Illumina	pHWE ≤ 1e-4, call rate ≤ 0.9, monomorphic SNPs	1,782,967	IMPUTE v2.1.2.3	HapMap rel. 22 - phased CEU haplotypes (build 36)	duplicated rsID with different positions	QUICKTEST v0.95, R, InforSense, InterSystems Caché

Supplementary Table 18 (continued).

Study name	Array type	Genotype calling	QC filters genotyped SNPs used for imputation‡	No. of SNPs used for imputation	Imputation software	Imputation Backbone (NCBI build)	Filtering of imputed genotypes†	Data management and statistical analysis
SORBS	500K Affymetrix GeneChip (250K Sty and 250K Nsp arrays, Affymetrix, Inc) and Affymetrix Genome-Wide Human SNP Array 6.0	Microarray Core Facility of the Interdisciplinary Centre for Clinical Research, University of Leipzig, Germany and ATLAS Biolabs GmbH, Berlin, Germany	MAF<1%, pHWE<1e-4, call rate < 95%,	378,513	IMPUTE	HapMap CEU rel. 21 (build 35)	proper_info ≤ 0.4	ProbAbel with robust variance option to account for remaining relatedness
WGHS	Illumina HumanHap300 Duo "plus"	BeadStudio v3.1	pHWE<1e-6, call rate < 98%, MAF<1%	331,993	MACH v1.0.15	HapMap rel. 22 (build 36)	none	PLINK, R, ProbAbel
YFS	Illumina 670k custom	Illuminus	call rate <95%, pHWE<10e-6	546,674	IMPUTE 2.1.2	1000 Genomes - Jun 2011 phased haplotypes	none	SAS

‡QC filters for genotyped SNPs used for imputation (SNPs satisfying the filter were excluded).

†Filtering of imputed genotypes (SNPs satisfying the filter were excluded).

Supplementary Table 19. Genotyping platform – *in silico* replication studies.

Study Name	Array type	Genotype calling	QC filters for genotyped SNPs used for imputation	No of SNPs used for imputation	Imputation	Imputation Backbone (NCBI build)	Filtering of imputed genotypes	Data management and statistical analysis
EGCUT-replic	Illumina OmniExpress	Genome Studio (GenCall)	call rate<95%, pHWE<10e-6, MAF<1%	616,063	IMPUTE v2.2	HapMap rel. 22 - phased CEU haplotypes (build 36)	none	Plink, SNPTEST 2, R
GENDIAN	Affymetrix Genome-Wide Human SNP Array 6.0	Birdseed (BRLMM)	N=126,259 SNPs (chr 1-chr22, chr X) were excluded from imputation by SNP quality control.	776,075	Mach 1.0.18.c MiniMac 2012-10-09	GIANT ALL 1000 Genomes v3 ref panel GRCh build 37	none	R
GSK cases/controls	Illumina 550 K	Illumina	call rate <98%, pHWE<10e-5	522,008	IMPUTE v2	CEU in HapMap3 and 1000 Genomes Pilot 1	none	R, Plink
Gutenberg Health Study	Affymetrix 6.0	Birdseed	call rate<95%, MAF<1%, pHWE<10e-4	662,405	IMPUTE v2.1.0	HapMap rel. 24 (build 36)	none	R
HRS	Illumina Omni2.5 Beadchip	Genome Studio v2011.2	call rate <98%, pHWE<10e-4	551,936	MACH v1.0.16	HapMap rel. 22 - phased CEU haplotypes (build 36)	none	SAS, R
IPM_EA_Affy	Affymetrix 6.0	Birdseed	sample call rate <95%, SNP call rate <95%, pHWE<1e-4, MAF<1%	711,270	IMPUTE 2	Phase I integrated variant set release (v3) (NCBI build 37)	none	SNPTEST 2, R
IPM_EA_Illu	Illumina Human OmniExpress Exome-8v1	Genome Studio	sample call rate <99%, SNP call rate <95%, pHWE<5e-5, MAF<1%	865,711	IMPUTE 2	Phase I integrated variant set release (v3) (NCBI build 37)	none	SNPTEST 2, R
LURIC	Affymetrix 6.0	Birdseed v2	call rate <98%, pHWE<10e-4	686,195	MACH	HapMap rel. 22 - phased CEU haplotypes (build 36)	none	QUICKTEST
Vanderbilt Omni1	Illumina HumanOmni1-Quad	BeadStudio	call-rate<98%, IBD (Z0<0.8), Mendel errors >0, Duplicate concordance <100%	946,523	IMPUTE v2.3.0	1000 Genomes Phase 1 integrated v3	Genotype Likelihood <0.9	Plink, R
Vanderbilt Omni5	Illumina HumanOmni5-Quad	BeadStudio	call rate <98%, IBD (Z0<0.8), Mendel errors >0, Duplicate concordance <100%	3,819,154	IMPUTE v2.3.0	1000 Genomes Phase 1 integrated v3	Genotype Likelihood <0.9	Plink, R
Vanderbilt 660W	Illumina Human660W-Quad	BeadStudio	call rate <98%, IBD (Z0<0.8), Mendel errors >0, Duplicate concordance <100%	530,014	IMPUTE v2.3.0	1000 Genomes Phase 1 integrated v3	Genotype Likelihood <0.9	Plink, R

Supplementary Table 20. Genotyping information: *de novo* replication studies.

Study Name	Bus Santé	ESTHER	KORA **F3** and **F4**	OGP	SAPHIR	SKIPOGH
**Genotyping platform**	LGC Genomics SNP-line, using KASP™ Chemistry and 1536-well plates	LGC Genomics SNP-line, using KASP™ Chemistry	MALDITOF MS, Bruker Daltonik GmbH, Leipzig, Germany	LGC Genomics SNP-line, using KASP™ Chemistry	Sequenom platform	LGC Genomics SNP-line, using KASP™ Chemistry and 1536-well plates
**Amount of DNA used per SNP** (in ng)	DNA amplified, but typically 5 -7.5 ng of gDNA per genotype	3.75 ng	1 ng	5 ng	15 ng	5 -7.5 ng of gDNA per genotype
**Genotyping method**	See note[1]	See note[1]	iPlex Gold	See note[1]	Mass ARRAY Analyzer 4 system	See note[1]
**No. of duplicates and concordance per SNP (provided per individual SNP)**	Not duplicated	LGC Genomics does not add duplicates. The data for each SNP represents one reaction per sample.	At least 15% duplicate genotyping per SNP. Concordance≥95%, median = 100%	LGC Genomics does not add duplicates. The data for each SNP represents one reaction per sample.	70 duplicates; of 46 SNPs genotyped, 44 had a concordance of 100%, 2 had 1 discordant sample each	29 samples genotyped in duplicate. SNP concordance varied from 86% to 100%.
**Number attempted / number genotyped samples per individual SNP**	The genotyping call rate (%) was typically between 0.95-0.97	Call-rate range 0.98-1	NA	Allele call rate 0.99	46 SNPs were genotyped: mean call-rate = 99.3% (min = 98.15%, max = 99.65%)	Median SNP call-rate = 97.2% (min =94.5%, max = 99.5%)
**Other QC laboratory-specific indices**	See note[2]	none indicated by the lab	NA	none indicated by the lab	automatic calculation of the HWE, comparision of the obtained genotypes with HapMap Data	See note[2]

[1] Genotyped using KASPar (Kompetitive Alelle Specific PCR) v4.0 after whole genome amplification by primer extension preamplification (PEP) using thermostable DNA polymerases.

[2] All assays have been validated on an in-house DNA panel (44 random Caucasian DNA samples). All sample plates genotyped include at least two negative controls. ie. blank/water controls. All genotyping data is initially generated by an automated algorithm (genotype calling based upon recorded fluorescence values). All genotyping data is manually checked and verified by no less than two experienced scientists at LGC genomics.

Supplementary Table 21. Morpholino (MO) sequences.

Target gene	Splice or ATG	MO sequence
A1cf	ATG	5' CCCCACATTTTTGATTGGTTTCCAT 3'
Dpep	ATG	5' AATCTTGACCCATTCCATCATCACC 3'
Kbtbd2	ATG	5' TGTTCGTATCCCATGAGTTTTCAAC 3'
Nfatc1	Splice	5' CGCATCTGTAAGGTACAATCACATT 3'
Nfkb1	ATG	5' TCCTCGCCAGCCATGATTCCTTTGC 3'
Ptpro	ATG	5' GATCGCACTCTTTGATTCTCGGCGT 3'
Skila	Splice	5' TTGCCCTGCAAACACACATACACAC 3'
Skilb	Splice	5' CCCGGATGACTGAAACAAGTCAAAA 3'
Sypl2a	Splice	5' TTGAAATGTGGTTGTTTATACCTGA 3'
Sypl2b	Splice	5' AGACTCTTTAATGAGGTTTACCTAA 3'
Tspan9a	Splice	5' GTAGGAGTGGCAAAACTTACGCTCA 3'
Tspan9b	ATG	5' GCACAGGCATCCACGAGCCATCTTC 3'
Uncx	Splice	5' ATCCCCCGAATCTATGTAAGAAACA 3'
Wnt7aa	ATG	5' TCCAGCGGCGCGTTTTCCTGCTCAT 3'

Supplementary Note.

Study specific acknowledgements and funding sources for participating studies, alphabetical order.

3C. *Three-City Study*. The work was made possible by the participation of the control subjects, the patients, and their families. We thank Dr. Anne Boland (CNG) for her technical help in preparing the DNA samples for analyses. This work was supported by the National Foundation for Alzheimer’s disease and related disorders, the Institut Pasteur de Lille and the Centre National de Génotypage. The 3C Study was performed as part of a collaboration between the Institut National de la Santé et de la Recherche Médicale (Inserm), the Victor Segalen Bordeaux II University and Sanofi-Synthélabo. The Fondation pour la Recherche Médicale funded the preparation and initiation of the study. The 3C Study was also funded by the Caisse Nationale Maladie des Travailleurs Salariés, Direction Générale de la Santé, MGEN, Institut de la Longévité, Agence Française de Sécurité Sanitaire des Produits de Santé, the Aquitaine and Bourgogne Regional Councils, Fondation de France and the joint French Ministry of Research/INSERM “Cohortes et collections de données biologiques” programme. Lille Génopôle received an unconditional grant from Eisai.

Advance. *Action in Diabetes and Vascular disease: preteraX and diamicroN mr Controlled Evaluation study*. The genetic epidemiological work was funded by Prognomix Inc. and by grants from Genome Quebec and Canadian Institutes for Health Research (CIHR). The clinical study was managed by the George Institute for International Health (Sydney, Australia) with grants received from Les Laboratoires Servier, France and from Medical Research Council of Australia. The genotyping was performed at the genomic platform of CRCHUM. The authors acknowledge the technical help of Carole Long and Mounsif Haloui and the bioinformatic analyses performed by Gilles Godefroid, François-Christophe Blanchet-Marois and François Harvey. The members of the genetic sub-study of ADVANCE, Stephen Harrap and Michel Marre are also acknowledged.

AGES. *Age, Gene/Environment Susceptibility-Reykjavik Study*. This study has been funded by NIH contract N01-AG-1-2100, the NIA Intramural Research Program, Hjartavernd (the Icelandic Heart Association), and the Althingi (the Icelandic Parliament). The study is approved by the Icelandic National Bioethics Committee, VSN: 00-063. The researchers are indebted to the participants for their willingness to participate in the study.

Amish. *Amish studies*. We thank our Amish research volunteers for their long-standing partnership in research, and the research staff at the Amish Research Clinic for their work. We are supported by grants and contracts from the NIH including R01 AG18728 (Amish Longevity Study), R01 HL088119 (Amish Calcification Study), U01 GM074518-04 (PAPI Study), U01 HL072515-06 (HAPI Study), U01 HL084756 and NIH K12RR023250 (University of Maryland MCRDP), the University of Maryland General Clinical Research Center, grant M01 RR 16500, the Baltimore Veterans Administration Medical Center Geriatrics Research and Education Clinical Center and the Paul Beeson Physician Faculty Scholars in Aging Program.

ARIC. *Atherosclerosis Risk in Communities study*. The ARIC study is carried out as a collaborative study supported by National Heart, Lung, and Blood Institute contracts (HHSN268201100005C, HHSN268201100006C, HHSN268201100007C, HHSN268201100008C, HHSN268201100009C, HHSN268201100010C, HHSN268201100011C, and HHSN268201100012C), R01HL087641, R01HL59367 and R01HL086694; National Human Genome Research Institute contract U01HG004402; and National Institutes of Health contract HHSN268200625226C. The authors thank the staff and participants of the ARIC study for their important contributions. Infrastructure was partly supported by Grant Number UL1RR025005, a component of the National Institutes of Health and NIH Roadmap for Medical Research. This work as well as YL and AK were supported by the Emmy Noether Program of the German Research Foundation (KO 3598/2-1 to AK).

ASPS. *Austrian Stroke Prevention Study*. The research reported in this article was funded by the Austrian Science Fond (FWF) grant number P20545-P05 and P13180. The Medical University of Graz supports the databank of the ASPS. The authors thank the staff and the participants of the ASPS for their valuable contributions. We thank Birgit Reinhart for her long-term administrative commitment and Ing Johann Semmler for the technical assistance at creating the DNA-bank.

AUSTWIN. The *Australian Twin-Family Studies* were supported by NIH grants AA07535, AA07728, AA13320, AA13321, AA13326, AA14041, AA11998, AA17688, DA012854, DA019951; by grants from the Australian National Health and Medical Research Council (241944, 339462, 389927, 389875, 389891, 389892, 389938, 442915, 442981, 496739, 552485, 552498); from the Australian Research Council (A7960034, A79906588, A79801419, DP0770096, DP0212016, DP0343921); and the FP-5 GenomEUtwin Project (QLG2-CT-2002-01254). Genotyping was partially supported by grant AA13320 to the late Richard Todd, PhD, MD. R.P.M., and G.W.M. are supported by the National Health and Medical Research Council (NHMRC) Fellowship Scheme.

BLSA. *Baltimore Longitudinal Study of Aging*. The BLSA was supported in part by the Intramural Research Program of the NIH, National Institute on Aging.

BMES. *Blue Mountains Eye Study*. The BMES has been supported by the Australian RADGAC grant (1992-94) and Australian National Health & Medical Research Council, Canberra Australia (Grant Nos: 974159, 211069, 991407, 457349). The GWAS studies of Blue Mountains Eye Study population are supported by the Australian National Health & Medical Research Council (Grant Nos: 512423, 475604, 529912) and the Wellcome Trust, UK (2008). EGH and JJW are funded by the Australian National Health & Medical Research Council Fellowship Schemes.

Bus Santé study. This research was conducted in part using data and resources from the Bus Santé study of the Geneva University Hospitals. This work was partially supported by the Geneva University Hospitals, the Canton of Geneva (Switzerland), the General Directorate of Health (Canton of Geneva), the Swiss School of Public Health Plus (SSPH+) and the Swiss Foundation for Science (Contract No.495 33CM30-124087). Data on creatinine was founded by the Loterie Romande. The investigators of the Bus Santé study thank all the collaborators of the Unit of Population Epidemiology, and Abbott Diagnostics (Baar, Switzerland). We thank Dr Olivier Golaz and Dr Pierre Lescuyer, Geneva University Hospitals.

CARDIA. *Coronary Artery Risk in Young Adults.* University of Alabama at Birmingham (N01-HC-48047), University of Minnesota (N01-HC-48048), Northwestern University (N01-HC-48049), Kaiser Foundation Research Institute (N01-HC-48050), University of Alabama at Birmingham (N01-HC-95095), Tufts-New England Medical Center (N01-HC-45204), Wake Forest University (N01-HC-45205), Harbor-UCLA Research and Education Institute (N01-HC-05187), University of California, Irvine (N01-HC-45134, N01-HC-95100).

CHS. *Cardiovascular Health Study*. The CHS research reported in this article was supported by contract numbers N01-HC-85079 through N01-HC-85086, N01-HC-35129, N01 HC-15103, N01 HC-55222, N01-HC-75150, N01-HC-45133, grant numbers U01 HL080295 and R01 HL087652 from the National Heart, Lung, and Blood Institute, with additional contribution from the National Institute of Neurological Disorders and Stroke. A full list of principal CHS investigators and institutions can be found at http://www.chs-nhlbi.org/pi.htm. DNA handling and genotyping was supported in part by National Center for Research Resources grant M01RR00425 to the Cedars-Sinai General Clinical Research Center Genotyping core and National Institute of Diabetes and Digestive and Kidney Diseases grant DK063491 to the Southern California Diabetes Endocrinology Research Center.

CROATIA-KORCULA. The Korcula study in the Croatian island of Vis was supported through the grants from the Medical Research Council UK to H.C., A.F.W. and I.R.; and Ministry of Science, Education and Sport of the Republic of Croatia to I.R. (number 108-1080315-0302) . We would like to acknowledge the contributions of the recruitment team in Korcula, the administrative teams in Croatia and Edinburgh (Rosa Bisset) and the people of Korcula.

CROATIA-SPLIT. The CROATIA-Split study was supported through the grants from the Medical Research Council UK.;and Ministry of Science, Education and Sport of the Republic of Croatia. (number 108-1080315-0302). We would like to acknowledge the invaluable contributions of the recruitment team from the Croatian Centre for Global Health, University of Split, the administrative teams in Croatia and Edinburgh and the people of Split. The SNP genotyping for the SPLIT cohort was performed by AROS Applied Biotechnology, Aarhus, Denmark.

CROATIA-VIS. The Vis study in the Croatian island of Vis was supported through the grants from the Medical Research Council UK to H.C., A.F.W. and I.R.; and Ministry of Science, Education and Sport of the Republic of Croatia to I.R. (number 108-1080315-0302) and the European Union framework program 6 EUROSPAN project (contract no. LSHG-CT-2006-018947). We would like to acknowledge the invaluable contributions of the recruitment

team (including those from the Institute of Anthropological Research in Zagreb) in Vis, the administrative teams in Croatia and Edinburgh (Rosa Bisset) and the people of Vis.

DESIR. *Data from an Epidemiological Study on the Insulin Resistance syndrome*. DESIR genetic data was supported in part by the "Conseil Regional Nord-Pas-de-Calais: Fonds européen de développement économique et regional (CPER 2011-2013); Genome Quebec-Genome Canada; and the British Medical Research Council.

EGCUT. *Estonian Genome Center University of Tartu*. EGCUT received financing from FP7 grants (278913, 306031, 313010) and targeted financing from Estonian Government (SF0180142s08). EGCUT studies were covered from Infra-structure grant no. 3.2.0304.11-0312 funded mostly by the European Regional Development Fund, Center of Excellence in Genomics (EXCEGEN) and University of Tartu (SP1GVARENG). We acknowledge EGCUT technical personnel, especially Mr V. Soo and S. Smit. Data analyses were carried out in part in the High Performance Computing Center of the University of Tartu.

ERF. *Erasmus Rucphen Family study*. The ERF study was supported by grants from the Netherlands Organization for Scientific Research (NWO; Pioneergrant), Erasmus Medical Center, the Centre for Medical Systems Biology (CMSB), and the Netherlands Kidney Foundation. We are grateful to all patients and their relatives, general practitioners and neurologists for their contributions and to P. Veraart for her help in genealogy, Jeannette Vergeer for the supervision of the laboratory work and P. Snijders for his help in data collection.

ESTHER. *Epidemiologische Studie zu Chancen der Verhütung, Früherkennung und optimierten THerapie chronischer ERkrankungen in der älteren Bevölkerung*. We thank all the individuals who took part in this study and all the researchers, clinicians, technicians and administrative staff who have enabled this work to be carried out.This work was supported in part by the Baden-Württemberg State Ministry of Science, Research and Arts; by the German Federal Ministry of Education and Research (grant number 01ET0717); and by the CHANCES project funded in the Seventh Framework Programme of the Directorate-General for Research and Innovation in the European Commission (grant number 242244).

FamHS. *Family Heart Study*. The FHS work was supported in part by NIH grants 5R01HL08770003, 5R01HL08821502 (Michael A.Province) from the NHLBI and 5R01DK07568102, 5R01DK06833603 from the NIDDK (I.B.B.). The authors thank the staff and participants of the FamHS for their important contributions.

FHS. *Framingham Heart Study*. This research was conducted in part using data and resources from the Framingham Heart Study of the National Heart Lung and Blood Institute of the National Institutes of Health and Boston University School of Medicine. The analyses reflect intellectual input and resource development from the Framingham Heart Study investigators participating in the SNP Health Association Resource (SHARe) project. This work was partially supported by the National Heart, Lung and Blood Institute's Framingham Heart Study (Contract No. N01-HC-25195) and its contract with Affymetrix, Inc for genotyping services (Contract No. N02-HL-6-4278). A portion of this research utilized the Linux Cluster for Genetic Analysis (LinGA-II) funded by the Robert Dawson Evans Endowment of the Department of Medicine at Boston University School of Medicine and Boston Medical Center.

GENDIAN. *GENetics of DIAbetic Nephropathy study*. The support of the physicians, the patients, and the staff of the Diabetes Zentrum Mergentheim (Head: Prof. Dr. Thomas Haak), the diabetes outpatient clinic Dr Nusser - Dr Kreisel, the dialysis centers KfH Amberg, KfH Bayreuth, KfH Deggendorf, KfH Donauwörth, KfH Freising, KfH Freyung, KfH Fürth, KfH Hof, KfH Ingolstadt, KfH Kelheim, KfH München Elsenheimerstraße, KfH München-Schwabing, KfH Neumarkt, KfH Neusäß, KfH Oberschleißheim, KfH Passau, KfH Plauen, KfH Regensburg Günzstraße, KfH Regensburg Caritas-Krankenhaus, KfH Straubing, KfH Sulzbach-Rosenberg, KfH Weiden, Dialysezentrum Augsburg Dr. Kirschner, Dialysezentrum Bad Alexandersbad, KfH Bamberg, Dialysezentrum Emmering, Dialysezentrum Klinikum Landshut, Dialysezentrum Landshut, Dialysezentrum Pfarrkirchen, Dialysezentrum Schwandorf, Dr. Angela Götz, the medical doctoral student Johanna Christ and the Study Nurse Ingrid Lugauer. The expert technical assistance of Claudia Strohmeier is acknowledged. Phenotyping was funded by the Dr. Robert Pfleger-Stiftung (Dr Carsten A. Böger), the MSD Stipend Diabetes (Dr Carsten A. Böger) and the University Hospital of Regensburg (intramural grant ReForM A to Dr. A. Götz, ReForM C to Dr. Carsten Böger). Genome-wide genotyping was funded by the KfH Stiftung Präventivmedizin e.V. (Dr. Carsten A. Böger, Dr. Jens Brüning), the Else Kröner-Fresenius-Stiftung (2012_A147 to Dr Carsten A. Böger and Dr Iris M. Heid) and the University Hospital Regensburg (Dr Carsten A. Böger). Data analysis was funded by the Else Kröner-Fresenius Stiftung (Dr. Iris M. Heid and Dr. Carsten A. Böger: 2012_A147; Dr. Carsten A. Böger and Dr. Bernhard K. Krämer: P48/08//A11/08). GENDIAN Study Group: Mathias Gorski, Iris M. Heid, Bernhard K. Krämer, Myriam Rheinberger, Michael Broll, Alexander Lammert, Jens

Brüning, Matthias Olden, Klaus Stark, Claudia Strohmeier, Simone Neumeier, Sarah Hufnagel, Petra Jackermeier, Emilia Ruff, Johanna Christ, Peter Nürnberg, Thomas Haak, Carsten A. Böger.

GENOA. *Genetic Epidemiology Network Of Arteriopathy*. This research was partially supported by the National Heart Lung and Blood Institute of the National Institutes of Health R01 HL-87660.

GHS. *Gutenberg Heart Study*. The Gutenberg Health study is funded through the government of Rheinland-Pfalz (No. AZ 961-386261/733), the research program “Wissen schafft Zukunft” and the “Schwerpunkt Vaskuläre Prävention” of the Johannes Gutenberg University of Mainz and its contract with Boehringer Ingelheim and PHILIPS medical systems, including an unrestricted grant for the Gutenberg Health Study. Further, this project has been supported by the National Genome Network “NGFNplus” by the federal Ministry of Education and Research, Germany (No. 01GS0833 and 01GS0831, projects A3/D1).

GSK cases/controls. Funding Source: Max-Planck Society, German Federal Ministry of Education and Research (BMBF) in the framework of the National Genome Research Network (NGFN), Foerderkennzeichen 01GS0481.

HABC. *Health Aging and Body Composition Study*. The HABC study was funded by the National Institutes of Aging. This research was supported by NIA contracts N01AG62101, N01AG62103, and N01AG62106. The genome-wide association study was funded by NIA grant 1R01AG032098-01A1 to Wake Forest University Health Sciences and genotyping services were provided by the Center for Inherited Disease Research (CIDR). CIDR is fully funded through a federal contract from the National Institutes of Health to The Johns Hopkins University, contract number HHSN268200782096C. This research was supported in part by the Intramural Research Program of the NIH, National Institute on Aging.

HANDLS. This research was supported by the Intramural Research Program of the NIH, National Institute on Aging and the National Center on Minority Health and Health Disparities (Intramural Project # Z01-AG000513 and human subjects protocol # 2009-149). Data analyses for the HANDLS study utilized the high-performance computational capabilities of the Biowulf Linux cluster at the National Institutes of Health, Bethesda, Md. (http://biowulf.nih.gov).

HCS. *Hunter Community Study*. The University of Newcastle provided $300,000 from its Strategic Initiatives Fund, and $600,000 from the Gladys M Brawn Senior Research Fellowship scheme; Vincent Fairfax Family Foundation, a private philanthropic trust, provided $195,000; The Hunter Medical Research Institute provided media support during the initial recruitment of participants; and Dr Anne Crotty, Prof. Rodney Scott and Associate Prof. Levi provided financial support towards freezing costs for the long-term storage of participant blood samples. The authors would like to thank the men and women participating in the HCS as well as all the staff, investigators and collaborators who have supported or been involved in the project to date. A special thank you should go to Alison Koschel and Debbie Quain who were instrumental in setting up the pilot study and initial phase of the project.

HPFS. *Health Professionals Follow-Up Study.* The NHS/HPFS type 2 diabetes GWAS (U01HG004399) is a component of a collaborative project that includes 13 other GWAS (U01HG004738, U01HG004422, U01HG004402, U01HG004729, U01HG004726, U01HG004735, U01HG004415, U01HG004436, U01HG004423, U01HG004728, RFAHG006033; National Institute of Dental & Craniofacial Research: U01DE018993, U01DE018903) funded as part of the Gene Environment-Association Studies (GENEVA) under the NIH Genes, Environment and Health Initiative (GEI). Assistance with phenotype harmonization and genotype cleaning, as well as with general study coordination, was provided by the GENEVA Coordinating Center (U01HG004446). Assistance with data cleaning was provided by the National Center for Biotechnology Information. Genotyping was performed at the Broad Institute of MIT and Harvard, with funding support from the NIH GEI (U01HG04424), and Johns Hopkins University Center for Inherited Disease Research, with support from the NIH GEI (U01HG004438) and the NIH contract "High throughput genotyping for studying the genetic contributions to human disease”(HHSN268200782096C). Additional funding for the current research was provided by the National Cancer Institute (P01CA087969, P01CA055075), and the National Institute of Diabetes and Digestive and Kidney Diseases (R01DK058845). We thank the staff and participants of the NHS and HPFS for their dedication and commitment.

HUFS. The *Howard University Family Study* was supported by National Institutes of Health grants S06GM008016-320107 and S06GM008016-380111. Enrollment was carried out at the Howard University General Clinical Research Center, supported by National Institutes of Health grant 2M01RR010284. This research was supported in part by the

Intramural Research Program of the Center for Research on Genomics and Global Health. The Center for Research on Genomics and Global Health is supported by the National Human Genome Research Institute, the National Institute of Diabetes and Digestive and Kidney Diseases, the Center for Information Technology, and the Office of the Director at the National Institutes of Health (Z01HG200362). Genotyping support was provided by the Coriell Institute for Medical Research. We thank the participants of the Howard University Family Study. The contents of this publication are solely the responsibility of the authors and do not necessarily represent the official view of the National Institutes of Health.

HYPERGENES. *European Network for Genetic-Epidemiological Studies: building a method to dissect complex genetic traits, using essential hypertension as a disease model*. HYPERGENES (FP7 - HEALTH-F4-2007-201550); INTEROMICS (MIUR - CNR Italian Flagship Project); IC15-CT98-0329-EPOGH, LSHM-CT-2006-037093; HEALTH-2011-278249-EU-MASCARA, and ERC Advanced Grant-2011-294713-EPLORE and the Fonds voor Wetenschappelijk Onderzoek Vlaanderen, Ministry of the Flemish Community, Brussels, Belgium (grants G.0575.06 and G.0734.09).

Patricia B. Munroe's work forms part of the research themes contributing to the translational research portfolio for the NIHR Barts Cardiovascular Biomedical Research Unit.

INCIPE. The INCIPE study was co-sponsored by Fondazione Cassa di Risparmio di Verona, Azienda Ospedaliera di Verona, and University of Verona, Italy.

INGI-CARLANTINO. *Italian Network on Genetic Isolates – Carlantino.* We thank Anna Morgan and Angela D'Eustacchio for technical support. We are grateful to the municipal administrators for their collaboration on the project and for logistic support. We would like to thank all participants to this study.

INGI-CILENTO. *Italian Network on Genetic Isolates – Cilento.* We thank the populations of Cilento for their participation in the study. This work was supported by grants from the EU (Vasoplus-037254), the Italian Ministry of Universities (FIRB -RBIN064YAT), the Assessorato Ricerca Regione Campania, the Ente Parco Nazionale del Cilento e Vallo di Diano and the Fondazione Banco di Napoli to MC.

INGI-FVG. *Italian Network on Genetic Isolates – Friuli Venezia-Giulia.* We thank Anna Morgan and Angela D'Eustacchio for technical support. We are grateful to the municipal administrators for their collaboration on the project and for logistic support. We would like to thank all participants to this study.

INGI-VAL BORBERA.*Italian Network on Genetic Isolates – Val Borbera*. The research was done using data obtained thanks to funds from Compagnia di San Paolo, Torino, Italy, Cariplo Fundation, Milano, Italy and Italian Ministry of Health Progetto Finalizzato 2007 and 2009.

IPM. *Mount Sinai BioMe Biobank Program*. The Mount Sinai BioMe Biobank Program is supported by The Andrea and Charles Bronfman Philanthropies.

JHS. *Jackson Heart Study*. Jackson State University (N01-HC-95170), University of Mississippi (N01-HC-95171), Tougaloo College (N01-HC-95172).

JUPITER. We thank Jean MacFadyen, Lynda Rose, the JUPITER study participants and the >1000 physicians worldwide for their personal time, effort and commitment to JUPITER. This work was supported by research funds from AstraZeneca to Drs. Ridker and Chasman.

HRS. *Health and Retirement Study*. HRS is supported by the National Institute on Aging (NIA U01AG009740). The genotyping was funded as a separate award from the National Institute on Aging (RC2 AG036495). Our genotyping was conducted by the NIH Center for Inherited Disease Research (CIDR) at Johns Hopkins University. Genotyping quality control and final preparation of the data were perfomed by the Genetics Coordinating Center at the University of Washington.

KORA F3 and **F4**. The genetic epidemiological work was funded by the NIH subcontract from the Children's Hospital, Boston, US, (H.E.W., I.M.H, prime grant 1 R01 DK075787-01A1), the German National Genome Research Net NGFN2 and NGFNplus (H.E.W. 01GS0823; WK project A3, number 01GS0834), the Munich Center of Health

Sciences (MC Health) as part of LMUinnovativ, and by the Else Kröner-Fresenius-Stiftung (P48/08//A11/08; C.A.B., B.K.K; 2012_A147 to CAB and IMH.). The kidney parameter measurements in F3 were funded by the Else Kröner-Fresenius-Stiftung (C.A.B., B.K.K.) and the Regensburg University Medical Center, Germany; in F4 by the University of Ulm, Germany (W.K.). Genome wide genotyping costs in F3 and F4 were in part funded by the Else Kröner-Fresenius-Stiftung (C.A.B., B.K.K.). Denovo genotyping in F3 and F4 were funded by the Else Kröner-Fresenius-Stiftung (C.A.B., B.K.K.). The KORA research platform and the MONICA Augsburg studies were initiated and financed by the Helmholtz Zentrum München, German Research Center for Environmental Health, by the German Federal Ministry of Education and Research and by the State of Bavaria. Genotyping was performed in the Genome Analysis Center (GAC) of the Helmholtz Zentrum München. The LINUX platform for computation were funded by the University of Regensburg for the Department of Epidemiology and Preventive Medicine at the Regensburg University Medical Center.

LURIC. *Ludwigshafen Risk and Cardiovascular Health Study*. We extend our appreciation to the participants of the LURIC study; without their collaboration, this article would not have been written. We thank the LURIC study team who were either temporarily or permanently involved in patient recruitment as well as sample and data handling, in addition to the laboratory staff at the Ludwigshafen General Hospital and the Universities of Freiburg and Ulm, Germany. LURIC has received funding from the 6th Framework Program (integrated project Bloodomics, grant LSHM-CT-2004-503485) and 7th Framework Program (Atheroremo, grant agreement number 201668 and RiskyCAD, grant agreement number 305739) of the European Union.

MESA. *Multi-Ethnic Study of Atherosclerosis*. University of Washington (N01-HC-95159),Regents of the University of California (N01-HC-95160), Columbia University (N01-HC-95161), Johns Hopkins University (N01-HC-95162, N01-HC-95168), University of Minnesota (N01-HC-95163), Northwestern University (N01-HC-95164), Wake Forest University (N01-HC-95165), University of Vermont (N01-HC-95166), New England Medical Center (N01-HC-95167), Harbor-UCLA Research and Education Institute (N01-HC-95169), Cedars-Sinai Medical Center (R01-HL-071205), University of Virginia (subcontract to R01-HL-071205)

MICROS. *Microisolates in South Tyrol study*. We owe a debt of gratitude to all participants. We thank the primary care practitioners Raffaela Stocker, Stefan Waldner, Toni Pizzecco, Josef Plangger, Ugo Marcadent and the personnel of the Hospital of Silandro (Department of Laboratory Medicine) for their participation and collaboration in the research project. We thank Dr. Peter Riegler (Hemodialysis Unit, Hospital of Merano) for the important discussions. In South Tyrol, the study was supported by the Ministry of Health and Department of Educational Assistance, University and Research of the Autonomous Province of Bolzano, the South Tyrolean Sparkasse Foundation, and the European Union framework program 6 EUROSPAN project (contract no. LSHG-CT-2006-018947).

NESDA. *The Netherlands Study of Depression and Anxiety*. The infrastructure for the NESDA is funded through the Geestkracht programme of the Dutch Scientific Organization (ZON-MW, grant number 10-000-1002) and matching funds from participating universities and mental health care organizations. Genotyping in NESDA was funded by the Genetic Association Information Network (GAIN) of the Foundation for the US National Institutes of Health. Statistical analyses were carried out on the Genetic Cluster Computer (http://www.geneticcluster.org), which is financially supported by the Netherlands Scientific Organization (I 480-05-003) along with a supplement from the Dutch Brain Foundation.

NHS. *Nurses' Health Study*. The NHS/HPFS type 2 diabetes GWAS (U01HG004399) is a component of a collaborative project that includes 13 other GWAS (U01HG004738, U01HG004422, U01HG004402, U01HG004729, U01HG004726, U01HG004735, U01HG004415, U01HG004436, U01HG004423, U01HG004728, RFAHG006033; National Institute of Dental & Craniofacial Research: U01DE018993, U01DE018903) funded as part of the Gene Environment-Association Studies (GENEVA) under the NIH Genes, Environment and Health Initiative (GEI). Assistance with phenotype harmonization and genotype cleaning, as well as with general study coordination, was provided by the GENEVA Coordinating Center (U01HG004446). Assistance with data cleaning was provided by the National Center for Biotechnology Information. Genotyping was performed at the Broad Institute of MIT and Harvard, with funding support from the NIH GEI (U01HG04424), and Johns Hopkins University Center for Inherited Disease Research, with support from the NIH GEI (U01HG004438) and the NIH contract "High throughput genotyping for studying the genetic contributions to human disease”(HHSN268200782096C). The NHS renal function and

albuminuria work was supported by DK66574. Additional funding for the current research was provided by the National Cancer Institute (P01CA087969, P01CA055075), and the National Institute of Diabetes and Digestive and Kidney Diseases (R01DK058845). We thank the staff and participants of the NHS and HPFS for their dedication and commitment.

NSPHS. *The Northern Swedish Population Health Study*. The NSPHS was supported by grants from the Swedish Natural Sciences Research Council, the European Union through the EUROSPAN project (contract no. LSHG-CT-2006-018947), the Foundation for Strategic Research (SSF) and the Linneaus Centre for Bioinformatics (LCB). We are also grateful for the contribution of samples from the Medical Biobank in Umeå and for the contribution of the district nurse Svea Hennix in the Karesuando study.

OGP (*Ogliastra Genetic Park*) including **OGP-TALANA**. We thank the Ogliastra population and all the individuals who participated in this study. We are grateful to the municipal administrators for their collaboration to the project and for economic and logistic support. This work was supported by grants from the Italian Ministry of Education, University and Research (MIUR) no.5571/DSPAR/2002 and (FIRB) D. M. no. 718/Ric/2005.

ORCADES. *Orkney Complex Disease Study*. ORCADES was supported by the the Chief Scientist Office of the Scottish Government , the Royal Society and the European Union framework program 6 EUROSPAN project (contract no. LSHG-CT-2006-018947). DNA extractions were performed at the Wellcome Trust Clinical Research Facility in Edinburgh. We would like to acknowledge the invaluable contributions of Lorraine Anderson, the research nurses in Orkney, the administrative team in Edinburgh and the people of Orkney.

POPGEN. The POPGen study was supported by the German Ministry of Education and Research (BMBF) through the National Genome Research Network (NGFN). It is currently funded by the Ministry of Science, Commerce and Transportation of the State of Schleswig-Holstein. The project has also received infrastructure support through the DFG excellence cluster “Inflammation at Interfaces” and the BMBF funded project P2N (01EY1103).

PREVEND. *Prevention of Renal and Vascular Endstage Disease Study*. PREVEND genetics is supported by the Dutch Kidney Foundation (Grant E033), the EU project grant GENECURE (FP-6 LSHM CT 2006 037697), the National Institutes of Health (grant 2R01LM010098), The Netherlands organisation for health research and development (NWO-Groot grant 175.010.2007.006, NWO VENI grant 916.761.70, ZonMw grant 90.700.441), and the Dutch Inter University Cardiology Institute Netherlands (ICIN).

PROSPER-PHASE. *Prospective Study of Pravastatin in the Elderly at Risk.* The PROSPER trial was supported by an investigator initiated grant from Bristol-Myers Squibb, USA. The study was conducted, analysed, and reported independently of the company.

RS-I and **II**. *The Rotterdam Study*. The GWA study was funded by the Netherlands Organisation of Scientific Research NWO Investments (nr. 175.010.2005.011, 911-03-012), the Research Institute for Diseases in the Elderly (014-93-015; RIDE2), the Netherlands Genomics Initiative (NGI)/Netherlands Consortium for Healthy Aging (NCHA) project nr. 050-060-810. We thank Pascal Arp, Mila Jhamai, Dr Michael Moorhouse, Marijn Verkerk, and Sander Bervoets for their help in creating the GWAS database. The Rotterdam Study is funded by Erasmus Medical Center and Erasmus University, Rotterdam, Netherlands Organization for the Health Research and Development (ZonMw), the Research Institute for Diseases in the Elderly (RIDE), the Ministry of Education, Culture and Science, the Ministry for Health, Welfare and Sports, the European Commission (DG XII), and the Municipality of Rotterdam. The authors are very grateful to the participants and staff from the Rotterdam Study, the participating general practioners and the pharmacists. We would like to thank Dr. Tobias A. Knoch, Luc V. de Zeeuw, Anis Abuseiris, and Rob de Graaf as well as their institutions the Erasmus Computing Grid, Rotterdam, The Netherlands, and especially the national German MediGRID and Services@MediGRID part of the German D-Grid, both funded by the German Bundesministerium fuer Forschung und Technology under grants #01 AK 803 A-H and # 01 IG 07015 G, for access to their grid resources. Abbas Dehghan is supported by NWO grant (vici, 918-76-619).

SAPALDIA. *Swiss Study on Air Pollution and Lung Diseases in Adults*. The SAPALDIA Team: Study directorate: T Rochat (p), NM Probst Hensch (e/g), N Künzli (e/exp), C Schindler (s), JM Gaspoz (c) Scientific team: JC Barthélémy (c), W Berger (g), R Bettschart (p), A Bircher (a), O Brändli (p), C Brombach (n), M Brutsche (p), L Burdet (p), M Frey (p), U Frey (pd), MW Gerbase (p), D Gold (e/c/p), E de Groot (c), W Karrer (p), R Keller (p), B Martin (pa), D Miedinger (o), U Neu (exp), L Nicod (p), M Pons (p), F Roche (c), T Rothe (p), E Russi (p), P Schmid-Grendelmeyer

(a), A Schmidt-Trucksäss (pa), A Turk (p), J Schwartz (e), D. Stolz (p), P Straehl (exp), JM Tschopp (p), A von Eckardstein (cc), E Zemp Stutz (e). Scientific team at coordinating centers: M Adam (e/g), C Autenrieth (pa), PO Bridevaux (p), D Carballo (c), E Corradi (exp), I Curjuric (e), J Dratva (e), A Di Pasquale (s), E Dupuis Lozeron (s), E Fischer (e), M Germond (s), L Grize (s), D Keidel (s), S Kriemler (pa), A Kumar (g), M Imboden (g), N Maire (s), A Mehta (e), H Phuleria (exp), E Schaffner (s), GA Thun (g) A Ineichen (exp), M Ragettli (e), M Ritter (exp), T Schikowski (e), M Tarantino (s), M Tsai (exp) (a) allergology, (c) cardiology, (cc) clinical chemistry, (e) epidemiology, (exp) exposure, (g) genetic and molecular biology, (m) meteorology, (n) nutrition, (o) occupational health, (p) pneumology, (pa) physical activity, (pd) pediatrics, (s) statistics. Funding: The Swiss National Science Foundation (grants no 33CSCO-134276/1, 33CSCO-108796, 3247BO-104283, 3247BO-104288, 3247BO-104284, 3247-065896, 3100-059302, 3200-052720, 3200-042532, 4026-028099), the Federal Office for Forest, Environment and Landscape, the Federal Office of Public Health, the Federal Office of Roads and Transport, the canton's government of Aargau, Basel-Stadt, Basel-Land, Geneva, Luzern, Ticino, Valais, and Zürich, the Swiss Lung League, the canton's Lung League of Basel Stadt/ Basel Landschaft, Geneva, Ticino, Valais and Zurich, SUVA, Freiwillige Akademische Gesellschaft, UBS Wealth Foundation, Talecris Biotherapeutics GmbH, Abbott Diagnostics, European Commission 018996 (GABRIEL), Wellcome Trust WT 084703MA. The study could not have been done without the help of the study participants, technical and administrative support and the medical teams and field workers at the local study sites. Local fieldworkers : Aarau: S Brun, G Giger, M Sperisen, M Stahel, Basel: C Bürli, C Dahler, N Oertli, I Harreh, F Karrer, G Novicic, N Wyttenbacher, Davos: A Saner, P Senn, R Winzeler, Geneva: F Bonfils, B Blicharz, C Landolt, J Rochat, Lugano: S Boccia, E Gehrig, MT Mandia, G Solari, B Viscardi, Montana: AP Bieri, C Darioly, M Maire, Payerne: F Ding, P Danieli A Vonnez, Wald: D Bodmer, E Hochstrasser, R Kunz, C Meier, J Rakic, U Schafroth, A Walder. Administrative staff: C Gabriel, R Gutknecht.

SAPHIR. *Salzburg Atherosclerosis Prevention program in subjects at High Individual Risk*. The SAPHIR-study was partially supported by a grant from the Kamillo Eisner Stiftung to B. Paulweber and by grants from the "Genomics of Lipid-associated Disorders – GOLD" of the "Austrian Genome Research Programme GEN-AU" to F. Kronenberg.

SHIP and **SHIP-TREND**. *The Study of Health in Pomerania*. SHIP is part of the Community Medicine Research net of the University of Greifswald, Germany, which is funded by the Federal Ministry of Education and Research (grants no. 01ZZ9603, 01ZZ0103, and 01ZZ0403), the Ministry of Cultural Affairs as well as the Social Ministry of the Federal State of Mecklenburg-West Pomerania, and the network 'Greifswald Approach to Individualized Medicine (GANI_MED)' funded by the Federal Ministry of Education and Research (grant 03IS2061A). Genome-wide data have been supported by the Federal Ministry of Education and Research (grant no. 03ZIK012) and a joint grant from Siemens Healthcare, Erlangen, Germany and the Federal State of Mecklenburg- West Pomerania. The University of Greifswald is a member of the 'Center of Knowledge Interchange' program of the Siemens AG and the Caché Campus program of the InterSystems GmbH. The SHIP authors are grateful to Mario Stanke for the opportunity to use his Server Cluster for the SNP imputation as well as to Holger Prokisch and Thomas Meitinger (Helmholtz Zentrum München) for the genotyping of the SHIP-TREND cohort.

SIGNET-Sea Islands and **SIGNET-REGARDS**. The *Sea Islands Genetics Network* is supported by R01 DK084350 (M Sale), and consists of data from the REasons for Geographic And Racial Differences in Stroke (REGARDS) cohort (U01 NS041588; G Howard), Project SuGAR (Sea Islands Genetic African American Registry; W.M. Keck Foundation; WT Garvey), and a South Carolina Center of Biomedical Research Excellence (COBRE) for Oral Health Project P20-RR-17696 (JK Fernandes).

SKIPOGH. *The Swiss Kidney Project on Genes in Hypertension.* This research was funded by a grant from the Swiss National Science Foundation (33CM30-124087). The study also received support from Lausanne University Hospital, Geneva University Hospital and Bern University Hospital, Switzerland. Murielle Bochud received support from the Swiss School of Public Health Plus (SSPH+).

SORBS. *The Sorbs study*. This work was supported by grants from the German Research Council (SFB- 1052 "Obesity mechanisms" to Michael Stumvoll, Anke Tönjes and Peter Kovacs), from the German Diabetes Association (to Anke Tönjes and Peter Kovacs) and from the DHFD (Diabetes Hilfs- und Forschungsfonds Deutschland to Michael Stumvoll and Peter Kovacs). IFB AdiposityDiseases is supported by the Federal Ministry of Education and Research (BMBF), Germany, FKZ: 01EO1001. We thank all those who participated in the study. Sincere thanks are

given to Knut Krohn (Microarray Core Facility of the Interdisciplinary Centre for Clinical Research, University of Leipzig) for the genotyping support.

Vanderbilt. Illumina HumanOmni1-Quad and Illumina HumanOmni5-Quad genoytping were supported by NIGMS RC2-GM092318. Illumina Human660W-Quad genotyping was supported by the eMERGE Network, initiated and funded by the National Human Genome Research Institute, with additional funding from the National Institute of General Medical Sciences (NIGMS), through U01-HG04603.

WGHS. *Women's Genome Health Study*. The WGHS is supported by HL 043851 and HL69757 from the National Heart, Lung, and Blood Institute and CA 047988 from the National Cancer Institute, the Donald W. Reynolds Foundation and the Fondation Leducq, with collaborative scientific support and funding for genotyping provided by Amgen.

YFS. *Young Finns Study*. The YFS has been financially supported by the Academy of Finland: grants 134309 (Eye), 126925, 121584, 124282, 129378 (Salve), 117787 (Gendi), and 41071 (Skidi), the Social Insurance Institution of Finland, Kuopio, Tampere and Turku University Hospital Medical Funds (grant 9M048 and 9N035 for TeLeht), Juho Vainio Foundation, Paavo Nurmi Foundation, Finnish Foundation of Cardiovascular Research and Finnish Cultural Foundation, Tampere Tuberculosis Foundation and Emil Aaltonen Foundation (T.L). The expert technical assistance in the statistical analyses by Ville Aalto and Irina Lisinen is acknowledged.

References

1. Freedman, M. L. *et al*. Assessing the impact of population stratification on genetic association studies. *Nat. Genet.* **36**, 388-393 (2004).
2. de Bakker, P. I. *et al*. Practical aspects of imputation-driven meta-analysis of genome-wide association studies. *Hum. Mol. Genet.* **17**, R122-8 (2008).
3. Pattaro, C. *et al*. Genome-wide association and functional follow-up reveals new loci for kidney function. *PLoS Genet.* **8**, e1002584 (2012).
4. Kottgen, A. *et al*. New loci associated with kidney function and chronic kidney disease. *Nat. Genet.* **42**, 376-384 (2010).
5. Kottgen, A. *et al*. Multiple loci associated with indices of renal function and chronic kidney disease. *Nat. Genet.* **41**, 712-717 (2009).
6. Chasman, D. I. *et al*. Integration of genome-wide association studies with biological knowledge identifies six novel genes related to kidney function. *Hum. Mol. Genet.* **21**, 5329-5343 (2012).
7. Johnson, A. D. *et al*. SNAP: a web-based tool for identification and annotation of proxy SNPs using HapMap. *Bioinformatics* **24**, 2938-2939 (2008).
8. Schunkert, H. *et al*. Large-scale association analysis identifies 13 new susceptibility loci for coronary artery disease. *Nat. Genet.* **43**, 333-338 (2011).
9. Vasan, R. S. *et al*. Genetic variants associated with cardiac structure and function: a meta-analysis and replication of genome-wide association data. *JAMA* **302**, 168-178 (2009).
10. Smith, N. L. *et al*. Association of genome-wide variation with the risk of incident heart failure in adults of European and African ancestry: a prospective meta-analysis from the cohorts for heart and aging research in genomic epidemiology (CHARGE) consortium. *Circ. Cardiovasc. Genet.* **3**, 256-266 (2010).
11. International Consortium for Blood Pressure Genome-Wide Association Studies *et al*. Genetic variants in novel pathways influence blood pressure and cardiovascular disease risk. *Nature* **478**, 103-109 (2011).
12. Dupuis, J. *et al*. New genetic loci implicated in fasting glucose homeostasis and their impact on type 2 diabetes risk. *Nat. Genet.* **42**, 105-116 (2010).
13. Schizophrenia Psychiatric Genome-Wide Association Study (GWAS) Consortium. Genome-wide association study identifies five new schizophrenia loci. *Nat. Genet.* **43**, 969-976 (2011).
14. Berndt, S. I. *et al*. Genome-wide meta-analysis identifies 11 new loci for anthropometric traits and provides insights into genetic architecture. *Nat. Genet.* **45**, 501-512 (2013).
15. Thorleifsson, G. *et al*. Genome-wide association yields new sequence variants at seven loci that associate with measures of obesity. *Nat. Genet.* **41**, 18-24 (2009).
16. Speliotes, E. K. *et al*. Association analyses of 249,796 individuals reveal 18 new loci associated with body mass index. *Nat. Genet.* **42**, 937-948 (2010).
17. Jostins, L. *et al*. Host-microbe interactions have shaped the genetic architecture of inflammatory bowel disease. *Nature* **491**, 119-124 (2012).
18. Mells, G. F. *et al*. Genome-wide association study identifies 12 new susceptibility loci for primary biliary cirrhosis. *Nat. Genet.* **43**, 329-332 (2011).
19. Okada, Y. *et al*. Meta-analysis identifies multiple loci associated with kidney function-related traits in east Asian populations. *Nat. Genet.* **44**, 904-909 (2012).
20. Kottgen, A. *et al*. Genome-wide association analyses identify 18 new loci associated with serum urate concentrations. *Nat. Genet.* **45**, 145-154 (2013).
21. Paternoster, L. *et al*. Meta-analysis of genome-wide association studies identifies three new risk loci for atopic dermatitis. *Nat. Genet.* **44**, 187-192 (2011).
22. Teslovich, T. M. *et al*. Biological, clinical and population relevance of 95 loci for blood lipids. *Nature* **466**, 707-713 (2010).
23. Yang, Q. *et al*. Multiple genetic loci influence serum urate levels and their relationship with gout and cardiovascular disease risk factors. *Circ. Cardiovasc. Genet.* **3**, 523-530 (2010).
24. van Meurs, J. B. *et al*. Common genetic loci influencing plasma homocysteine concentrations and their effect on risk of coronary artery disease. *Am. J. Clin. Nutr.* **98**, 668-676 (2013).
25. Barrett, J. H. *et al*. Genome-wide association study identifies three new melanoma susceptibility loci. *Nat. Genet.* **43**, 1108-1113 (2011).
26. Bishop, D. T. *et al*. Genome-wide association study identifies three loci associated with melanoma risk. *Nat. Genet.* **41**, 920-925 (2009).
27. Han, J. *et al*. A genome-wide association study identifies novel alleles associated with hair color and skin pigmentation. *PLoS Genet.* **4**, e1000074 (2008).
28. Zhang, M. *et al*. Genome-wide association studies identify several new loci associated with pigmentation traits and skin cancer risk in European Americans. *Hum. Mol. Genet.* **22**, 2948-2959 (2013).
29. Nan, H. *et al*. Genome-wide association study identifies novel alleles associated with risk of cutaneous basal cell carcinoma and squamous cell carcinoma. *Hum. Mol. Genet.* **20**, 3718-3724 (2011).

30. Sulem, P. *et al*. Genetic determinants of hair, eye and skin pigmentation in Europeans. *Nat. Genet.* **39**, 1443-1452 (2007).
31. Siddiq, A. *et al*. A meta-analysis of genome-wide association studies of breast cancer identifies two novel susceptibility loci at 6q14 and 20q11. *Hum. Mol. Genet.* **21**, 5373-5384 (2012).
32. Sulem, P. *et al*. Two newly identified genetic determinants of pigmentation in Europeans. *Nat. Genet.* **40**, 835-837 (2008).
33. Brown, K. M. *et al*. Common sequence variants on 20q11.22 confer melanoma susceptibility. *Nat. Genet.* **40**, 838-840 (2008).
34. Morris, A. P. Transethnic meta-analysis of genomewide association studies. *Genet. Epidemiol.* **35**, 809-822 (2011).
35. Zhang, B. *et al*. Integrated systems approach identifies genetic nodes and networks in late-onset Alzheimer's disease. *Cell* **153**, 707-720 (2013).
36. Greenawalt, D. M. *et al*. A survey of the genetics of stomach, liver, and adipose gene expression from a morbidly obese cohort. *Genome Res.* **21**, 1008-1016 (2011).
37. Schadt, E. E. *et al*. Mapping the genetic architecture of gene expression in human liver. *PLoS Biol.* **6**, e107 (2008).
38. Innocenti, F. *et al*. Identification, replication, and functional fine-mapping of expression quantitative trait loci in primary human liver tissue. *PLoS Genet.* **7**, e1002078 (2011).
39. Schroder, A. *et al*. Genomics of ADME gene expression: mapping expression quantitative trait loci relevant for absorption, distribution, metabolism and excretion of drugs in human liver. *Pharmacogenomics J.* **13**, 12-20 (2013).
40. Fehrmann, R. S. *et al*. Trans-eQTLs reveal that independent genetic variants associated with a complex phenotype converge on intermediate genes, with a major role for the HLA. *PLoS Genet.* **7**, e1002197 (2011).
41. Grundberg, E. *et al*. Mapping cis- and trans-regulatory effects across multiple tissues in twins. *Nat. Genet.* **44**, 1084-1089 (2012).
42. Dimas, A. S. *et al*. Common regulatory variation impacts gene expression in a cell type-dependent manner. *Science* **325**, 1246-1250 (2009).
43. Zeller, T. *et al*. Genetics and beyond--the transcriptome of human monocytes and disease susceptibility. *PLoS One* **5**, e10693 (2010).
44. Emilsson, V. *et al*. Genetics of gene expression and its effect on disease. *Nature* **452**, 423-428 (2008).
45. Hao, K. *et al*. Lung eQTLs to help reveal the molecular underpinnings of asthma. *PLoS Genet.* **8**, e1003029 (2012).
46. Kabakchiev, B. & Silverberg, M. S. Expression quantitative trait loci analysis identifies associations between genotype and gene expression in human intestine. *Gastroenterology* **144**, 1488-96, 1496.e1-3 (2013).
47. Liang, L. *et al*. A cross-platform analysis of 14,177 expression quantitative trait loci derived from lymphoblastoid cell lines. *Genome Res.* **23**, 716-726 (2013).
48. Degner, J. F. *et al*. DNase I sensitivity QTLs are a major determinant of human expression variation. *Nature* **482**, 390-394 (2012).
49. Li, Q. *et al*. Integrative eQTL-based analyses reveal the biology of breast cancer risk loci. *Cell* **152**, 633-641 (2013).
50. Zou, F. *et al*. Brain expression genome-wide association study (eGWAS) identifies human disease-associated variants. *PLoS Genet.* **8**, e1002707 (2012).
51. Goring, H. H. *et al*. Discovery of expression QTLs using large-scale transcriptional profiling in human lymphocytes. *Nat. Genet.* **39**, 1208-1216 (2007).
52. Zhu, M. *et al*. Beta-mannosidosis mice: a model for the human lysosomal storage disease. *Hum. Mol. Genet.* **15**, 493-500 (2006).
53. Gill, P. S. & Rosenblum, N. D. Control of murine kidney development by sonic hedgehog and its GLI effectors. *Cell. Cycle* **5**, 1426-1430 (2006).
54. Lu, B. C. *et al*. Etv4 and Etv5 are required downstream of GDNF and Ret for kidney branching morphogenesis. *Nat. Genet.* **41**, 1295-1302 (2009).
55. Kozak, E. M. & Tate, S. S. Glutathione-degrading enzymes of microvillus membranes. *J. Biol. Chem.* **257**, 6322-6327 (1982).
56. Campbell, B. J., Forrester, L. J., Zahler, W. L. & Burks, M. Beta-lactamase activity of purified and partially characterized human renal dipeptidase. *J. Biol. Chem.* **259**, 14586-14590 (1984).
57. Mazumder, B. *et al*. Regulated release of L13a from the 60S ribosomal subunit as a mechanism of transcript-specific translational control. *Cell* **115**, 187-198 (2003).
58. Koppen, M., Metodiev, M. D., Casari, G., Rugarli, E. I. & Langer, T. Variable and tissue-specific subunit composition of mitochondrial m-AAA protease complexes linked to hereditary spastic paraplegia. *Mol. Cell. Biol.* **27**, 758-767 (2007).
59. Elleuch, N. *et al*. Mutation analysis of the paraplegin gene (SPG7) in patients with hereditary spastic paraplegia. *Neurology* **66**, 654-659 (2006).
60. Arnoldi, A. *et al*. A clinical, genetic, and biochemical characterization of SPG7 mutations in a large cohort of patients with hereditary spastic paraplegia. *Hum. Mutat.* **29**, 522-531 (2008).

61. Sanchez-Ferrero, E. *et al*. SPG7 mutational screening in spastic paraplegia patients supports a dominant effect for some mutations and a pathogenic role for p.A510V. *Clin. Genet.* **83**, 257-262 (2013).
62. Tekin, M. *et al*. The KBG syndrome: confirmation of autosomal dominant inheritance and further delineation of the phenotype. *Am. J. Med. Genet. A.* **130A**, 284-287 (2004).
63. Sirmaci, A. *et al*. Mutations in ANKRD11 cause KBG syndrome, characterized by intellectual disability, skeletal malformations, and macrodontia. *Am. J. Hum. Genet.* **89**, 289-294 (2011).
64. Li, X. *et al*. Cloning and chromosomal localization of the human and murine genes for the T-cell transcription factors NFATc and NFATp. *Cytogenet. Cell Genet.* **68**, 185-191 (1995).
65. Langworthy, M., Zhou, B., de Caestecker, M., Moeckel, G. & Baldwin, H. S. NFATc1 identifies a population of proximal tubule cell progenitors. *J. Am. Soc. Nephrol.* **20**, 311-321 (2009).
66. Wang, Y. *et al*. Activation of NFAT signaling in podocytes causes glomerulosclerosis. *J. Am. Soc. Nephrol.* **21**, 1657-1666 (2010).
67. Varon, R. *et al*. Partial deficiency of the C-terminal-domain phosphatase of RNA polymerase II is associated with congenital cataracts facial dysmorphism neuropathy syndrome. *Nat. Genet.* **35**, 185-189 (2003).
68. Ahuja, R. *et al*. Interactions of cubilin with megalin and the product of the amnionless gene (AMN): effect on its stability. *Biochem. J.* **410**, 301-308 (2008).
69. Kantarci, S. *et al*. Mutations in LRP2, which encodes the multiligand receptor megalin, cause Donnai-Barrow and facio-oculo-acoustico-renal syndromes. *Nat. Genet.* **39**, 957-959 (2007).
70. Attakpa, E. S., Djibril, N. M., Baba-Moussa, F., Yessoufou, G. & Sezan, A. Expression and role of the genes involved in the transport of bile acids in the liver and kidneys in mice. *J. Basic Clin. Physiol. Pharmacol.* **24**, 97-103 (2013).
71. Parr, B. A. & McMahon, A. P. Sexually dimorphic development of the mammalian reproductive tract requires Wnt-7a. *Nature* **395**, 707-710 (1998).
72. Hirata, H. *et al*. Polymorphisms of DNA repair genes are associated with renal cell carcinoma. *Biochem. Biophys. Res. Commun.* **342**, 1058-1062 (2006).
73. Key, M. D., Andres, D. A., Der, C. J. & Repasky, G. A. Characterization of RERG: an estrogen-regulated tumor suppressor gene. *Methods Enzymol.* **407**, 513-527 (2006).
74. Ozaltin, F. *et al*. Disruption of PTPRO causes childhood-onset nephrotic syndrome. *Am. J. Hum. Genet.* **89**, 139-147 (2011).
75. Wharram, B. L. *et al*. Altered podocyte structure in GLEPP1 (Ptpro)-deficient mice associated with hypertension and low glomerular filtration rate. *J. Clin. Invest.* **106**, 1281-1290 (2000).
76. Zwaenepoel, I. *et al*. Ezrin regulates microvillus morphogenesis by promoting distinct activities of Eps8 proteins. *Mol. Biol. Cell* **23**, 1080-1094 (2012).
77. Garrett, S. H. *et al*. Short and long term gene expression variation and networking in human proximal tubule cells when exposed to cadmium. *BMC Med. Genomics* **6 Suppl 1**, S2-8794-6-S1-S2. Epub 2013 Jan 23 (2013).
78. Cheng, S. B. *et al*. Anatomical location and redistribution of G protein-coupled estrogen receptor-1 during the estrus cycle in mouse kidney and specific binding to estrogens but not aldosterone. *Mol. Cell. Endocrinol.* **382**, 950-959 (2014).
79. Prabhushankar, R., Krueger, C. & Manrique, C. Membrane estrogen receptors: their role in blood pressure regulation and cardiovascular disease. *Curr. Hypertens. Rep.* **16**, 408-013-0408-6 (2014).
80. Kurt, A. H. & Buyukafsar, K. Vasoconstriction induced by G1, a G-protein-coupled oestrogen receptor1 (GPER-1) agonist, in the isolated perfused rat kidney. *Eur. J. Pharmacol.* **702**, 71-78 (2013).
81. Barton, M. Position paper: The membrane estrogen receptor GPER--Clues and questions. *Steroids* **77**, 935-942 (2012).
82. Osborne, M. R. *et al*. The reaction of 7,8-dihydro-7,8-dihydroxybenzo (a) pyrene-9,10-oxide with DNA in relation to the benzo (a) pyrene-DNA products isolated from cells. *Chem. Biol. Interact.* **13**, 343-348 (1976).
83. Huang, W. *et al*. High glucose induces activation of NF-kappaB inflammatory signaling through IkappaBalpha sumoylation in rat mesangial cells. *Biochem. Biophys. Res. Commun.* **438**, 568-574 (2013).
84. Pandey, D. *et al*. SUMO1 negatively regulates reactive oxygen species production from NADPH oxidases. *Arterioscler. Thromb. Vasc. Biol.* **31**, 1634-1642 (2011).
85. Zhang, L. *et al*. A high-throughput screen for chemical inhibitors of exocytic transport in yeast. *Chembiochem* **11**, 1291-1301 (2010).
86. Zhang, L., Huang, M. & Harsay, E. A chemical genetic screen for modulators of exocytic transport identifies inhibitors of a transport mechanism linked to GTR2 function. *Eukaryot. Cell.* **9**, 116-126 (2010).
87. Nowak, J. *et al*. The TP53INP2 protein is required for autophagy in mammalian cells. *Mol. Biol. Cell* **20**, 870-881 (2009).
88. Teng, A., Nair, M., Wells, J., Segre, J. A. & Dai, X. Strain-dependent perinatal lethality of Ovol1-deficient mice and identification of Ovol2 as a downstream target of Ovol1 in skin epidermis. *Biochim. Biophys. Acta* **1772**, 89-95 (2007).
89. Lee, S. D. *et al*. TonEBP stimulates multiple cellular pathways for adaptation to hypertonic stress: organic osmolyte-dependent and -independent pathways. *Am. J. Physiol. Renal Physiol.* **300**, F707-15 (2011).
90. Davis, L. K., Rodgers, B. D. & Kelley, K. M. Angiotensin II- and glucose-stimulated extracellular matrix production: mediation by the insulin-like growth factor (IGF) axis in a murine mesangial cell line. *Endocrine* **33**, 32-39 (2008).

91. Fukasawa, H. *et al*. Ubiquitin-dependent degradation of SnoN and Ski is increased in renal fibrosis induced by obstructive injury. *Kidney Int.* **69**, 1733-1740 (2006).
92. Kirk, A., Campbell, S., Bass, P., Mason, J. & Collins, J. Differential expression of claudin tight junction proteins in the human cortical nephron. *Nephrol. Dial. Transplant.* **25**, 2107-2119 (2010).
93. Kugita, M. *et al*. Global gene expression profiling in early-stage polycystic kidney disease in the Han:SPRD Cy rat identifies a role for RXR signaling. *Am. J. Physiol. Renal Physiol.* **300**, F177-88 (2011).
94. Ng, M. C. *et al*. Transferability and fine mapping of type 2 diabetes loci in African Americans: the Candidate Gene Association Resource Plus Study. *Diabetes* **62**, 965-976 (2013).
95. Lim, X. L. *et al*. KCNQ1 SNPS and susceptibility to diabetic nephropathy in East Asians with type 2 diabetes. *Diabetologia* **55**, 2402-2406 (2012).
96. Liu, C. T. *et al*. Genetic association for renal traits among participants of African ancestry reveals new loci for renal function. *PLoS Genet.* **7**, e1002264 (2011).
97. 3C Study Group. Vascular factors and risk of dementia: design of the Three-City Study and baseline characteristics of the study population. *Neuroepidemiology* **22**, 316-325 (2003).
98. Lambert, J. C. *et al*. Genome-wide association study identifies variants at CLU and CR1 associated with Alzheimer's disease. *Nat. Genet.* **41**, 1094-1099 (2009).
99. Patel, A. *et al*. Effects of a fixed combination of perindopril and indapamide on macrovascular and microvascular outcomes in patients with type 2 diabetes mellitus (the ADVANCE trial): a randomised controlled trial. *Lancet* **370**, 829-840 (2007).
100. Harris, T. B. *et al*. Age, Gene/Environment Susceptibility-Reykjavik Study: multidisciplinary applied phenomics. *Am. J. Epidemiol.* **165**, 1076-1087 (2007).
101. Mitchell, B. D. *et al*. The genetic response to short-term interventions affecting cardiovascular function: rationale and design of the Heredity and Phenotype Intervention (HAPI) Heart Study. *Am. Heart J.* **155**, 823-828 (2008).
102. Rampersaud, E. *et al*. The association of coronary artery calcification and carotid artery intima-media thickness with distinct, traditional coronary artery disease risk factors in asymptomatic adults. *Am. J. Epidemiol.* **168**, 1016-1023 (2008).
103. The ARIC Investigators. The Atherosclerosis Risk in Communities (ARIC) Study: design and objectives. The ARIC investigators. *Am. J. Epidemiol.* **129**, 687-702 (1989).
104. Schmidt, R. *et al*. Assessment of cerebrovascular risk profiles in healthy persons: definition of research goals and the Austrian Stroke Prevention Study (ASPS). *Neuroepidemiology* **13**, 308-313 (1994).
105. Schmidt, R., Fazekas, F., Kapeller, P., Schmidt, H. & Hartung, H. P. MRI white matter hyperintensities: three-year follow-up of the Austrian Stroke Prevention Study. *Neurology* **53**, 132-139 (1999).
106. Middelberg, R. P. *et al*. Loci affecting gamma-glutamyl transferase in adults and adolescents show age x SNP interaction and cardiometabolic disease associations. *Hum. Mol. Genet.* **21**, 446-455 (2012).
107. Shock, N. W., et al. Normal Human Aging: The Baltimore Longitudinal Study of Aging. *Superintendent of Documents, U.S. Government Printing Office, Washington, DC 20402.* (1984).
108. Mitchell, P., Smith, W., Attebo, K. & Wang, J. J. Prevalence of age-related maculopathy in Australia. The Blue Mountains Eye Study. *Ophthalmology* **102**, 1450-1460 (1995).
109. Attebo, K., Mitchell, P. & Smith, W. Visual acuity and the causes of visual loss in Australia. The Blue Mountains Eye Study. *Ophthalmology* **103**, 357-364 (1996).
110. Leeder, S. R. *et al*. Low hemoglobin, chronic kidney disease, and risk for coronary heart disease-related death: the Blue Mountains Eye Study. *J. Am. Soc. Nephrol.* **17**, 279-284 (2006).
111. Fried, L. P. *et al*. The Cardiovascular Health Study: design and rationale. *Ann. Epidemiol.* **1**, 263-276 (1991).
112. Heard-Costa, N. L. *et al*. NRXN3 is a novel locus for waist circumference: a genome-wide association study from the CHARGE Consortium. *PLoS Genet.* **5**, e1000539 (2009).
113. Polasek, O. *et al*. Genome-wide association study of anthropometric traits in Korcula Island, Croatia. *Croat. Med. J.* **50**, 7-16 (2009).
114. Rudan, I. *et al*. "10001 Dalmatians:" Croatia launches its national biobank. *Croat. Med. J.* **50**, 4-6 (2009).
115. Rudan, I., Campbell, H. & Rudan, P. Genetic epidemiological studies of eastern Adriatic Island isolates, Croatia: objective and strategies. *Coll. Antropol.* **23**, 531-546 (1999).
116. Rudan, I. *et al*. Effects of inbreeding, endogamy, genetic admixture, and outbreeding on human health: a (1001 Dalmatians) study. *Croat. Med. J.* **47**, 601-610 (2006).
117. Guder, W. G. *et al*. Multicentre evaluation of an enzymatic method for creatinine determination using a sensitive colour reagent. *J. Clin. Chem. Clin. Biochem.* **24**, 889-902 (1986).
118. Balkau, B. An epidemiologic survey from a network of French Health Examination Centres, (D.E.S.I.R.): epidemiologic data on the insulin resistance syndrome. *Rev. Epidemiol. Sante Publique* **44**, 373-375 (1996).
119. Metspalu, A. The Estonian Genome Project. *Drug Dev. Res.* **62**, 97-101 (2004).
120. Nelis, M. *et al*. Genetic structure of Europeans: a view from the North-East. *PLoS One* **4**, e5472 (2009).
121. Aulchenko, Y. S. *et al*. Linkage disequilibrium in young genetically isolated Dutch population. *Eur. J. Hum. Genet.* **12**, 527-534 (2004).
122. Higgins, M. *et al*. NHLBI Family Heart Study: objectives and design. *Am. J. Epidemiol.* **143**, 1219-1228 (1996).

123. Kannel, W. B., Feinleib, M., McNamara, P. M., Garrison, R. J. & Castelli, W. P. An investigation of coronary heart disease in families. The Framingham offspring study. *Am. J. Epidemiol.* **110**, 281-290 (1979).
124. Feinleib, M., Kannel, W. B., Garrison, R. J., McNamara, P. M. & Castelli, W. P. The Framingham Offspring Study. Design and preliminary data. *Prev. Med.* **4**, 518-525 (1975).
125. Splansky, G. L. *et al*. The Third Generation Cohort of the National Heart, Lung, and Blood Institute's Framingham Heart Study: design, recruitment, and initial examination. *Am. J. Epidemiol.* **165**, 1328-1335 (2007).
126. Daniels, P. R. *et al*. Familial aggregation of hypertension treatment and control in the Genetic Epidemiology Network of Arteriopathy (GENOA) study. *Am. J. Med.* **116**, 676-681 (2004).
127. Turner, S. T. *et al*. Influence of genomic loci on measures of chronic kidney disease in hypertensive sibships. *J. Am. Soc. Nephrol.* **17**, 2048-2055 (2006).
128. Rule, A. D. *et al*. A comparison of serum creatinine-based methods for identifying chronic kidney disease in hypertensive individuals and their siblings. *Am. J. Hypertens.* **19**, 608-614 (2006).
129. McEvoy, M. *et al*. Cohort profile: The Hunter Community Study. *Int. J. Epidemiol.* **39**, 1452-1463 (2010).
130. Qi, L. *et al*. Genetic variants at 2q24 are associated with susceptibility to type 2 diabetes. *Hum. Mol. Genet.* **19**, 2706-2715 (2010).
131. Rimm, E. B. *et al*. Prospective study of alcohol consumption and risk of coronary disease in men. *Lancet.* **338**, 464-468 (1991).
132. Hu, F. B. *et al*. Physical activity and television watching in relation to risk for type 2 diabetes mellitus in men. *Arch. Intern. Med.* **161**, 1542-1548 (2001).
133. Manson, J. E. *et al*. Physical activity and incidence of non-insulin-dependent diabetes mellitus in women. *Lancet* **338**, 774-778 (1991).
134. Lin, J., Hu, F. B. & Curhan, G. Serum adiponectin and renal dysfunction in men with type 2 diabetes. *Diabetes Care* **30**, 239-244 (2007).
135. Salvi, E. *et al*. Genomewide association study using a high-density single nucleotide polymorphism array and case-control design identifies a novel essential hypertension susceptibility locus in the promoter region of endothelial NO synthase. *Hypertension* **59**, 248-255 (2012).
136. Gambaro, G. *et al*. Prevalence of CKD in northeastern Italy: results of the INCIPE study and comparison with NHANES. *Clin. J. Am. Soc. Nephrol.* **5**, 1946-1953 (2010).
137. D'Adamo, P. *et al*. Metabonomics and population studies: age-related amino acids excretion and inferring networks through the study of urine samples in two Italian isolated populations. *Amino Acids* **38**, 65-73 (2010).
138. Tepper, B. J. *et al*. Variation in the bitter-taste receptor gene TAS2R38, and adiposity in a genetically isolated population in Southern Italy. *Obesity (Silver Spring)* **16**, 2289-2295 (2008).
139. Sala, C. *et al*. Variation of hemoglobin levels in normal Italian populations from genetic isolates. *Haematologica* **93**, 1372-1375 (2008).
140. Ciullo, M. *et al*. New susceptibility locus for hypertension on chromosome 8q by efficient pedigree-breaking in an Italian isolate. *Hum. Mol. Genet.* **15**, 1735-1743 (2006).
141. Colonna, V. *et al*. Campora: a young genetic isolate in South Italy. *Hum. Hered.* **64**, 123-135 (2007).
142. Ciullo, M. *et al*. Identification and replication of a novel obesity locus on chromosome 1q24 in isolated populations of Cilento. *Diabetes* **57**, 783-790 (2008).
143. Colonna, V. *et al*. Comparing population structure as inferred from genealogical versus genetic information. *Eur. J. Hum. Genet.* **17**, 1635-1641 (2009).
144. Siervo, M. *et al*. Angiogenesis and biomarkers of cardiovascular risk in adults with metabolic syndrome. *J. Intern. Med.* **268**, 338-347 (2010).
145. Sorice, R. *et al*. Association of a variant in the CHRNA5-A3-B4 gene cluster region to heavy smoking in the Italian population. *Eur. J. Hum. Genet.* **19**, 593-596 (2011).
146. Ruggiero, D. *et al*. Genetics of VEGF serum variation in human isolated populations of cilento: importance of VEGF polymorphisms. *PLoS One* **6**, e16982 (2011).
147. Siervo, M. *et al*. Body mass index is directly associated with biomarkers of angiogenesis and inflammation in children and adolescents. *Nutrition* **28**, 262-266 (2012).
148. Traglia, M. *et al*. Heritability and demographic analyses in the large isolated population of Val Borbera suggest advantages in mapping complex traits genes. *PLoS One* **4**, e7554 (2009).
149. Ridker, P. M. *et al*. Rosuvastatin to prevent vascular events in men and women with elevated C-reactive protein. *N. Engl. J. Med.* **359**, 2195-2207 (2008).
150. Chasman, D. I. *et al*. Genetic determinants of statin-induced low-density lipoprotein cholesterol reduction: the Justification for the Use of Statins in Prevention: an Intervention Trial Evaluating Rosuvastatin (JUPITER) trial. *Circ. Cardiovasc. Genet.* **5**, 257-264 (2012).
151. Wichmann, H. E., Gieger, C., Illig, T. & MONICA/KORA Study Group. KORA-gen--resource for population genetics, controls and a broad spectrum of disease phenotypes. *Gesundheitswesen* **67 Suppl 1**, S26-30 (2005).
152. Baumeister, S. E. *et al*. Effect of chronic kidney disease and comorbid conditions on health care costs: A 10-year observational study in a general population. *Am. J. Nephrol.* **31**, 222-229 (2010).
153. Bild, D. E. *et al*. Multi-ethnic study of atherosclerosis: objectives and design. *Am. J. Epidemiol.* **156**, 871-881 (2002).

154. Pattaro, C. *et al*. The genetic study of three population microisolates in South Tyrol (MICROS): study design and epidemiological perspectives. *BMC Med. Genet.* **8**, 29 (2007).
155. Marroni, F., Grazio, D., Pattaro, C., Devoto, M. & Pramstaller, P. Estimates of genetic and environmental contribution to 43 quantitative traits support sharing of a homogeneous environment in an isolated population from South Tyrol, Italy. *Hum. Hered.* **65**, 175-182 (2008).
156. Pattaro, C. *et al*. A meta-analysis of genome-wide data from five European isolates reveals an association of COL22A1, SYT1, and GABRR2 with serum creatinine level. *BMC Med. Genet.* **11**, 41 (2010).
157. Penninx, B. W. *et al*. The Netherlands Study of Depression and Anxiety (NESDA): rationale, objectives and methods. *Int. J. Methods Psychiatr. Res.* **17**, 121-140 (2008).
158. Sullivan, P. F. *et al*. Genome-wide association for major depressive disorder: a possible role for the presynaptic protein piccolo. *Mol. Psychiatry* **14**, 359-375 (2009).
159. Colditz, G. A. & Hankinson, S. E. The Nurses' Health Study: lifestyle and health among women. *Nat. Rev. Cancer.* **5**, 388-396 (2005).
160. Lin, J., Fung, T. T., Hu, F. B. & Curhan, G. C. Association of dietary patterns with albuminuria and kidney function decline in older white women: a subgroup analysis from the Nurses' Health Study. *Am. J. Kidney Dis.* **57**, 245-254 (2011).
161. Johansson, A., Vavruch-Nilsson, V., Edin-Liljegren, A., Sjolander, P. & Gyllensten, U. Linkage disequilibrium between microsatellite markers in the Swedish Sami relative to a worldwide selection of populations. *Hum. Genet.* **116**, 105-113 (2005).
162. Johansson, A., Vavruch-Nilsson, V., Cox, D. R., Frazer, K. A. & Gyllensten, U. Evaluation of the SNP tagging approach in an independent population sample--array-based SNP discovery in Sami. *Hum. Genet.* **122**, 141-150 (2007).
163. Portas, L. *et al*. History, geography and population structure influence the distribution and heritability of blood and anthropometric quantitative traits in nine Sardinian genetic isolates. *Genet. Res. (Camb)* **92**, 199-208 (2010).
164. Biino, G. *et al*. Genetic architecture of hand quantitative ultrasound measures: a population-based study in a Sardinian genetic isolate. *Bone* **46**, 1197-1203 (2010).
165. Pistis, G. *et al*. High differentiation among eight villages in a secluded area of Sardinia revealed by genome-wide high density SNPs analysis. *PLoS One* **4**, e4654 (2009).
166. McQuillan, R. *et al*. Runs of homozygosity in European populations. *Am. J. Hum. Genet.* **83**, 359-372 (2008).
167. Krawczak, M. *et al*. PopGen: population-based recruitment of patients and controls for the analysis of complex genotype-phenotype relationships. *Community Genet.* **9**, 55-61 (2006).
168. Hillege, H. L. *et al*. Urinary albumin excretion predicts cardiovascular and noncardiovascular mortality in general population. *Circulation* **106**, 1777-1782 (2002).
169. Shepherd, J. *et al*. The design of a prospective study of Pravastatin in the Elderly at Risk (PROSPER). PROSPER Study Group. PROspective Study of Pravastatin in the Elderly at Risk. *Am. J. Cardiol.* **84**, 1192-1197 (1999).
170. Shepherd, J. *et al*. Pravastatin in elderly individuals at risk of vascular disease (PROSPER): a randomised controlled trial. *Lancet* **360**, 1623-1630 (2002).
171. Ford, I. *et al*. Reduced glomerular filtration rate and its association with clinical outcome in older patients at risk of vascular events: secondary analysis. *PLoS Med.* **6**, e16 (2009).
172. Hofman, A., Grobbee, D. E., de Jong, P. T. & van den Ouweland, F. A. Determinants of disease and disability in the elderly: the Rotterdam Elderly Study. *Eur. J. Epidemiol.* **7**, 403-422 (1991).
173. Hofman, A. *et al*. The Rotterdam Study: objectives and design update. *Eur. J. Epidemiol.* **22**, 819-829 (2007).
174. Hofman, A. *et al*. The Rotterdam Study: 2010 objectives and design update. *Eur. J. Epidemiol.* **24**, 553-572 (2009).
175. John, U. *et al*. Study of Health In Pomerania (SHIP): a health examination survey in an east German region: objectives and design. *Soz. Praventivmed.* **46**, 186-194 (2001).
176. Volzke, H. *et al*. Cohort profile: the study of health in Pomerania. *Int. J. Epidemiol.* **40**, 294-307 (2011).
177. Tonjes, A. *et al*. Genetic variation in GPR133 is associated with height: genome wide association study in the self-contained population of Sorbs. *Hum. Mol. Genet.* **18**, 4662-4668 (2009).
178. Tonjes, A. *et al*. Association of FTO variants with BMI and fat mass in the self-contained population of Sorbs in Germany. *Eur. J. Hum. Genet.* **18**, 104-110 (2010).
179. Veeramah, K. R. *et al*. Genetic variation in the Sorbs of eastern Germany in the context of broader European genetic diversity. *Eur. J. Hum. Genet.* **19**, 995-1001 (2011).
180. Ridker, P. M. *et al*. Rationale, design, and methodology of the Women's Genome Health Study: a genome-wide association study of more than 25,000 initially healthy american women. *Clin. Chem.* **54**, 249-255 (2008).
181. Guessous, I. *et al*. Caffeine intake and CYP1A2 variants associated with high caffeine intake protect non-smokers from hypertension. *Hum. Mol. Genet.* **21**, 3283-3292 (2012).
182. Guessous, I., Bochud, M., Theler, J. M., Gaspoz, J. M. & Pechere-Bertschi, A. 1999-2009 Trends in prevalence, unawareness, treatment and control of hypertension in Geneva, Switzerland. *PLoS One* **7**, e39877 (2012).
183. Weck, M. N., Stegmaier, C., Rothenbacher, D. & Brenner, H. Epidemiology of chronic atrophic gastritis: population-based study among 9444 older adults from Germany. *Aliment. Pharmacol. Ther.* **26**, 879-887 (2007).

184. Raum, E. *et al*. Changes of cardiovascular risk factors and their implications in subsequent birth cohorts of older adults in Germany: a life course approach. *Eur. J. Cardiovasc. Prev. Rehabil.* **14**, 809-814 (2007).
185. Schottker, B. *et al*. Strong associations of 25-hydroxyvitamin D concentrations with all-cause, cardiovascular, cancer, and respiratory disease mortality in a large cohort study. *Am. J. Clin. Nutr.* **97**, 782-793 (2013).
186. Boger, C. A. *et al*. Effect of ACE and AT-2 inhibitors on mortality and progression to microalbuminuria in a nested case-control study of diabetic nephropathy in diabetes mellitus type 2: results from the GENDIAN study. *Int. J. Clin. Pharmacol. Ther.* **44**, 364-374 (2006).
187. Boger, C. A. *et al*. Association of eGFR-Related Loci Identified by GWAS with Incident CKD and ESRD. *PLoS Genet.* **7**, e1002292 (2011).
188. Wild, P. S. *et al*. Distribution and categorization of left ventricular measurements in the general population: results from the population-based Gutenberg Heart Study. *Circ. Cardiovasc. Imaging* **3**, 604-613 (2010).
189. Wild, P. S. *et al*. A genome-wide association study identifies LIPA as a susceptibility gene for coronary artery disease. *Circ. Cardiovasc. Genet.* **4**, 403-412 (2011).
190. Lucae, S. *et al*. P2RX7, a gene coding for a purinergic ligand-gated ion channel, is associated with major depressive disorder. *Hum. Mol. Genet.* **15**, 2438-2445 (2006).
191. Kloiber, S. *et al*. Variations in tryptophan hydroxylase 2 linked to decreased serotonergic activity are associated with elevated risk for metabolic syndrome in depression. *Mol. Psychiatry* **15**, 736-747 (2010).
192. Kohli, M. A. *et al*. The neuronal transporter gene SLC6A15 confers risk to major depression. *Neuron* **70**, 252-265 (2011).
193. Tayo, B. O. *et al*. Genetic background of patients from a university medical center in Manhattan: implications for personalized medicine. *PLoS One* **6**, e19166 (2011).
194. Winkelmann, B. R. *et al*. Rationale and design of the LURIC study--a resource for functional genomics, pharmacogenomics and long-term prognosis of cardiovascular disease. *Pharmacogenomics* **2**, S1-73 (2001).
195. Heid, I. M. *et al*. Genetic architecture of the APM1 gene and its influence on adiponectin plasma levels and parameters of the metabolic syndrome in 1,727 healthy Caucasians. *Diabetes* **55**, 375-384 (2006).
196. Kollerits, B. *et al*. A common variant in the adiponutrin gene influences liver enzyme values. *J. Med. Genet.* **47**, 116-119 (2010).
197. Pruijm, M. *et al*. Heritability, determinants and reference values of renal length: a family-based population study. *Eur. Radiol.* **23**, 2899-2905 (2013).
